# Supplementary material for: Past speculations of future health technologies: a description of technologies predicted in 15 forecasting studies published between 1986 and 2010
Source: BMJ Open. 2017 Jul 31;7(7):e016206. doi: 10.1136/bmjopen-2017-016206 (PMC5642756; doi:10.1136/bmjopen-2017-016206)
Supplement: Author's manuscript [file bmjopen-2017-016206.draft_revisions.pdf]

BMJ Open

Past speculations of future health technologies: What did they predict?

Journal:	*BMJ Open*
Manuscript ID	bmjopen-2017-016206
Article Type:	Research
Date Submitted by the Author:	31-Jan-2017
Complete List of Authors:	Doos, Lucy; University of Birmingham, Institute of Applied Health Research, NIHR Horizon Scanning Research and Intelligence Centre<br>Packer, Claire; NIHR Horizon Scanning Research and Intelligence Centre, Insitute of Applied Health Research, University of Birmingham<br>Ward, Derek; University of Birmingham, NIHR Horizon Scanning Research and Intelligence Centre, Institute of Applied Health Research<br>Simpson, Sue; University of Birmingham, NIHR Horizon Scanning Research and Intelligence Centre, Institute of Applied Health Research<br>Stevens, Andrew; University of Birmingham, Institute of Applied Health Research
**Primary Subject Heading**:	Public health
Secondary Subject Heading:	Health policy
Keywords:	Forecasting, prediction, health technology, innovation, healthcare

SCHOLARONE™
Manuscripts

For peer review only

Past speculations of future health technologies: What did they predict?

Lucy Doos[1], Claire Packer[1], Derek Ward[1], Sue Simpson[1], Andrew Stevens[2]

[1]NIHR Horizon Scanning Research and Intelligence Centre, Institute of Applied Health Research, University of Birmingham

[2] Institute of Applied Health Research, University of Birmingham

Corresponding author:

Dr Lucy Doos

Institute of Applied Health Research,

Public Health Building,

University of Birmingham, Edgbaston,

Birmingham, B15 2TT, UK.

Email: l.doos@bham.ac.uk

Phone: (+44)121 414 8594

Keywords: Forecasting, prediction, health technology, innovation, healthcare

Word count: 3,553

For peer review only

ABSTRACT

Objective: To describe and classify health technologies predicted in forecasting studies.

Design and methods: Description of the results of previously identified forecasting studies with a classification of health technologies according to their type, purpose and clinical use; relating these to the original purpose and timing of the forecasting studies.

Data sources: Health-related topics and technologies predicted in 15 forecasting studies identified in a previously published systematic review.

Main outcome measure: Outcomes related to i) each forecasting study including country, year, intention and forecasting methods used and ii) the predicted technologies including technology type, purpose, targeted clinical area and forecast timeframe.

Results: Of the 896 identified health-related topics and technologies, 685 (76.5%) were health technologies with an explicit or implied health application and included in our study. Of these, 19.1% were diagnostic or imaging tests, 14.3% devices or biomaterials, 12.6% information technology systems, eHealth or mHealth, and 12% drugs. The majority of the technologies were intended to treat or manage disease (38.1%) or diagnose or monitor disease (26.1%). The most frequent targeted clinical areas were infectious diseases followed by cancer, circulatory and nervous system disorders. The most frequent technology types were: for infectious diseases - prophylactic vaccines (45.8%); for cancer - drugs (40%); for circulatory disease - devices and biomaterials (26.3%), and for diseases of the nervous system - equally devices and biomaterials (25%) and regenerative medicine (25%). The mean timeframe for forecasting was 11.6 years (range 0-33 years, median=10, SD= 6.6). The forecasting timeframe significantly differed by technology type (p=0.002), the intent of the forecasting group (p<0.001), and the methods used (p<001).

For peer review only - http://bmjopen.bmj.com/site/about/guidelines.xhtml

Conclusion: Our study provided detailed description and classification of predicted health-related technologies from prior forecasting studies. This is crucial in preparing healthcare systems for adopting new innovations. Further work is needed to test the accuracy of predictions made.

Strengths and limitations of the study

- The study is a comprehensive description and classification of health-related technologies predicted in 15 forecasting studies identified in a previously published systematic review.
- The study summarises the forecast technologies by type e.g. medical devices, diagnostic tests or drugs; by clinical purpose e.g. prevention, diagnosis or treatment and by targeted clinical area by ICD10 chapter; and relates this to the forecasting purpose and timeframe.
- The analysis and the health-related technologies identified are constrained by the methods used in the forecasting studies for example by the areas of interest of the experts involved.
- Although the study highlights the identified technologies, the retrospective accuracy of the studies remains to be researched.

For peer review only

For peer review only - http://bmjopen.bmj.com/site/about/guidelines.xhtml

INTRODUCTION

People are in general living longer and surviving previously life threatening illnesses. A proportion of this effect is attributed to the use of novel health interventions and technologies, such as new vaccines to prevent disease; new diagnostic tests to diagnose diseases earlier and with improved accuracy; and new drugs, devices and surgical procedures to treat diseases more effectively. Technological advances and innovation are leading to new health interventions becoming available to healthcare markets at an increasing speed; these often cost more than current alternatives and significantly affect the cost of healthcare services and delivery.[1,2] To ensure that patients can access the most effective and cost effective interventions, and because many health systems work within constrained budgets, healthcare systems need to be prepared to respond to new developments. A crucial step in such preparation is the accurate identification and forecasting of likely significant technological healthcare developments.[3,4]

Forecasting is widely used for planning and strategic decision making around industrial and economic development at organisational, regional and national levels.[5] Within healthcare, forecasting can be used to build strategies for supporting innovators and companies to develop new interventions; to plan future research programmes and delivery of healthcare; and to prepare health services and personnel to respond to technological developments of benefit to patients and service provision. Health technology forecasting uses various methods such as Delphi surveys and reviews of the literature[6,7,8] to identify emerging health technologies that are intended to address important unmet healthcare needs or that need additional evaluation, and to analyse their potential impact on patients and health systems.[4] To get the maximum benefit from forecasting, it is important that the methods used to identify emerging interventions and technologies are appropriate for the aims and timeframe of the forecasting exercise and also as accurate as possible.

In a recent systematic review[9] we found 15 forecasting studies with 3 to 20 year timeframes that identified emerging health-related topics and technologies from 12 high income countries, including six from the United Kingdom (UK). Here we describe and classify the health technologies predicted in the forecasting studies

For peer review only - http://bmjopen.bmj.com/site/about/guidelines.xhtml

according to their type, purpose and clinical use. We relate these to the forecast time period and develop an overview of the technological and clinical frontiers of innovation in health and healthcare provision.

METHODS

See table 1 for a full list of the 15 studies identified in our prior systematic review. We extracted all forecast topics from the 15 forecasting studies using a standardised set of data for each of these predicted topics and on the source study. Data related to the forecast topics included the name of the topic as written in the forecasting reports and papers, and the predicted forecast timeframe. Data related to the study included the country or region of the forecast, year the study was undertaken, its intention and remit, the study forecasting timeframe, and the number and type of forecasting methods used.

Identification and inclusion of health technologies

For final inclusion and analysis, topics had to be health technologies as defined by the National Institute of Health Research (NIHR) Health Technology Assessment (HTA) programme[10]: "*any method used to promote health; prevent and treat disease; and improve rehabilitation or long-term care. They are not confined to new drugs and include any intervention used in the treatment, prevention or diagnosis of disease*". We also included topics that related to the recognition of, or change in, health states or emotions, or health-related behaviours. All topics needed to have an explicit or implied health-related application or identified patient group for final inclusion. Topics were excluded if they related exclusively to foods, plants, animals, insects, new sources of fuel or energy, environmental contamination, legislation or health insurance.

Two authors (CP and LD) independently scrutinised each identified topic and applied the inclusion and exclusion criteria. Where there was insufficient information on a topic to make the inclusion/exclusion decision, we undertook brief and very limited searches on the Internet to find additional information.

For peer review only - http://bmjopen.bmj.com/site/about/guidelines.xhtml

All topics classed as a health technology with an explicit or implied health-related application, were subsequently coded based on their technology type, intended purpose, and targeted clinical use:

Technology type: we used broad categories: assistive devices and rehabilitation aids; devices and biomaterials; diagnostic tests and imaging; drugs; information technology (IT) systems, electronic health systems (eHealth) and mobile health (mHealth); medical equipment; non-surgical therapy; organisational programmes; prophylactic vaccines; population programmes; regenerative medicine approaches; and therapeutic procedures.

Technology purpose: we used categories that relate to the intended point on the patient pathway: health promotion and the prevention of disease; diagnosis and monitoring of disease; treatment and management of symptoms and disease; and rehabilitation. We supplemented these with two groupings that categorised technologies that support the provision of care i) supporting patients receiving care and designed to be used by individuals (patients, caregivers or healthcare professionals) and ii) supporting the provision of care and/or increasing service efficiency.

Targeted clinical area (specialty): we used the international statistical classification of diseases and related health problems 10th revision (ICD10)[11] chapter headings relating to diseases and other morbid conditions (chapters I-XVII); symptoms, signs and abnormal findings (chapter XVIII); injuries, poisoning and other consequences of external causes (chapter XIX); external causes of diseases and morbid conditions chapter XX); and factors influencing health status and contact with health services for people not currently sick (chapter XXI). Chapter XXI includes contraception, technologies used in population screening, health promotion and disease prevention, and general rehabilitation.

All topics with disputed inclusion, exclusion or categorisation were initially discussed between two authors (LD and CP) and disagreements resolved where possible. Where disagreements remained, topics were discussed with the other authors as a group and the final categorisation agreed through consensus.

For peer review only - http://bmjopen.bmj.com/site/about/guidelines.xhtml

Technology forecast timeframe: for each health-related technology forecast we calculated the difference in years between each technology's predicted year of impact as identified in the original study, and the year when the prediction was made, i.e. the year of the original study. Technology forecasts were classified as short term if their forecasting timeframe was less than 3 years, short to medium term if it was 3-10 years, medium to long term if it was 11-20 years and long term for those with timeframes of more than 20 years. Although the original systematic review excluded forecasting studies which exclusively adopted a short time frame of less than 3 years, some studies with longer time frames also provided predictions with shorter time frames, and these were included in the analysis.

Data analysis

We analysed the data using IBM SPSS statistics (version 22) for Windows. We present descriptive analyses as means and standard deviations (SD) for normally distributed continuous variables. Statistically significant differences were determined using ANOVA for continuous normally distributed data and X^2 for dichotomous variables.

RESULTS

We identified 896 predicted topics from the 15 forecasting studies. Of these, we judged that 685 (76.5%) were health-related technologies with an explicit or implied health-related application. The most frequently excluded topics were those that described advances in the underpinning scientific knowledge (8.7%), such as determination of whole human DNA base sequence and identifying genetic links to diseases. A full list of the included health-related technologies is attached as an online appendix.

Of the 685 health-related technologies, 52.3% were forecast from 6 studies by governmental organisations (such as the UK Department of Trade and Industry[12]), 21.9% by commercial/consultancy organisations (such as British Telecommunications plc[13]), 14.5% by policy planning groups (such as Loveridge et al 1995[6]), 8.9% by research groups (such as those by Stevens et al 1997[7]), and 2.5% by non-profit organisations (such as Institute of the Future[14]). Looking at the

For peer review only - http://bmjopen.bmj.com/site/about/guidelines.xhtml

For peer review only

purpose of forecasting, our data showed that two thirds (68%) of the health technologies were forecast for policy planning purposes, 18.1% for research purposes and 13.9% for commercial purposes.

Nearly half (48.9%) of the identified technologies were from 6 UK-based studies and reports, 15.0% were from Japan, 11.7% from the Netherlands, 11.4% from the United States of America (USA) and 8.0% from Canada. The remainder were from a homogenous group of developing countries and South Africa (3.8%), other European countries (0.9%) and China (0.3%).

Technology type

Of the 685 health technologies forecast, 19.1% were diagnostic tests and imaging technologies, such as advanced ultrasound imaging systems and molecular diagnosis; 14.3% were devices and biomaterials, such as tissue engineered devices and drug impregnated devices; 12.6% were IT systems, eHealth and mHealth, such as electronic prescriptions and telemedicine; 12.0% were drugs, such as new anaesthetic vapours and electronically activated drugs; and 11.7% were regenerative medicine approaches, such as gene therapy for diabetes and widespread use of gene therapy for familial hypercholesterolemia (Table 2 and online appendix). Regenerative medicine approaches were the most commonly forecast technologies (23.2%) by studies with a commercial intent while diagnostic tests and imaging were the most commonly forecast technologies by studies with policy planning and research intentions (20.6% and 23.4% respectively) ($p<0.001$).

Technology purpose

Of the 685 health technologies included in the forecasting reports, 38.1% were for the treatment and management of symptoms and disease, such as development of effective treatment for amyotrophic lateral sclerosis and the practical use of gene therapy for genetic disorders; 21.6% for the diagnosis and monitoring of disease, for example a diagnostic bio-chip for cancer and the further development of 3D imaging techniques; 13% for health promotion and the prevention of disease, such as the first effective vaccine of HIV and DNA vaccines for AIDS, malaria, hepatitis B and certain cancers; 10.2% for supporting the provision of care, for example tele-monitoring and teleconsultation; 8.0% for supporting patients receiving care, such as smart pill

For peer review only - http://bmjopen.bmj.com/site/about/guidelines.xhtml

bottles to remotely monitor medication use and thought-controlled robots for personal healthcare; and 4.7% for rehabilitation, for example artificial legs and robotic prosthetics.

Technologies for treatment and disease management were the most common regardless of the intention of the study: 35.8% among studies with a commercial intent, 35.8% for policy planning and 48.4% for research.

Targeted clinical area

Using the ICD10 codes to define the targeted clinical area, just over half of the health technologies (50.4%) were for diseases and conditions that were not specified or that could not be coded or that crossed multiple specialities. Of the 340 health technologies that could be coded to a specific ICD10 chapter, the top five targeted clinical areas were: 1) infectious and parasitic diseases ICD-I (14.1%), such as development of vaccines for AIDS and vaccines for malaria, 2) neoplasms ICD-II (11.8%), such as expanding metal stents for oesophageal cancer and diagnostic bio-chip for cancer, 3) diseases of the circulatory system ICD-IX (11.2%), such as artificial muscles in replace of heart transplants and implantable vascular stents, 4) diseases of the nervous system ICD-VI (10.6%), such as artificial brain cells and artificial peripheral nerves and 5) factors influencing health status and contact with health services ICD-XXI (10.3%), such as a personal wearable health monitor and extension of average lifespan to over 100.

Congenital malformations, deformations and chromosomal abnormalities ICD-XVII and symptoms, signs and abnormal clinical and laboratory findings, not elsewhere classified ICD- XVIII had the fewest forecast technologies (0.3% each).

Table 3 shows the technology types and purposes for the forecast technologies within the five most frequent targeted clinical areas. The most frequent technology type forecast for infectious and parasitic diseases were prophylactic vaccines (45.8%); for cancer were drugs (40%); for diseases of the circulatory system were devices and biomaterials (26.3%); for diseases of the nervous system were devices and biomaterials (25%) and regenerative medicine (25%); and for factors influencing health status and contact with health services including screening and rehabilitation,

For peer review only - http://bmjopen.bmj.com/site/about/guidelines.xhtml

devices and biomaterials (31.4%). The most frequent technology purpose was health promotion and disease prevention for both infectious and parasitic diseases (ICD-I) (60.4%) and factors influencing health status and contact with health services including screening and rehabilitation (ICD-XXI) (51.4%). Technologies for treatment and disease management were most common for cancer (55.0%), diseases of the circulatory system (65.8%) and nervous system disorders (61.1%).

Technology forecast timeframe

Data was available to calculate the prediction timeframe for 607 of the identified health technologies. The mean timeframe for forecasting predictions was 11.6 years (range 0-33 years, median=10, SD=6.6). There was a significant difference in the mean prediction timeframe by technology type (p=0.002), with non-surgical therapies having the shortest mean forecast timeframe (9.07 years) and population health programmes having the longest mean forecast (14.53 years) (Table 4). There were no statistically significant differences in the mean duration of the technology prediction timeframe by the technology purpose and we were not able to detect any particular trend in the technology types or purposes of forecast over time.

There was a statistically significant difference in the forecast timeframe by the type of group undertaking the forecasting (p<0.001): 58% of the long-term forecasts (>20 years) were made by policy planning groups and 51% of short-term forecasts (<3 years) were by research groups.

Technologies forecast by studies with research intentions had the shortest mean forecast timeframe (4.49 years) while those for policy making had the longest timeframe (14.16 years) (p<0.001). Similarly, technologies forecast by researchers had significantly shorter mean timeframes (3.56 years) compared to those made by governmental agencies which had the longest mean forecast timeframes (13.42 years) (P<0.001).

There was a statistically significant difference in the mean forecast timeframe when considering the forecasting method (p<0.001) (Figure 1). Technologies forecast using methods that included creativity-based methods which require brainstorming, such as scenario building, had a significantly longer forecasting timeframe (13.5

For peer review only - http://bmjopen.bmj.com/site/about/guidelines.xhtml

years) compared to those did not include creativity-based methods such as literature reviews (9.7 years) ($p<0.001$). On the other hand technologies forecast using methods that included evidence-based methods such as literature reviews and evidence from health technology assessment (HTA) organisations, had significantly shorter forecasting timeframes (8.2 years) than those that did not include evidence-based methods (10.8 years) ($p<0.001$).

DISCUSSION

Being aware of forthcoming developments in healthcare and preparing for the future is essential for health policy makers, whether developing healthcare policy, directing future research or commissioning health services. We found that more than two thirds of the identified technologies were forecast for policy planning purposes and over half were forecast by governmental organisations, and that the majority of long-term forecasts were made by policy planning groups as opposed to research groups. This finding supports the premise that the majority of forecasting is carried out for service preparedness and long-term planning, and strategic decision making.[5]

Our study identified and classified a number of important health-related technologies, which health services may have already had to manage or will have to do so in the future. Our finding that nearly 60% of the technologies were either for the treatment and management of symptoms and disease or for the diagnosis and monitoring of disease can perhaps be explained by the methods used for forecasting. Many of the studies involved soliciting the opinion of experts and, although it is not possible to be certain about the areas of interest of those involved, their expertise may have made them more likely to focus on diagnosis and treatment than, for example, organisational programmes or rehabilitation. Regenerative medicine approaches were the most commonly forecast technologies by studies with a commercial intent.

The four clinical areas with the greatest number of forecast health technologies: infectious diseases, cancer, and circulatory and nervous system diseases and disorders, include many of the major causes of death and disability in countries today. Again we are not able to determine if this merely reflects the interests of the groups carrying out the forecasting and their areas of expertise or whether it truly

For peer review only - http://bmjopen.bmj.com/site/about/guidelines.xhtml

represents the technology innovation frontier. It is understandable that the forecasts for infectious disease included many prophylactic vaccines as these have proven to be a cost effective preventive strategy for their prevention.[15] In cancer, forecasting included both drugs and diagnostic tests and imaging, perhaps reflecting increasing targeting in novel cancer therapies.[16,17] Several forecasts of regenerative medicine approaches for nervous system diseases and disorders is also interesting, with many of these diseases not currently curable or effectively treated.[18,19]

Our finding that forecasts which included creativity-based methods such as scenario building and analysis had, on the whole, longer forecast timeframes than studies that included evidence-based methods may have a rationale. Evidence-based methods require there to be research evidence on technologies which will therefore probably be nearer to any predicted impact. In contrast, creativity-based methods require experts to think more widely about what the future could be, and are not therefore constrained by what is actually being currently researched and published. This may also explain our finding that just over half of the short-term forecasts were made by research groups who may be more likely to use published evidence to inform their deliberations.

We believe that this is the first study to comprehensively identify and summarise health-related technologies predicted in past forecasting projects and to categorise and describe them. Although as many predicted topics as possible were included, we did exclude a substantial number. This was often because we could not envisage a future health application or because there was insufficient information to make a judgement (and many topics had very sparse titles or descriptive information). By excluding topics in this way, we may have excluded some that would have had an explicit health application within a reasonable timeframe.

Our description and classification of predicted health-related technologies from prior forecasting studies provides an overview of the technological and clinical frontiers of innovation in health and health care provision. To complete our evaluation of the accuracy of previous forecasting predictions and the forecasting methods used, the accuracy of the predictions made should also be assessed. This may be difficult across all technology types and clinical areas, but possible by limiting the evaluation

For peer review only

For peer review only - http://bmjopen.bmj.com/site/about/guidelines.xhtml

to specific clinical areas and/or technology types or to those topics with more obvious health applications and timeframes that have already occurred.

CONTRIBUTERS

All authors have directly participated in the planning and execution of the study. LD extracted the study data. LD and CP independently reviewed the technologies to be included. LD and CP drafted the paper and DW, SS and AS critically revised the manuscript. All authors agreed the final version of the paper. All authors had full access to all of the data.

DISCLAIMER

This report presents independent research funded by the National Institute for Health Research (NIHR). The views expressed in this publication are those of the authors and not necessarily those of the National Health Service, the NIHR or the Department of Health.

COMPETING INTERESTS

The authors report that they do not have any conflicts of interest.

FUNDING

At the time of the study LD, CP, DW and SS were funded by the United Kingdom's National Institute for Health Research (NIHR), and AS was partly funded by the UK NIHR.

DATA SHARING

A full list of health technologies included in the study is available in a supplemental document submitted with this article. More details on data are available from the corresponding author at l.doos@bham.ac.uk.

For peer review only - http://bmjopen.bmj.com/site/about/guidelines.xhtml

REFERENCES

1. Herndon JH, Wang RH, Bozic KH. Healthcare technology and technology assessment. *Eur Spine J* 2007;16:1293–1302.
2. Beller GA. New technology and the cost of health care. *J Nucl Cardiol* 2005;12:515-7.
3. Simpson S, Hyde C, Cook A, et al. Assessing the accuracy of forecasting: applying standard diagnostic assessment tools to a health technology early warning system. Int J Technol Assess Health Care 2004;20(3):381-4.
4. Lerner JC, Robertson DC, Goldstein SM. Case studies on forecasting for innovative technologies: Frequent revisions improve accuracy. *Health Aff* 2015; 34(2):311-318.
5. Pankratova N, Bubnov RV. Solving strategic problems of the future on the basis of foresight methodology. *EPMA Journal* 2014;5(Suppl 1) A65-66.
6. Loveridge D, Georghiou L, Nedeva M. *United Kingdom Technology Foresight Programme: Delphi Survey*. A report by the Office of Science and Technology. Manchester, UK: Policy Research in Engineering, Science and Technology (PREST), 1995.
7. Stevens A, Robert N, Gabbay J. Identifying new health care technologies in the United Kingdom. *Int J Technol Assess Health Care* 1997;13:59–67.
8. Tremblay M, Yiu SH. Horizon Scan of Diagnostic Technologies-Technology push or treatment pull? Report published by Rainmaker Publications 2006. http://www.foresightfordevelopment.org/sobipro/55/365-horizon-scan-of-diagnostic-technologies-technology-push-or-treatment-pull (Accessed 2June 2014).
9. Doos L, Packer C, Ward D, Simpson S, Stevens A. Past speculations of the future: A review of the methods used for forecasting emerging health technologies. BMJ Open 2016 6:e010479 doi:10.1136/bmjopen-2015-010479.
10. The National Institute for Health Research Health Technology Assessment (NIHR HTA) programme. http://www.nets.nihr.ac.uk/programmes/hta/remit (Accessed 4 July 2016).
11. World Health Organisation (WHO). International statistical classification of diseases and related health problems. - 10th revision (ICD-10), Fifth edition,

For peer review only - http://bmjopen.bmj.com/site/about/guidelines.xhtml

2016. http://apps.who.int/classifications/icd10/browse/2016/en (Accessed 5 July 2016).

12. Rosin RD, Kemp CR. Operating theatre of the year 2010. Report by Department of Trade and Industry. DTI, 1999.
13. BT calendar 2005. Personal communication with the editor Ian Pearson.
14. Institute of the Future_HC2020 _The future of science, technology & wellbeing. http://www.iftf.org/our-work/health-self/health-horizons/hh2010-the-future-of-science-tech-and-well-being/ (Accessed 21 March 2015).
15. Berzofsky JA, Ahlers JD, Janik J, et al. Progress on new vaccine strategies against chronic viral infections. *J Clin Invest* Aug 2004;114:4: 450:462.
16. Cahill E, Scapolo F. The Future Project Technology Map. A report by European Commission Directorate General, December 1999.
17. Begley CG, Ellis LM. Raise standards for preclinical cancer research. *Nature* 29 March 2012;483:531-533.
18. Lindvall O, Kokaia Z, Martinez-Serrano A. Stem cell therapy for neurodegenerative disorders-How to make it work. *Nat Med* July 2004 (10); S42-S50.
19. Aboody K, Capela A, Niazi N, et al. Translating stem cell studies to the clinic for CNS repair: Current state of the art and the need for a rosetta stone. *Neuron* May 26, 2011;70:597-613.

For peer review only

For peer review only - http://bmjopen.bmj.com/site/about/guidelines.xhtml

Table 1: The 15 forecasting studies identified in the prior systematic review

Study name or first author	Year of forecast	Country
Dutch Steering Committee on Future Health Scenarios (STG) 1988	1986	Netherlands
Spiby, 1988	1988	UK
Loveridge et al 1995	1994	UK
Stevens et al, 1997	1995	UK
Karim, National Research and Technology Foresight Project, 1999	1996	South Africa
Operating theatre of the year 2010; Department of Trade and Industry report 1999	1996	UK
Cahill and Scapolo, 1999	1998	Europe
Daar et al, 2002	2002	Developing countries
Technology foresight towards 2020_China	2003	China
British Telecommunications (BT) calendar	1997 & 2005	United Kingdom (UK)
Tremblay and Yiu, 2006	2006	Canada
Food and Drug Administration (FDA) surveys	1998 & 2008	United States of America (USA)
Institute of the Future, 2009	2009	USA
Science & Technology Foresight Survey, 2010	Every 5 years from 1971, latest survey 2009-10	Japan
UK technology and innovation futures for the 2020s, 2010	2010	UK

Source: Doos et al 2016[9]

For peer review only

Table 2: Health technologies by technology type

**Technology type**	**Number**	**%**
Diagnostic tests and imaging	131	19.1
Devices and biomaterials	98	14.3
IT systems, eHealth and mHealth	86	12.6
Drugs (not prophylactic vaccines)	82	12.0
Regenerative medicine	80	11.7
Medical equipment	37	5.4
Therapeutic procedures	36	5.3
Unknown	33	4.8
Organisational programmes	26	3.8
Prophylactic vaccines	24	3.5
Population programmes	24	3.5
Non-surgical therapy	15	2.2
Other	9	1.3
Assistive devices	4	0.6
**Total**	**685**	**100.0**

For peer review only

For peer review only - http://bmjopen.bmj.com/site/about/guidelines.xhtml

Table 3: Technology type and purpose for the five most common ICD_10 chapter headings

**Technology type**	Infectious disease ICD_I	Cancer ICD_II	Circulatory disorders ICD_IX	Nervous system ICD_VI	Health status, screening, rehab ICD_XXI	**Total**
Assistive devices	-	-	-	-	3 (8.6)	**3 (1.5)**
Diagnostic tests and imaging	5 (10.4)	12 (30.0)	7 (18.4)	6 (16.7)	2 (5.7)	**32 (16.2)**
Devices and biomaterials	3 (6.3)	1 (2.5)	10 (26.3)	9 (25.0)	11 (31.4)	**34 (17.3)**
IT systems, eHealth and mHealth	-	-	-	1 (2.8)	3 (8.6)	**4 (2.0)**
Drugs (not prophylactic vaccines)	10 (20.8)	16 (40.0)	7 (18.4)	2 (5.6)	5 (14.3)	**40 (20.3)**
Regenerative medicine	-	2 (5.0)	7 (18.4)	9 (25.0)	-	**18 (9.1)**
Medical equipment	2 (4.2)	-	-	1 (2.8)	1 (2.9)	**4 (2.0)**
Therapeutic procedures	1 (2.1)	1 (2.5)	3 (7.9)	4 (11.1)	-	**9 (4.6)**
Organisational programmes	3 (6.3)	-	-	-	1 (2.9)	**4 (2.0)**
Prophylactic vaccines	22 (45.8)	-	-	-	1 (2.9)	**23 (11.7)**
Population programmes	1 (2.1)	2 (5.0)	2 (5.3)	1 (2.8)	3 (8.6)	**9 (4.6)**
Non-surgical therapy	-	2 (5.0)		1 (2.8)	-	**3 (1.5)**
Other/ unknown	1 (2.1)	4 (10.0)	2 (5.3)	2 (5.6)	5 (14.3)	**14 (7.1)**
**Total**	**48 (100%)**	**40 (100%)**	**38 (100%)**	**36 (100%)**	**35 (100%)**	**197 (100%)**
**Technology purpose**						
Diagnosis & monitoring of disease	7 (14.6)	12 (30.0)	8 (21.1)	7 (19.4)	1 (2.9)	**35 (17.8)**
Health promotion & disease prevention	29 (60.4)	5 (12.5)	5 (13.2)	1 (2.8)	18 (51.4)	**58 (29.4)**

For peer review only - http://bmjopen.bmj.com/site/about/guidelines.xhtml

Treatment & disease management	11 (22.9)	22 (55.0)	25 (65.8)	22 (61.1)	1 (2.9)	**81 (41.1)**
Rehabilitation	0 (0.0)	0 (0.0)	0 (0.0)	4 (11.1)	15 (42.9)	**19 (9.6)**
Other and unknown	1 (2.1)	1 (2.5)	0 (0.0)	2 (5.6)	0 (0.0)	**4 (2.0)**
**Total**	**48 (100%)**	**40 (100%)**	**38 (100%)**	**36 (100%)**	**35 (100%)**	**197 (100%)**

For peer review only

For peer review only - http://bmjopen.bmj.com/site/about/guidelines.xhtml

Table 4: Mean technology prediction timeframe (years) by technology type*

Technology type	Number	Mean timeframe Years (SD)
Non-surgical therapy	14	9.07 (5.7)
Diagnostic tests and imaging	113	10.11 (6.0)
Drugs (not prophylactic vaccines)	75	10.31 (6.3)
Therapeutic procedures	31	10.45 (8.2)
Organisational programmes	25	10.48 (5.3)
IT systems, eHealth and mHealth	77	11.08 (5.5)
Devices and biomaterials	88	11.93 (6.8)
Assistive devices	3	12.33 (17.9)
Other/ unknown	40	13.35 (7.2)
Medical equipment	36	13.53 (2.7)
Regenerative medicine	73	13.58 (7.4)
Prophylactic vaccines	13	14.00 (6.3)
Population programmes	19	14.53 (5.9)
**Total**	**607**	**11.59 (6.6)**

*p<0.002

For peer review only

Figure 1: Mean forecast timeframe by methods of forecasting*

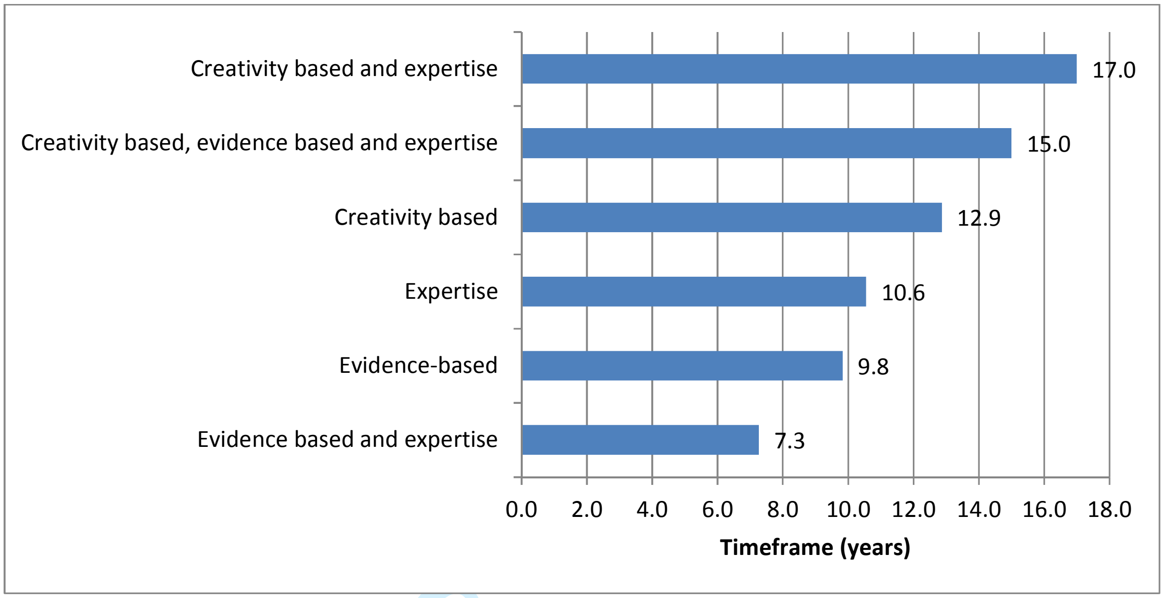

*p<0.001

For peer review only - http://bmjopen.bmj.com/site/about/guidelines.xhtml

Appendix1: List of identified healthcare technologies

Report name (year)	Intention of the study	Technology Name	Tech. time frame	Purpose of the technology	Specialty	Technology type
BT Calendar 1997	Commercial	Artificial muscle use in place of heart transplants	1	Treatment and management of symptoms and disease	IX - circulation	Regenerative medicine
BT Calendar 1997	Commercial	Telemed link to home with home blood test kit	1	Diagnosis and monitoring of disease	Other - generic	IT systems, eHealth and mHealth
BT Calendar 1997	Commercial	Artificial intelligence based elderly and handicapped support devices	1	Rehabilitation	XXI - screening, rehab	Assistive devices
BT Calendar 1997	Commercial	Artificial pancreas	1	Treatment and management of symptoms and disease	XI - GI tract, dental health	Regenerative medicine
BT Calendar 1997	Commercial	Artificial blood	3	Treatment and management of symptoms and disease	III - blood	Regenerative medicine
BT Calendar 1997	Commercial	Disability assistance devices using thought recognition	3	Rehabilitation	XXI - screening, rehab	Assistive devices
BT Calendar 1997	Commercial	Artificial ears	3	Rehabilitation	VIII - ear	Regenerative medicine
BT Calendar 1997	Commercial	Electronic implants used to stimulate muscles in disabled people	5	Rehabilitation	XIII - musculoskeletal	Devices and biomaterials
BT Calendar 1997	Commercial	Home (health) diagnostic systems, daily/real time check-up	1	Diagnosis and monitoring of disease	Other - generic	IT systems, eHealth and mHealth
BT Calendar 1997	Commercial	Virtual Reality based exercisers in homes	1	Health promotion and prevention of disease	XXI - screening, rehab	IT systems, eHealth and mHealth
BT Calendar 1997	Commercial	Synthetic retinal implants for simple vision	8	Rehabilitation	VII - eye	Regenerative medicine
BT Calendar 1997	Commercial	Personal wearable health monitor	6	Health promotion and prevention of disease	XXI - screening, rehab	IT systems, eHealth and mHealth
BT Calendar 1997	Commercial	Artificial heart	13	Treatment and management of symptoms and disease	IX - circulation	Regenerative medicine

For peer review only - http://bmjopen.bmj.com/site/about/guidelines.xhtml

Appendix1: List of identified healthcare technologies

BT Calendar 1997	Commercial	Genetic screening widely used	10	Health promotion and prevention of disease	XXI - screening, rehab	Population programmes
BT Calendar 1997	Commercial	Fine particle beam gene engineering	12	Treatment and management of symptoms and disease	Other - generic	Regenerative medicine
BT Calendar 1997	Commercial	Artificial senses, sensors directly stimulating nerves	15	Rehabilitation	XXI - screening, rehab	Devices and biomaterials
BT Calendar 1997	Commercial	Prevention of cancer	16	Health promotion and prevention of disease	II - cancers	Population programmes
BT Calendar 1997	Commercial	Custom foods for particular medical conditions will exist.	13	Treatment and management of symptoms and disease	Other - generic	Other
BT Calendar 1997	Commercial	Genetic programs to enhance human well-being	23	Health promotion and prevention of disease	V - mental health	Population programmes
BT Calendar 1997	Commercial	Artificial lungs	18	Treatment and management of symptoms and disease	X - respiratory	Regenerative medicine
BT Calendar 1997	Commercial	Artificial kidneys	18	Treatment and management of symptoms and disease	XIV - genitourina ry	Regenerative medicine
BT Calendar 1997	Commercial	Artificial brain cells	20	Treatment and management of symptoms and disease	VI - nervous	Regenerative medicine
BT Calendar 1997	Commercial	Artificial liver	23	Treatment and management of symptoms and disease	XI - GI tract, dental health	Regenerative medicine
BT Calendar 1997	Commercial	Extension of average lifespan to over 100	23	Health promotion and prevention of disease	XXI - screening, rehab	Unknown
BT Calendar 1997	Commercial	Artificial brain implants	28	Treatment and management of symptoms and disease	VI - nervous	Regenerative medicine
BT Calendar 1997	Commercial	Artificial peripheral nerves	28	Treatment and management of symptoms and disease	VI - nervous	Regenerative medicine
BT Calendar 1997	Commercial	Artificial legs	33	Rehabilitation	XXI - screening, rehab	Unknown

For peer review only - http://bmjopen.bmj.com/site/about/guidelines.xhtml

Appendix1: List of identified healthcare technologies

BT Calendar 1997	Commercial	Fully functional artificial eyes	33	Treatment and management of symptoms and disease	VII - eye	Regenerative medicine
BT Calendar 2005	Commercial	laser activated drug capsules	3	Treatment and management of symptoms and disease	Other - generic	drugs (not prophylactic vaccines)
BT Calendar 2005	Commercial	ultrasound or radio activated capsules	3	Treatment and management of symptoms and disease	Other - generic	drugs (not prophylactic vaccines)
BT Calendar 2005	Commercial	blood analysis chips	3	Diagnosis and monitoring of disease	Other - generic	Diagnostic tests and imaging
BT Calendar 2005	Commercial	e-Baybies electronic conception	5	Health promotion and prevention of disease	XXI - screening, rehab	Unknown
BT Calendar 2005	Commercial	Instant electronic identification of pathogens	5	Diagnosis and monitoring of disease	I - infections	Diagnostic tests and imaging
BT Calendar 2005	Commercial	Online surgeries dominate first line medical care	5	Supporting the provision of care and/or increasing service efficiency	Other - generic	IT systems, eHealth and mHealth
BT Calendar 2005	Commercial	active skin	5	Unknown	XII - skin	Unknown
BT Calendar 2005	Commercial	Cyber-drugs (electronically activated drugs)	5	Treatment and management of symptoms and disease	Other - generic	Drugs (not prophylactic vaccines)
BT Calendar 2005	Commercial	automated pain relief for soldiers	5	Treatment and management of symptoms and disease	XIX - injury	Devices and biomaterials
BT Calendar 2005	Commercial	bacteria in toothpaste to attack plaque	5	Health promotion and prevention of disease	XI - GI tract, dental health	Non-surgical therapy
BT Calendar 2005	Commercial	e-baybies, binary conception	5	Health promotion and prevention of disease	XXI - screening, rehab	Unknown
BT Calendar 2005	Commercial	hand-held scanner to detect tumours using tissue resonance interferometer	5	Diagnosis and monitoring of disease	II - cancers	Diagnostic tests and imaging
BT Calendar 2005	Commercial	smart pill bottles remotely monitor medication taking and use alarms	5	Supporting patients receiving care (designed to be used by individuals)	Other - generic	IT systems, eHealth and mHealth

For peer review only - http://bmjopen.bmj.com/site/about/guidelines.xhtml

Appendix1: List of identified healthcare technologies

BT Calendar 2005	Commercial	diabetes cure via stem cell research	5	Treatment and management of symptoms and disease	IV - endocrine	Regenerative medicine
BT Calendar 2005	Commercial	Use of human's own tissues to grow replacement organs	8	Treatment and management of symptoms and disease	Other - generic	Regenerative medicine
BT Calendar 2005	Commercial	Artificial senses	8	Rehabilitation	XXI - screening, rehab	Devices and biomaterials
BT Calendar 2005	Commercial	context sensitive cyber drugs	8	Treatment and management of symptoms and disease	Other - generic	Unknown
BT Calendar 2005	Commercial	electronic stimulation of brain sensations as recreational substitute for drugs	8	Other	VI - nervous	Devices and biomaterials
BT Calendar 2005	Commercial	Custom (GM) foods for particular medical conditions will exist.	8	Treatment and management of symptoms and disease	Other - generic	Other
BT Calendar 2005	Commercial	Gene-gel stimulation of re-growth of natural teeth on demand	8	Treatment and management of symptoms and disease	XI - GI tract, dental health	drugs (not prophylactic vaccines)
BT Calendar 2005	Commercial	retina regeneration using foetal retinal cell injection	8	Treatment and management of symptoms and disease	VII - eye	Regenerative medicine
BT Calendar 2005	Commercial	tooth regeneration	8	Treatment and management of symptoms and disease	XI - GI tract, dental health	Regenerative medicine
BT Calendar 2005	Commercial	plastic bones	8	Treatment and management of symptoms and disease	XIII - musculoskeletal	Devices and biomaterials
BT Calendar 2005	Commercial	emotionally specific drugs	8	Unknown	V - mental health	drugs (not prophylactic vaccines)
BT Calendar 2005	Commercial	synthetic organs by printing layers of cells	10	Treatment and management of symptoms and disease	Other - generic	Regenerative medicine
BT Calendar 2005	Commercial	synthetic viruses	10	Unknown	I -	Other

For peer review only

Appendix1: List of identified healthcare technologies

					infections	
BT Calendar 2005	Commercial	sensory augmentation using sensory implants, nanoparticles etc	10	Rehabilitation	VI - nervous	Devices and biomaterials
BT Calendar 2005	Commercial	use of stem cells in brain after strokes or accidents	10	Treatment and management of symptoms and disease	VI - nervous	Regenerative medicine
BT Calendar 2005	Commercial	gene therapy generates new hair cells in humans	10	Treatment and management of symptoms and disease	XII - skin	drugs (not prophylactic vaccines)
BT Calendar 2005	Commercial	artificial sensors used in cosmetic upgrade surgery	13	Treatment and management of symptoms and disease	Other - generic	Devices and biomaterials
BT Calendar 2005	Commercial	Electronic memory enhancement	20	Unknown	Other - generic	Unknown
BT Calendar 2005	Commercial	Nanobots in toothpaste attack plaque	20	Health promotion and prevention of disease	XI - GI tract, dental health	drugs (not prophylactic vaccines)
BT Calendar 2005	Commercial	Fully functioning artificial eyes	20	Treatment and management of symptoms and disease	VII - eye	Regenerative medicine
BT Calendar 2005	Commercial	electronic brain implants	20	Treatment and management of symptoms and disease	VI - nervous	Devices and biomaterials
BT Calendar 2005	Commercial	Intelligence or memory enhancement by external means	20	Unknown	Other - generic	Unknown
BT Calendar 2005	Commercial	e-baybies, genetic conception	20	Health promotion and prevention of disease	XXI - screening, rehab	Unknown
BT Calendar 2005	Commercial	synthetic immune system	20	Treatment and management of symptoms and disease	III - blood	Regenerative medicine
BT Calendar 2005	Commercial	Artificial peripheral nerves	30	Treatment and management of symptoms and disease	VI - nervous	Regenerative medicine
BT Calendar 2005	Commercial	e-baybies, digital emulation conception	30	Health promotion and prevention of disease	XXI - screening, rehab	Unknown

For peer review only - http://bmjopen.bmj.com/site/about/guidelines.xhtml

Appendix1: List of identified healthcare technologies

BT Calendar 2005	Commercial	Electronic prescriptions reduce fraud and improve speed	3	Supporting the provision of care and/or increasing service efficiency	Other - generic	IT systems, eHealth and mHealth
BT Calendar 2005	Commercial	24/7 blood chemistry monitoring	3	Diagnosis and monitoring of disease	Other - generic	Diagnostic tests and imaging
BT Calendar 2005	Commercial	supermarkets used as major source of medical alerts	3	Health promotion and prevention of disease	Other - generic	Other
BT Calendar 2005	Commercial	Expert systems used extensively in GP surgeries	5	Supporting patients receiving care (designed to be used by individuals)	Other - generic	IT systems, eHealth and mHealth
BT Calendar 2005	Commercial	Hospitals use virtual queuing systems	5	Supporting the provision of care and/or increasing service efficiency	Other - generic	IT systems, eHealth and mHealth
BT Calendar 2005	Commercial	Neural networks used for patient appointment management	5	Supporting the provision of care and/or increasing service efficiency	Other - generic	IT systems, eHealth and mHealth
BT Calendar 2005	Commercial	Multimedia patient records	5	Supporting patients receiving care (designed to be used by individuals)	Other - generic	IT systems, eHealth and mHealth
BT Calendar 2005	Commercial	Lifestyle monitoring and insurance linked to medical records	5	Health promotion and prevention of disease	Other - generic	Population programmes
BT Calendar 2005	Commercial	antibacterial coatings on domestic appliances, phones etc, especially in hospitals	5	Health promotion and prevention of disease	I - infections	Devices and biomaterials
BT Calendar 2005	Commercial	flexible displays used for body monitoring and alerts	5	Diagnosis and monitoring of disease	Other - generic	IT systems, eHealth and mHealth
BT Calendar 2005	Commercial	emotion alerts	5	Health promotion and prevention of disease	Other - generic	Devices and biomaterials
BT Calendar 2005	Commercial	(drug shortages caused by) internet diagnosis	5	Diagnosis and monitoring of disease	Other - generic	IT systems, eHealth and mHealth

For peer review only - http://bmjopen.bmj.com/site/about/guidelines.xhtml

Appendix1: List of identified healthcare technologies

BT Calendar 2005	Commercial	MRSA detection chips on sale in high street	5	Diagnosis and monitoring of disease	I - infections	Population programmes
BT Calendar 2005	Commercial	Devices roaming within blood vessels under own power	8	Unknown	Other - generic	Devices and biomaterials
BT Calendar 2005	Commercial	Operations videoed and stored as part of medical record	8	Supporting patients receiving care (designed to be used by individuals)	Other - generic	IT systems, eHealth and mHealth
BT Calendar 2005	Commercial	Direct electronic pleasure production	8	Other	V - mental health	IT systems, eHealth and mHealth
BT Calendar 2005	Commercial	Orgasm by email	8	Supporting patients receiving care (designed to be used by individuals)	XIV - genitourinary	IT systems, eHealth and mHealth
BT Calendar 2005	Commercial	Artificial senses, sensors directly stimulating nerves	8	Rehabilitation	XXI - screening, rehab	Devices and biomaterials
BT Calendar 2005	Commercial	Smart membranes for remote control of drug injection	8	Treatment and management of symptoms and disease	Other - generic	Devices and biomaterials
BT Calendar 2005	Commercial	Domestic bacterial detection devices	8	Health promotion and prevention of disease	I - infections	Diagnostic tests and imaging
BT Calendar 2005	Commercial	Micro-fluidic chips used for gene sequencing in every GP surgery	8	Diagnosis and monitoring of disease	Other - generic	Diagnostic tests and imaging
BT Calendar 2005	Commercial	Self certification for prescriptions using electronic diagnostics	8	Supporting patients receiving care (designed to be used by individuals)	Other - generic	IT systems, eHealth and mHealth
BT Calendar 2005	Commercial	Visitor sanitising in hospitals	8	Health promotion and prevention of disease	I - infections	Organisational programmes
BT Calendar 2005	Commercial	Outpatients at home - remote tele-medical consultations	8	Supporting patients receiving care (designed to be used by individuals)	Other - generic	IT systems, eHealth and mHealth
BT Calendar 2005	Commercial	Individual's genome part of their medical record	8	Supporting patients receiving care (designed to be used by	Other - generic	Organisational programmes

For peer review only - http://bmjopen.bmj.com/site/about/guidelines.xhtml

Appendix1: List of identified healthcare technologies

				individuals)		
BT Calendar 2005	Commercial	Portable bactericidal devices	10	Health promotion and prevention of disease	I - infections	Medical equipment
BT Calendar 2005	Commercial	Bactericidal saws incorporated onto materials in hospitals	10	Health promotion and prevention of disease	I - infections	Devices and biomaterials
BT Calendar 2005	Commercial	Robotic cleaners in hospitals	10	Supporting the provision of care and/or increasing service efficiency	Other - generic	Medical equipment
Daar et al 2002	Research	Modified molecular technologies for affordable, simple diagnosis of infectious diseases	8	Diagnosis and monitoring of disease	I - infections	Diagnostic tests and imaging
Daar et al 2002	Research	Recombinant technologies to develop vaccines against infectious diseases	8	Health promotion and prevention of disease	I - infections	Prophylactic vaccines
Daar et al 2002	Research	Technologies for more efficient drug and vaccine delivery systems	8	Treatment and management of symptoms and disease	Other - generic	Unknown
Daar et al 2002	Research	Technologies for environmental improvement (sanitation, clean water, bioremediation)	8	Health promotion and prevention of disease	Other - generic	Unknown
Daar et al 2002	Research	Female-controlled protection against sexually transmitted diseases, both with and without contraceptive effect	8	Health promotion and prevention of disease	XIV - genitourinary	Devices and biomaterials
Daar et al 2002	Research	Genetically modified crops with increased nutrients to counter specific deficiencies	8	Health promotion and prevention of disease	IV - endocrine	Unknown

For peer review only - http://bmjopen.bmj.com/site/about/guidelines.xhtml

Appendix1: List of identified healthcare technologies

Daar et al 2002	Research	Recombinant technology to make therapeutic products (for example, insulin, interferons) more affordable	8	Treatment and management of symptoms and disease	Other - generic	drugs (not prophylactic vaccines)
Karim2000	Policy planning	Telemedicine	15	Supporting patients receiving care (designed to be used by individuals)	Other - generic	IT systems, eHealth and mHealth
Karim2000	Policy planning	Medical databases	15	Supporting the provision of care and/or increasing service efficiency	Other - generic	IT systems, eHealth and mHealth
Karim2000	Policy planning	Expert System in managed care and preventive medicine	15	Supporting patients receiving care (designed to be used by individuals)	Other - generic	IT systems, eHealth and mHealth
Karim2000	Policy planning	diagnostic procedures (e.g. recombinant DNA techniques for parental screening)	15	Diagnosis and monitoring of disease	Other - generic	Diagnostic tests and imaging
Karim2000	Policy planning	Therapeutic procedures (e.g. microsurgery and implanted muscle stimulators).	15	Treatment and management of symptoms and disease	Other - generic	Therapeutic procedures
Karim2000	Policy planning	Nanotechnology (e.g. devices which interact with tissues within the body to boost the immune system).	15	Treatment and management of symptoms and disease	III - blood	Devices and biomaterials
Karim2000	Policy planning	Vaccines against infectious diseases	15	Health promotion and prevention of disease	I - infections	Prophylactic vaccines
Karim2000	Policy planning	Development of an AIDS vaccine	15	Health promotion and prevention of disease	I - infections	Prophylactic vaccines
Karim2000	Policy planning	Development of a TB vaccine	15	Health promotion and prevention of disease	I - infections	Prophylactic vaccines
Karim2000	Policy planning	Health promotion targeted at the youth	15	Health promotion and prevention of disease	Other - generic	Population programmes

For peer review only - http://bmjopen.bmj.com/site/about/guidelines.xhtml

Appendix1: List of identified healthcare technologies

Karim2000	Policy planning	Development of enriched and fortified staple foods	15	Health promotion and prevention of disease	Other - generic	Population programmes
Karim2000	Policy planning	Development of a malaria vaccine	15	Health promotion and prevention of disease	I - infections	Prophylactic vaccines
Karim2000	Policy planning	Development of on-site diagnostic tools	15	Diagnosis and monitoring of disease	Other - generic	Diagnostic tests and imaging
Karim2000	Policy planning	Development of female contraceptives	15	Health promotion and prevention of disease	XXI - screening, rehab	drugs (not prophylactic vaccines)
Karim2000	Policy planning	Development of barrier methods and microbicides for sexually transmitted diseases and HIV	15	Health promotion and prevention of disease	I - infections	Devices and biomaterials
Karim2000	Policy planning	Development of malaria drugs	15	Treatment and management of symptoms and disease	I - infections	drugs (not prophylactic vaccines)
Karim2000	Policy planning	Development of new TB drugs	15	Treatment and management of symptoms and disease	I - infections	drugs (not prophylactic vaccines)
Karim2000	Policy planning	Self management tools for chronic diseases (e.g. hypertension, diabetes, diarrhoea, psychiatric conditions)	15	Supporting patients receiving care (designed to be used by individuals)	Other - generic	Organisational programmes
Karim2000	Policy planning	Tissue regeneration and gene therapy	15	Treatment and management of symptoms and disease	Other - generic	Regenerative medicine
Sbipy 1988	Research	Monoclonal antibodies will be used in: Histopathological techniques	0	Diagnosis and monitoring of disease	Other - generic	Diagnostic tests and imaging
Sbipy 1988	Research	Monoclonal antibodies will be used in: Biochemical techniques	0	Diagnosis and monitoring of disease	Other - generic	Diagnostic tests and imaging
Sbipy 1988	Research	Monoclonal antibodies will be	0	Diagnosis and monitoring of	Other - generic	Diagnostic tests and imaging

For peer review only - http://bmjopen.bmj.com/site/about/guidelines.xhtml

Appendix1: List of identified healthcare technologies

		used in: in-vivo diagnostic techniques		disease		
Sbipy 1988	Research	Monoclonal antibodies will be used in: Identification and localization of tumours	0	Diagnosis and monitoring of disease	II - cancers	Diagnostic tests and imaging
Sbipy 1988	Research	Gene probes will be used in: diagnostic microbiology	0	Diagnosis and monitoring of disease	I - infections	Diagnostic tests and imaging
Sbipy 1988	Research	Gene probes will be used in: diagnostic virology	0	Diagnosis and monitoring of disease	I - infections	Diagnostic tests and imaging
Sbipy 1988	Research	Gene probes will be used in: To screen for potentially deleterious genes*	0	Diagnosis and monitoring of disease	Other - generic	Diagnostic tests and imaging
Sbipy 1988	Research	Gene probes will be used in: Diagnosing most genetic disorders	10	Diagnosis and monitoring of disease	XVII - congenital	Diagnostic tests and imaging
Sbipy 1988	Research	Genetic engineering will enable the isolation of high risk groups for common diseases	10	Health promotion and prevention of disease	Other - generic	Population programmes
Sbipy 1988	Research	Imaging techniques will be in widespread and less hazardous use including: Ultrasound	0	Diagnosis and monitoring of disease	Other - generic	Diagnostic tests and imaging
Sbipy 1988	Research	Imaging techniques will be in widespread and less hazardous use including: Doppler measurement	0	Diagnosis and monitoring of disease	Other - generic	Diagnostic tests and imaging
Sbipy 1988	Research	Imaging techniques will be in widespread and less hazardous use including: CT scanning	0	Diagnosis and monitoring of disease	Other - generic	Diagnostic tests and imaging

For peer review only

Appendix1: List of identified healthcare technologies

Sbipy 1988	Research	Imaging techniques will be in widespread and less hazardous use including: NMR scanning	0	Diagnosis and monitoring of disease	Other - generic	Diagnostic tests and imaging
Sbipy 1988	Research	Imaging techniques will be in widespread and less hazardous use including: Nuclear medicine	0	Diagnosis and monitoring of disease	Other - generic	Diagnostic tests and imaging
Sbipy 1988	Research	Imaging techniques will be in widespread and less hazardous use including: Positron detection*	0	Diagnosis and monitoring of disease	Other - generic	Diagnostic tests and imaging
Sbipy 1988	Research	Imaging techniques will be in widespread and less hazardous use including: Three-dimensional dynamic imaging	10	Diagnosis and monitoring of disease	Other - generic	Diagnostic tests and imaging
Sbipy 1988	Research	Drug therapy will be enhanced by: Genetic engineering	10	Treatment and management of symptoms and disease	Other - generic	drugs (not prophylactic vaccines)
Sbipy 1988	Research	Drug therapy will be enhanced by: Monoclonal antibodies used for drug targeting	10	Treatment and management of symptoms and disease	Other - generic	drugs (not prophylactic vaccines)
Sbipy 1988	Research	Drug delivery devices with or without in-vivo sensors	10	Treatment and management of symptoms and disease	Other - generic	Devices and biomaterials
Sbipy 1988	Research	More effective Therapeutics will be available for: Viral infections	0	Treatment and management of symptoms and disease	I - infections	drugs (not prophylactic vaccines)
Sbipy 1988	Research	More effective Therapeutics will be available for: Autoimmune diseases (monoclonal antibodies)	10	Treatment and management of symptoms and disease	III - blood	drugs (not prophylactic vaccines)
Sbipy 1988	Research	More effective Therapeutics will	0	Treatment and management of	IX - circulation	drugs (not prophylactic

For peer review only - http://bmjopen.bmj.com/site/about/guidelines.xhtml

Appendix1: List of identified healthcare technologies

		be available for: Heart failure (better drugs)		symptoms and disease		vaccines)
Sbipy 1988	Research	More effective Therapeutics will be available for: Obstructive arterial lesions (by removal and control of platelets and synthetic vascular prostheses)	10	Treatment and management of symptoms and disease	IX - circulation	drugs (not prophylactic vaccines)
Sbipy 1988	Research	Cancer (hyperthermia, growth regulation factors and monoclonal antibodies)	10	Other	II - cancers	Other
Sbipy 1988	Research	Cardiac arrhythmias (implantable mechanisms)	10	Treatment and management of symptoms and disease	IX - circulation	Devices and biomaterials
Sbipy 1988	Research	More effective Therapeutics will be available for: Arthritic joints (more biocompatible prosthetic materials and a wider range of joint replacements)	0	Treatment and management of symptoms and disease	XIII - musculoskeletal	Devices and biomaterials
Sbipy 1988	Research	More effective Therapeutics will be available for: Incontinence (stimulation via implantable electrodes)	0	treatment and management of symptoms and disease	XIV - genitourinary	Devices and biomaterials
Sbipy 1988	Research	More effective Therapeutics will be available for: Disability (wider range of flow technology aids and biological servomechanisms linked to computers and	0	Rehabilitation	Other - generic	Other

For peer review only - http://bmjopen.bmj.com/site/about/guidelines.xhtml

Appendix1: List of identified healthcare technologies

		robotics)				
Sbipy 1988	Research	More effective Therapeutics will be available for: Limb damage (permanent internal bone repair)	0	Treatment and management of symptoms and disease	XIII - musculoskeletal	Devices and biomaterials
Sbipy 1988	Research	More effective Therapeutics will be available for: Tropical parasitic diseases (vaccines)	0	Health promotion and prevention of disease	I - infections	Prophylactic vaccines
Sbipy 1988	Research	More effective Therapeutics will be available for: Mental disorders (neurotransmitters)	10	Treatment and management of symptoms and disease	V - mental health	drugs (not prophylactic vaccines)
Sbipy 1988	Research	Laser microsurgery	0	Treatment and management of symptoms and disease	Other - generic	Therapeutic procedures
Sbipy 1988	Research	Laser endoscopic surgery	0	Treatment and management of symptoms and disease	Other - generic	Therapeutic procedures
Sbipy 1988	Research	Laser angioplasty and angiography	0	Treatment and management of symptoms and disease	IX - circulation	Therapeutic procedures
Sbipy 1988	Research	Other laser and endoscopic surgical techniques	10	Treatment and management of symptoms and disease	Other - generic	Therapeutic procedures
Sbipy 1988	Research	Cryosurgery	10	Treatment and management of symptoms and disease	Other - generic	Therapeutic procedures
Sbipy 1988	Research	Lithotripsy	10	Treatment and management of symptoms and disease	Other - generic	Therapeutic procedures
Sbipy 1988	Research	Transplantation will be enhanced by better techniques	0	Treatment and management of symptoms and disease	Other - generic	Devices and biomaterials

For peer review only - http://bmjopen.bmj.com/site/about/guidelines.xhtml

Appendix1: List of identified healthcare technologies

		enabling long term in-vitro organ preservation and the prevention of rejection				
Sbipy 1988	Research	Bone marrow transplantation with purified stem cells and autologous material will be available	0	Treatment and management of symptoms and disease	III - blood	Regenerative medicine
Sbipy 1988	Research	A safe male pill for long-term use will be available	10	Health promotion and prevention of disease	XXI - screening, rehab	drugs (not prophylactic vaccines)
Sbipy 1988	Research	Improved techniques for detecting ovulation will enhance natural family planning*	0	Diagnosis and monitoring of disease	XIV - genitourinary	Diagnostic tests and imaging
Sbipy 1988	Research	Optical disc storage and communication will be used with X-rays and other diagnostic images	0	Supporting the provision of care and/or increasing service efficiency	Other - generic	Diagnostic tests and imaging
Sbipy 1988	Research	Computers will be used by clinical departments for data collection	10	Supporting the provision of care and/or increasing service efficiency	Other - generic	IT systems, eHealth and mHealth
Sbipy 1988	Research	Expert interrogation will be possible due to data centralization	0	Supporting the provision of care and/or increasing service efficiency	Other - generic	IT systems, eHealth and mHealth
Sbipy 1988	Research	Patient interviewing systems will be available in the surgery and in patients' homes	10	Supporting patients receiving care (designed to be used by individuals)	Other - generic	IT systems, eHealth and mHealth
Sbipy 1988	Research	Expert systems will enable diagnosis and define the most applicable treatment	10	Supporting patients receiving care (designed to be used by individuals)	Other - generic	IT systems, eHealth and mHealth

For peer review only - http://bmjopen.bmj.com/site/about/guidelines.xhtml

Appendix1: List of identified healthcare technologies

Sbipy 1988	Research	Linkage of computerized patient data will be available between all hospital, community and primary care services and settings	10	Supporting the provision of care and/or increasing service efficiency	Other - generic	IT systems, eHealth and mHealth
STG 1988	Policy planning	Life style changing behaviours	N/A	Health promotion and prevention of disease	Other - generic	Population programmes
STG 1988	Policy planning	Screening for phenylketonuria	N/A	Health promotion and prevention of disease	IV - endocrine	Population programmes
STG 1988	Policy planning	Screening for congenital hypothyroidism	N/A	Health promotion and prevention of disease	IV - endocrine	Population programmes
STG 1988	Policy planning	Blood pressure can be checked by self-service meters	N/A	Diagnosis and monitoring of disease	IX - circulation	Diagnostic tests and imaging
STG 1988	Policy planning	High serum cholesterol can be checked by fingerprick blood test	N/A	Diagnosis and monitoring of disease	IX - circulation	Diagnostic tests and imaging
STG 1988	Policy planning	Certain cancer tests can be self-administered	N/A	Diagnosis and monitoring of disease	II - cancers	Diagnostic tests and imaging
STG 1988	Policy planning	Improved diagnostic tests may be available for widespread use for screening at the community level	N/A	Health promotion and prevention of disease	Other - generic	Population programmes
STG 1988	Policy planning	Improved vaccine against cholera in the tropical developing countries	N/A	Health promotion and prevention of disease	I - infections	Prophylactic vaccines
STG 1988	Policy planning	New malaria vaccine in the tropical developing countries	N/A	Health promotion and prevention of disease	I - infections	Prophylactic vaccines
STG 1988	Policy planning	New vaccine against hepatitus B	N/A	Health promotion and prevention of disease	I - infections	Prophylactic vaccines

For peer review only - http://bmjopen.bmj.com/site/about/guidelines.xhtml

Appendix1: List of identified healthcare technologies

STG 1988	Policy planning	New vaccine against respiratory syncytial virus	N/A	Health promotion and prevention of disease	I - infections	Prophylactic vaccines
STG 1988	Policy planning	New vaccine against influenza	N/A	Health promotion and prevention of disease	I - infections	Prophylactic vaccines
STG 1988	Policy planning	New vaccine against AIDS	N/A	Health promotion and prevention of disease	I - infections	Prophylactic vaccines
STG 1988	Policy planning	New vaccine against cytomegalovirus	N/A	Health promotion and prevention of disease	I - infections	Prophylactic vaccines
STG 1988	Policy planning	Better vaccine against Perussis	N/A	Health promotion and prevention of disease	I - infections	Prophylactic vaccines
STG 1988	Policy planning	Oral administration or administartion by nasal spray will facilitate mass immunization	N/A	Health promotion and prevention of disease	I - infections	Prophylactic vaccines
STG 1988	Policy planning	Creating vaccines containing antigens for several organisms given together (e.g. DPTPM)	N/A	Health promotion and prevention of disease	I - infections	Prophylactic vaccines
STG 1988	Policy planning	Shifts in diet toward lower intake of fat especially saturated fat	N/A	Health promotion and prevention of disease	Other - generic	Population programmes
STG 1988	Policy planning	Reliable and simple test for cholesterol	N/A	Diagnosis and monitoring of disease	IX - circulation	Diagnostic tests and imaging
STG 1988	Policy planning	More effective and safer drugs for treatment of elevated cholesterol	N/A	Health promotion and prevention of disease	IX - circulation	drugs (not prophylactic vaccines)
STG 1988	Policy planning	Technologies for home use without professional supervision (e.g. home diagnostic kits)	N/A	Supporting patients receiving care (designed to be used by individuals)	Other - generic	Diagnostic tests and imaging
STG 1988	Policy planning	Disease conditions will be monitored from distant professional sites	N/A	Supporting patients receiving care (designed to be used by	Other - generic	IT systems, eHealth and mHealth

For peer review only

Appendix1: List of identified healthcare technologies

				individuals)		
STG 1988	Policy planning	Robotics will be used for mobility and assistance at home such as nutrition and cleaning	N/A	Rehabilitation	Other - generic	Assistive devices
STG 1988	Policy planning	Regeneration of central nervous system through the use of nerve growth factors or transplant neurotransmitter-producing cells into the brain	N/A	Treatment and management of symptoms and disease	VI - nervous	Regenerative medicine
STG 1988	Policy planning	Grafting of the spinal cord or the peripheral nervous system	N/A	Treatment and management of symptoms and disease	VI - nervous	Therapeutic procedures
STG 1988	Policy planning	More safer & effective contraception	N/A	Health promotion and prevention of disease	XXI - screening, rehab	Drugs (not prophylactic vaccines)
STG 1988	Policy planning	Contraceptive vaccine	N/A	Health promotion and prevention of disease	XXI - screening, rehab	Prophylactic vaccines
STG 1988	Policy planning	Alternatives to surgical abortion such as combination of antiprogestins and prostaglandins	N/A	Treatment and management of symptoms and disease	XV - pregnancy	drugs (not prophylactic vaccines)
STG 1988	Policy planning	Treatment of infertility using new technologies such as in vitro fertilization, surrogate motherhood, freezing of germ cells and embro with embryo transfer	N/A	Treatment and management of symptoms and disease	XIV - genitourinary	Other
STG 1988	Policy planning	Fetal cells can be isolated from the mother's bloodstream as a method for prenatal screening	N/A	Health promotion and prevention of disease	XXI - screening, rehab	Diagnostic tests and imaging

For peer review only - http://bmjopen.bmj.com/site/about/guidelines.xhtml

Appendix1: List of identified healthcare technologies

STG 1988	Policy planning	Metabolic, physiologic information can be obtained by spectroscopy	N/A	Diagnosis and monitoring of disease	Other - generic	Diagnostic tests and imaging
STG 1988	Policy planning	Data from CT scanner will be stored on a disk, transmit by a phone line or othe communication	N/A	Supporting patients receiving care (designed to be used by individuals)	Other - generic	Diagnostic tests and imaging
STG 1988	Policy planning	Film may disappear and computer will directly interpret the diagnosis	N/A	Supporting patients receiving care (designed to be used by individuals)	Other - generic	Diagnostic tests and imaging
STG 1988	Policy planning	Biosensors to monitor disease condition or results of therapy (e.g. diabetes)	N/A	Diagnosis and monitoring of disease	Other - generic	Diagnostic tests and imaging
STG 1988	Policy planning	Tumor markers that can be measured in blood	N/A	Diagnosis and monitoring of disease	II - cancers	Diagnostic tests and imaging
STG 1988	Policy planning	Flow cytometer will be used in transplantation or monitoring of cancer therapy	N/A	Diagnosis and monitoring of disease	II - cancers	Diagnostic tests and imaging
STG 1988	Policy planning	Monoclonal antibodies for diagnosis such as colon cancer and area of cardiac infarctions	N/A	Diagnosis and monitoring of disease	Other - generic	Diagnostic tests and imaging
STG 1988	Policy planning	Human gene therapy for a wide variety of diseases including cancer, diabetes, cardiovascular and affective disorders such as depression	N/A	Treatment and management of symptoms and disease	Other - generic	Regenerative medicine
STG 1988	Policy planning	DNA probe for intra-uterine diagnosis of genetic diseases	N/A	Diagnosis and monitoring of disease	XVII - congenital	Diagnostic tests and imaging
STG 1988	Policy planning	Home diagnostic kits for sexually	7	Diagnosis and monitoring of	I - infections	Organisational programmes

For peer review only

Appendix1: List of identified healthcare technologies

		transmitted diseases and hepatitits		disease		
STG 1988	Policy planning	Home diagnostic kits for screening for cancer	7	Diagnosis and monitoring of disease	I - infections	Organisational programmes
STG 1988	Policy planning	Home diagnostic kits to test for common diseases with genetic basis such as diabetes	N/A	Diagnosis and monitoring of disease	Other - generic	Organisational programmes
STG 1988	Policy planning	Genetic insertion therapy for treatment of certain blood disorders such as thalassaemia and sickle cell anaemia	N/A	Treatment and management of symptoms and disease	III - blood	Regenerative medicine
STG 1988	Policy planning	Oral treatment forms for hormones, enzymes and other peptides may be developed	N/A	Treatment and management of symptoms and disease	Other - generic	Drugs (not prophylactic vaccines)
STG 1988	Policy planning	New drugs for asthma	N/A	Treatment and management of symptoms and disease	X - respiratory	Drugs (not prophylactic vaccines)
STG 1988	Policy planning	New drugs for coronary artery diseases	N/A	Treatment and management of symptoms and disease	IX - circulation	Drugs (not prophylactic vaccines)
STG 1988	Policy planning	Monoclonal antibodies to replace immune globulins	N/A	Treatment and management of symptoms and disease	III - blood	Drugs (not prophylactic vaccines)
STG 1988	Policy planning	Cloning of skin	N/A	Treatment and management of symptoms and disease	XII - skin	Regenerative medicine
STG 1988	Policy planning	Growth of retinal tissue and corneal endothelium	N/A	Treatment and management of symptoms and disease	VII - eye	Regenerative medicine
STG 1988	Policy planning	Total body irradiation for transplant (to prevent rejection)	N/A	Treatment and management of symptoms and disease	III - blood	Non-surgical therapy
STG 1988	Policy planning	Artificial heart	N/A	Treatment and management of symptoms and	IX - circulation	Regenerative medicine

For peer review only

Appendix1: List of identified healthcare technologies

				disease		
STG 1988	Policy planning	Artificial pancreas	N/A	Treatment and management of symptoms and disease	IV - endocrine	Regenerative medicine
STG 1988	Policy planning	Shoulder joint replacement	N/A	Treatment and management of symptoms and disease	XIII - musculoskeletal	Devices and biomaterials
STG 1988	Policy planning	Cochlear implants	N/A	Treatment and management of symptoms and disease	VIII - ear	Devices and biomaterials
STG 1988	Policy planning	Coating of implant materials to improve acceptability	N/A	Other	Other - generic	Devices and biomaterials
STG 1988	Policy planning	Functional electrical stimulation in prosthetics allowing brain to control the action	N/A	Rehabilitation	XXI - screening, rehab	Devices and biomaterials
STG 1988	Policy planning	Prostheic fingers	N/A	Rehabilitation	XIII - musculoskeletal	Devices and biomaterials
STG 1988	Policy planning	Organ transplant and improvement of functional activities of organs (pancreas, bone, small bowel, endocine glands..)	N/A	Treatment and management of symptoms and disease	Other - generic	Other
STG 1988	Policy planning	Microprocessor to restore damaged areas of the brain	N/A	Rehabilitation	VI - nervous	Devices and biomaterials
STG 1988	Policy planning	Laser cytofluorometry will be used to identify RNA and DNA in the plasma	N/A	Diagnosis and monitoring of disease	Other - generic	Diagnostic tests and imaging
STG 1988	Policy planning	Laser doppler to measure blood flow in the retina	N/A	Diagnosis and monitoring of disease	Other - generic	Diagnostic tests and imaging
STG 1988	Policy planning	Laser to stop gastrointestinal bleeding	N/A	Treatment and management of symptoms and disease	XI - GI tract, dental health	Therapeutic procedures

For peer review only - http://bmjopen.bmj.com/site/about/guidelines.xhtml

Appendix1: List of identified healthcare technologies

STG 1988	Policy planning	Laser to break urinary stones	N/A	Treatment and management of symptoms and disease	XIV - genitourinary	Therapeutic procedures
STG 1988	Policy planning	Laser surgery through endoscope	N/A	Treatment and management of symptoms and disease	Other - generic	Therapeutic procedures
STG 1988	Policy planning	Widespread use of microsurgery	N/A	Treatment and management of symptoms and disease	Other - generic	Therapeutic procedures
STG 1988	Policy planning	Electronic gas valve regulator for safety in anaesthaesia	N/A	Supporting the provision of care and/or increasing service efficiency	XIX - injury	Medical equipment
STG 1988	Policy planning	Instantaneous measurement of blood gases and drug levels in the body during anasthaesia	N/A	Supporting patients receiving care (designed to be used by individuals)	XIX - injury	Diagnostic tests and imaging
STG 1988	Policy planning	Computerised ECG operating room	N/A	Supporting the provision of care and/or increasing service efficiency	Other - generic	IT systems, eHealth and mHealth
STG 1988	Policy planning	Data on each patient available at any moment during the operation	N/A	Supporting patients receiving care (designed to be used by individuals)	Other - generic	IT systems, eHealth and mHealth
STG 1988	Policy planning	Computerised electroanaesthesia	N/A	Supporting patients receiving care (designed to be used by individuals)	Other - generic	IT systems, eHealth and mHealth
STG 1988	Policy planning	New methods to close wounds e.g. tissue glue with ultrasound	N/A	Treatment and management of symptoms and disease	Other - generic	Devices and biomaterials
STG 1988	Policy planning	Spectroscopy using optical methods to diagnose caries	N/A	Diagnosis and monitoring of disease	XI - GI tract, dental health	Diagnostic tests and imaging
STG 1988	Policy planning	Bone like implants for teeth and bones	N/A	Treatment and management of symptoms and disease	XI - GI tract, dental health	Devices and biomaterials
STG 1988	Policy planning	Home monitoring especially of the elderly	N/A	Supporting patients receiving care (designed to	Other - generic	IT systems, eHealth and mHealth

For peer review only - http://bmjopen.bmj.com/site/about/guidelines.xhtml

Appendix1: List of identified healthcare technologies

				be used by individuals)		
STG 1988	Policy planning	Networks for communications between and within hospitals	N/A	Supporting the provision of care and/or increasing service efficiency	Other - generic	IT systems, eHealth and mHealth
STG 1988	Policy planning	Huge mass memories (e.g. on laser disc) for the storage of medical records	N/A	Supporting the provision of care and/or increasing service efficiency	Other - generic	IT systems, eHealth and mHealth
STG 1988	Policy planning	Video disk oriented data base for medical records	N/A	Supporting the provision of care and/or increasing service efficiency	Other - generic	IT systems, eHealth and mHealth
STG 1988	Policy planning	Computer aided examination and testing	N/A	Supporting patients receiving care (designed to be used by individuals)	Other - generic	IT systems, eHealth and mHealth
STG 1988	Policy planning	Improved biomaterials for joint implants and plastic surgery	N/A	Treatment and management of symptoms and disease	XIII - musculoskeletal	Devices and biomaterials
STG 1988	Policy planning	Improved materials for artificial blood vessels	N/A	Treatment and management of symptoms and disease	IX - circulation	Devices and biomaterials
Cahill et al Technology Map 1999	policy planning	Therapeutics for arterial occlusion and myocardial infarction	4	Treatment and management of symptoms and disease	IX - circulation	Drugs (not prophylactic vaccines)
Cahill et al Technology Map 1999	Policy planning	Guidelines for adult disease preventing life style	8	Supporting the provision of care and/or increasing service efficiency	XXI - screening, rehab	Organisational programmes
Cahill et al Technology Map 1999	policy planning	Development of anti-cancer agents (gene level)	11	Treatment and management of symptoms and disease	II - cancers	Drugs (not prophylactic vaccines)
Cahill et al Technology Map 1999	policy planning	drugs preventing the occurrence of cancer	12	Health promotion and prevention of disease	II - cancers	Drugs (not prophylactic vaccines)
Cahill et al Technology Map 1999	policy planning	Effective means to prevent metastasis	15	Health promotion and prevention of disease	II - cancers	Unknown
Cahill et al Technology Map 1999	policy planning	Genetic/biochemical tests for schizophrenia and depression	9	Diagnosis and monitoring of disease	VI - nervous	Diagnostic tests and imaging

For peer review only

Appendix1: List of identified healthcare technologies

Food and Drug Administration (FDA) surveys 1998	Policy planning	Biosensors	10	Unknown	Other - generic	Diagnostic tests and imaging
Food and Drug Administration (FDA) surveys 1998	Policy planning	Laser diagnosis	10	Diagnosis and monitoring of disease	Other - generic	Diagnostic tests and imaging
Food and Drug Administration (FDA) surveys 1998	Policy planning	Tissue engineered devices	10	Treatment and management of symptoms and disease	Other - generic	Devices and biomaterials
Food and Drug Administration (FDA) surveys 1998	Policy planning	Blood vessel prosthetics	10	Treatment and management of symptoms and disease	IX - circulation	Regenerative medicine
Food and Drug Administration (FDA) surveys 1998	Policy planning	Bone prosthetics/ growth	10	Treatment and management of symptoms and disease	XIII - musculoskeletal	Regenerative medicine
Food and Drug Administration (FDA) surveys 1998	Policy planning	Cardiac stimulation	10	Treatment and management of symptoms and disease	IX - circulation	Unknown
Food and Drug Administration (FDA) surveys 1998	Policy planning	cartilage prosthetics	10	Treatment and management of symptoms and disease	XIII - musculoskeletal	Devices and biomaterials
Food and Drug Administration (FDA) surveys 1998	Policy planning	Computer aided clinical labs	10	Diagnosis and monitoring of disease	Other - generic	IT systems, eHealth and mHealth
Food and Drug Administration (FDA) surveys 1998	Policy planning	Computer aided diagnosis	10	Supporting the provision of care and/or increasing service efficiency	Other - generic	IT systems, eHealth and mHealth
Food and Drug Administration (FDA) surveys 1998	Policy planning	Telemedicine	10	Supporting patients receiving care (designed to be used by individuals)	Other - generic	IT systems, eHealth and mHealth
Food and Drug Administration (FDA) surveys 1998	Policy planning	Drug impregnated devices	10	Treatment and management of symptoms and disease	Other - generic	Devices and biomaterials
Food and Drug Administration (FDA) surveys 1998	Policy planning	Endoscopy	10	Diagnosis and monitoring of disease	Other - generic	Diagnostic tests and imaging
Food and Drug Administration	Policy planning	Hearing aids/Nonimplante	10	Rehabilitation	VIII - ear	Devices and biomaterials

For peer review only

For peer review only - http://bmjopen.bmj.com/site/about/guidelines.xhtml

Appendix1: List of identified healthcare technologies

(FDA) surveys 1998		d sensory aids				
Food and Drug Administration (FDA) surveys 1998	Policy planning	Heart valves	10	Treatment and management of symptoms and disease	IX - circulation	Devices and biomaterials
Food and Drug Administration (FDA) surveys 1998	Policy planning	Heart pumps	10	Treatment and management of symptoms and disease	IX - circulation	Devices and biomaterials
Food and Drug Administration (FDA) surveys 1998	Policy planning	Home diagnostics /Home/self monitoring & diagnosis	10	Supporting patients receiving care (designed to be used by individuals)	Other - generic	Organisational programmes
Food and Drug Administration (FDA) surveys 1998	Policy planning	Image contrast agents	10	Diagnosis and monitoring of disease	Other - generic	Diagnostic tests and imaging
Food and Drug Administration (FDA) surveys 1998	Policy planning	Integrated patient medical info systems	10	Supporting the provision of care and/or increasing service efficiency	Other - generic	IT systems, eHealth and mHealth
Food and Drug Administration (FDA) surveys 1998	Policy planning	Laser treatment	10	Treatment and management of symptoms and disease	Other - generic	Non-surgical therapy
Food and Drug Administration (FDA) surveys 1998	Policy planning	kidney prosthetics	10	Treatment and management of symptoms and disease	XIV - genitourina ry	Regenerative medicine
Food and Drug Administration (FDA) surveys 1998	Policy planning	Laser surgery	10	Treatment and management of symptoms and disease	Other - generic	Non-surgical therapy
Food and Drug Administration (FDA) surveys 1998	Policy planning	Liver prosthetics	10	Treatment and management of symptoms and disease	XI - GI tract, dental health	Regenerative medicine
Food and Drug Administration (FDA) surveys 1998	Policy planning	Mini invasive neurosurgery	10	Treatment and management of symptoms and disease	VI - nervous	Therapeutic procedures
Food and Drug Administration (FDA) surveys 1998	Policy planning	Mini invasive cardiac surgery	10	Treatment and management of symptoms and disease	IX - circulation	Therapeutic procedures
Food and Drug Administration (FDA) surveys 1998	Policy planning	MRI	10	Diagnosis and monitoring of disease	Other - generic	Diagnostic tests and imaging

For peer review only - http://bmjopen.bmj.com/site/about/guidelines.xhtml

Appendix1: List of identified healthcare technologies

Food and Drug Administration (FDA) surveys 1998	Policy planning	Neuromuscular stimulation	10	Treatment and management of symptoms and disease	Other - generic	Non-surgical therapy
Food and Drug Administration (FDA) surveys 1998	Policy planning	Nerve regeneration	10	Treatment and management of symptoms and disease	VI - nervous	Regenerative medicine
Food and Drug Administration (FDA) surveys 1998	Policy planning	Neural stimulation	10	Treatment and management of symptoms and disease	VI - nervous	Non-surgical therapy
Food and Drug Administration (FDA) surveys 1998	Policy planning	Ocular prosthetics	10	Treatment and management of symptoms and disease	VII - eye	Devices and biomaterials
Food and Drug Administration (FDA) surveys 1998	Policy planning	Pancreas prosthetics	10	Treatment and management of symptoms and disease	IV - endocrine	Regenerative medicine
Food and Drug Administration (FDA) surveys 1998	Policy planning	Patient smart cards	10	Supporting patients receiving care (designed to be used by individuals)	Other - generic	IT systems, eHealth and mHealth
Food and Drug Administration (FDA) surveys 1998	Policy planning	Robotic surgery	10	Treatment and management of symptoms and disease	Other - generic	Therapeutic procedures
Food and Drug Administration (FDA) surveys 1998	Policy planning	Skin prosthetics	10	Treatment and management of symptoms and disease	XII - skin	Regenerative medicine
Food and Drug Administration (FDA) surveys 1998	Policy planning	Telemedicine-home use	10	Supporting patients receiving care (designed to be used by individuals)	Other - generic	IT systems, eHealth and mHealth
Food and Drug Administration (FDA) surveys 1998	Policy planning	Telemedicine-radiology	10	Supporting patients receiving care (designed to be used by individuals)	Other - generic	IT systems, eHealth and mHealth
Food and Drug Administration (FDA) surveys 2008	Policy planning	Robotic surgical systems	0	Treatment and management of symptoms and disease	Other - generic	Therapeutic procedures
Food and Drug Administration (FDA) surveys 1998	Policy planning	Point-of-care products	10	Diagnosis and monitoring of disease	Other - generic	Diagnostic tests and imaging

For peer review only - http://bmjopen.bmj.com/site/about/guidelines.xhtml

Appendix1: List of identified healthcare technologies

Food and Drug Administration (FDA) surveys 1998	Policy planning	Computer-assisted diagnostic systems	10	Diagnosis and monitoring of disease	Other - generic	IT systems, eHealth and mHealth
Food and Drug Administration (FDA) surveys 1998	Policy planning	Neuro-sensory devices	10	Diagnosis and monitoring of disease	VI - nervous	Devices and biomaterials
Food and Drug Administration (FDA) surveys 1998	Policy planning	Optical diagnostic products	10	Diagnosis and monitoring of disease	Other - generic	Diagnostic tests and imaging
Food and Drug Administration (FDA) surveys 1998	Policy planning	Integrated electronic patient medical records	10	Supporting the provision of care and/or increasing service efficiency	Other - generic	IT systems, eHealth and mHealth
Food and Drug Administration (FDA) surveys 1998	Policy planning	Remote patient monitoring systems	10	Supporting patients receiving care (designed to be used by individuals)	Other - generic	IT systems, eHealth and mHealth
Food and Drug Administration (FDA) surveys 1998	Policy planning	Glucose monitoring products	10	Diagnosis and monitoring of disease	IV - endocrine	Diagnostic tests and imaging
Food and Drug Administration (FDA) surveys 1998	Policy planning	Tissue engineered products	10	Treatment and management of symptoms and disease	Other - generic	Regenerative medicine
Food and Drug Administration (FDA) surveys 1998	Policy planning	Advanced prosthetic limbs	10	Rehabilitation	XIII - musculoskeletal	Devices and biomaterials
Food and Drug Administration (FDA) surveys 1998	Policy planning	Minimally invasive implants (e.g., percutaneous or natural orifice delivery)	10	Treatment and management of symptoms and disease	Other - generic	Devices and biomaterials
Food and Drug Administration (FDA) surveys 1998	Policy planning	Virtual reality systems for immersive training and other applications	10	Unknown	Other - generic	IT systems, eHealth and mHealth
Food and Drug Administration (FDA) surveys 1998	Policy planning	Optical therapeutic devices	10	Treatment and management of symptoms and disease	Other - generic	Devices and biomaterials
Food and Drug Administration (FDA) surveys 1998	Policy planning	Internet-based medical device systems	10	Supporting patients receiving care (designed to be used by	Other - generic	IT systems, eHealth and mHealth

For peer review only - http://bmjopen.bmj.com/site/about/guidelines.xhtml

Appendix1: List of identified healthcare technologies

				individuals)		
Food and Drug Administration (FDA) surveys 1998	Policy planning	New (non-eluting) drug delivery systems	10	Treatment and management of symptoms and disease	Other - generic	Drugs (not prophylactic vaccines)
Food and Drug Administration (FDA) surveys 1998	Policy planning	Robotic prosthetics	20	Rehabilitation	Other - generic	Devices and biomaterials
Food and Drug Administration (FDA) surveys 1998	Policy planning	New types of insulin pumps and delivery systems	20	Treatment and management of symptoms and disease	IV - endocrine	Devices and biomaterials
Food and Drug Administration (FDA) surveys 1998	Policy planning	New types of stents	20	Treatment and management of symptoms and disease	IX - circulation	Devices and biomaterials
Food and Drug Administration (FDA) surveys 1998	Policy planning	Joint replacements	20	Treatment and management of symptoms and disease	XIII - musculoskeletal	Devices and biomaterials
Food and Drug Administration (FDA) surveys 1998	Policy planning	Image-guided therapy systems	20	Treatment and management of symptoms and disease	Other - generic	Therapeutic procedures
Food and Drug Administration (FDA) surveys 1998	Policy planning	Advanced ultrasound imaging systems	20	Diagnosis and monitoring of disease	Other - generic	Diagnostic tests and imaging
Food and Drug Administration (FDA) surveys 1998	Policy planning	Advanced optical imaging systems	20	Diagnosis and monitoring of disease	Other - generic	Diagnostic tests and imaging
Food and Drug Administration (FDA) surveys 1998	Policy planning	Advanced MR imaging systems	20	Diagnosis and monitoring of disease	Other - generic	Diagnostic tests and imaging
Food and Drug Administration (FDA) surveys 1998	Policy planning	Minimally invasive radiotherapy systems	20	Treatment and management of symptoms and disease	II - cancers	Non-surgical therapy
Food and Drug Administration (FDA) surveys 1998	Policy planning	Genetic diagnostic products	20	Diagnosis and monitoring of disease	Other - generic	Diagnostic tests and imaging
Food and Drug Administration (FDA) surveys 1998	Policy planning	Remote patient monitoring systems	20	Supporting patients receiving care (designed to be used by	Other - generic	IT systems, eHealth and mHealth

For peer review only - http://bmjopen.bmj.com/site/about/guidelines.xhtml

Appendix1: List of identified healthcare technologies

				individuals)		
Institute of the Future, 2009	Research	Bio-marker based tools will screen for future disease risk and produce new diagnoses based on probabilities	11	Diagnosis and monitoring of disease	Other - generic	Diagnostic tests and imaging
Institute of the Future, 2009	Research	Visualizations improve diet & exercise	11	Health promotion and prevention of disease	Other - generic	Non-surgical therapy
Institute of the Future, 2009	Research	GPS-enabled asthma inhalers	11	Supporting the provision of care and/or increasing service efficiency	X - respiratory	IT systems, eHealth and mHealth
Institute of the Future, 2009	Research	Workplace clinics	11	Health promotion and prevention of disease	Other - generic	Organisational programmes
Institute of the Future, 2009	Research	Urban planning for walkability, pollution reduction, neighbourood food systems	11	Health promotion and prevention of disease	Other - generic	Population programmes
Institute of the Future, 2009	Research	Report cards include Body Mass Index (BMI) scores	11	Health promotion and prevention of disease	Other - generic	Population programmes
Institute of the Future, 2009	Research	iPhone-synched medical devices	11	Supporting patients receiving care (designed to be used by individuals)	Other - generic	IT systems, eHealth and mHealth
Institute of the Future, 2009	Research	Quantifying, visualizing, and mapping health implications of environments	11	Health promotion and prevention of disease	Other - generic	Population programmes
Institute of the Future, 2009	Research	Deep brain stimulation used for neurological, behavioral issues	11	Treatment and management of symptoms and disease	VI - nervous	Devices and biomaterials
Institute of the Future, 2009	Research	Home sensors for elder care	11	Supporting patients receiving care (designed to be used by individuals)	Other - generic	IT systems, eHealth and mHealth
Institute of the Future, 2009	Research	Individually targeted therapies	11	Treatment and management of symptoms and	Other - generic	Drugs (not prophylactic vaccines)

For peer review only

Appendix1: List of identified healthcare technologies

				disease		
Institute of the Future, 2009	Research	Sensors track drug efficacy	11	Supporting patients receiving care (designed to be used by individuals)	Other - generic	Devices and biomaterials
Institute of the Future, 2009	Research	Genotypic diagnostics and risk	11	Diagnosis and monitoring of disease	Other - generic	Diagnostic tests and imaging
Institute of the Future, 2009	Research	New technologies enable individuals to experience the future effects of present-day behavior choices	11	Treatment and management of symptoms and disease	Other - generic	Unknown
Institute of the Future, 2009	Research	Regenerative medicine (general)will replace, restore, maintain, or enhance tissue and organ functions	11	Treatment and management of symptoms and disease	Other - generic	Regenerative medicine
Institute of the Future, 2009	Research	New ways of reliably measuring subjective states such as happiness, anxiety, or pain, and finding the neural correlates of personality and behavioral patterns	11	Diagnosis and monitoring of disease	VI - nervous	Diagnostic tests and imaging
Institute of the Future, 2009	Research	prototype of a prosthetic arm that can be controlled naturally and provide sensory feedback.	11	Rehabilitation	XIII - musculoskeletal	Devices and biomaterials
Stevens et al 1997	Research	Magnetic resonance imaging	1	Diagnosis and monitoring of disease	Other - generic	Diagnostic tests and imaging
Stevens et al 1997	Research	Minimally invasive surgery	1	Treatment and management of symptoms and disease	Other - generic	Therapeutic procedures
Stevens et al 1997	Research	drugs for Therapeutic refractory	1	Treatment and management of symptoms and	V - mental health	Drugs (not prophylactic vaccines)

For peer review only - http://bmjopen.bmj.com/site/about/guidelines.xhtml

Appendix1: List of identified healthcare technologies

		schizphrenia		disease		
Stevens et al 1997	Research	Implantable vascular stents	1	Treatment and management of symptoms and disease	IX - circulation	Devices and biomaterials
Stevens et al 1997	Research	Peripheral blood stem cells transplantation	1	Treatment and management of symptoms and disease	Other - generic	Regenerative medicine
Stevens et al 1997	Research	Laser Therapeutic of benign prostatic hyperplasia	1	Treatment and management of symptoms and disease	XIV - genitourinary	Non-surgical therapy
Stevens et al 1997	Research	Gene therapy advances	4	Treatment and management of symptoms and disease	Other - generic	Regenerative medicine
Stevens et al 1997	Research	Polymerase chain reaction	4	Diagnosis and monitoring of disease	Other - generic	Diagnostic tests and imaging
Stevens et al 1997	Research	Telemedicine	4	Supporting the provision of care and/or increasing service efficiency	Other - generic	IT systems, eHealth and mHealth
Stevens et al 1997	Research	Interventional radiology	1	Treatment and management of symptoms and disease	Other - generic	Non-surgical therapy
Stevens et al 1997	Research	Angioplasty	1	Treatment and management of symptoms and disease	IX - circulation	Therapeutic procedures
Stevens et al 1997	Research	Interferon for chronic granulocytic leukemia	1	Treatment and management of symptoms and disease	II - cancers	Drugs (not prophylactic vaccines)
Stevens et al 1997	Research	Interferon for hepatitis C	1	Treatment and management of symptoms and disease	I - infections	Drugs (not prophylactic vaccines)
Stevens et al 1997	Research	Lasers for dermatology	1	Treatment and management of symptoms and disease	XII - skin	Devices and biomaterials
Stevens et al 1997	Research	Adjuvant chemotherapy in lung cancer	4	Treatment and management of symptoms and disease	II - cancers	Drugs (not prophylactic vaccines)

For peer review only

Appendix1: List of identified healthcare technologies

Stevens et al 1997	Research	Near patient testing	2	Diagnosis and monitoring of disease	Other - generic	Diagnostic tests and imaging
Stevens et al 1997	Research	Revision of joint replacement	1	Treatment and management of symptoms and disease	XIII - musculoskeletal	Therapeutic procedures
Stevens et al 1997	Research	Genetic screening	4	Diagnosis and monitoring of disease	Other - generic	Diagnostic tests and imaging
Stevens et al 1997	Research	Helicobactor pylori eradication	1	Treatment and management of symptoms and disease	I - infections	Drugs (not prophylactic vaccines)
Stevens et al 1997	Research	Positron emission tomography	4	Diagnosis and monitoring of disease	Other - generic	Diagnostic tests and imaging
Stevens et al 1997	Research	Phacoemulsification	1	Treatment and management of symptoms and disease	VII - eye	Therapeutic procedures
Stevens et al 1997	Research	Cochlear implants	1	Rehabilitation	VIII - ear	Devices and biomaterials
Stevens et al 1997	Research	Paclitaxels for ovarian & breast cancer	2	Treatment and management of symptoms and disease	II - cancers	Drugs (not prophylactic vaccines)
Stevens et al 1997	Research	Bone densitometry screening	1	Diagnosis and monitoring of disease	XIII - musculoskeletal	Diagnostic tests and imaging
Stevens et al 1997	Research	Anticoagulants for atrial fibrillation	1	Health promotion and prevention of disease	IX - circulation	Drugs (not prophylactic vaccines)
Stevens et al 1997	Research	Picture archiving and communication system	4	Supporting the provision of care and/or increasing service efficiency	Other - generic	IT systems, eHealth and mHealth
Stevens et al 1997	Research	rhDNase	2	Treatment and management of symptoms and disease	IV - endocrine	Drugs (not prophylactic vaccines)
Stevens et al 1997	Research	Biomaterials in bone and joint replacement	2	Treatment and management of symptoms and disease	XIII - musculoskeletal	Devices and biomaterials
Stevens et al 1997	Research	Increased use of adjuvant chemotherapy in colorectal cancer	2	Treatment and management of symptoms and disease	II - cancers	Drugs (not prophylactic vaccines)
Stevens et al 1997	Research	Increased use of high purity factor	2	Treatment and management of	III - blood	Drugs (not prophylactic

For peer review only - http://bmjopen.bmj.com/site/about/guidelines.xhtml

Appendix1: List of identified healthcare technologies

		VIII and recombinant factor VIII in hemophilia		symptoms and disease		vaccines)
Stevens et al 1997	Research	Mirena intrauterine contraceptive device	2	Health promotion and prevention of disease	XIV - genitourinary	Drugs (not prophylactic vaccines)
Stevens et al 1997	Research	Adjuvant chemotherapy in lung cancer	4	Treatment and management of symptoms and disease	II - cancers	Drugs (not prophylactic vaccines)
Stevens et al 1997	Research	drugs for Alzheimer's disease	6	Treatment and management of symptoms and disease	V - mental health	Drugs (not prophylactic vaccines)
Stevens et al 1997	Research	Xenotransplantation	6	Treatment and management of symptoms and disease	Other - generic	Regenerative medicine
Stevens et al 1997	Research	Screening for abdominal aortic aneurysm	6	Health promotion and prevention of disease	IX - circulation	Population programmes
Stevens et al 1997	Research	Remotely controlled robots (laboratory)	6	Supporting the provision of care and/or increasing service efficiency	Other - generic	Medical equipment
Stevens et al 1997	Research	Gene therapy for cystic fibrosis	6	Treatment and management of symptoms and disease	IV - endocrine	Regenerative medicine
Stevens et al 1997	Research	New anesthetic vapors	2	Treatment and management of symptoms and disease	Other - generic	Drugs (not prophylactic vaccines)
Stevens et al 1997	Research	Magnetic resonance angiography	4	Diagnosis and monitoring of disease	IX - circulation	Diagnostic tests and imaging
Stevens et al 1997	Research	Digital radiography	4	Supporting the provision of care and/or increasing service efficiency	Other - generic	IT systems, eHealth and mHealth
Stevens et al 1997	Research	Alendronate for osteoporosis	2	Treatment and management of symptoms and disease	XIII - musculoskeletal	Drugs (not prophylactic vaccines)
Stevens et al 1997	Research	Continuous positive airways pressure	1	Treatment and management of symptoms and disease	X - respiratory	Non-surgical therapy

For peer review only - http://bmjopen.bmj.com/site/about/guidelines.xhtml

Appendix1: List of identified healthcare technologies

Stevens et al 1997	Research	Expanding metal stents for esophageal cancer	1	Treatment and management of symptoms and disease	II - cancers	Devices and biomaterials
Stevens et al 1997	Research	Community placements for severe mental illness	1	Supporting patients receiving care (designed to be used by individuals)	V - mental health	Organisational programmes
Stevens et al 1997	Research	Fludarabine in lymphomas and chronic leukemias	2	Treatment and management of symptoms and disease	II - cancers	Drugs (not prophylactic vaccines)
Stevens et al 1997	Research	Combined therapy for HIV/AIDS	2	Treatment and management of symptoms and disease	I - infections	Drugs (not prophylactic vaccines)
Stevens et al 1997	Research	Epilepsy surgery	1	Treatment and management of symptoms and disease	VI - nervous	Therapeutic procedures
Stevens et al 1997	Research	Lipid-lowering drugs for elevated cholesterol	1	Health promotion and prevention of disease	IX - circulation	Drugs (not prophylactic vaccines)
Stevens et al 1997	Research	Repair of abdominal aortic aneurysm with transfemoral endovasculate (bifurcated) graft	4	Treatment and management of symptoms and disease	IX - circulation	Devices and biomaterials
Stevens et al 1997	Research	Intracytoplasmatic sperm injection	2	Treatment and management of symptoms and disease	XIV - genitourinary	Therapeutic procedures
Stevens et al 1997	Research	Computed tomography (CT) scan advances	2	Diagnosis and monitoring of disease	Other - generic	Diagnostic tests and imaging
Stevens et al 1997	Research	Voice activated dictation technology	1	Supporting the provision of care and/or increasing service efficiency	Other - generic	Organisational programmes
Stevens et al 1997	Research	Intra-arterial metallic stents	1	Treatment and management of symptoms and disease	IX - circulation	Devices and biomaterials
Tremblay and Yiu, 2006	Policy planning	Hand-held robotic and remote ultrasound	14	Diagnosis and monitoring of disease	Other - generic	Diagnostic tests and imaging
Tremblay and Yiu, 2006	Policy planning	MRI guided catheterization	14	Diagnosis and monitoring of disease	IX - circulation	Diagnostic tests and imaging

For peer review only

Appendix1: List of identified healthcare technologies

Tremblay and Yiu, 2006	Policy planning	Image-guided therapy and surgery and robotics	14	Treatment and management of symptoms and disease	Other - generic	Therapeutic procedures
Tremblay and Yiu, 2006	Policy planning	Computed Tomographic Colonography	14	Diagnosis and monitoring of disease	XI - GI tract, dental health	Diagnostic tests and imaging
Tremblay and Yiu, 2006	Policy planning	Digital Tomosynthesis	14	Diagnosis and monitoring of disease	Other - generic	Diagnostic tests and imaging
Tremblay and Yiu, 2006	Policy planning	Molecular-level imaging	14	Diagnosis and monitoring of disease	Other - generic	Diagnostic tests and imaging
Tremblay and Yiu, 2006	Policy planning	More economical scanners with improved image processing	14	Supporting the provision of care and/or increasing service efficiency	Other - generic	Medical equipment
Tremblay and Yiu, 2006	Policy planning	4D imaging	14	Diagnosis and monitoring of disease	Other - generic	Diagnostic tests and imaging
Tremblay and Yiu, 2006	Policy planning	Faster cycle time to capture an image and general reduction in scan times	14	Supporting the provision of care and/or increasing service efficiency	Other - generic	Medical equipment
Tremblay and Yiu, 2006	Policy planning	Hybrid PET/CT increasingly replacing invasive procedures	14	Diagnosis and monitoring of disease	Other - generic	Diagnostic tests and imaging
Tremblay and Yiu, 2006	Policy planning	Stereotactic radio-surgery for early detection of coronary heart diseases	14	Diagnosis and monitoring of disease	IX - circulation	Diagnostic tests and imaging
Tremblay and Yiu, 2006	Policy planning	Magnetic Resonance Guided Focused Ultrasound for Imaging of inflammation	14	Diagnosis and monitoring of disease	Other - generic	Diagnostic tests and imaging
Tremblay and Yiu, 2006	Policy planning	Wireless home-health monitoring	14	Supporting patients receiving care (designed to be used by individuals)	Other - generic	IT systems, eHealth and mHealth
Tremblay and Yiu, 2006	Policy planning	E-health diagnostic platform	14	Supporting the provision of care and/or increasing service efficiency	Other - generic	IT systems, eHealth and mHealth
Tremblay and Yiu, 2006	Policy planning	Hand-held gamma camera	14	Diagnosis and monitoring of	Other - generic	Diagnostic tests and imaging

For peer review only - http://bmjopen.bmj.com/site/about/guidelines.xhtml

Appendix1: List of identified healthcare technologies

For peer review only

				disease		
Tremblay and Yiu, 2006	Policy planning	"one-stop" diagnosis and Therapeutic	14	Supporting the provision of care and/or increasing service efficiency	Other - generic	Organisational programmes
Tremblay and Yiu, 2006	Policy planning	customised hybrid systems for specific clinical situations	14	Unknown	Other - generic	Organisational programmes
Tremblay and Yiu, 2006	Policy planning	Near Infra-red Spectroscopy	14	Diagnosis and monitoring of disease	Other - generic	Diagnostic tests and imaging
Tremblay and Yiu, 2006	Policy planning	Spatially-resolved Spectroscopy	14	Diagnosis and monitoring of disease	Other - generic	Diagnostic tests and imaging
Tremblay and Yiu, 2006	Policy planning	Optical Coherence Tomography	14	Diagnosis and monitoring of disease	Other - generic	Diagnostic tests and imaging
Tremblay and Yiu, 2006	Policy planning	Capsule Endoscopy	14	Diagnosis and monitoring of disease	Other - generic	Devices and biomaterials
Tremblay and Yiu, 2006	Policy planning	Tele-echography	14	Supporting the provision of care and/or increasing service efficiency	Other - generic	IT systems, eHealth and mHealth
Tremblay and Yiu, 2006	Policy planning	Tele-colposcopy	14	Supporting the provision of care and/or increasing service efficiency	Other - generic	IT systems, eHealth and mHealth
Tremblay and Yiu, 2006	Policy planning	Electronic Nose	14	Diagnosis and monitoring of disease	Other - generic	Diagnostic tests and imaging
Tremblay and Yiu, 2006	Policy planning	New contrast agents for Rubidium	14	Diagnosis and monitoring of disease	Other - generic	Diagnostic tests and imaging
Tremblay and Yiu, 2006	Policy planning	Hyperpolarised gases	14	Diagnosis and monitoring of disease	Other - generic	Diagnostic tests and imaging
Tremblay and Yiu, 2006	Policy planning	Terahertz waves	14	Diagnosis and monitoring of disease	Other - generic	Diagnostic tests and imaging
Tremblay and Yiu, 2006	Policy planning	Microwaves, light spectrum	14	Unknown	Other - generic	Devices and biomaterials
Tremblay and Yiu, 2006	Policy planning	Hyperpolarised imaging	14	Diagnosis and monitoring of disease	Other - generic	Diagnostic tests and imaging
Tremblay and Yiu, 2006	Policy planning	Specific advances in MRI, Ultrasound, Radiography…etc	14	Diagnosis and monitoring of disease	Other - generic	Diagnostic tests and imaging

For peer review only - http://bmjopen.bmj.com/site/about/guidelines.xhtml

Appendix1: List of identified healthcare technologies

Tremblay and Yiu, 2006	Policy planning	Reduction in use of invasive diagnostic procedures	14	Supporting the provision of care and/or increasing service efficiency	Other - generic	Organisational programmes
Tremblay and Yiu, 2006	Policy planning	Diagnosis of kidney disorders without renal biopsy	14	Diagnosis and monitoring of disease	XIV - genitourina ry	Unknown
Tremblay and Yiu, 2006	Policy planning	Ex vivo molecular-computer diagnosis	14	Diagnosis and monitoring of disease	Other - generic	IT systems, eHealth and mHealth
Tremblay and Yiu, 2006	Policy planning	Smart-drug delivery systems	14	Treatment and management of symptoms and disease	Other - generic	Devices and biomaterials
Tremblay and Yiu, 2006	Policy planning	Polymer-encapsulated, bioconjugated quantum dots	9	Diagnosis and monitoring of disease	Other - generic	Diagnostic tests and imaging
Tremblay and Yiu, 2006	Policy planning	Patient accessed universal health records	4	Supporting the provision of care and/or increasing service efficiency	Other - generic	Organisational programmes
Tremblay and Yiu, 2006	Policy planning	In silico-in vivo integration (lab on-a-chip)	14	Diagnosis and monitoring of disease	Other - generic	Diagnostic tests and imaging
Tremblay and Yiu, 2006	Policy planning	Wide deployment of non-invasive technologies and biosensors	9	Supporting the provision of care and/or increasing service efficiency	Other - generic	Devices and biomaterials
Tremblay and Yiu, 2006	Policy planning	Direct consumer access to diagnostic tests	6	Supporting patients receiving care (designed to be used by individuals)	Other - generic	Organisational programmes
Tremblay and Yiu, 2006	Policy planning	Molecular imaging with single molecular pecision	9	Diagnosis and monitoring of disease	Other - generic	Diagnostic tests and imaging
Tremblay and Yiu, 2006	Policy planning	diagnostic testing throughout the care pathway	7	Supporting the provision of care and/or increasing service efficiency	Other - generic	Diagnostic tests and imaging
Tremblay and Yiu, 2006	Policy planning	Neuro-imaging at cellular level of brain processes (thoughts)	14	Diagnosis and monitoring of disease	VI - nervous	Diagnostic tests and imaging
Tremblay and Yiu, 2006	Policy planning	Molecular-computers used to design smart therapies	14	Other	Other - generic	Drugs (not prophylactic vaccines)

For peer review only - http://bmjopen.bmj.com/site/about/guidelines.xhtml

Appendix1: List of identified healthcare technologies

Tremblay and Yiu, 2006	Policy planning	Remote internet diagnosis based on data received from patient's home	9	Supporting patients receiving care (designed to be used by individuals)	Other - generic	IT systems, eHealth and mHealth
Tremblay and Yiu, 2006	Policy planning	Molecular diagnosis	14	Diagnosis and monitoring of disease	Other - generic	Diagnostic tests and imaging
Tremblay and Yiu, 2006	Policy planning	diagnostic -guided therapy for individualised medicine	14	Treatment and management of symptoms and disease	Other - generic	Drugs (not prophylactic vaccines)
Tremblay and Yiu, 2006	Policy planning	Computer-assisted diagnosis with pattern recognition	14	Diagnosis and monitoring of disease	Other - generic	IT systems, eHealth and mHealth
Tremblay and Yiu, 2006	Policy planning	Self-propelled micro-machines for diagnosis and Therapeutic	9	Unknown	Other - generic	Devices and biomaterials
Tremblay and Yiu, 2006	Policy planning	diagnostic bio-chip for cancer	9	Diagnosis and monitoring of disease	II - cancers	Diagnostic tests and imaging
Tremblay and Yiu, 2006	Policy planning	Investigation of thrombosis and vascular pathology using 3D tomography with remote controlled micro-instruments	14	Diagnosis and monitoring of disease	IX - circulation	Devices and biomaterials
Tremblay and Yiu, 2006	Policy planning	Personal care nurse-robots	12	Supporting patients receiving care (designed to be used by individuals)	Other - generic	Medical equipment
Tremblay and Yiu, 2006	Policy planning	Functional combination of targeting and imaging	14	Diagnosis and monitoring of disease	Other - generic	Diagnostic tests and imaging
Tremblay and Yiu, 2006	Policy planning	Intelligent and context sensitive clinical systems	14	Supporting the provision of care and/or increasing service efficiency	Other - generic	Organisational programmes
Tremblay and Yiu, 2006	Policy planning	Personal access to genomic information	14	Supporting patients receiving care (designed to be used by individuals)	Other - generic	Unknown
Tremblay and Yiu, 2006	Policy planning	Development of effective gene	9	Treatment and management of	Other - generic	Regenerative medicine

For peer review only - http://bmjopen.bmj.com/site/about/guidelines.xhtml

Appendix1: List of identified healthcare technologies

		therapies		symptoms and disease		
Science & Technology Foresight survey Japan 1997	Policy planning	Widespread use of scientific guidelines for adult-disease-preventing lifestyles (nutrition, rest and exercise)	9	Health promotion and prevention of disease	Other - generic	Organisational programmes
Science & Technology Foresight survey Japan 1997	Policy planning	Practical use of chemotherapy that brings complete remission to digestive organ cancer with low drug-responsiveness	18	Treatment and management of symptoms and disease	II - cancers	Drugs (not prophylactic vaccines)
Science & Technology Foresight survey Japan 1997	Policy planning	Widespread use of techniques to overcome the drug resistance of malignant tumours	16	Treatment and management of symptoms and disease	II - cancers	Drugs (not prophylactic vaccines)
Science & Technology Foresight survey Japan 1997	Policy planning	Widespread use of drugs that cure viral liver disease	13	Treatment and management of symptoms and disease	XI - GI tract, dental health	Drugs (not prophylactic vaccines)
Science & Technology Foresight survey Japan 1997	Policy planning	Widespread use of biological and immunological therapy effective for cancer	14	Treatment and management of symptoms and disease	II - cancers	Drugs (not prophylactic vaccines)
Science & Technology Foresight survey Japan 1997	Policy planning	Development of effective methods of preventing Alzheimer's disease	16	Health promotion and prevention of disease	V - mental health	Unknown
Science & Technology Foresight survey Japan 1997	Policy planning	Possible to cure allergic diseases	21	Treatment and management of symptoms and disease	III - blood	Unknown
Science & Technology Foresight survey Japan 1997	Policy planning	Development of an HIV vaccine	10	Health promotion and prevention of disease	I - infections	Prophylactic vaccines
Science & Technology Foresight survey Japan 1997	Policy planning	Widespread use of gene therapy against malignant tumors	17	Treatment and management of symptoms and disease	II - cancers	Regenerative medicine

For peer review only - http://bmjopen.bmj.com/site/about/guidelines.xhtml

Appendix1: List of identified healthcare technologies

Science & Technology Foresight survey Japan 1997	Policy planning	Widespread use of prevention methods for the contraction of diabetic complications	11	Health promotion and prevention of disease	IV - endocrine	Unknown
Science & Technology Foresight survey Japan 1997	Policy planning	Practical use of anti-AIDS therapy	12	Treatment and management of symptoms and disease	I - infections	Drugs (not prophylactic vaccines)
Science & Technology Foresight survey Japan 1997	Policy planning	Widespread use of an early cancer diagnosis technique based on biochemical examination	10	Diagnosis and monitoring of disease	II - cancers	Diagnostic tests and imaging
Science & Technology Foresight survey Japan 1997	Policy planning	Widespread use of a technique to eliminate viruses from blood	12	Treatment and management of symptoms and disease	I - infections	Therapeutic procedures
Science & Technology Foresight survey Japan 1997	Policy planning	Practical use of prevention methods for stress-induced mental disorders	14	Health promotion and prevention of disease	V - mental health	Unknown
Science & Technology Foresight survey Japan 1997	Policy planning	Practical use of a method to quantitatively assess the level of aging (biological age) in relation to chronological age.	9	Diagnosis and monitoring of disease	Other - generic	Unknown
Science & Technology Foresight survey Japan 1997	Policy planning	Practical use of a preventive measure against diabetic complications.	14	Health promotion and prevention of disease	IV - endocrine	Unknown
Science & Technology Foresight survey Japan 1997	Policy planning	Widespread use of presbyopia prevention methods	29	Health promotion and prevention of disease	VII - eye	Unknown
Science & Technology Foresight survey Japan 1997	Policy planning	Widespread use of a laser-applied tooth decay treatment method	14	Treatment and management of symptoms and disease	XI - GI tract, dental health	Devices and biomaterials
Science & Technology Foresight survey Japan 1997	Policy planning	Widespread method of prevention against a premature delivery through	8	Health promotion and prevention of disease	XV - pregnancy	Organisational programmes

For peer review only

For peer review only - http://bmjopen.bmj.com/site/about/guidelines.xhtml

Appendix1: List of identified healthcare technologies

		the progression of management system of fetal information.				
Science & Technology Foresight survey Japan 1997	Policy planning	Practical use of the prevention against congenital anomaly originating in the embryonic or fetal period.	19	Health promotion and prevention of disease	XV - pregnancy	Unknown
Science & Technology Foresight survey Japan 1997	Policy planning	Widespread use of preventive measure for cerebral palsy	19	Health promotion and prevention of disease	VI - nervous	Unknown
Science & Technology Foresight survey Japan 1997	Policy planning	Widespread use of a cancer spread diagnosis technique based on 3-D imaging.	7	Diagnosis and monitoring of disease	II - cancers	Diagnostic tests and imaging
Science & Technology Foresight survey Japan 1997	Policy planning	Widespread use of a cancer risk assessment technique based on genetic analysis.	11	Diagnosis and monitoring of disease	II - cancers	Unknown
Science & Technology Foresight survey Japan 1997	Policy planning	Widespread use of against of cancer cells drug sensitivity measurement method.	9	Unknown	II - cancers	Unknown
Science & Technology Foresight survey Japan 1997	Policy planning	Widespread use of automatic testing equipment for cancer cytodiagnosis.	9	Diagnosis and monitoring of disease	II - cancers	Diagnostic tests and imaging
Science & Technology Foresight survey Japan 1997	Policy planning	Practical use of classification and stage determination of schizophrenia based on diagnostic imaging.	24	Diagnosis and monitoring of disease	V - mental health	Diagnostic tests and imaging
Science & Technology Foresight survey Japan 1997	Policy planning	Practical use of diagnosing methods for determining the level and spread of arteriosclerosis	9	Diagnosis and monitoring of disease	IX - circulation	Diagnostic tests and imaging

For peer review only - http://bmjopen.bmj.com/site/about/guidelines.xhtml

Appendix1: List of identified healthcare technologies

		focused by a non-invasive process				
Science & Technology Foresight survey Japan 1997	Policy planning	Establishment of a correct diagnostic measure for kidney diseases without renal biopsy.	19	Diagnosis and monitoring of disease	XIV - genitourinary	Diagnostic tests and imaging
Science & Technology Foresight survey Japan 1997	Policy planning	Development of a capsule-type total gastro-intestinal tract monitoring system.	6	Diagnosis and monitoring of disease	XI - GI tract, dental health	Devices and biomaterials
Science & Technology Foresight survey Japan 1997	Policy planning	Practical use of specialist-level medical diagnosis assistance systems.	9	Supporting the provision of care and/or increasing service efficiency	Other - generic	IT systems, eHealth and mHealth
Science & Technology Foresight survey Japan 1997	Policy planning	Development of non-invasive cell-level diagnostic imaging.	8	Diagnosis and monitoring of disease	Other - generic	Diagnostic tests and imaging
Science & Technology Foresight survey Japan 1997	Policy planning	Practical use of a non-invasive technique to obtain blood biochemical examination data using body surface sensors.	9	Diagnosis and monitoring of disease	III - blood	Devices and biomaterials
Science & Technology Foresight survey Japan 1997	Policy planning	Practical use of a rapid diagnostic method for rejection in organ and tissue transplantation.	6	Diagnosis and monitoring of disease	III - blood	Unknown
Science & Technology Foresight survey Japan 1997	Policy planning	Widespread use of malaria vaccines.	10	Health promotion and prevention of disease	I - infections	Prophylactic vaccines
Science & Technology Foresight survey Japan 1997	Policy planning	Development of an effective insulin that can be administered orally.	16	Treatment and management of symptoms and disease	IV - endocrine	Drugs (not prophylactic vaccines)
Science & Technology Foresight survey Japan 1997	Policy planning	Practical use of gene therapy for diabetes.	16	Treatment and management of symptoms and disease	IV - endocrine	Regenerative medicine

For peer review only - http://bmjopen.bmj.com/site/about/guidelines.xhtml

Appendix1: List of identified healthcare technologies

Science & Technology Foresight survey Japan 1997	Policy planning	Widespread use of gene therapy for familial hypercholesterolemia.	14	Treatment and management of symptoms and disease	IX - circulation	Regenerative medicine
Science & Technology Foresight survey Japan 1997	Policy planning	Development of radiosensitizers effective in cancer treatment.	8	Treatment and management of symptoms and disease	II - cancers	Therapeutic procedures
Science & Technology Foresight survey Japan 1997	Policy planning	Widespread use of heavy particle against cancer	14	Treatment and management of symptoms and disease	II - cancers	Non-surgical therapy
Science & Technology Foresight survey Japan 1997	Policy planning	Development of a technique to cause drug delivery to targeted sites in the brain.	19	Treatment and management of symptoms and disease	Other - generic	Drugs (not prophylactic vaccines)
Science & Technology Foresight survey Japan 1997	Policy planning	Practical use of a safe pain control method without side effects	12	Treatment and management of symptoms and disease	Other - generic	Unknown
Science & Technology Foresight survey Japan 1997	Policy planning	Widespread use of gene therapy for muscular dystrophy.	24	Treatment and management of symptoms and disease	VI - nervous	Regenerative medicine
Science & Technology Foresight survey Japan 1997	Policy planning	Development of effective treatment for amyotrophic lateral sclerosis.	31	Treatment and management of symptoms and disease	VI - nervous	Unknown
Science & Technology Foresight survey Japan 1997	Policy planning	Practical use of artificial muscles for limbs.	21	Rehabilitation	XXI - screening, rehab	Devices and biomaterials
Science & Technology Foresight survey Japan 1997	Policy planning	Practical use of a technique to induce the differentiation of fibroblasts into cardiac muscle and its application to the treatment of myocardial infarction.	20	Treatment and management of symptoms and disease	IX - circulation	Regenerative medicine
Science & Technology Foresight survey Japan 1997	Policy planning	Development of hybrid-type artificial endocrine organs in which living	11	Treatment and management of symptoms and disease	IV - endocrine	Regenerative medicine

For peer review only - http://bmjopen.bmj.com/site/about/guidelines.xhtml

Appendix1: List of identified healthcare technologies

		cells and artificial objects coexist.				
Science & Technology Foresight survey Japan 1997	Policy planning	Widespread use of functional blood purifiers that selectively remove specified components from blood	19	Treatment and management of symptoms and disease	III - blood	Devices and biomaterials
Science & Technology Foresight survey Japan 1997	Policy planning	Widespread use of artificial hemoglobin as a red blood cell substitute.	15	Treatment and management of symptoms and disease	III - blood	Regenerative medicine
Science & Technology Foresight survey Japan 1997	Policy planning	Development of fully implanted artificial heart	14	Treatment and management of symptoms and disease	IX - circulation	Devices and biomaterials
Science & Technology Foresight survey Japan 1997	Policy planning	Practical use of fully implanted artificial lungs.	29	Treatment and management of symptoms and disease	X - respiratory	Devices and biomaterials
Science & Technology Foresight survey Japan 1997	Policy planning	Practical use of fully implanted artificial kidneys	26	Treatment and management of symptoms and disease	XIV - genitourina ry	Devices and biomaterials
Science & Technology Foresight survey Japan 1997	Policy planning	Practical use of fully implanted artificial pancreas	12	Treatment and management of symptoms and disease	XI - GI tract, dental health	Devices and biomaterials
Science & Technology Foresight survey Japan 1997	Policy planning	Widespread use of prevention and treatment methods for tooth decay and periodontitis to ensure the retention of 20 or more teeth at 80 year of age	14	Health promotion and prevention of disease	XI - GI tract, dental health	Population programmes
Science & Technology Foresight survey Japan 1997	Policy planning	Widespread use of a technique to store, cultivate and transplant one's own tissues.	15	Treatment and management of symptoms and disease	Other - generic	Regenerative medicine
Science & Technology Foresight survey Japan 1997	Policy planning	Practical use of implanted bladder control devices	12	Treatment and management of symptoms and disease	XIV - genitourina ry	Devices and biomaterials

For peer review only

Appendix1: List of identified healthcare technologies

Science & Technology Foresight survey Japan 1997	Policy planning	Practical use of gene therapy for genetic disorders	19	Treatment and management of symptoms and disease	Other - generic	Regenerative medicine
Science & Technology Foresight survey Japan 1997	Policy planning	Availability of genetherapy based on oral delivery	24	Treatment and management of symptoms and disease	Other - generic	Regenerative medicine
Science & Technology Foresight survey Japan 1997	Policy planning	Practical use of heterogeneous organ transplantation as means of treatment.	21	Treatment and management of symptoms and disease	Other - generic	Therapeutic procedures
Science & Technology Foresight survey Japan 1997	Policy planning	Widespread use of a worldwide organ supply system	12	Supporting the provision of care and/or increasing service efficiency	Other - generic	Organisational programmes
Science & Technology Foresight survey Japan 1997	Policy planning	Development of a partial tissue destruction method non-invasive to the living body.	6	Treatment and management of symptoms and disease	Other - generic	Non-surgical therapy
Science & Technology Foresight survey Japan 1997	Policy planning	Practical use of implanted artificial cochleas effective for both conductive hearing loss and perceptive hearing loss.	14	Rehabilitation	VIII - ear	Devices and biomaterials
Science & Technology Foresight survey Japan 1997	Policy planning	Development of a device to sense the needs of demented aged people.	21	Supporting patients receiving care (designed to be used by individuals)	V - mental health	Devices and biomaterials
Science & Technology Foresight survey Japan 1997	Policy planning	Development of controlling devices which help the cooperative muscular actions enabling object-oriented movement.	14	Rehabilitation	XXI - screening, rehab	Devices and biomaterials
Science & Technology Foresight survey Japan 1997	Policy planning	Widespread use of artificial legs featuring a small power source and computer control.	11	Rehabilitation	XXI - screening, rehab	Devices and biomaterials

For peer review only - http://bmjopen.bmj.com/site/about/guidelines.xhtml

Appendix1: List of identified healthcare technologies

Science & Technology Foresight survey Japan 1997	Policy planning	Practical use of a blood cell production and supply technique based on industrial-scale cultivation.	15	Treatment and management of symptoms and disease	III - blood	Regenerative medicine
Science & Technology Foresight survey Japan 1997	Policy planning	Practical use of systems for monitoring health condition and providing information for an appropriate diagnosis at home in event of accident or diseases.	11	Supporting patients receiving care (designed to be used by individuals)	XIX - injury	IT systems, eHealth and mHealth
Science & Technology Foresight survey Japan 1997	Policy planning	Widespread use of portable conversational speech interpretation systems.	24	Unknown	Other - generic	IT systems, eHealth and mHealth
Science & Technology Foresight survey Japan 1997	Policy planning	Widespread use of robots that care for people with severe physical and mental disabilities.	12	Supporting patients receiving care (designed to be used by individuals)	Other - generic	Medical equipment
Science & Technology Foresight survey Japan 1997	Policy planning	Advancements in patient and hospital information management, leading to a reduction in waiting time at Japanese hospitals to 15 minutes or less.	7	Supporting the provision of care and/or increasing service efficiency	Other - generic	Organisational programmes
Science & Technology Foresight survey Japan 1997	Policy planning	Practical use in Japan of an ID card system that thoroughly covers an individual's health conditions and medical data.	9	Supporting the provision of care and/or increasing service efficiency	Other - generic	Organisational programmes
Science & Technology Foresight survey	Policy planning	Elucidation of the biological clock, leading to drug	9	Supporting patients receiving care (designed to	Other - generic	Unknown

For peer review only - http://bmjopen.bmj.com/site/about/guidelines.xhtml

Appendix1: List of identified healthcare technologies

Japan 1997		administration that takes it into consideration		be used by individuals)		
Science & Technology Foresight survey Japan 1997	Policy planning	Practical application of the production of artificial cells with organ characteristics.	29	Treatment and management of symptoms and disease	Other - generic	Regenerative medicine
Science & Technology Foresight survey Japan 1997	Policy planning	Practical use of anti-AIDS therapy	12	Treatment and management of symptoms and disease	I - infections	Drugs (not prophylactic vaccines)
Science & Technology Foresight survey Japan 1997	Policy planning	Practical use of a rapid diagnostic method for rejection in organ and tissue transplantation	10	Diagnosis and monitoring of disease	Other - generic	Diagnostic tests and imaging
Science & Technology Foresight survey Japan 1997	Policy planning	Development of anti-cancer agents which target the manifestation functions of cancer genes	10	Treatment and management of symptoms and disease	II - cancers	Drugs (not prophylactic vaccines)
Science & Technology Foresight survey Japan 1997	Policy planning	Utilization of information about the gene structure of each individual patient in diagnosis and treatment	16	Diagnosis and monitoring of disease	Other - generic	Organisational programmes
Science & Technology Foresight survey Japan 1997	Policy planning	Development of technology to regenerate organs or individuals from separated animal cells	20	Treatment and management of symptoms and disease	Other - generic	Regenerative medicine
Science & Technology Foresight survey Japan 1997	Policy planning	Practical use of methods for directly determining DNA sequences by physical means such as X-rays.	14	Diagnosis and monitoring of disease	Other - generic	Diagnostic tests and imaging
Science & Technology Foresight survey Japan 1997	Policy planning	Control of signal transduction in the carcinogenesis of cells, and widespread use of treatment	20	Treatment and management of symptoms and disease	II - cancers	Drugs (not prophylactic vaccines)

For peer review only

Appendix1: List of identified healthcare technologies

		methods for dysdifferentiating carcinogenic cells.				
Science & Technology Foresight survey Japan 1997	Policy planning	Complete understanding of the factors contributing to stem cell multiplication, and widespread use of the practice of multiplying stem cells, as necessary, in test tubes and using them for treatment purpose	15	Treatment and management of symptoms and disease	Other - generic	Regenerative medicine
Science & Technology Foresight survey Japan 1997	Policy planning	Development of technology capable of synthesizing living cells by using only artificially produced chemical compounds.	31	Treatment and management of symptoms and disease	Other - generic	Regenerative medicine
Science & Technology Foresight survey Japan 1997	Policy planning	Practical use of elementary particle beam technology in genetic engineering.	19	Treatment and management of symptoms and disease	Other - generic	Regenerative medicine
Science & Technology Foresight survey Japan 1997	Policy planning	Development of drugs capable of preventing the occurrence of certain types of cancer.	11	Health promotion and prevention of disease	II - cancers	Drugs (not prophylactic vaccines)
Science & Technology Foresight survey Japan 1997	Policy planning	Practical use of effective means to prevent metastasis of cancer.	14	Treatment and management of symptoms and disease	II - cancers	Unknown
Science & Technology Foresight survey Japan 1997	Policy planning	Widespread use of technologies for long-term (semi-permanent) culturing and preservation of	19	Treatment and management of symptoms and disease	Other - generic	Regenerative medicine

For peer review only - http://bmjopen.bmj.com/site/about/guidelines.xhtml

Appendix1: List of identified healthcare technologies

		organs.				
Science & Technology Foresight survey Japan 1997	Policy planning	Practical use of artificial organs (pancreases, kidneys, livers, etc.) incorporating human cells and tissues	14	Treatment and management of symptoms and disease	Other - generic	Regenerative medicine
Science & Technology Foresight survey Japan 1997	Policy planning	Development of an entirely implantable artificial kidney.	18	Treatment and management of symptoms and disease	XIV - genitourinary	Regenerative medicine
Science & Technology Foresight survey Japan 1997	Policy planning	Development of an artificial cornea, allowing the visually impaired to regain their sight.	25	Treatment and management of symptoms and disease	VII - eye	Devices and biomaterials
Science & Technology Foresight survey Japan 1997	Policy planning	Development of artificial muscle elements.	21	Treatment and management of symptoms and disease	Other - generic	Regenerative medicine
Science & Technology Foresight survey Japan 1997	Policy planning	Establishment of interface technology between neural information and artificial organism structures.	25	Treatment and management of symptoms and disease	VI - nervous	Medical equipment
Science & Technology Foresight survey Japan 1997	Policy planning	Development of neural-computers with new logic structures modeled on brain functions.	24	Unknown	VI - nervous	IT systems, eHealth and mHealth
Science & Technology Foresight survey Japan 1997	Policy planning	Development of interfaces enabling direct linkage between the computer and the brain.	21	Treatment and management of symptoms and disease	XXI - screening, rehab	Devices and biomaterials
Science & Technology Foresight survey Japan 1997	Policy planning	Development of electric circuits with similar self-organization and self-restoration functions as neural networks.	14	Treatment and management of symptoms and disease	VI - nervous	Devices and biomaterials

For peer review only - http://bmjopen.bmj.com/site/about/guidelines.xhtml

Appendix1: List of identified healthcare technologies

Science & Technology Foresight survey Japan 1997	Policy planning	Development of diagnostic and medical treatment micromachines capable of traveling on their own inside organisms (body cavity organs).	24	Diagnosis and monitoring of disease	Other - generic	Devices and biomaterials
Science & Technology Foresight survey Japan 1997	Policy planning	Widespread use of remote surgery systems utilizing virtual reality technology.	14	Supporting the provision of care and/or increasing service efficiency	Other - generic	IT systems, eHealth and mHealth
Science & Technology Foresight survey Japan 1997	Policy planning	Widespread use of vicarious experience devices (using virtual reality technology) which allow bed-ridden patients to take vacations, etc.	9	Rehabilitation	XXI - screening, rehab	IT systems, eHealth and mHealth
Science & Technology Foresight survey Japan 1997	Policy planning	Widespread use of methods for controlling the immune system locally	14	Treatment and management of symptoms and disease	III - blood	Unknown
Science & Technology Foresight survey Japan 1997	Policy planning	Development of effective Therapeutic for amyotrophic lateral sclerosis	21	Treatment and management of symptoms and disease	VI - nervous	Drugs (not prophylactic vaccines)
Science & Technology Foresight survey Japan 1997	Policy planning	Practical use of batteries of artificial organs implanted in the living body	17	Treatment and management of symptoms and disease	Other - generic	Devices and biomaterials
Science & Technology Foresight survey Japan 1997	Policy planning	Development of artificial liver (external devices supporting liver functions) usable on a long-term, continuous basis	19	Treatment and management of symptoms and disease	XI - GI tract, dental health	Devices and biomaterials
Science & Technology Foresight survey Japan 1997	Policy planning	Practical use of heterogeneous organ transplantation as means of Therapeutic	19	Treatment and management of symptoms and disease	Other - generic	Therapeutic procedures

For peer review only - http://bmjopen.bmj.com/site/about/guidelines.xhtml

Appendix1: List of identified healthcare technologies

Science & Technology Foresight survey Japan 1997	Policy planning	Development of artificial eyes featuring electronic circuitry that can be connected to nerve and brain cells	24	Rehabilitation	VII - eye	Devices and biomaterials
Science & Technology Foresight survey Japan 1997	Policy planning	Development of methods for recombining disconnected central nerves	29	Rehabilitation	VI - nervous	Therapeutic procedures
Loveridge et al 1998	Policy planning	Practical use of directly visualising molecular structure at an atomic level (e.g. ultramicroscopy)	21	Diagnosis and monitoring of disease	Other - generic	Diagnostic tests and imaging
Loveridge et al 1998	Policy planning	At least 3 common diseases will be treated by portable or implanted systems that combine individually tailored drug delivery with automatic feedback control from a devices continuously monitoring the condition (e.g. diabetes control)	21	Treatment and management of symptoms and disease	Other - generic	Devices and biomaterials
Loveridge et al 1998	Policy planning	Over-the-counter nucleicacid-based tests suitable for self-administration become available for the first time	21	Supporting patients receiving care (designed to be used by individuals)	Other - generic	IT systems, eHealth and mHealth
Loveridge et al 1998	Policy planning	Results of more than 50% of all types of diagnostic tests are available at the point of need within one hour	21	Diagnosis and monitoring of disease	Other - generic	Diagnostic tests and imaging

For peer review only - http://bmjopen.bmj.com/site/about/guidelines.xhtml

Appendix1: List of identified healthcare technologies

Loveridge et al 1998	Policy planning	Widespread use of real time 3-D high resolutiom imaging of physiological and metabolic functions	21	Diagnosis and monitoring of disease	Other - generic	Diagnostic tests and imaging
Loveridge et al 1998	Policy planning	First practical use of non-invasive spectroscopy for in situ chemical pathology	21	Diagnosis and monitoring of disease	Other - generic	Diagnostic tests and imaging
Loveridge et al 1998	Policy planning	Practical use of biosensors in a wide range of medical manufacturing and environmental applications	21	Unknown	Other - generic	Devices and biomaterials
Loveridge et al 1998	Policy planning	All common single genes diseases can be treated successfully by gene theraby	21	Treatment and management of symptoms and disease	Other - generic	Drugs (not prophylactic vaccines)
Loveridge et al 1998	Policy planning	Practical use of gene therapy is extended to the treatment of 30% of major life threatening diseases	21	Treatment and management of symptoms and disease	Other - generic	Drugs (not prophylactic vaccines)
Loveridge et al 1998	Policy planning	Nucleic acid based diagnostic techniques account for 10% of all diagnostic e work load	21	Diagnosis and monitoring of disease	Other - generic	Organisational programmes
Loveridge et al 1998	Policy planning	First practical use of therapies based on purpose·designed non-peptide molecules which mimic the activity of peptides.	21	Treatment and management of symptoms and disease	Other - generic	Drugs (not prophylactic vaccines)
Loveridge et al 1998	Policy planning	Widespread use of polypeptide drugs which are delivered orally and targeted to	21	Treatment and management of symptoms and disease	Other - generic	Drugs (not prophylactic vaccines)

For peer review only - http://bmjopen.bmj.com/site/about/guidelines.xhtml

Appendix1: List of identified healthcare technologies

		specific tissues				
Loveridge et al 1998	Policy planning	Widespread use of cosmaceuticals with scientifically proven effectiveness to treat the sypmtoms of skin ageing (e.g. wrinkles)	21	Health promotion and prevention of disease	XII - skin	Drugs (not prophylactic vaccines)
Loveridge et al 1998	Policy planning	Widespread use of improved technologies for early diagnosis increases the 5-year survival rate of cancer patients by 50%	21	Diagnosis and monitoring of disease	II - cancers	Diagnostic tests and imaging
Loveridge et al 1998	Policy planning	Practical use of drugs modulating cellular systems to treat cancer	21	Treatment and management of symptoms and disease	II - cancers	Drugs (not prophylactic vaccines)
Loveridge et al 1998	Policy planning	Widespread use of nutritional supplements or special diets based on an understading of the role of nutrients such as anti-oxidants in cancer prevention	21	Health promotion and prevention of disease	II - cancers	Population programmes
Loveridge et al 1998	Policy planning	Practical use of substances that contain rather eleminate cancer (e.g. use agents that control metastasis)	21	Treatment and management of symptoms and disease	II - cancers	Drugs (not prophylactic vaccines)
Loveridge et al 1998	Policy planning	New methods of treating arterial occlusion and myocardial infarction dramatically reduce morbidity from cardiovascular diseases in 40-50 age group	21	Treatment and management of symptoms and disease	IX - circulation	Unknown

For peer review only - http://bmjopen.bmj.com/site/about/guidelines.xhtml

Appendix1: List of identified healthcare technologies

Loveridge et al 1998	Policy planning	50% of the UK population receive individual risk assessment for cardiovascular disease based on genetic screening and lifestyle measures	21	Health promotion and prevention of disease	IX - circulation	Population programmes
Loveridge et al 1998	Policy planning	Understanding the immune system is sufficietly developed to allow routine transplantation without the use of immuno-suupression	21	Treatment and management of symptoms and disease	III - blood	Therapeutic procedures
Loveridge et al 1998	Policy planning	Practical use of xenotransplantation of organs from animals to human	21	Treatment and management of symptoms and disease	Other - generic	Regenerative medicine
Loveridge et al 1998	Policy planning	Incidence of each of the 3 major viral diseases is reduced by 90% by new vaccines or drug development	21	Health promotion and prevention of disease	I - infections	Prophylactic vaccines
Loveridge et al 1998	Policy planning	Practical use of technologies for oral administration of multi-valent vaccines for the most important childhood illnesses	21	Health promotion and prevention of disease	I - infections	Prophylactic vaccines
Loveridge et al 1998	Policy planning	Widespread introduction of malaria vaccine reduces the worldwide incidence of the diseases by 50%	21	Health promotion and prevention of disease	I - infections	Prophylactic vaccines
Loveridge et al 1998	Policy planning	First effective vaccine of HIV becomes available	21	Health promotion and prevention of disease	I - infections	Prophylactic vaccines
Loveridge et al 1998	Policy planning	Development of drugs with proven	21	Treatment and management of	V - mental health	Drugs (not prophylactic

For peer review only - http://bmjopen.bmj.com/site/about/guidelines.xhtml

Appendix1: List of identified healthcare technologies

		efficacy for enhancing cognitive performance		symptoms and disease		vaccines)
Loveridge et al 1998	Policy planning	50% of patients presenting with mental illness such as schizphrenia and depression are diagnosed using objective genetic, biochemical or biophysical tests	21	Diagnosis and monitoring of disease	V - mental health	Diagnostic tests and imaging
Loveridge et al 1998	Policy planning	50% of surgical interventions carried out by minimum-access technique	21	Treatment and management of symptoms and disease	Other - generic	Therapeutic procedures
Loveridge et al 1998	Policy planning	10% of surgical interventions are carried out by robotic techniques	21	Treatment and management of symptoms and disease	Other - generic	Therapeutic procedures
Loveridge et al 1998	Policy planning	Development of biocompatible materials for implants which elicit the required host response (e.g. non-thrombogenic or stimulation of host repair mechanisms).	21	Treatment and management of symptoms and disease	Other - generic	Devices and biomaterials
Loveridge et al 1998	Policy planning	Widespread use for skin replacemt and/or repair of reliable products based on tissue engineering	21	Treatment and management of symptoms and disease	XII - skin	Regenerative medicine
Loveridge et al 1998	Policy planning	Practical availability for regular clinical use of functional human organ tissue for kidney, liver or pancreas	21	Treatment and management of symptoms and disease	Other - generic	Regenerative medicine
Loveridge et al 1998	Policy planning	Widespread use of non-surgical (eg. by	21	Health promotion and prevention of disease	XXI - screening, rehab	Drugs (not prophylactic vaccines)

For peer review only - http://bmjopen.bmj.com/site/about/guidelines.xhtml

Appendix1: List of identified healthcare technologies

		vaccination) single application contraception offering lifetime control				
Loveridge et al 1998	Policy planning	50% of contraception involving pharmacological or vaccine methods resides with men	21	Health promotion and prevention of disease	XXI - screening, rehab	Drugs (not prophylactic vaccines)
Loveridge et al 1998	Policy planning	50% of diagnostic and treatment decisions involve use of expert systems	21	Supporting patients receiving care (designed to be used by individuals)	Other - generic	IT systems, eHealth and mHealth
Loveridge et al 1998	Policy planning	20% of time spent by doctors in primary care screeing is replaced by patient interaction with experts systems	21	Supporting patients receiving care (designed to be used by individuals)	Other - generic	IT systems, eHealth and mHealth
Loveridge et al 1998	Policy planning	Practical use of remote electronic systems which substitute human labour in montoring the physical parameter of elderly and people with disability in the community	21	Supporting patients receiving care (designed to be used by individuals)	Other - generic	IT systems, eHealth and mHealth
Loveridge et al 1998	Policy planning	Widespread use of unified and standardised electronic data collection systems and data informats for medical records	21	Supporting the provision of care and/or increasing service efficiency	Other - generic	IT systems, eHealth and mHealth
Loveridge et al 1998	Policy planning	At least 50% of health care centres (hospitals, clinic,etc) are connected to national information	21	Supporting the provision of care and/or increasing service efficiency	Other - generic	IT systems, eHealth and mHealth

For peer review only - http://bmjopen.bmj.com/site/about/guidelines.xhtml

Appendix1: List of identified healthcare technologies

		electronic networks for patient records and information on diagnosis, treatment and outcome				
Loveridge et al 1998	Policy planning	Widespread use in health care of electronic information systems which span existing professional and organisational boundries (health, social service, housing etc)	21	Supporting the provision of care and/or increasing service efficiency	Other - generic	IT systems, eHealth and mHealth
Loveridge et al 1998	Policy planning	Improved understanding of the role of gene-environment interactions in causing common structural birth birth defects (e.g. cleft palate) permits the implementation of primary preventable programmes	21	Health promotion and prevention of disease	XXI - screening, rehab	Population programmes
Loveridge et al 1998	Policy planning	Understanding how environmental factors encountered in early life cause adult disease leads to national standards for diet and lifestyle in pre-natal and early life	21	Health promotion and prevention of disease	XXI - screening, rehab	Population programmes
Loveridge et al 1998	Policy planning	Widespread use of financial inducements by national and private health insurers or similar bodies to adopt	21	Health promotion and prevention of disease	Other - generic	Organisational programmes

For peer review only - http://bmjopen.bmj.com/site/about/guidelines.xhtml

Appendix1: List of identified healthcare technologies

		healthier lifestyle or diets				
Loveridge et al 1998	Policy planning	The causes of the rising prevalence of asthma are elucidated and action based on this knowledge leads to a 50% reduction in the incidence of severe disease	21	Health promotion and prevention of disease	X - respiratory	Population programmes
Operating theatre of the year 2010; Department of Trade and Industry	Policy planning	X-ray replaced by Magnetic Resonance Imaging (MRI)	14	Diagnosis and monitoring of disease	Other - generic	Diagnostic tests and imaging
Operating theatre of the year 2010; Department of Trade and Industry	Policy planning	operating theatre of 2010 will be built with an ultra-open MRI system with parallel ultrasound facilities and complete optical capability	14	Supporting the provision of care and/or increasing service efficiency	Other - generic	Medical equipment
Operating theatre of the year 2010; Department of Trade and Industry	Policy planning	Endoscopes will reduce in size as fibres, lenses and electronic sensors get smaller,	14	Supporting patients receiving care (designed to be used by individuals)	Other - generic	Devices and biomaterials
Operating theatre of the year 2010; Department of Trade and Industry	Policy planning	CCD image sensors have the ability to see in both ultra-violet and near infra-red.	14	Diagnosis and monitoring of disease	Other - generic	Diagnostic tests and imaging
Operating theatre of the year 2010; Department of Trade and Industry	Policy planning	New magnetic sensing technologies will detect the configuration of the endoscope and accessory devices inside the patient.	14	Supporting patients receiving care (designed to be used by individuals)	Other - generic	Devices and biomaterials

For peer review only - http://bmjopen.bmj.com/site/about/guidelines.xhtml

Appendix1: List of identified healthcare technologies

Operating theatre of the year 2010; Department of Trade and Industry	Policy planning	Auto focus and zoom optical systems will be interfaced with 30 to take the endoscope imaging capability beyond what is currently possible with human vision.	14	Diagnosis and monitoring of disease	Other - generic	Diagnostic tests and imaging
Operating theatre of the year 2010; Department of Trade and Industry	Policy planning	High resolution ultrasound in closer proximity to the tissue and structures to be studied	14	Diagnosis and monitoring of disease	Other - generic	Diagnostic tests and imaging
Operating theatre of the year 2010; Department of Trade and Industry	Policy planning	Laser induced fluorescence imaging systems for diagnosis of early malignant changes, monitoring and therapy will become available.	14	Diagnosis and monitoring of disease	II - cancers	Diagnostic tests and imaging
Operating theatre of the year 2010; Department of Trade and Industry	Policy planning	Near infra-red spectroscopy, for looking at tissue oxygenation state	14	Diagnosis and monitoring of disease	Other - generic	Diagnostic tests and imaging
Operating theatre of the year 2010; Department of Trade and Industry	Policy planning	Confocal, or other optical, ultrasonic or magnetic resonance microscopy, for on-line histology of bulk samples	14	Diagnosis and monitoring of disease	Other - generic	Diagnostic tests and imaging
Operating theatre of the year 2010; Department of Trade and Industry	Policy planning	Minimal Access Surgery is likely to account for as much as 75% of surgical procedures	14	Treatment and management of symptoms and disease	Other - generic	Therapeutic procedures
Operating theatre of the year 2010; Department of Trade and Industry	Policy planning	Robotic camera holders are likely to become routine in laparoscopic procedures.	14	Treatment and management of symptoms and disease	Other - generic	Medical equipment

For peer review only - http://bmjopen.bmj.com/site/about/guidelines.xhtml

Appendix1: List of identified healthcare technologies

Operating theatre of the year 2010; Department of Trade and Industry	Policy planning	The operating table's control system and the robotics system could become integrated in the future operating table.	14	Supporting the provision of care and/or increasing service efficiency	Other - generic	Medical equipment
Operating theatre of the year 2010; Department of Trade and Industry	Policy planning	Ability to ventilate all patients from neonates to adults	14	Treatment and management of symptoms and disease	X - respiratory	Non-surgical therapy
Operating theatre of the year 2010; Department of Trade and Industry	Policy planning	Control handsets will incorporate dot matrix displays which will give the operator information about the condition of the table, position of the top and warnings about possible hazards	14	Supporting the provision of care and/or increasing service efficiency	Other - generic	Medical equipment
Operating theatre of the year 2010; Department of Trade and Industry	Policy planning	The operating table may have a built-in patient temperature regulation system.	14	Supporting the provision of care and/or increasing service efficiency	Other - generic	Medical equipment
Operating theatre of the year 2010; Department of Trade and Industry	Policy planning	Connection between the Operating Room and the Anaesthetic machine in the Control Room will be via the Anaesthetist in the Operating Room.	14	Supporting the provision of care and/or increasing service efficiency	Other - generic	Medical equipment
Operating theatre of the year 2010; Department of Trade and Industry	Policy planning	The Anaesthetic machine will incorporate both inhalation agent vaporisation and multi-drug infusion systems These devices will be electronically integrated into the monitoring	14	Supporting the provision of care and/or increasing service efficiency	Other - generic	Medical equipment

For peer review only - http://bmjopen.bmj.com/site/about/guidelines.xhtml

Appendix1: List of identified healthcare technologies

		and information management and anaesthetic record keeping system so that vaporiser concentration settings and infusion rates will be continuously recorded				
Operating theatre of the year 2010; Department of Trade and Industry	Policy planning	Mechanical flow-meters will be replaced by electronic gas mixing systems	14	Supporting the provision of care and/or increasing service efficiency	Other - generic	Medical equipment
Operating theatre of the year 2010; Department of Trade and Industry	Policy planning	Patient monitoring will be very simple and intuitive probably touch screen and quite possibly voice activation.	14	Supporting patients receiving care (designed to be used by individuals)	Other - generic	IT systems, eHealth and mHealth
Operating theatre of the year 2010; Department of Trade and Industry	Policy planning	Monitors will also network into central areas outside the operating theatre so that, for example, anaesthetics being delivered by less experienced staff can be monitored remotely.	14	Supporting patients receiving care (designed to be used by individuals)	Other - generic	IT systems, eHealth and mHealth
Operating theatre of the year 2010; Department of Trade and Industry	Policy planning	The operation room will capture the data from one or perhaps two small 'wireless' sensors placed on the body that will provide pulse oxyimetry C02, ECG and other key parameter data and then transmit them to the monitor via radio or IR technology.	14	Supporting patients receiving care (designed to be used by individuals)	Other - generic	IT systems, eHealth and mHealth

For peer review only - http://bmjopen.bmj.com/site/about/guidelines.xhtml

Appendix1: List of identified healthcare technologies

Operating theatre of the year 2010; Department of Trade and Industry	Policy planning	Patient will be equipped with a belt (or other suitable garment) into which all monitoring and feed lines will be terminated, and which will include significant active capability. Drip and other lines will also be terminated in the belt	14	Supporting patients receiving care (designed to be used by individuals)	Other - generic	Devices and biomaterials
Operating theatre of the year 2010; Department of Trade and Industry	Policy planning	Patient can be "plugged" into electrical support, monitoring and stimulation as well as fluid and chemical agents using a single connector to facilitate transfer of patient from one ward to another or from the operation theatre	14	Supporting patients receiving care (designed to be used by individuals)	Other - generic	IT systems, eHealth and mHealth
Operating theatre of the year 2010; Department of Trade and Industry	Policy planning	Operation lamp will be replaced by a better light source	14	Supporting the provision of care and/or increasing service efficiency	Other - generic	Medical equipment
Operating theatre of the year 2010; Department of Trade and Industry	Policy planning	Automatic control of light pattern in the operation theatre	14	Supporting the provision of care and/or increasing service efficiency	Other - generic	Medical equipment
Operating theatre of the year 2010; Department of Trade and Industry	Policy planning	The operating lamp of the future is likely to use much more electronics to accurately control the voltage, dimming, focus, tracking and to limit maximum light intensity to	14	Supporting the provision of care and/or increasing service efficiency	Other - generic	Medical equipment

For peer review only - http://bmjopen.bmj.com/site/about/guidelines.xhtml

Appendix1: List of identified healthcare technologies

		safe levels.				
Operating theatre of the year 2010; Department of Trade and Industry	Policy planning	The operating lamps of the future will be sealed for easy disinfection	14	Supporting the provision of care and/or increasing service efficiency	Other - generic	Medical equipment
Operating theatre of the year 2010; Department of Trade and Industry	Policy planning	Telemedicine	14	Unknown	Other - generic	IT systems, eHealth and mHealth
Operating theatre of the year 2010; Department of Trade and Industry	Policy planning	Telemonitoring	14	Supporting the provision of care and/or increasing service efficiency	Other - generic	IT systems, eHealth and mHealth
Operating theatre of the year 2010; Department of Trade and Industry	Policy planning	Teleconsultation	14	Supporting the provision of care and/or increasing service efficiency	Other - generic	IT systems, eHealth and mHealth
Operating theatre of the year 2010; Department of Trade and Industry	Policy planning	Telesurgery for demonstartion or teaching	14	Supporting the provision of care and/or increasing service efficiency	Other - generic	IT systems, eHealth and mHealth
Operating theatre of the year 2010; Department of Trade and Industry	Policy planning	Minimal access surgery (MAS)	14	Treatment and management of symptoms and disease	Other - generic	Therapeutic procedures
Operating theatre of the year 2010; Department of Trade and Industry	Policy planning	Systems in which as much of the patient's own blood is recovered and recycled as possible.	14	Treatment and management of symptoms and disease	III - blood	Non-surgical therapy
Operating theatre of the year 2010; Department of Trade and Industry	Policy planning	Use of flexible endoscope technology to allow surgeons to see around and behind structures, giving the surgeon greater access and the patient a greater degree of safety.	14	Treatment and management of symptoms and disease	XI - GI tract, dental health	Devices and biomaterials

For peer review only - http://bmjopen.bmj.com/site/about/guidelines.xhtml

Appendix1: List of identified healthcare technologies

Operating theatre of the year 2010; Department of Trade and Industry	Policy planning	A move away from the use of X-rays and image intensification towards more mobile methods of MRI scanning.	14	Diagnosis and monitoring of disease	Other - generic	Diagnostic tests and imaging
Operating theatre of the year 2010; Department of Trade and Industry	Policy planning	Developments in the use of laser and particularly electrosurgery	14	Treatment and management of symptoms and disease	Other - generic	Therapeutic procedures
Operating theatre of the year 2010; Department of Trade and Industry	Policy planning	Use of robotics and head up display allowing surgeon sitting in a control room rather than being in the theatre with the patient	14	Supporting the provision of care and/or increasing service efficiency	Other - generic	Medical equipment
Operating theatre of the year 2010; Department of Trade and Industry	Policy planning	Coding will be used to associate patient, operating procedure (and so appropriate tools and drugs), support facilities and to generate automatic records of usage.	14	Supporting the provision of care and/or increasing service efficiency	Other - generic	IT systems, eHealth and mHealth
Operating theatre of the year 2010; Department of Trade and Industry	Policy planning	Tools and devices for a procedure will be located in a carousel analogous to that for a computer numerically controlled machine tool. This will allow the surgeon to present his tool holder to it, have the old tool extracted and a new one inserted.	14	Supporting the provision of care and/or increasing service efficiency	Other - generic	Medical equipment
Operating theatre of the year 2010; Department of Trade and Industry	Policy planning	Robots will be used as transport vehicles both within the operating room	14	Supporting the provision of care and/or increasing service efficiency	Other - generic	Medical equipment

For peer review only - http://bmjopen.bmj.com/site/about/guidelines.xhtml

Appendix1: List of identified healthcare technologies

		and for the surrounding support areas				
Operating theatre of the year 2010; Department of Trade and Industry	Policy planning	Robots will carry out very precise procedures controlled by the surgeon from a distance,	14	Supporting patients receiving care (designed to be used by individuals)	Other - generic	Medical equipment
Operating theatre of the year 2010; Department of Trade and Industry	Policy planning	Joint replacement be performed robotically	14	Treatment and management of symptoms and disease	XIII - musculoskeletal	Therapeutic procedures
Operating theatre of the year 2010; Department of Trade and Industry	Policy planning	The operation theatre may be controlled by a robot "software" box th at will respond to voice recognition of the surgeon and change factors such as the heating of the theatre, the lighting and position of the table	14	Supporting the provision of care and/or increasing service efficiency	Other - generic	Medical equipment
Operating theatre of the year 2010; Department of Trade and Industry	Policy planning	A robot would be able to transfer sterile instrument trays from a central sterile store to the surgeon at the operating table.	14	Supporting the provision of care and/or increasing service efficiency	Other - generic	Medical equipment
Operating theatre of the year 2010; Department of Trade and Industry	Policy planning	Transport Robots could be used in the supply and disposal functions outside the operating room within the operating suite	14	Supporting the provision of care and/or increasing service efficiency	Other - generic	Medical equipment
Operating theatre of the year 2010; Department of Trade and Industry	Policy planning	Robots will be used in moving patients from ward to theatre areas (to replace the use of porters)	14	Supporting the provision of care and/or increasing service efficiency	Other - generic	Medical equipment

For peer review only

For peer review only - http://bmjopen.bmj.com/site/about/guidelines.xhtml

Appendix1: List of identified healthcare technologies

Operating theatre of the year 2010; Department of Trade and Industry	Policy planning	Using dedicated robot trucks similar to the supply trucks, waste could be collected on a regular basis from each theatre and taken to an automatic local waste reduction/ disposal facility adjacent to the theatre suite.	14	Supporting the provision of care and/or increasing service efficiency	Other - generic	Medical equipment
Operating theatre of the year 2010; Department of Trade and Industry	Policy planning	Disposal of hospital material could be performed on site with the use of machines such as "needle eaters"	14	Supporting the provision of care and/or increasing service efficiency	Other - generic	Medical equipment
Operating theatre of the year 2010; Department of Trade and Industry	Policy planning	Bulky X-Ray machines will probably be a thing of the past and replaced by computerised radiology	14	Diagnosis and monitoring of disease	Other - generic	Medical equipment
Operating theatre of the year 2010; Department of Trade and Industry	Policy planning	A central control room, with visual links to each theatre served, could house all of the equipment.	14	Supporting the provision of care and/or increasing service efficiency	Other - generic	Medical equipment
Operating theatre of the year 2010; Department of Trade and Industry	Policy planning	All theatres could be designed with ultra-clean ventilation systems or an adapted containment system to reduce the risk of the spread of infection	14	Health promotion and prevention of disease	I - infections	Medical equipment
Operating theatre of the year 2010; Department of Trade and Industry	Policy planning	Patients identification bracelets could be coded and bar checked as they moved through	14	Supporting patients receiving care (designed to be used by individuals)	Other - generic	Medical equipment

For peer review only - http://bmjopen.bmj.com/site/about/guidelines.xhtml

Appendix1: List of identified healthcare technologies

		the system so that their progress could be monitored at all times				
Operating theatre of the year 2010; Department of Trade and Industry	Policy planning	Automated trolleys and doors that can be electronically controlled by robots	14	Supporting the provision of care and/or increasing service efficiency	Other - generic	Medical equipment
UK technology and innovation futures for the 2020s, 2010	Policy planning	Further development of 3D imaging techniques.	10	Diagnosis and monitoring of disease	Other - generic	Diagnostic tests and imaging
UK technology and innovation futures for the 2020s, 2010	Policy planning	Fluorescenceassisted Resection and Exploration (FLARE) technologies, which combine both visible light imaging with near-infra red imaging of fluorescent biomarkers, can detect particular pathologies associated with tumours without the need for biopsy	10	Diagnosis and monitoring of disease	Other - generic	Diagnostic tests and imaging
UK technology and innovation futures for the 2020s, 2010	Policy planning	Colour X-ray or TEDDI imaging can be used to biopsy tissues by utilising scattered X-rays to build a spatially-resolved picture that can distinguish between different materials.	10	Diagnosis and monitoring of disease	Other - generic	Diagnostic tests and imaging
UK technology and innovation futures for the 2020s, 2010	Policy planning	In vivo neuroimaging, both at the tissue and cellular level	10	Diagnosis and monitoring of disease	Other - generic	Diagnostic tests and imaging
UK technology and innovation futures for the	Policy planning	Brain imaging techniques such as functional	10	Diagnosis and monitoring of disease	Other - generic	Diagnostic tests and imaging

For peer review only - http://bmjopen.bmj.com/site/about/guidelines.xhtml

Appendix1: List of identified healthcare technologies

2020s, 2010		magnetic resonance imaging (fMRI)				
UK technology and innovation futures for the 2020s, 2010	Policy planning	Lab-on-a-Chip (LOC) devices integrate and scale-down biomedical and other analytical laboratory functions and processes to a miniaturised chip format.	10	Diagnosis and monitoring of disease	Other - generic	Diagnostic tests and imaging
UK technology and innovation futures for the 2020s, 2010	Policy planning	Nucleic acid probes and diagnostic microarray systems can detect the nucleic acid sequence or sequences that identify a specific infectious disease agent, human genetic disease or individual (DNA profiling).	10	Diagnosis and monitoring of disease	Other - generic	Diagnostic tests and imaging
UK technology and innovation futures for the 2020s, 2010	Policy planning	A new focus on single-stranded DNA, micro RNA (miRNA) and double-stranded RNA (dsRNA) technologies as therapeutics may lead to clinically-approved interventions for gene therapy and treatment of medical disease.	10	Treatment and management of symptoms and disease	Other - generic	Regenerative medicine
UK technology and innovation futures for the 2020s, 2010	Policy planning	RNA interference (RNAi) may lead to new treatments.	10	Treatment and management of symptoms and disease	Other - generic	Regenerative medicine
UK technology and innovation futures for the 2020s, 2010	Policy planning	Viruses, bacteria or yeasts may be able to act as carriers for new sequences of DNA	10	Treatment and management of symptoms and disease	Other - generic	Regenerative medicine

For peer review only - http://bmjopen.bmj.com/site/about/guidelines.xhtml

Appendix1: List of identified healthcare technologies

UK technology and innovation futures for the 2020s, 2010	Policy planning	Increased understanding of individual sensitivities to drugs	10	Diagnosis and monitoring of disease	III - blood	Drugs (not prophylactic vaccines)
UK technology and innovation futures for the 2020s, 2010	Policy planning	DNA vaccines for AIDS, malaria, hepatitis B and certain cancers may be available within 10 years	10	Health promotion and prevention of disease	Other - generic	Prophylactic vaccines
UK technology and innovation futures for the 2020s, 2010	Policy planning	New treatments for for neurodegenerative conditions such as dementia.	10	Treatment and management of symptoms and disease	VI - nervous	Unknown
UK technology and innovation futures for the 2020s, 2010	Policy planning	'Cognition enhancers'; substances that enhance the performance of our brain in specific ways, such as improving short-term memory and increasing our speed of thought enhancement through lifestyle drugs may will become the norm.	10	Treatment and management of symptoms and disease	VI - nervous	Drugs (not prophylactic vaccines)
UK technology and innovation futures for the 2020s, 2010	Policy planning	'Emoticeuticals', drugs that act by changing hormone levels in the body, may mediate our responses in our private lives and work.	10	Treatment and management of symptoms and disease	V - mental health	Drugs (not prophylactic vaccines)
UK technology and innovation futures for the 2020s, 2010	Policy planning	Sensoceuticals' might enhance pleasure by restoring or accentuating the senses.	10	Treatment and management of symptoms and disease	Other - generic	Drugs (not prophylactic vaccines)
UK technology and innovation futures for the 2020s, 2010	Policy planning	The advent of a drug that mimics the positive effects of alcohol	10	Health promotion and prevention of disease	V - mental health	Drugs (not prophylactic vaccines)

For peer review only - http://bmjopen.bmj.com/site/about/guidelines.xhtml

Appendix1: List of identified healthcare technologies

		with none of its drawbacks.				
UK technology and innovation futures for the 2020s, 2010	Policy planning	Stem cells replacing malfunctioning liver cells or even growing entire livers to replace diseased ones	10	Treatment and management of symptoms and disease	XI - GI tract, dental health	Regenerative medicine
UK technology and innovation futures for the 2020s, 2010	Policy planning	Stem cells giving new neurons to those with brain damage	10	Treatment and management of symptoms and disease	VI - nervous	Regenerative medicine
UK technology and innovation futures for the 2020s, 2010	Policy planning	Stem cells giving new heart cells to those with heart disease	10	Treatment and management of symptoms and disease	IX - circulation	Regenerative medicine
UK technology and innovation futures for the 2020s, 2010	Policy planning	Stem cells giving therapies for Parkinson's and Alzheimer's diseases	10	Treatment and management of symptoms and disease	VI - nervous	Regenerative medicine
UK technology and innovation futures for the 2020s, 2010	Policy planning	Stem cells giving therapies for spinal cord injury, stroke, cancer, burns, cardiovascular disease, diabetes, osteoarthritis and rheumatoid arthritis; as well as furnishing an endless supply of red blood cells for transfusions.	10	Treatment and management of symptoms and disease	Other - generic	Regenerative medicine
UK technology and innovation futures for the 2020s, 2010	Policy planning	Development of a cheap anti-malarial drug.	10	Treatment and management of symptoms and disease	I - infections	Drugs (not prophylactic vaccines)
UK technology and innovation futures for the 2020s, 2010	Policy planning	Programming a bacterium or virus that can identify malignant cells and can deliver a therapeutic agent could have major benefits for treating cancer and similar illnesses	10	Treatment and management of symptoms and disease	II - cancers	Regenerative medicine

For peer review only - http://bmjopen.bmj.com/site/about/guidelines.xhtml

Appendix1: List of identified healthcare technologies

UK technology and innovation futures for the 2020s, 2010	Policy planning	Stratified medicine: it will become increasingly possible to analyse an individual for certain biomarkers to determine the best course of treatment	10	Diagnosis and monitoring of disease	Other - generic	Diagnostic tests and imaging
UK technology and innovation futures for the 2020s, 2010	Policy planning	Personalised medicine	10	Treatment and management of symptoms and disease	Other - generic	Drugs (not prophylactic vaccines)
UK technology and innovation futures for the 2020s, 2010	Policy planning	In-situ tissue regeneration: Material is implanted directly in the body and regenerates the tissue over time as the material degrades.	10	Treatment and management of symptoms and disease	Other - generic	Regenerative medicine
UK technology and innovation futures for the 2020s, 2010	Policy planning	Tissue engineering: Material can act as temporary scaffold for cell culture and tissue growth in vitro	10	Treatment and management of symptoms and disease	Other - generic	Regenerative medicine
UK technology and innovation futures for the 2020s, 2010	Policy planning	Micro-masonry and 3D organ printing techniques	10	Treatment and management of symptoms and disease	Other - generic	Regenerative medicine
UK technology and innovation futures for the 2020s, 2010	Policy planning	Brain Computer Interface	10	Rehabilitation	Other - generic	Devices and biomaterials
UK technology and innovation futures for the 2020s, 2010	Policy planning	Neuroprosthetics: the connection can occur between any part of the nervous system (such as peripheral nerves) and a device.	10	Rehabilitation	VI - nervous	Devices and biomaterials

For peer review only

Appendix1: List of identified healthcare technologies

UK technology and innovation futures for the 2020s, 2010	Policy planning	Magnetoencephalography (MEG): maps brain activity by recording the magnetic fields produced by electrical currents occurring in the brain	10	Diagnosis and monitoring of disease	VI - nervous	Diagnostic tests and imaging
UK technology and innovation futures for the 2020s, 2010	Policy planning	functional magnetic imaging (fMRI) – involves measuring changes in blood flow related to neural activity in the brain.	10	Diagnosis and monitoring of disease	VI - nervous	Diagnostic tests and imaging
UK technology and innovation futures for the 2020s, 2010	Policy planning	functional Near-Infrared Spectroscopy (fNIRS) for optical topography	10	Diagnosis and monitoring of disease	Other - generic	Diagnostic tests and imaging
UK technology and innovation futures for the 2020s, 2010	Policy planning	Electrocorticography (EoCG), measure the electrical activity of the brain using electrodes which are implanted inside the skull, but outside the brain tissue.	10	Diagnosis and monitoring of disease	VI - nervous	Diagnostic tests and imaging
UK technology and innovation futures for the 2020s, 2010	Policy planning	Implanted brain chips for controlling computers	10	Rehabilitation	XXI - screening, rehab	Devices and biomaterials
UK technology and innovation futures for the 2020s, 2010	Policy planning	Neural prosthetics replace lost limbs	5	Rehabilitation	XXI - screening, rehab	Devices and biomaterials
UK technology and innovation futures for the 2020s, 2010	Policy planning	Thought-controlled robots for personal healthcare	10	Supporting patients receiving care (designed to be used by individuals)	Other - generic	Medical equipment
UK technology and innovation futures for the 2020s, 2010	Policy planning	Thought-controlled wheelchairs	10	Rehabilitation	XXI - screening, rehab	Medical equipment

For peer review only - http://bmjopen.bmj.com/site/about/guidelines.xhtml

Appendix1: List of identified healthcare technologies

UK technology and innovation futures for the 2020s, 2010	Policy planning	ehealth: Examples include health information networks, electronic health records, tele-medicine services, personal wearable and portable mobile devices, health portals and other tools that assist in health monitoring, diagnosis and treatment.	10	Supporting the provision of care and/or increasing service efficiency	Other - generic	IT systems, eHealth and mHealth
UK technology and innovation futures for the 2020s, 2010	Policy planning	Lab-on-a-chip technologies could also bring diagnostics down to a personal scale, where patients can test themselves and transmit data electronically for analysis and treatment.	10	Supporting patients receiving care (designed to be used by individuals)	Other - generic	IT systems, eHealth and mHealth
UK technology and innovation futures for the 2020s, 2010	Policy planning	Tailored Medicine	10	Treatment and management of symptoms and disease	Other - generic	Drugs (not prophylactic vaccines)
Technology foresight towards 2020_China	Policy planning	The anti-viral medicine with highly efficiency applies to clinical medicine widely	17	Treatment and management of symptoms and disease	I - infections	Drugs (not prophylactic vaccines)
Technology foresight towards 2020_China	Policy planning	standardized safe evaluation system which include the technology for monitoring and examination of trans-gene biology will be established;	17	Supporting the provision of care and/or increasing service efficiency	Other - generic	Organisational programmes

For peer review only - http://bmjopen.bmj.com/site/about/guidelines.xhtml

BMJ Open

Past speculations of future health technologies: a description of technologies predicted in 15 forecasting studies 1986-2010

Journal:	*BMJ Open*
Manuscript ID	bmjopen-2017-016206.R1
Article Type:	Research
Date Submitted by the Author:	04-Apr-2017
Complete List of Authors:	Doos, Lucy; University of Birmingham, Institute of Applied Health Research, NIHR Horizon Scanning Research and Intelligence Centre<br>Packer, Claire; NIHR Horizon Scanning Research and Intelligence Centre, Insitute of Applied Health Research, University of Birmingham<br>Ward, Derek; University of Birmingham, NIHR Horizon Scanning Research and Intelligence Centre, Institute of Applied Health Research<br>Simpson, Sue; University of Birmingham, NIHR Horizon Scanning Research and Intelligence Centre, Institute of Applied Health Research<br>Stevens, Andrew; University of Birmingham, Institute of Applied Health Research
**Primary Subject Heading**:	Public health
Secondary Subject Heading:	Health policy
Keywords:	Forecasting, prediction, health technology, innovation, healthcare

SCHOLARONE™
Manuscripts

Past speculations of future health technologies: a description of technologies predicted in 15 forecasting studies 1986-2010

Lucy Doos[1], Claire Packer[1], Derek Ward[1], Sue Simpson[1], Andrew Stevens[2]

[1]NIHR Horizon Scanning Research and Intelligence Centre, Institute of Applied Health Research, University of Birmingham

[2] Institute of Applied Health Research, University of Birmingham

Corresponding author:

Dr Lucy Doos

Institute of Applied Health Research,

Public Health Building,

University of Birmingham, Edgbaston,

Birmingham, B15 2TT, UK.

Email: l.doos@bham.ac.uk

Phone: (+44)121 414 8594

Keywords: Forecasting, prediction, health technology, innovation, healthcare

Word count: 3,780

For peer review only

ABSTRACT

Objective: To describe and classify health technologies predicted in forecasting studies.

Design and methods: Description of health technologies predicted in 15 forecasting studies published between 1986 and 2010 that were identified in a previous systematic review. Health technologies are classified according to their type, purpose and clinical use; relating these to the original purpose and timing of the forecasting studies.

Data sources: All health-related technologies predicted in 15 forecasting studies identified in a previously published systematic review.

Main outcome measure: Outcomes related to i) each forecasting study including country, year, intention and forecasting methods used and ii) the predicted technologies including technology type, purpose, targeted clinical area and forecast timeframe.

Results: Of the 896 identified health-related technologies, 685 (76.5%) were health technologies with an explicit or implied health application and included in our study. Of these, 19.1% were diagnostic or imaging tests, 14.3% devices or biomaterials, 12.6% information technology systems, eHealth or mHealth, and 12% drugs. The majority of the technologies were intended to treat or manage disease (38.1%) or diagnose or monitor disease (26.1%). The most frequent targeted clinical areas were infectious diseases followed by cancer, circulatory and nervous system disorders. The most frequent technology types were for: infectious diseases - prophylactic vaccines (45.8%); cancer - drugs (40%); circulatory disease - devices and biomaterials (26.3%), and diseases of the nervous system - equally devices and biomaterials (25%) and regenerative medicine (25%). The mean timeframe for forecasting was 11.6 years (range 0-33 years, median=10, SD= 6.6). The forecasting timeframe significantly differed by technology type ($p=0.002$), the intent of the forecasting group ($p<0.001$), and the methods used ($p<001$).

For peer review only

For peer review only - http://bmjopen.bmj.com/site/about/guidelines.xhtml

Conclusion: Description and classification of predicted health-related technologies is crucial in preparing healthcare systems for adopting new innovations. Further work is needed to test the accuracy of predictions made.

Strengths and limitations of the study

- The study is a comprehensive description and classification of health-related technologies predicted in 15 forecasting studies identified in a previously published systematic review.
- The study summarises the forecast technologies by type e.g. medical devices, diagnostic tests or drugs; by clinical purpose e.g. prevention, diagnosis or treatment and by targeted clinical area by ICD10 chapter; and relates this to the forecasting purpose and timeframe.
- The analysis and the health-related technologies identified are constrained by the methods used in the forecasting studies for example by the areas of interest of the experts involved.
- Although the study highlights the identified technologies, the retrospective accuracy of the studies remains to be researched.

For peer review only

For peer review only - http://bmjopen.bmj.com/site/about/guidelines.xhtml

INTRODUCTION

People are in general living longer and surviving previously life threatening illnesses. A proportion of this effect is attributed to the use of novel health interventions and technologies, such as new vaccines to prevent disease; new diagnostic tests to diagnose diseases earlier and with improved accuracy; and new drugs, devices and surgical procedures to treat diseases more effectively. In addition, new health technologies may also be used to enhance function and improve quality of life, such as prostheses, sensory aids and cosmetic enhancements. Technological advances and innovation are leading to new health interventions becoming available to healthcare markets at an increasing speed; these often cost more than current alternatives and significantly affect the cost of healthcare services and delivery.[1,2] To ensure that patients can access the most effective and cost effective interventions, and because many health systems work within constrained budgets, healthcare systems need to be prepared to respond to new developments. A crucial step in such preparation is the accurate identification and forecasting of likely significant technological healthcare developments.[3,4]

Forecasting is widely used for planning and strategic decision making around industrial and economic development at organisational, regional and national levels.[5] Within healthcare, forecasting can be used to build strategies for supporting innovators and companies to develop new interventions; to plan future research programmes and delivery of healthcare; and to prepare health services and personnel to respond to technological developments of benefit to patients and service provision. Health technology forecasting uses various methods such as Delphi surveys and reviews of the literature[6,7,8] to identify emerging health technologies that are intended to address important unmet healthcare needs or that need additional evaluation, and to analyse their potential impact on patients and health systems.[4]

To get the maximum benefit from forecasting, it is important that the methods used to identify emerging interventions and technologies are appropriate for the aims and timeframe of the forecasting exercise and also as accurate as possible.

For peer review only - http://bmjopen.bmj.com/site/about/guidelines.xhtml

In a recent systematic review[9] we found 15 forecasting studies with 3 to 20 year timeframes that identified emerging health-related topics and technologies predicted between 1986 and 2010 from 12 high income countries, including six from the United Kingdom (UK). Identifying and classifying the predicted new technologies into meaningful groupings is important for healthcare system preparedness, allowing planned assessment and adoption as appropriate. Here we describe and classify the health technologies predicted in the forecasting studies according to their type, purpose and clinical use. We relate these to the forecast time period and develop an overview of the technological and clinical frontiers of innovation in health and healthcare provision.

METHODS

See table 1 for a full list of the 15 studies identified in our prior systematic review. We extracted all forecast topics from the 15 forecasting studies using a standardised set of data for each of these predicted topics and on the source study. Data related to the forecast topics included the name of the topic as written in the forecasting reports and papers, and the predicted forecast timeframe. Data related to the study included the country or region of the forecast, year the study was undertaken, its intention and remit, the study forecasting timeframe, and the number and type of forecasting methods used.

Identification and inclusion of health technologies

For final inclusion and analysis, topics had to be health technologies as defined by the National Institute of Health Research (NIHR) Health Technology Assessment (HTA) programme[10]: "*any method used to promote health; prevent and treat disease; and improve rehabilitation or long-term care. They are not confined to new drugs and include any intervention used in the treatment, prevention or diagnosis of disease*". We also included topics that related to the recognition of, or change in, health states or emotions, or health-related behaviours. All topics needed to have an explicit or implied health-related application or identified patient group for final inclusion. Topics were excluded if they related exclusively to foods, plants, animals, insects, new sources of fuel or energy, environmental contamination, legislation or health insurance.

For peer review only - http://bmjopen.bmj.com/site/about/guidelines.xhtml

For peer review only

LD read all the included studies and manually extracted all technologies mentioned in the forecast from the published text and related tables. Two authors (CP and LD) independently scrutinised each identified topic and applied the inclusion and exclusion criteria. Where there was insufficient information on a topic to make the inclusion/exclusion decision, we undertook brief and very limited searches on the Internet to find additional information.

All topics classed as a health technology with an explicit or implied health-related application, were subsequently coded based on their technology type, intended purpose, and targeted clinical use:

Technology type: we used broad categories: assistive devices and rehabilitation aids; devices and biomaterials; diagnostic tests and imaging; drugs; information technology (IT) systems, electronic health systems (eHealth) and mobile health (mHealth); medical equipment; non-surgical therapy; organisational programmes; prophylactic vaccines; population programmes; regenerative medicine approaches; and therapeutic procedures.

Technology purpose: we used categories that relate to the intended point on the patient pathway: health promotion and the prevention of disease; diagnosis and monitoring of disease; treatment and management of symptoms and disease; and rehabilitation. We supplemented these with two groupings that categorised technologies that support the provision of care i) supporting patients receiving care and designed to be used by individuals (patients, caregivers or healthcare professionals) and ii) supporting the provision of care and/or increasing service efficiency.

Targeted clinical area (specialty): we used the international statistical classification of diseases and related health problems 10th revision (ICD10)[11] chapter headings relating to diseases and other morbid conditions (chapters I-XVII); symptoms, signs and abnormal findings (chapter XVIII); injuries, poisoning and other consequences of external causes (chapter XIX); external causes of diseases and morbid conditions chapter XX); and factors influencing health status and contact with health services for people not currently sick (chapter XXI). Chapter XXI includes contraception,

For peer review only - http://bmjopen.bmj.com/site/about/guidelines.xhtml

technologies used in population screening, health promotion and disease prevention, and general rehabilitation.

All topics with disputed inclusion, exclusion or categorisation were initially discussed between two authors (LD and CP) and disagreements resolved where possible. Where disagreements remained, topics were discussed with the other authors as a group and the final categorisation agreed through consensus.

Technology forecast timeframe: for each health-related technology forecast we calculated the difference in years between each technology's predicted year of impact as identified in the original study, and the year when the prediction was made, i.e. the year of the original study. Technology forecasts were classified as short term if their forecasting timeframe was less than 3 years, short to medium term if it was 3-10 years, medium to long term if it was 11-20 years and long term for those with timeframes of more than 20 years. Although the original systematic review excluded forecasting studies which exclusively adopted a short time frame of less than 3 years, some studies with longer time frames also provided predictions with shorter time frames, and these were included in the analysis.

Data analysis

We analysed the data using IBM SPSS statistics (version 22) for Windows. We present descriptive analyses as means and standard deviations (SD) for normally distributed continuous variables. Statistically significant differences were determined using ANOVA for continuous normally distributed data and X^2 for dichotomous variables.

RESULTS

We identified 896 predicted topics from the 15 forecasting studies. Of these, we judged that 685 (76.5%) were health-related technologies with an explicit or implied health-related application. The most frequently excluded topics were those that described advances in the underpinning scientific knowledge (8.7%), such as determination of whole human DNA base sequence and identifying genetic links to

For peer review only

diseases. A full list of the included health-related technologies is attached as an online appendix.

Of the 685 health-related technologies, 52.3% were forecast from 6 studies by governmental organisations (such as the UK Department of Trade and Industry[12]), 21.9% by commercial/consultancy organisations (such as British Telecommunications plc[13]), 14.5% by policy planning groups (such as Loveridge et al 1995[6]), 8.9% by research groups (such as those by Stevens et al 1997[7]), and 2.5% by non-profit organisations (such as Institute of the Future[14]). Looking at the purpose of forecasting, our data showed that two thirds (68%) of the health technologies were forecast for policy planning purposes, 18.1% for research purposes and 13.9% for commercial purposes.

Nearly half (48.9%) of the identified technologies were from 6 UK-based studies and reports, 15.0% were from Japan, 11.7% from the Netherlands, 11.4% from the United States of America (USA) and 8.0% from Canada. The remainder were from a homogenous group of developing countries and South Africa (3.8%), other European countries (0.9%) and China (0.3%).

Technology type

Of the 685 health technologies forecast, 19.1% were diagnostic tests and imaging technologies, such as advanced ultrasound imaging systems and molecular diagnosis; 14.3% were devices and biomaterials, such as tissue engineered devices and drug impregnated devices; 12.6% were IT systems, eHealth and mHealth, such as electronic prescriptions and telemedicine; 12.0% were drugs, such as new anaesthetic vapours and electronically activated drugs; and 11.7% were regenerative medicine approaches, such as gene therapy for diabetes and widespread use of gene therapy for familial hypercholesterolemia (Table 2 and online appendix).Drug technologies were more commonly forecast in studies carried out by researchers (36.1%) than other groups, while technologies for diagnostic tests and imaging were more commonly forecast in studies by governmental agencies than other groups (Table 3). Regenerative medicine approaches were the most commonly forecast technologies (23.2%) by studies with a commercial intent while diagnostic tests and imaging were the most commonly forecast technologies by

For peer review only - http://bmjopen.bmj.com/site/about/guidelines.xhtml

studies with policy planning and research intentions (20.6% and 23.4% respectively) ($p<0.001$).

Technology purpose

Of the 685 health technologies included in the forecasting reports, 38.1% were for the treatment and management of symptoms and disease, such as development of effective treatment for amyotrophic lateral sclerosis and the practical use of gene therapy for genetic disorders; 21.6% for the diagnosis and monitoring of disease, for example a diagnostic bio-chip for cancer and the further development of 3D imaging techniques; 13% for health promotion and the prevention of disease, such as the first effective vaccine of HIV and DNA vaccines for AIDS, malaria, hepatitis B and certain cancers; 10.2% for supporting the provision of care, for example tele-monitoring and teleconsultation; 8.0% for supporting patients receiving care, such as smart pill bottles to remotely monitor medication use and thought-controlled robots for personal healthcare; and 4.7% for rehabilitation, for example artificial legs and robotic prosthetics.

Technologies for treatment and disease management were the most common regardless of the intention of the study: 35.8% among studies with a commercial intent, 35.8% for policy planning and 48.4% for research. Technologies for treatment and disease management were also the most common technologies forecast by all the various groups (60.7% among researchers, 39.9% among governmental organisations and 35.8% among commercial organisations).

Targeted clinical area

Using the ICD10 codes to define the targeted clinical area, just over half of the health technologies (50.4%) were for diseases and conditions that were not specified or that could not be coded or that crossed multiple specialities. Of the 340 health technologies that could be coded to a specific ICD10 chapter, the top five targeted clinical areas were: 1) infectious and parasitic diseases ICD-I (14.1%), such as development of vaccines for AIDS and vaccines for malaria, 2) neoplasms ICD-II (11.8%), such as expanding metal stents for oesophageal cancer and diagnostic bio-chip for cancer, 3) diseases of the circulatory system ICD-IX (11.2%), such as artificial muscles in replace of heart transplants and implantable vascular stents, 4)

For peer review only - http://bmjopen.bmj.com/site/about/guidelines.xhtml

diseases of the nervous system ICD-VI (10.6%), such as artificial brain cells and artificial peripheral nerves and 5) factors influencing health status and contact with health services ICD-XXI (10.3%), such as a personal wearable health monitor and extension of average lifespan to over 100.

Congenital malformations, deformations and chromosomal abnormalities ICD-XVII and symptoms, signs and abnormal clinical and laboratory findings, not elsewhere classified ICD- XVIII had the fewest forecast technologies (0.3% each).

Table 4 shows the technology types and purposes for the forecast technologies within the five most frequent targeted clinical areas. The most frequent technology type forecast for infectious and parasitic diseases were prophylactic vaccines (45.8%); for cancer were drugs (40%); for diseases of the circulatory system were devices and biomaterials (26.3%); for diseases of the nervous system were devices and biomaterials (25%) and regenerative medicine (25%); and for factors influencing health status and contact with health services including screening and rehabilitation, devices and biomaterials (31.4%). The most frequent technology purpose was health promotion and disease prevention for both infectious and parasitic diseases (ICD-I) (60.4%) and factors influencing health status and contact with health services including screening and rehabilitation (ICD-XXI) (51.4%). Technologies for treatment and disease management were most common for cancer (55.0%), diseases of the circulatory system (65.8%) and nervous system disorders (61.1%).

Technology forecast timeframe

Data was available to calculate the prediction timeframe for 607 of the identified health technologies. The mean timeframe for forecasting predictions was 11.6 years (range 0-33 years, median=10, SD=6.6). There was a significant difference in the mean prediction timeframe by technology type (p=0.002), with non-surgical therapies having the shortest mean forecast timeframe (9.07 years) and population health programmes having the longest mean forecast (14.53 years) (Table 5). There were no statistically significant differences in the mean duration of the technology prediction timeframe by the technology purpose and we were not able to detect any particular trend in the technology types or purposes of forecast over time.

For peer review only - http://bmjopen.bmj.com/site/about/guidelines.xhtml

There was a statistically significant difference in the forecast timeframe by the type of group undertaking the forecasting ($p<0.001$): 58% of the long-term forecasts (>20 years) were made by policy planning groups and 51% of short-term forecasts (<3 years) were by research groups.

Technologies forecast by studies with research intentions had the shortest mean forecast timeframe (4.49 years) while those for policy making had the longest timeframe (14.16 years) ($p<0.001$). Similarly, technologies forecast by researchers had significantly shorter mean timeframes (3.56 years) compared to those made by governmental agencies which had the longest mean forecast timeframes (13.42 years) ($P<0.001$).

There was a statistically significant difference in the mean forecast timeframe when considering the forecasting method ($p<0.001$) (Figure 1). Technologies forecast using methods that included creativity-based methods which require brainstorming, such as scenario building, had a significantly longer forecasting timeframe (13.5 years) compared to those did not include creativity-based methods such as literature reviews (9.7 years) ($p<0.001$). On the other hand technologies forecast using methods that included evidence-based methods such as literature reviews and evidence from health technology assessment (HTA) organisations, had significantly shorter forecasting timeframes (8.2 years) than those that did not include evidence-based methods (10.8 years) ($p<0.001$).

DISCUSSION

Being aware of forthcoming developments in healthcare and preparing for the future is essential for health policy makers, whether developing healthcare policy, directing future research or commissioning health services. We found that more than two thirds of the identified technologies were forecast for policy planning purposes and over half were forecast by governmental organisations, and that the majority of long-term forecasts were made by policy planning groups as opposed to research groups. This finding supports the premise that the majority of forecasting is carried out for service preparedness and long-term planning, and strategic decision making.[5]

For peer review only - http://bmjopen.bmj.com/site/about/guidelines.xhtml

Our study identified and classified a number of important health-related technologies, which health services may have already had to manage or will have to do so in the future. Our finding that nearly 60% of the technologies were either for the treatment and management of symptoms and disease or for the diagnosis and monitoring of disease can perhaps be explained by the methods used for forecasting. Many of the studies involved soliciting the opinion of experts and, although it is not possible to be certain about the areas of interest of those involved, their expertise may have made them more likely to focus on diagnosis and treatment than, for example, organisational programmes or rehabilitation. Regenerative medicine approaches were the most commonly forecast technologies by studies with a commercial intent. In contrast, drugs and related technologies (but not prophylactic vaccines) were the most common forecasts made by researchers.

The four clinical areas with the greatest number of forecast health technologies: infectious diseases, cancer, and circulatory and nervous system diseases and disorders, include many of the major causes of death and disability in countries today. Again we are not able to determine if this merely reflects the interests of the groups carrying out the forecasting and their areas of expertise or whether it truly represents the technology innovation frontier. It is understandable that the forecasts for infectious disease included many prophylactic vaccines as these have proven to be a cost effective preventive strategy for their prevention.[15]This was also supported by our finding that forecasts of vaccine related technologies were only made by governmental agencies and policy research groups. In cancer, forecasting included both drugs and diagnostic tests and imaging, perhaps reflecting increasing targeting in novel cancer therapies.[16,17] Several forecasts of regenerative medicine approaches for nervous system diseases and disorders is also interesting, with many of these diseases not currently curable or effectively treated.[18,19]

Our finding that forecasts which included creativity-based methods such as scenario building and analysis had, on the whole, longer forecast timeframes than studies that included evidence-based methods may have a rationale. Evidence-based methods require there to be research evidence on technologies which will therefore probably be nearer to any predicted impact. In contrast, creativity-based methods require experts to think more widely about what the future could be, and are not therefore

For peer review only - http://bmjopen.bmj.com/site/about/guidelines.xhtml

constrained by what is actually being currently researched and published. This may also explain our finding that just over half of the short-term forecasts were made by research groups who may be more likely to use published evidence to inform their deliberations.

We believe that this is the first study to comprehensively identify and summarise health-related technologies predicted in past forecasting projects and to categorise and describe them. Although as many predicted topics as possible were included, we did exclude a substantial number. This was often because we could not envisage a future health application or because there was insufficient information to make a judgement (and many topics had very sparse titles or descriptive information). By excluding topics in this way, we may have excluded some that would have had an explicit health application within a reasonable timeframe. In addition, it is likely that we were unable to identify a number of relevant forecasts made by commercial organisations, which will have been conducted and disseminated in confidence.

Our description and classification of predicted health-related technologies from prior forecasting studies provides an overview of the technological and clinical frontiers of innovation in health and health care provision. To complete our evaluation of the accuracy of previous forecasting predictions and the forecasting methods used, the accuracy of the predictions made should also be assessed. This may be difficult across all technology types and clinical areas, but possible by limiting the evaluation to specific clinical areas and/or technology types or to those topics with more obvious health applications and timeframes that have already occurred.

CONTRIBUTERS

All authors have directly participated in the planning and execution of the study. LD extracted the study data. LD and CP independently reviewed the technologies to be included. LD and CP drafted the paper and DW, SS and AS critically revised the manuscript. All authors agreed the final version of the paper. All authors had full access to all of the data.

For peer review only - http://bmjopen.bmj.com/site/about/guidelines.xhtml

DISCLAIMER

This report presents independent research funded by the National Institute for Health Research (NIHR). The views expressed in this publication are those of the authors and not necessarily those of the National Health Service, the NIHR or the Department of Health.

COMPETING INTERESTS

The authors report that they do not have any conflicts of interest.

FUNDING

At the time of the study LD, CP, DW and SS were funded by the United Kingdom's National Institute for Health Research (NIHR), and AS was partly funded by the UK NIHR.

DATA SHARING

A full list of health technologies included in the study is available in a supplemental document submitted with this article. More details on data are available from the corresponding author at l.doos@bham.ac.uk.

REFERENCES

1. Herndon JH, Wang RH, Bozic KH. Healthcare technology and technology assessment. *Eur Spine J* 2007;16:1293–1302.
2. Beller GA. New technology and the cost of health care. *J Nucl Cardiol* 2005;12:515-7.
3. Simpson S, Hyde C, Cook A, et al. Assessing the accuracy of forecasting: applying standard diagnostic assessment tools to a health technology early warning system. Int J Technol Assess Health Care 2004;20(3):381-4.
4. Lerner JC, Robertson DC, Goldstein SM. Case studies on forecasting for innovative technologies: Frequent revisions improve accuracy. *Health Aff* 2015; 34(2):311-318.
5. Pankratova N, Bubnov RV. Solving strategic problems of the future on the basis of foresight methodology. *EPMA Journal* 2014;5(Suppl 1) A65-66.

For peer review only - http://bmjopen.bmj.com/site/about/guidelines.xhtml

6. Loveridge D, Georghiou L, Nedeva M. *United Kingdom Technology Foresight Programme: Delphi Survey*. A report by the Office of Science and Technology. Manchester, UK: Policy Research in Engineering, Science and Technology (PREST), 1995.
7. Stevens A, Robert N, Gabbay J. Identifying new health care technologies in the United Kingdom. *Int J Technol Assess Health Care* 1997;13:59–67.
8. Tremblay M, Yiu SH. Horizon Scan of Diagnostic Technologies-Technology push or treatment pull? Report published by Rainmaker Publications 2006. http://www.foresightfordevelopment.org/sobipro/55/365-horizon-scan-of-diagnostic-technologies-technology-push-or-treatment-pull (Accessed 2June 2014).
9. Doos L, Packer C, Ward D, Simpson S, Stevens A. Past speculations of the future: A review of the methods used for forecasting emerging health technologies. BMJ Open 2016 6:e010479 doi:10.1136/bmjopen-2015-010479.
10. The National Institute for Health Research Health Technology Assessment (NIHR HTA) programme. http://www.nets.nihr.ac.uk/programmes/hta/remit (Accessed 4 July 2016).
11. World Health Organisation (WHO). International statistical classification of diseases and related health problems. - 10th revision (ICD-10), Fifth edition, 2016. http://apps.who.int/classifications/icd10/browse/2016/en (Accessed 5 July 2016).
12. Rosin RD, Kemp CR. Operating theatre of the year 2010. Report by Department of Trade and Industry. DTI, 1999.
13. BT calendar 2005. Personal communication with the editor Ian Pearson.
14. Institute of the Future_HC2020 _The future of science, technology & wellbeing. http://www.iftf.org/our-work/health-self/health-horizons/hh2010-the-future-of-science-tech-and-well-being/ (Accessed 21 March 2015).
15. Berzofsky JA, Ahlers JD, Janik J, et al. Progress on new vaccine strategies against chronic viral infections. *J Clin Invest* Aug 2004;114:4: 450:462.
16. Cahill E, Scapolo F. The Future Project Technology Map. A report by European Commission Directorate General, December 1999.
17. Begley CG, Ellis LM. Raise standards for preclinical cancer research. *Nature* 29 March 2012;483:531-533.

For peer review only - http://bmjopen.bmj.com/site/about/guidelines.xhtml

18. Lindvall O, Kokaia Z, Martinez-Serrano A. Stem cell therapy for neurodegenerative disorders-How to make it work. *Nat Med* July 2004 (10); S42-S50.

19. Aboody K, Capela A, Niazi N, et al. Translating stem cell studies to the clinic for CNS repair: Current state of the art and the need for a rosetta stone. *Neuron* May 26, 2011;70:597-613.

For peer review only

For peer review only - http://bmjopen.bmj.com/site/about/guidelines.xhtml

Table 1: The 15 forecasting studies identified in the prior systematic review

Study name or first author	Year of forecast	Country
Dutch Steering Committee on Future Health Scenarios (STG) 1988	1986	Netherlands
Spiby, 1988	1988	UK
Loveridge et al 1995	1994	UK
Stevens et al, 1997	1995	UK
Karim, National Research and Technology Foresight Project, 1999	1996	South Africa
Operating theatre of the year 2010; Department of Trade and Industry report 1999	1996	UK
Cahill and Scapolo, 1999	1998	Europe
Daar et al, 2002	2002	Developing countries
Technology foresight towards 2020_China	2003	China
British Telecommunications (BT) calendar	1997 & 2005	United Kingdom (UK)
Tremblay and Yiu, 2006	2006	Canada
Food and Drug Administration (FDA) surveys	1998 & 2008	United States of America (USA)
Institute of the Future, 2009	2009	USA
Science & Technology Foresight Survey, 2010	Every 5 years from 1971, latest survey 2009-10	Japan
UK technology and innovation futures for the 2020s, 2010	2010	UK

Source: Doos et al 2016[9]

For peer review only - http://bmjopen.bmj.com/site/about/guidelines.xhtml

Table 2: Health technologies by technology type

**Technology type**	**Number**	**%**
Diagnostic tests and imaging	131	19.1
Devices and biomaterials	98	14.3
IT systems, eHealth and mHealth	86	12.6
Drugs (not prophylactic vaccines)	82	12.0
Regenerative medicine	80	11.7
Medical equipment	37	5.4
Therapeutic procedures	36	5.3
Unknown	33	4.8
Organisational programmes	26	3.8
Prophylactic vaccines	24	3.5
Population programmes	24	3.5
Non-surgical therapy	15	2.2
Other	9	1.3
Assistive devices	4	0.6
**Total**	**685**	**100.0**

For peer review only

Table 3: Technology type by forecasting group

Technology type	Forecasting group						Total
	Commercial organisation	Consultancy agency	Governmental agency	Independent non-profit organisation	Researchers	Policy research group	
Assistive devices	3 (3.2%)	0 (0.0%)	1 (0.3%)	0 (0.0%)	0 (0.0%)	0 (0.0%)	4 (0.6%)
Devices & biomaterials	16 (16.8%)	6 (10.9%)	56 (15.6%)	3 (17.6%)	7 (11.5%)	10 (10.1)	98 (14.3%)
Diagnostic tests & imaging	6 (6.3%)	26 (47.3%)	63 (17.6%)	3 (17.6%)	9 (14.8%)	24 (24.2%)	131 (19.1)
Drugs (non-prophylactic vaccine)	7 (7.4%)	2 (3.6%)	31 (8.7%)	1 (5.9%)	22 (36.1%)	19 (19.2%)	82 (12.0%)
IT systems, eHealth, mHealth	18 (18.9%)	7 (12.7%)	43 (12.0%)	3 (17.6%)	3 (4.9%)	12 (12.1%)	86 (12.6%)
Medical equipment	2 (2.1%)	5 (5.5%)	31 (8.7%)	0 (0.0%)	1 (1.6%)	0 (0.0%)	37 (5.4%)
Non-surgical therapy	1 (1.1%)	0 (0.0%)	9 (2.5%)	1 (5.9%)	3 (4.9%)	1 (1.0%)	15 (2.2%)
Population programme	5 (5.3%)	0 (0.0%)	9 (2.5%)	3 (17.6%)	1 (1.6%)	6 (6.1%)	24 (3.5%)

For peer review only

For peer review only - http://bmjopen.bmj.com/site/about/guidelines.xhtml

Prophylactic vaccine	0 (0.0%)	0 (0.0%)	18 (5.0%)	0 (0.0%)	0 (0.0%)	6 (6.1%)	24 (3.5%)
Regenerative Medicine	22 (23.2%)	1 (1.8%)	48 (13.4%)	1 (5.9%)	4 (6.6%)	4 (4.0%)	80 (11.7%)
Therapeutic procedure	0 (0.0%)	1 (1.8%)	20 (5.6%)	0 (0.0%)	6 (9.8%)	9 (9.1%)	36 (5.3%)
Organisational programme	2 (2.1%)	10 (10.6%)	11 (3.1%)	1 (5.9%)	4 (6.6%)	2 (2.0%)	26 (3.8%)
Unknown	9 (9.5%)	2 (3.6%)	16 (4.5%)	1 (5.9%)	1 (1.6%)	4 (4.0%)	33 (4.8%)
Others	4 (4.2%)	1 (1.8%)	2 (0.6%)	0 (0.0%)	0 (0.0%)	2 (2.0%)	9 (1.3%)
**Total**	95 (100%)	55 (100%)	358 (100%)	17 (100%)	61 (100%)	99 (100%)	685 (100%)

For peer review only - http://bmjopen.bmj.com/site/about/guidelines.xhtml

Table 4: Technology type and purpose for the five most common ICD_10 chapter headings

**Technology type**	Infectious disease ICD_I	Cancer ICD_II	Circulatory disorders ICD_IX	Nervous system ICD_VI	Health status, screening, rehab ICD_XXI	**Total**
Assistive devices	-	-	-	-	3 (8.6)	**3 (1.5)**
Diagnostic tests and imaging	5 (10.4)	12 (30.0)	7 (18.4)	6 (16.7)	2 (5.7)	**32 (16.2)**
Devices and biomaterials	3 (6.3)	1 (2.5)	10 (26.3)	9 (25.0)	11 (31.4)	**34 (17.3)**
IT systems, eHealth and mHealth	-	-	-	1 (2.8)	3 (8.6)	**4 (2.0)**
Drugs (not prophylactic vaccines)	10 (20.8)	16 (40.0)	7 (18.4)	2 (5.6)	5 (14.3)	**40 (20.3)**
Regenerative medicine	-	2 (5.0)	7 (18.4)	9 (25.0)	-	**18 (9.1)**
Medical equipment	2 (4.2)	-	-	1 (2.8)	1 (2.9)	**4 (2.0)**
Therapeutic procedures	1 (2.1)	1 (2.5)	3 (7.9)	4 (11.1)	-	**9 (4.6)**
Organisational programmes	3 (6.3)	-	-	-	1 (2.9)	**4 (2.0)**
Prophylactic vaccines	22 (45.8)	-	-	-	1 (2.9)	**23 (11.7)**
Population programmes	1 (2.1)	2 (5.0)	2 (5.3)	1 (2.8)	3 (8.6)	**9 (4.6)**
Non-surgical therapy	-	2 (5.0)		1 (2.8)	-	**3 (1.5)**
Other/ unknown	1 (2.1)	4 (10.0)	2 (5.3)	2 (5.6)	5 (14.3)	**14 (7.1)**
**Total**	**48 (100%)**	**40 (100%)**	**38 (100%)**	**36 (100%)**	**35 (100%)**	**197 (100%)**
**Technology purpose**						
Diagnosis & monitoring of disease	7 (14.6)	12 (30.0)	8 (21.1)	7 (19.4)	1 (2.9)	**35 (17.8)**
Health promotion & disease prevention	29 (60.4)	5 (12.5)	5 (13.2)	1 (2.8)	18 (51.4)	**58 (29.4)**

For peer review only - http://bmjopen.bmj.com/site/about/guidelines.xhtml

Treatment & disease management	11 (22.9)	22 (55.0)	25 (65.8)	22 (61.1)	1 (2.9)	**81 (41.1)**
Rehabilitation	0 (0.0)	0 (0.0)	0 (0.0)	4 (11.1)	15 (42.9)	**19 (9.6)**
Other and unknown	1 (2.1)	1 (2.5)	0 (0.0)	2 (5.6)	0 (0.0)	**4 (2.0)**
**Total**	**48 (100%)**	**40 (100%)**	**38 (100%)**	**36 (100%)**	**35 (100%)**	**197 (100%)**

For peer review only - http://bmjopen.bmj.com/site/about/guidelines.xhtml

Table5: Mean technology prediction timeframe (years) by technology type*

Technology type	Number	Mean timeframe Years (SD)
Non-surgical therapy	14	9.07 (5.7)
Diagnostic tests and imaging	113	10.11 (6.0)
Drugs (not prophylactic vaccines)	75	10.31 (6.3)
Therapeutic procedures	31	10.45 (8.2)
Organisational programmes	25	10.48 (5.3)
IT systems, eHealth and mHealth	77	11.08 (5.5)
Devices and biomaterials	88	11.93 (6.8)
Assistive devices	3	12.33 (17.9)
Other/ unknown	40	13.35 (7.2)
Medical equipment	36	13.53 (2.7)
Regenerative medicine	73	13.58 (7.4)
Prophylactic vaccines	13	14.00 (6.3)
Population programmes	19	14.53 (5.9)
**Total**	**607**	**11.59 (6.6)**

*p<0.002

Figure 1: Mean forecast timeframe by methods of forecasting

For peer review only

For peer review only - http://bmjopen.bmj.com/site/about/guidelines.xhtml

Figure 1: Mean forecast timeframe by methods of forecasting*

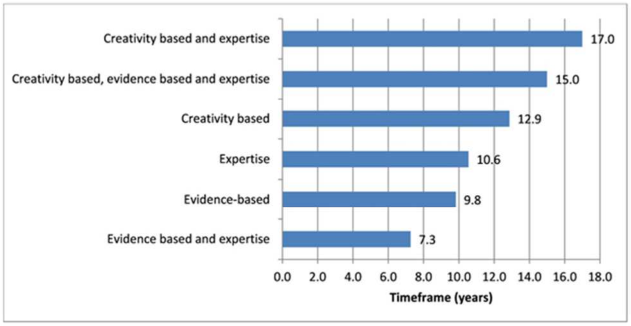

*p<0.001

81x60mm (300 x 300 DPI)

ew only

Appendix1: List of identified healthcare technologies

Report name (year)	Intention of the study	Technology Name	Tech. time frame	Purpose of the technology	Specialty	Technology type
BT Calendar 1997	Commercial	Artificial muscle use in place of heart transplants	1	Treatment and management of symptoms and disease	IX - circulation	Regenerative medicine
BT Calendar 1997	Commercial	Telemed link to home with home blood test kit	1	Diagnosis and monitoring of disease	Other - generic	IT systems, eHealth and mHealth
BT Calendar 1997	Commercial	Artificial intelligence based elderly and handicapped support devices	1	Rehabilitation	XXI - screening, rehab	Assistive devices
BT Calendar 1997	Commercial	Artificial pancreas	1	Treatment and management of symptoms and disease	XI - GI tract, dental health	Regenerative medicine
BT Calendar 1997	Commercial	Artificial blood	3	Treatment and management of symptoms and disease	III - blood	Regenerative medicine
BT Calendar 1997	Commercial	Disability assistance devices using thought recognition	3	Rehabilitation	XXI - screening, rehab	Assistive devices
BT Calendar 1997	Commercial	Artificial ears	3	Rehabilitation	VIII - ear	Regenerative medicine
BT Calendar 1997	Commercial	Electronic implants used to stimulate muscles in disabled people	5	Rehabilitation	XIII - musculoskeletal	Devices and biomaterials
BT Calendar 1997	Commercial	Home (health) diagnostic systems, daily/real time check-up	1	Diagnosis and monitoring of disease	Other - generic	IT systems, eHealth and mHealth
BT Calendar 1997	Commercial	Virtual Reality based exercisers in homes	1	Health promotion and prevention of disease	XXI - screening, rehab	IT systems, eHealth and mHealth
BT Calendar 1997	Commercial	Synthetic retinal implants for simple vision	8	Rehabilitation	VII - eye	Regenerative medicine
BT Calendar 1997	Commercial	Personal wearable health monitor	6	Health promotion and prevention of disease	XXI - screening, rehab	IT systems, eHealth and mHealth
BT Calendar 1997	Commercial	Artificial heart	13	Treatment and management of symptoms and disease	IX - circulation	Regenerative medicine

For peer review only - http://bmjopen.bmj.com/site/about/guidelines.xhtml

Appendix1: List of identified healthcare technologies

BT Calendar 1997	Commercial	Genetic screening widely used	10	Health promotion and prevention of disease	XXI - screening, rehab	Population programmes
BT Calendar 1997	Commercial	Fine particle beam gene engineering	12	Treatment and management of symptoms and disease	Other - generic	Regenerative medicine
BT Calendar 1997	Commercial	Artificial senses, sensors directly stimulating nerves	15	Rehabilitation	XXI - screening, rehab	Devices and biomaterials
BT Calendar 1997	Commercial	Prevention of cancer	16	Health promotion and prevention of disease	II - cancers	Population programmes
BT Calendar 1997	Commercial	Custom foods for particular medical conditions will exist.	13	Treatment and management of symptoms and disease	Other - generic	Other
BT Calendar 1997	Commercial	Genetic programs to enhance human well-being	23	Health promotion and prevention of disease	V - mental health	Population programmes
BT Calendar 1997	Commercial	Artificial lungs	18	Treatment and management of symptoms and disease	X - respiratory	Regenerative medicine
BT Calendar 1997	Commercial	Artificial kidneys	18	Treatment and management of symptoms and disease	XIV - genitourinary	Regenerative medicine
BT Calendar 1997	Commercial	Artificial brain cells	20	Treatment and management of symptoms and disease	VI - nervous	Regenerative medicine
BT Calendar 1997	Commercial	Artificial liver	23	Treatment and management of symptoms and disease	XI - GI tract, dental health	Regenerative medicine
BT Calendar 1997	Commercial	Extension of average lifespan to over 100	23	Health promotion and prevention of disease	XXI - screening, rehab	Unknown
BT Calendar 1997	Commercial	Artificial brain implants	28	Treatment and management of symptoms and disease	VI - nervous	Regenerative medicine
BT Calendar 1997	Commercial	Artificial peripheral nerves	28	Treatment and management of symptoms and disease	VI - nervous	Regenerative medicine
BT Calendar 1997	Commercial	Artificial legs	33	Rehabilitation	XXI - screening, rehab	Unknown

For peer review only - http://bmjopen.bmj.com/site/about/guidelines.xhtml

Appendix1: List of identified healthcare technologies

BT Calendar 1997	Commercial	Fully functional artificial eyes	33	Treatment and management of symptoms and disease	VII - eye	Regenerative medicine
BT Calendar 2005	Commercial	laser activated drug capsules	3	Treatment and management of symptoms and disease	Other - generic	drugs (not prophylactic vaccines)
BT Calendar 2005	Commercial	ultrasound or radio activated capsules	3	Treatment and management of symptoms and disease	Other - generic	drugs (not prophylactic vaccines)
BT Calendar 2005	Commercial	blood analysis chips	3	Diagnosis and monitoring of disease	Other - generic	Diagnostic tests and imaging
BT Calendar 2005	Commercial	e-Baybies electronic conception	5	Health promotion and prevention of disease	XXI - screening, rehab	Unknown
BT Calendar 2005	Commercial	Instant electronic identification of pathogens	5	Diagnosis and monitoring of disease	I - infections	Diagnostic tests and imaging
BT Calendar 2005	Commercial	Online surgeries dominate first line medical care	5	Supporting the provision of care and/or increasing service efficiency	Other - generic	IT systems, eHealth and mHealth
BT Calendar 2005	Commercial	active skin	5	Unknown	XII - skin	Unknown
BT Calendar 2005	Commercial	Cyber-drugs (electronically activated drugs)	5	Treatment and management of symptoms and disease	Other - generic	Drugs (not prophylactic vaccines)
BT Calendar 2005	Commercial	automated pain relief for soldiers	5	Treatment and management of symptoms and disease	XIX - injury	Devices and biomaterials
BT Calendar 2005	Commercial	bacteria in toothpaste to attack plaque	5	Health promotion and prevention of disease	XI - GI tract, dental health	Non-surgical therapy
BT Calendar 2005	Commercial	e-baybies, binary conception	5	Health promotion and prevention of disease	XXI - screening, rehab	Unknown
BT Calendar 2005	Commercial	hand-held scanner to detect tumours using tissue resonance interferometer	5	Diagnosis and monitoring of disease	II - cancers	Diagnostic tests and imaging
BT Calendar 2005	Commercial	smart pill bottles remotely monitor medication taking and use alarms	5	Supporting patients receiving care (designed to be used by individuals)	Other - generic	IT systems, eHealth and mHealth

For peer review only - http://bmjopen.bmj.com/site/about/guidelines.xhtml

Appendix1: List of identified healthcare technologies

BT Calendar 2005	Commercial	diabetes cure via stem cell research	5	Treatment and management of symptoms and disease	IV - endocrine	Regenerative medicine
BT Calendar 2005	Commercial	Use of human's own tissues to grow replacement organs	8	Treatment and management of symptoms and disease	Other - generic	Regenerative medicine
BT Calendar 2005	Commercial	Artificial senses	8	Rehabilitation	XXI - screening, rehab	Devices and biomaterials
BT Calendar 2005	Commercial	context sensitive cyber drugs	8	Treatment and management of symptoms and disease	Other - generic	Unknown
BT Calendar 2005	Commercial	electronic stimulation of brain sensations as recreational substitute for drugs	8	Other	VI - nervous	Devices and biomaterials
BT Calendar 2005	Commercial	Custom (GM) foods for particular medical conditions will exist.	8	Treatment and management of symptoms and disease	Other - generic	Other
BT Calendar 2005	Commercial	Gene-gel stimulation of re-growth of natural teeth on demand	8	Treatment and management of symptoms and disease	XI - GI tract, dental health	drugs (not prophylactic vaccines)
BT Calendar 2005	Commercial	retina regeneration using foetal retinal cell injection	8	Treatment and management of symptoms and disease	VII - eye	Regenerative medicine
BT Calendar 2005	Commercial	tooth regeneration	8	Treatment and management of symptoms and disease	XI - GI tract, dental health	Regenerative medicine
BT Calendar 2005	Commercial	plastic bones	8	Treatment and management of symptoms and disease	XIII - musculoskeletal	Devices and biomaterials
BT Calendar 2005	Commercial	emotionally specific drugs	8	Unknown	V - mental health	drugs (not prophylactic vaccines)
BT Calendar 2005	Commercial	synthetic organs by printing layers of cells	10	Treatment and management of symptoms and disease	Other - generic	Regenerative medicine
BT Calendar 2005	Commercial	synthetic viruses	10	Unknown	I -	Other

For peer review only - http://bmjopen.bmj.com/site/about/guidelines.xhtml

Appendix1: List of identified healthcare technologies

					infections	
BT Calendar 2005	Commercial	sensory augmentation using sensory implants, nanoparticles etc	10	Rehabilitation	VI - nervous	Devices and biomaterials
BT Calendar 2005	Commercial	use of stem cells in brain after strokes or accidents	10	Treatment and management of symptoms and disease	VI - nervous	Regenerative medicine
BT Calendar 2005	Commercial	gene therapy generates new hair cells in humans	10	Treatment and management of symptoms and disease	XII - skin	drugs (not prophylactic vaccines)
BT Calendar 2005	Commercial	artificial sensors used in cosmetic upgrade surgery	13	Treatment and management of symptoms and disease	Other - generic	Devices and biomaterials
BT Calendar 2005	Commercial	Electronic memory enhancement	20	Unknown	Other - generic	Unknown
BT Calendar 2005	Commercial	Nanobots in toothpaste attack plaque	20	Health promotion and prevention of disease	XI - GI tract, dental health	drugs (not prophylactic vaccines)
BT Calendar 2005	Commercial	Fully functioning artificial eyes	20	Treatment and management of symptoms and disease	VII - eye	Regenerative medicine
BT Calendar 2005	Commercial	electronic brain implants	20	Treatment and management of symptoms and disease	VI - nervous	Devices and biomaterials
BT Calendar 2005	Commercial	Intelligence or memory enhancement by external means	20	Unknown	Other - generic	Unknown
BT Calendar 2005	Commercial	e-baybies, genetic conception	20	Health promotion and prevention of disease	XXI - screening, rehab	Unknown
BT Calendar 2005	Commercial	synthetic immune system	20	Treatment and management of symptoms and disease	III - blood	Regenerative medicine
BT Calendar 2005	Commercial	Artificial peripheral nerves	30	Treatment and management of symptoms and disease	VI - nervous	Regenerative medicine
BT Calendar 2005	Commercial	e-baybies, digital emulation conception	30	Health promotion and prevention of disease	XXI - screening, rehab	Unknown

For peer review only - http://bmjopen.bmj.com/site/about/guidelines.xhtml

Appendix1: List of identified healthcare technologies

BT Calendar 2005	Commercial	Electronic prescriptions reduce fraud and improve speed	3	Supporting the provision of care and/or increasing service efficiency	Other - generic	IT systems, eHealth and mHealth
BT Calendar 2005	Commercial	24/7 blood chemistry monitoring	3	Diagnosis and monitoring of disease	Other - generic	Diagnostic tests and imaging
BT Calendar 2005	Commercial	supermarkets used as major source of medical alerts	3	Health promotion and prevention of disease	Other - generic	Other
BT Calendar 2005	Commercial	Expert systems used extensively in GP surgeries	5	Supporting patients receiving care (designed to be used by individuals)	Other - generic	IT systems, eHealth and mHealth
BT Calendar 2005	Commercial	Hospitals use virtual queuing systems	5	Supporting the provision of care and/or increasing service efficiency	Other - generic	IT systems, eHealth and mHealth
BT Calendar 2005	Commercial	Neural networks used for patient appointment management	5	Supporting the provision of care and/or increasing service efficiency	Other - generic	IT systems, eHealth and mHealth
BT Calendar 2005	Commercial	Multimedia patient records	5	Supporting patients receiving care (designed to be used by individuals)	Other - generic	IT systems, eHealth and mHealth
BT Calendar 2005	Commercial	Lifestyle monitoring and insurance linked to medical records	5	Health promotion and prevention of disease	Other - generic	Population programmes
BT Calendar 2005	Commercial	antibacterial coatings on domestic appliances, phones etc, especially in hospitals	5	Health promotion and prevention of disease	I - infections	Devices and biomaterials
BT Calendar 2005	Commercial	flexible displays used for body monitoring and alerts	5	Diagnosis and monitoring of disease	Other - generic	IT systems, eHealth and mHealth
BT Calendar 2005	Commercial	emotion alerts	5	Health promotion and prevention of disease	Other - generic	Devices and biomaterials
BT Calendar 2005	Commercial	(drug shortages caused by) internet diagnosis	5	Diagnosis and monitoring of disease	Other - generic	IT systems, eHealth and mHealth

For peer review only - http://bmjopen.bmj.com/site/about/guidelines.xhtml

Appendix1: List of identified healthcare technologies

BT Calendar 2005	Commercial	MRSA detection chips on sale in high street	5	Diagnosis and monitoring of disease	I - infections	Population programmes
BT Calendar 2005	Commercial	Devices roaming within blood vessels under own power	8	Unknown	Other - generic	Devices and biomaterials
BT Calendar 2005	Commercial	Operations videoed and stored as part of medical record	8	Supporting patients receiving care (designed to be used by individuals)	Other - generic	IT systems, eHealth and mHealth
BT Calendar 2005	Commercial	Direct electronic pleasure production	8	Other	V - mental health	IT systems, eHealth and mHealth
BT Calendar 2005	Commercial	Orgasm by email	8	Supporting patients receiving care (designed to be used by individuals)	XIV - genitourinary	IT systems, eHealth and mHealth
BT Calendar 2005	Commercial	Artificial senses, sensors directly stimulating nerves	8	Rehabilitation	XXI - screening, rehab	Devices and biomaterials
BT Calendar 2005	Commercial	Smart membranes for remote control of drug injection	8	Treatment and management of symptoms and disease	Other - generic	Devices and biomaterials
BT Calendar 2005	Commercial	Domestic bacterial detection devices	8	Health promotion and prevention of disease	I - infections	Diagnostic tests and imaging
BT Calendar 2005	Commercial	Micro-fluidic chips used for gene sequencing in every GP surgery	8	Diagnosis and monitoring of disease	Other - generic	Diagnostic tests and imaging
BT Calendar 2005	Commercial	Self certification for prescriptions using electronic diagnostics	8	Supporting patients receiving care (designed to be used by individuals)	Other - generic	IT systems, eHealth and mHealth
BT Calendar 2005	Commercial	Visitor sanitising in hospitals	8	Health promotion and prevention of disease	I - infections	Organisational programmes
BT Calendar 2005	Commercial	Outpatients at home - remote tele-medical consultations	8	Supporting patients receiving care (designed to be used by individuals)	Other - generic	IT systems, eHealth and mHealth
BT Calendar 2005	Commercial	Individual's genome part of their medical record	8	Supporting patients receiving care (designed to be used by	Other - generic	Organisational programmes

For peer review only - http://bmjopen.bmj.com/site/about/guidelines.xhtml

Appendix1: List of identified healthcare technologies

				individuals)		
BT Calendar 2005	Commercial	Portable bactericidal devices	10	Health promotion and prevention of disease	I - infections	Medical equipment
BT Calendar 2005	Commercial	Bactericidal saws incorporated onto materials in hospitals	10	Health promotion and prevention of disease	I - infections	Devices and biomaterials
BT Calendar 2005	Commercial	Robotic cleaners in hospitals	10	Supporting the provision of care and/or increasing service efficiency	Other - generic	Medical equipment
Daar et al 2002	Research	Modified molecular technologies for affordable, simple diagnosis of infectious diseases	8	Diagnosis and monitoring of disease	I - infections	Diagnostic tests and imaging
Daar et al 2002	Research	Recombinant technologies to develop vaccines against infectious diseases	8	Health promotion and prevention of disease	I - infections	Prophylactic vaccines
Daar et al 2002	Research	Technologies for more efficient drug and vaccine delivery systems	8	Treatment and management of symptoms and disease	Other - generic	Unknown
Daar et al 2002	Research	Technologies for environmental improvement (sanitation, clean water, bioremediation)	8	Health promotion and prevention of disease	Other - generic	Unknown
Daar et al 2002	Research	Female-controlled protection against sexually transmitted diseases, both with and without contraceptive effect	8	Health promotion and prevention of disease	XIV - genitourinary	Devices and biomaterials
Daar et al 2002	Research	Genetically modified crops with increased nutrients to counter specific deficiencies	8	Health promotion and prevention of disease	IV - endocrine	Unknown

For peer review only - http://bmjopen.bmj.com/site/about/guidelines.xhtml

Appendix1: List of identified healthcare technologies

Daar et al 2002	Research	Recombinant technology to make therapeutic products (for example, insulin, interferons) more affordable	8	Treatment and management of symptoms and disease	Other - generic	drugs (not prophylactic vaccines)
Karim2000	Policy planning	Telemedicine	15	Supporting patients receiving care (designed to be used by individuals)	Other - generic	IT systems, eHealth and mHealth
Karim2000	Policy planning	Medical databases	15	Supporting the provision of care and/or increasing service efficiency	Other - generic	IT systems, eHealth and mHealth
Karim2000	Policy planning	Expert System in managed care and preventive medicine	15	Supporting patients receiving care (designed to be used by individuals)	Other - generic	IT systems, eHealth and mHealth
Karim2000	Policy planning	diagnostic procedures (e.g. recombinant DNA techniques for parental screening)	15	Diagnosis and monitoring of disease	Other - generic	Diagnostic tests and imaging
Karim2000	Policy planning	Therapeutic procedures (e.g. microsurgery and implanted muscle stimulators).	15	Treatment and management of symptoms and disease	Other - generic	Therapeutic procedures
Karim2000	Policy planning	Nanotechnology (e.g. devices which interact with tissues within the body to boost the immune system).	15	Treatment and management of symptoms and disease	III - blood	Devices and biomaterials
Karim2000	Policy planning	Vaccines against infectious diseases	15	Health promotion and prevention of disease	I - infections	Prophylactic vaccines
Karim2000	Policy planning	Development of an AIDS vaccine	15	Health promotion and prevention of disease	I - infections	Prophylactic vaccines
Karim2000	Policy planning	Development of a TB vaccine	15	Health promotion and prevention of disease	I - infections	Prophylactic vaccines
Karim2000	Policy planning	Health promotion targeted at the youth	15	Health promotion and prevention of disease	Other - generic	Population programmes

For peer review only - http://bmjopen.bmj.com/site/about/guidelines.xhtml

Appendix1: List of identified healthcare technologies

Karim2000	Policy planning	Development of enriched and fortified staple foods	15	Health promotion and prevention of disease	Other - generic	Population programmes
Karim2000	Policy planning	Development of a malaria vaccine	15	Health promotion and prevention of disease	I - infections	Prophylactic vaccines
Karim2000	Policy planning	Development of on-site diagnostic tools	15	Diagnosis and monitoring of disease	Other - generic	Diagnostic tests and imaging
Karim2000	Policy planning	Development of female contraceptives	15	Health promotion and prevention of disease	XXI - screening, rehab	drugs (not prophylactic vaccines)
Karim2000	Policy planning	Development of barrier methods and microbicides for sexually transmitted diseases and HIV	15	Health promotion and prevention of disease	I - infections	Devices and biomaterials
Karim2000	Policy planning	Development of malaria drugs	15	Treatment and management of symptoms and disease	I - infections	drugs (not prophylactic vaccines)
Karim2000	Policy planning	Development of new TB drugs	15	Treatment and management of symptoms and disease	I - infections	drugs (not prophylactic vaccines)
Karim2000	Policy planning	Self management tools for chronic diseases (e.g. hypertension, diabetes, diarrhoea, psychiatric conditions)	15	Supporting patients receiving care (designed to be used by individuals)	Other - generic	Organisational programmes
Karim2000	Policy planning	Tissue regeneration and gene therapy	15	Treatment and management of symptoms and disease	Other - generic	Regenerative medicine
Sbipy 1988	Research	Monoclonal antibodies will be used in: Histopathological techniques	0	Diagnosis and monitoring of disease	Other - generic	Diagnostic tests and imaging
Sbipy 1988	Research	Monoclonal antibodies will be used in: Biochemical techniques	0	Diagnosis and monitoring of disease	Other - generic	Diagnostic tests and imaging
Sbipy 1988	Research	Monoclonal antibodies will be	0	Diagnosis and monitoring of	Other - generic	Diagnostic tests and imaging

For peer review only - http://bmjopen.bmj.com/site/about/guidelines.xhtml

Appendix1: List of identified healthcare technologies

		used in: in-vivo diagnostic techniques		disease		
Sbipy 1988	Research	Monoclonal antibodies will be used in: Identification and localization of tumours	0	Diagnosis and monitoring of disease	II - cancers	Diagnostic tests and imaging
Sbipy 1988	Research	Gene probes will be used in: diagnostic microbiology	0	Diagnosis and monitoring of disease	I - infections	Diagnostic tests and imaging
Sbipy 1988	Research	Gene probes will be used in: diagnostic virology	0	Diagnosis and monitoring of disease	I - infections	Diagnostic tests and imaging
Sbipy 1988	Research	Gene probes will be used in: To screen for potentially deleterious genes*	0	Diagnosis and monitoring of disease	Other - generic	Diagnostic tests and imaging
Sbipy 1988	Research	Gene probes will be used in: Diagnosing most genetic disorders	10	Diagnosis and monitoring of disease	XVII - congenital	Diagnostic tests and imaging
Sbipy 1988	Research	Genetic engineering will enable the isolation of high risk groups for common diseases	10	Health promotion and prevention of disease	Other - generic	Population programmes
Sbipy 1988	Research	Imaging techniques will be in widespread and less hazardous use including: Ultrasound	0	Diagnosis and monitoring of disease	Other - generic	Diagnostic tests and imaging
Sbipy 1988	Research	Imaging techniques will be in widespread and less hazardous use including: Doppler measurement	0	Diagnosis and monitoring of disease	Other - generic	Diagnostic tests and imaging
Sbipy 1988	Research	Imaging techniques will be in widespread and less hazardous use including: CT scanning	0	Diagnosis and monitoring of disease	Other - generic	Diagnostic tests and imaging

For peer review only - http://bmjopen.bmj.com/site/about/guidelines.xhtml

Appendix1: List of identified healthcare technologies

Sbipy 1988	Research	Imaging techniques will be in widespread and less hazardous use including: NMR scanning	0	Diagnosis and monitoring of disease	Other - generic	Diagnostic tests and imaging
Sbipy 1988	Research	Imaging techniques will be in widespread and less hazardous use including: Nuclear medicine	0	Diagnosis and monitoring of disease	Other - generic	Diagnostic tests and imaging
Sbipy 1988	Research	Imaging techniques will be in widespread and less hazardous use including: Positron detection*	0	Diagnosis and monitoring of disease	Other - generic	Diagnostic tests and imaging
Sbipy 1988	Research	Imaging techniques will be in widespread and less hazardous use including: Three-dimensional dynamic imaging	10	Diagnosis and monitoring of disease	Other - generic	Diagnostic tests and imaging
Sbipy 1988	Research	Drug therapy will be enhanced by: Genetic engineering	10	Treatment and management of symptoms and disease	Other - generic	drugs (not prophylactic vaccines)
Sbipy 1988	Research	Drug therapy will be enhanced by: Monoclonal antibodies used for drug targeting	10	Treatment and management of symptoms and disease	Other - generic	drugs (not prophylactic vaccines)
Sbipy 1988	Research	Drug delivery devices with or without in-vivo sensors	10	Treatment and management of symptoms and disease	Other - generic	Devices and biomaterials
Sbipy 1988	Research	More effective Therapeutics will be available for: Viral infections	0	Treatment and management of symptoms and disease	I - infections	drugs (not prophylactic vaccines)
Sbipy 1988	Research	More effective Therapeutics will be available for: Autoimmune diseases (monoclonal antibodies)	10	Treatment and management of symptoms and disease	III - blood	drugs (not prophylactic vaccines)
Sbipy 1988	Research	More effective Therapeutics will	0	Treatment and management of	IX - circulation	drugs (not prophylactic

For peer review only - http://bmjopen.bmj.com/site/about/guidelines.xhtml

Appendix1: List of identified healthcare technologies

		be available for: Heart failure (better drugs)		symptoms and disease		vaccines)
Sbipy 1988	Research	More effective Therapeutics will be available for: Obstructive arterial lesions (by removal and control of platelets and synthetic vascular prostheses)	10	Treatment and management of symptoms and disease	IX - circulation	drugs (not prophylactic vaccines)
Sbipy 1988	Research	Cancer (hyperthermia, growth regulation factors and monoclonal antibodies)	10	Other	II - cancers	Other
Sbipy 1988	Research	Cardiac arrhythmias (implantable mechanisms)	10	Treatment and management of symptoms and disease	IX - circulation	Devices and biomaterials
Sbipy 1988	Research	More effective Therapeutics will be available for: Arthritic joints (more biocompatible prosthetic materials and a wider range of joint replacements)	0	Treatment and management of symptoms and disease	XIII - musculoske letal	Devices and biomaterials
Sbipy 1988	Research	More effective Therapeutics will be available for: Incontinence (stimulation via implantable electrodes)	0	treatment and management of symptoms and disease	XIV - genitourina ry	Devices and biomaterials
Sbipy 1988	Research	More effective Therapeutics will be available for: Disability (wider range of flow technology aids and biological servomechanisms linked to computers and	0	Rehabilitation	Other - generic	Other

For peer review only - http://bmjopen.bmj.com/site/about/guidelines.xhtml

Appendix1: List of identified healthcare technologies

		robotics)				
Sbipy 1988	Research	More effective Therapeutics will be available for: Limb damage (permanent internal bone repair)	0	Treatment and management of symptoms and disease	XIII - musculoskeletal	Devices and biomaterials
Sbipy 1988	Research	More effective Therapeutics will be available for: Tropical parasitic diseases (vaccines)	0	Health promotion and prevention of disease	I - infections	Prophylactic vaccines
Sbipy 1988	Research	More effective Therapeutics will be available for: Mental disorders (neurotransmitters)	10	Treatment and management of symptoms and disease	V - mental health	drugs (not prophylactic vaccines)
Sbipy 1988	Research	Laser microsurgery	0	Treatment and management of symptoms and disease	Other - generic	Therapeutic procedures
Sbipy 1988	Research	Laser endoscopic surgery	0	Treatment and management of symptoms and disease	Other - generic	Therapeutic procedures
Sbipy 1988	Research	Laser angioplasty and angiography	0	Treatment and management of symptoms and disease	IX - circulation	Therapeutic procedures
Sbipy 1988	Research	Other laser and endoscopic surgical techniques	10	Treatment and management of symptoms and disease	Other - generic	Therapeutic procedures
Sbipy 1988	Research	Cryosurgery	10	Treatment and management of symptoms and disease	Other - generic	Therapeutic procedures
Sbipy 1988	Research	Lithotripsy	10	Treatment and management of symptoms and disease	Other - generic	Therapeutic procedures
Sbipy 1988	Research	Transplantation will be enhanced by better techniques	0	Treatment and management of symptoms and disease	Other - generic	Devices and biomaterials

For peer review only - http://bmjopen.bmj.com/site/about/guidelines.xhtml

Appendix1: List of identified healthcare technologies

		enabling long term in-vitro organ preservation and the prevention of rejection				
Sbipy 1988	Research	Bone marrow transplantation with purified stem cells and autologous material will be available	0	Treatment and management of symptoms and disease	III - blood	Regenerative medicine
Sbipy 1988	Research	A safe male pill for long-term use will be available	10	Health promotion and prevention of disease	XXI - screening, rehab	drugs (not prophylactic vaccines)
Sbipy 1988	Research	Improved techniques for detecting ovulation will enhance natural family planning*	0	Diagnosis and monitoring of disease	XIV - genitourinary	Diagnostic tests and imaging
Sbipy 1988	Research	Optical disc storage and communication will be used with X-rays and other diagnostic images	0	Supporting the provision of care and/or increasing service efficiency	Other - generic	Diagnostic tests and imaging
Sbipy 1988	Research	Computers will be used by clinical departments for data collection	10	Supporting the provision of care and/or increasing service efficiency	Other - generic	IT systems, eHealth and mHealth
Sbipy 1988	Research	Expert interrogation will be possible due to data centralization	0	Supporting the provision of care and/or increasing service efficiency	Other - generic	IT systems, eHealth and mHealth
Sbipy 1988	Research	Patient interviewing systems will be available in the surgery and in patients' homes	10	Supporting patients receiving care (designed to be used by individuals)	Other - generic	IT systems, eHealth and mHealth
Sbipy 1988	Research	Expert systems will enable diagnosis and define the most applicable treatment	10	Supporting patients receiving care (designed to be used by individuals)	Other - generic	IT systems, eHealth and mHealth

For peer review only - http://bmjopen.bmj.com/site/about/guidelines.xhtml

Appendix1: List of identified healthcare technologies

Sbipy 1988	Research	Linkage of computerized patient data will be available between all hospital, community and primary care services and settings	10	Supporting the provision of care and/or increasing service efficiency	Other - generic	IT systems, eHealth and mHealth
STG 1988	Policy planning	Life style changing behaviours	N/A	Health promotion and prevention of disease	Other - generic	Population programmes
STG 1988	Policy planning	Screening for phenylketonuria	N/A	Health promotion and prevention of disease	IV - endocrine	Population programmes
STG 1988	Policy planning	Screening for congenital hypothyroidism	N/A	Health promotion and prevention of disease	IV - endocrine	Population programmes
STG 1988	Policy planning	Blood pressure can be checked by self-service meters	N/A	Diagnosis and monitoring of disease	IX - circulation	Diagnostic tests and imaging
STG 1988	Policy planning	High serum cholesterol can be checked by fingerprick blood test	N/A	Diagnosis and monitoring of disease	IX - circulation	Diagnostic tests and imaging
STG 1988	Policy planning	Certain cancer tests can be self-administered	N/A	Diagnosis and monitoring of disease	II - cancers	Diagnostic tests and imaging
STG 1988	Policy planning	Improved diagnostic tests may be available for widespread use for screening at the community level	N/A	Health promotion and prevention of disease	Other - generic	Population programmes
STG 1988	Policy planning	Improved vaccine against cholera in the tropical developing countries	N/A	Health promotion and prevention of disease	I - infections	Prophylactic vaccines
STG 1988	Policy planning	New malaria vaccine in the tropical developing countries	N/A	Health promotion and prevention of disease	I - infections	Prophylactic vaccines
STG 1988	Policy planning	New vaccine against hepatitus B	N/A	Health promotion and prevention of disease	I - infections	Prophylactic vaccines

For peer review only - http://bmjopen.bmj.com/site/about/guidelines.xhtml

Appendix1: List of identified healthcare technologies

STG 1988	Policy planning	New vaccine against respiratory syncytial virus	N/A	Health promotion and prevention of disease	I - infections	Prophylactic vaccines
STG 1988	Policy planning	New vaccine against influenza	N/A	Health promotion and prevention of disease	I - infections	Prophylactic vaccines
STG 1988	Policy planning	New vaccine against AIDS	N/A	Health promotion and prevention of disease	I - infections	Prophylactic vaccines
STG 1988	Policy planning	New vaccine against cytomegalovirus	N/A	Health promotion and prevention of disease	I - infections	Prophylactic vaccines
STG 1988	Policy planning	Better vaccine against Perussis	N/A	Health promotion and prevention of disease	I - infections	Prophylactic vaccines
STG 1988	Policy planning	Oral administration or administartion by nasal spray will facilitate mass immunization	N/A	Health promotion and prevention of disease	I - infections	Prophylactic vaccines
STG 1988	Policy planning	Creating vaccines containing antigens for several organisms given together (e.g. DPTPM)	N/A	Health promotion and prevention of disease	I - infections	Prophylactic vaccines
STG 1988	Policy planning	Shifts in diet toward lower intake of fat especially saturated fat	N/A	Health promotion and prevention of disease	Other - generic	Population programmes
STG 1988	Policy planning	Reliable and simple test for cholesterol	N/A	Diagnosis and monitoring of disease	IX - circulation	Diagnostic tests and imaging
STG 1988	Policy planning	More effective and safer drugs for treatment of elevated cholesterol	N/A	Health promotion and prevention of disease	IX - circulation	drugs (not prophylactic vaccines)
STG 1988	Policy planning	Technologies for home use without professional supervision (e.g. home diagnostic kits)	N/A	Supporting patients receiving care (designed to be used by individuals)	Other - generic	Diagnostic tests and imaging
STG 1988	Policy planning	Disease conditions will be monitored from distant professional sites	N/A	Supporting patients receiving care (designed to be used by	Other - generic	IT systems, eHealth and mHealth

For peer review only - http://bmjopen.bmj.com/site/about/guidelines.xhtml

Appendix1: List of identified healthcare technologies

				individuals)		
STG 1988	Policy planning	Robotics will be used for mobility and assistance at home such as nutrition and cleaning	N/A	Rehabilitation	Other - generic	Assistive devices
STG 1988	Policy planning	Regeneration of central nervous system through the use of nerve growth factors or transplant neurotransmitter-producing cells into the brain	N/A	Treatment and management of symptoms and disease	VI - nervous	Regenerative medicine
STG 1988	Policy planning	Grafting of the spinal cord or the peripheral nervous system	N/A	Treatment and management of symptoms and disease	VI - nervous	Therapeutic procedures
STG 1988	Policy planning	More safer & effective contraception	N/A	Health promotion and prevention of disease	XXI - screening, rehab	Drugs (not prophylactic vaccines)
STG 1988	Policy planning	Contraceptive vaccine	N/A	Health promotion and prevention of disease	XXI - screening, rehab	Prophylactic vaccines
STG 1988	Policy planning	Alternatives to surgical abortion such as combination of antiprogestins and prostaglandins	N/A	Treatment and management of symptoms and disease	XV - pregnancy	drugs (not prophylactic vaccines)
STG 1988	Policy planning	Treatment of infertility using new technologies such as in vitro fertilization, surrogate motherhood, freezing of germ cells and embro with embryo transfer	N/A	Treatment and management of symptoms and disease	XIV - genitourinary	Other
STG 1988	Policy planning	Fetal cells can be isolated from the mother's bloodstream as a method for prenatal screening	N/A	Health promotion and prevention of disease	XXI - screening, rehab	Diagnostic tests and imaging

For peer review only - http://bmjopen.bmj.com/site/about/guidelines.xhtml

Appendix1: List of identified healthcare technologies

STG 1988	Policy planning	Metabolic, physiologic information can be obtained by spectroscopy	N/A	Diagnosis and monitoring of disease	Other - generic	Diagnostic tests and imaging
STG 1988	Policy planning	Data from CT scanner will be stored on a disk, transmit by a phone line or othe communication	N/A	Supporting patients receiving care (designed to be used by individuals)	Other - generic	Diagnostic tests and imaging
STG 1988	Policy planning	Film may disappear and computer will directly interpret the diagnosis	N/A	Supporting patients receiving care (designed to be used by individuals)	Other - generic	Diagnostic tests and imaging
STG 1988	Policy planning	Biosensors to monitor disease condition or results of therapy (e.g. diabetes)	N/A	Diagnosis and monitoring of disease	Other - generic	Diagnostic tests and imaging
STG 1988	Policy planning	Tumor markers that can be measured in blood	N/A	Diagnosis and monitoring of disease	II - cancers	Diagnostic tests and imaging
STG 1988	Policy planning	Flow cytometer will be used in transplantation or monitoring of cancer therapy	N/A	Diagnosis and monitoring of disease	II - cancers	Diagnostic tests and imaging
STG 1988	Policy planning	Monoclonal antibodies for diagnosis such as colon cancer and area of cardiac infarctions	N/A	Diagnosis and monitoring of disease	Other - generic	Diagnostic tests and imaging
STG 1988	Policy planning	Human gene therapy for a wide variety of diseases including cancer, diabetes, cardiovascular and affective disorders such as depression	N/A	Treatment and management of symptoms and disease	Other - generic	Regenerative medicine
STG 1988	Policy planning	DNA probe for intra-uterine diagnosis of genetic diseases	N/A	Diagnosis and monitoring of disease	XVII - congenital	Diagnostic tests and imaging
STG 1988	Policy planning	Home diagnostic kits for sexually	7	Diagnosis and monitoring of	I - infections	Organisational programmes

For peer review only - http://bmjopen.bmj.com/site/about/guidelines.xhtml

Appendix1: List of identified healthcare technologies

		transmitted diseases and hepatitits		disease		
STG 1988	Policy planning	Home diagnostic kits for screening for cancer	7	Diagnosis and monitoring of disease	I - infections	Organisational programmes
STG 1988	Policy planning	Home diagnostic kits to test for common diseases with genetic basis such as diabetes	N/A	Diagnosis and monitoring of disease	Other - generic	Organisational programmes
STG 1988	Policy planning	Genetic insertion therapy for treatment of certain blood disorders such as thalassaemia and sickle cell anaemia	N/A	Treatment and management of symptoms and disease	III - blood	Regenerative medicine
STG 1988	Policy planning	Oral treatment forms for hormones, enzymes and other peptides may be developed	N/A	Treatment and management of symptoms and disease	Other - generic	Drugs (not prophylactic vaccines)
STG 1988	Policy planning	New drugs for asthma	N/A	Treatment and management of symptoms and disease	X - respiratory	Drugs (not prophylactic vaccines)
STG 1988	Policy planning	New drugs for coronary artery diseases	N/A	Treatment and management of symptoms and disease	IX - circulation	Drugs (not prophylactic vaccines)
STG 1988	Policy planning	Monoclonal antibodies to replace immune globulins	N/A	Treatment and management of symptoms and disease	III - blood	Drugs (not prophylactic vaccines)
STG 1988	Policy planning	Cloning of skin	N/A	Treatment and management of symptoms and disease	XII - skin	Regenerative medicine
STG 1988	Policy planning	Growth of retinal tissue and corneal endothelium	N/A	Treatment and management of symptoms and disease	VII - eye	Regenerative medicine
STG 1988	Policy planning	Total body irradiation for transplant (to prevent rejection)	N/A	Treatment and management of symptoms and disease	III - blood	Non-surgical therapy
STG 1988	Policy planning	Artificial heart	N/A	Treatment and management of symptoms and	IX - circulation	Regenerative medicine

For peer review only

Appendix1: List of identified healthcare technologies

				disease		
STG 1988	Policy planning	Artificial pancreas	N/A	Treatment and management of symptoms and disease	IV - endocrine	Regenerative medicine
STG 1988	Policy planning	Shoulder joint replacement	N/A	Treatment and management of symptoms and disease	XIII - musculoskeletal	Devices and biomaterials
STG 1988	Policy planning	Cochlear implants	N/A	Treatment and management of symptoms and disease	VIII - ear	Devices and biomaterials
STG 1988	Policy planning	Coating of implant materials to improve acceptability	N/A	Other	Other - generic	Devices and biomaterials
STG 1988	Policy planning	Functional electrical stimulation in prosthetics allowing brain to control the action	N/A	Rehabilitation	XXI - screening, rehab	Devices and biomaterials
STG 1988	Policy planning	Prostheic fingers	N/A	Rehabilitation	XIII - musculoskeletal	Devices and biomaterials
STG 1988	Policy planning	Organ transplant and improvement of functional activities of organs (pancreas, bone, small bowel, endocine glands..)	N/A	Treatment and management of symptoms and disease	Other - generic	Other
STG 1988	Policy planning	Microprocessor to restore damaged areas of the brain	N/A	Rehabilitation	VI - nervous	Devices and biomaterials
STG 1988	Policy planning	Laser cytofluorometry will be used to identify RNA and DNA in the plasma	N/A	Diagnosis and monitoring of disease	Other - generic	Diagnostic tests and imaging
STG 1988	Policy planning	Laser doppler to measure blood flow in the retina	N/A	Diagnosis and monitoring of disease	Other - generic	Diagnostic tests and imaging
STG 1988	Policy planning	Laser to stop gastrointestinal bleeding	N/A	Treatment and management of symptoms and disease	XI - GI tract, dental health	Therapeutic procedures

For peer review only - http://bmjopen.bmj.com/site/about/guidelines.xhtml

Appendix1: List of identified healthcare technologies

STG 1988	Policy planning	Laser to break urinary stones	N/A	Treatment and management of symptoms and disease	XIV - genitourinary	Therapeutic procedures
STG 1988	Policy planning	Laser surgery through endoscope	N/A	Treatment and management of symptoms and disease	Other - generic	Therapeutic procedures
STG 1988	Policy planning	Widespread use of microsurgery	N/A	Treatment and management of symptoms and disease	Other - generic	Therapeutic procedures
STG 1988	Policy planning	Electronic gas valve regulator for safety in anaesthaesia	N/A	Supporting the provision of care and/or increasing service efficiency	XIX - injury	Medical equipment
STG 1988	Policy planning	Instantaneous measurement of blood gases and drug levels in the body during anasthaesia	N/A	Supporting patients receiving care (designed to be used by individuals)	XIX - injury	Diagnostic tests and imaging
STG 1988	Policy planning	Computerised ECG operating room	N/A	Supporting the provision of care and/or increasing service efficiency	Other - generic	IT systems, eHealth and mHealth
STG 1988	Policy planning	Data on each patient available at any moment during the operation	N/A	Supporting patients receiving care (designed to be used by individuals)	Other - generic	IT systems, eHealth and mHealth
STG 1988	Policy planning	Computerised electroanaesthesia	N/A	Supporting patients receiving care (designed to be used by individuals)	Other - generic	IT systems, eHealth and mHealth
STG 1988	Policy planning	New methods to close wounds e.g. tissue glue with ultrasound	N/A	Treatment and management of symptoms and disease	Other - generic	Devices and biomaterials
STG 1988	Policy planning	Spectroscopy using optical methods to diagnose caries	N/A	Diagnosis and monitoring of disease	XI - GI tract, dental health	Diagnostic tests and imaging
STG 1988	Policy planning	Bone like implants for teeth and bones	N/A	Treatment and management of symptoms and disease	XI - GI tract, dental health	Devices and biomaterials
STG 1988	Policy planning	Home monitoring especially of the elderly	N/A	Supporting patients receiving care (designed to	Other - generic	IT systems, eHealth and mHealth

For peer review only - http://bmjopen.bmj.com/site/about/guidelines.xhtml

Appendix1: List of identified healthcare technologies

				be used by individuals)		
STG 1988	Policy planning	Networks for communications between and within hospitals	N/A	Supporting the provision of care and/or increasing service efficiency	Other - generic	IT systems, eHealth and mHealth
STG 1988	Policy planning	Huge mass memories (e.g. on laser disc) for the storage of medical records	N/A	Supporting the provision of care and/or increasing service efficiency	Other - generic	IT systems, eHealth and mHealth
STG 1988	Policy planning	Video disk oriented data base for medical records	N/A	Supporting the provision of care and/or increasing service efficiency	Other - generic	IT systems, eHealth and mHealth
STG 1988	Policy planning	Computer aided examination and testing	N/A	Supporting patients receiving care (designed to be used by individuals)	Other - generic	IT systems, eHealth and mHealth
STG 1988	Policy planning	Improved biomaterials for joint implants and plastic surgery	N/A	Treatment and management of symptoms and disease	XIII - musculoskeletal	Devices and biomaterials
STG 1988	Policy planning	Improved materials for artificial blood vessels	N/A	Treatment and management of symptoms and disease	IX - circulation	Devices and biomaterials
Cahill et al Technology Map 1999	policy planning	Therapeutics for arterial occlusion and myocardial infarction	4	Treatment and management of symptoms and disease	IX - circulation	Drugs (not prophylactic vaccines)
Cahill et al Technology Map 1999	Policy planning	Guidelines for adult disease preventing life style	8	Supporting the provision of care and/or increasing service efficiency	XXI - screening, rehab	Organisational programmes
Cahill et al Technology Map 1999	policy planning	Development of anti-cancer agents (gene level)	11	Treatment and management of symptoms and disease	II - cancers	Drugs (not prophylactic vaccines)
Cahill et al Technology Map 1999	policy planning	drugs preventing the occurrence of cancer	12	Health promotion and prevention of disease	II - cancers	Drugs (not prophylactic vaccines)
Cahill et al Technology Map 1999	policy planning	Effective means to prevent metastasis	15	Health promotion and prevention of disease	II - cancers	Unknown
Cahill et al Technology Map 1999	policy planning	Genetic/biochemical tests for schizophrenia and depression	9	Diagnosis and monitoring of disease	VI - nervous	Diagnostic tests and imaging

For peer review only - http://bmjopen.bmj.com/site/about/guidelines.xhtml

Appendix1: List of identified healthcare technologies

Food and Drug Administration (FDA) surveys 1998	Policy planning	Biosensors	10	Unknown	Other - generic	Diagnostic tests and imaging
Food and Drug Administration (FDA) surveys 1998	Policy planning	Laser diagnosis	10	Diagnosis and monitoring of disease	Other - generic	Diagnostic tests and imaging
Food and Drug Administration (FDA) surveys 1998	Policy planning	Tissue engineered devices	10	Treatment and management of symptoms and disease	Other - generic	Devices and biomaterials
Food and Drug Administration (FDA) surveys 1998	Policy planning	Blood vessel prosthetics	10	Treatment and management of symptoms and disease	IX - circulation	Regenerative medicine
Food and Drug Administration (FDA) surveys 1998	Policy planning	Bone prosthetics/ growth	10	Treatment and management of symptoms and disease	XIII - musculoskeletal	Regenerative medicine
Food and Drug Administration (FDA) surveys 1998	Policy planning	Cardiac stimulation	10	Treatment and management of symptoms and disease	IX - circulation	Unknown
Food and Drug Administration (FDA) surveys 1998	Policy planning	cartilage prosthetics	10	Treatment and management of symptoms and disease	XIII - musculoskeletal	Devices and biomaterials
Food and Drug Administration (FDA) surveys 1998	Policy planning	Computer aided clinical labs	10	Diagnosis and monitoring of disease	Other - generic	IT systems, eHealth and mHealth
Food and Drug Administration (FDA) surveys 1998	Policy planning	Computer aided diagnosis	10	Supporting the provision of care and/or increasing service efficiency	Other - generic	IT systems, eHealth and mHealth
Food and Drug Administration (FDA) surveys 1998	Policy planning	Telemedicine	10	Supporting patients receiving care (designed to be used by individuals)	Other - generic	IT systems, eHealth and mHealth
Food and Drug Administration (FDA) surveys 1998	Policy planning	Drug impregnated devices	10	Treatment and management of symptoms and disease	Other - generic	Devices and biomaterials
Food and Drug Administration (FDA) surveys 1998	Policy planning	Endoscopy	10	Diagnosis and monitoring of disease	Other - generic	Diagnostic tests and imaging
Food and Drug Administration	Policy planning	Hearing aids/Nonimplante	10	Rehabilitation	VIII - ear	Devices and biomaterials

For peer review only - http://bmjopen.bmj.com/site/about/guidelines.xhtml

Appendix1: List of identified healthcare technologies

(FDA) surveys 1998		d sensory aids				
Food and Drug Administration (FDA) surveys 1998	Policy planning	Heart valves	10	Treatment and management of symptoms and disease	IX - circulation	Devices and biomaterials
Food and Drug Administration (FDA) surveys 1998	Policy planning	Heart pumps	10	Treatment and management of symptoms and disease	IX - circulation	Devices and biomaterials
Food and Drug Administration (FDA) surveys 1998	Policy planning	Home diagnostics /Home/self monitoring & diagnosis	10	Supporting patients receiving care (designed to be used by individuals)	Other - generic	Organisational programmes
Food and Drug Administration (FDA) surveys 1998	Policy planning	Image contrast agents	10	Diagnosis and monitoring of disease	Other - generic	Diagnostic tests and imaging
Food and Drug Administration (FDA) surveys 1998	Policy planning	Integrated patient medical info systems	10	Supporting the provision of care and/or increasing service efficiency	Other - generic	IT systems, eHealth and mHealth
Food and Drug Administration (FDA) surveys 1998	Policy planning	Laser treatment	10	Treatment and management of symptoms and disease	Other - generic	Non-surgical therapy
Food and Drug Administration (FDA) surveys 1998	Policy planning	kidney prosthetics	10	Treatment and management of symptoms and disease	XIV - genitourina ry	Regenerative medicine
Food and Drug Administration (FDA) surveys 1998	Policy planning	Laser surgery	10	Treatment and management of symptoms and disease	Other - generic	Non-surgical therapy
Food and Drug Administration (FDA) surveys 1998	Policy planning	Liver prosthetics	10	Treatment and management of symptoms and disease	XI - GI tract, dental health	Regenerative medicine
Food and Drug Administration (FDA) surveys 1998	Policy planning	Mini invasive neurosurgery	10	Treatment and management of symptoms and disease	VI - nervous	Therapeutic procedures
Food and Drug Administration (FDA) surveys 1998	Policy planning	Mini invasive cardiac surgery	10	Treatment and management of symptoms and disease	IX - circulation	Therapeutic procedures
Food and Drug Administration (FDA) surveys 1998	Policy planning	MRI	10	Diagnosis and monitoring of disease	Other - generic	Diagnostic tests and imaging

For peer review only - http://bmjopen.bmj.com/site/about/guidelines.xhtml

Appendix1: List of identified healthcare technologies

Food and Drug Administration (FDA) surveys 1998	Policy planning	Neuromuscular stimulation	10	Treatment and management of symptoms and disease	Other - generic	Non-surgical therapy
Food and Drug Administration (FDA) surveys 1998	Policy planning	Nerve regeneration	10	Treatment and management of symptoms and disease	VI - nervous	Regenerative medicine
Food and Drug Administration (FDA) surveys 1998	Policy planning	Neural stimulation	10	Treatment and management of symptoms and disease	VI - nervous	Non-surgical therapy
Food and Drug Administration (FDA) surveys 1998	Policy planning	Ocular prosthetics	10	Treatment and management of symptoms and disease	VII - eye	Devices and biomaterials
Food and Drug Administration (FDA) surveys 1998	Policy planning	Pancreas prosthetics	10	Treatment and management of symptoms and disease	IV - endocrine	Regenerative medicine
Food and Drug Administration (FDA) surveys 1998	Policy planning	Patient smart cards	10	Supporting patients receiving care (designed to be used by individuals)	Other - generic	IT systems, eHealth and mHealth
Food and Drug Administration (FDA) surveys 1998	Policy planning	Robotic surgery	10	Treatment and management of symptoms and disease	Other - generic	Therapeutic procedures
Food and Drug Administration (FDA) surveys 1998	Policy planning	Skin prosthetics	10	Treatment and management of symptoms and disease	XII - skin	Regenerative medicine
Food and Drug Administration (FDA) surveys 1998	Policy planning	Telemedicine-home use	10	Supporting patients receiving care (designed to be used by individuals)	Other - generic	IT systems, eHealth and mHealth
Food and Drug Administration (FDA) surveys 1998	Policy planning	Telemedicine-radiology	10	Supporting patients receiving care (designed to be used by individuals)	Other - generic	IT systems, eHealth and mHealth
Food and Drug Administration (FDA) surveys 2008	Policy planning	Robotic surgical systems	0	Treatment and management of symptoms and disease	Other - generic	Therapeutic procedures
Food and Drug Administration (FDA) surveys 1998	Policy planning	Point-of-care products	10	Diagnosis and monitoring of disease	Other - generic	Diagnostic tests and imaging

For peer review only - http://bmjopen.bmj.com/site/about/guidelines.xhtml

Appendix1: List of identified healthcare technologies

Food and Drug Administration (FDA) surveys 1998	Policy planning	Computer-assisted diagnostic systems	10	Diagnosis and monitoring of disease	Other - generic	IT systems, eHealth and mHealth
Food and Drug Administration (FDA) surveys 1998	Policy planning	Neuro-sensory devices	10	Diagnosis and monitoring of disease	VI - nervous	Devices and biomaterials
Food and Drug Administration (FDA) surveys 1998	Policy planning	Optical diagnostic products	10	Diagnosis and monitoring of disease	Other - generic	Diagnostic tests and imaging
Food and Drug Administration (FDA) surveys 1998	Policy planning	Integrated electronic patient medical records	10	Supporting the provision of care and/or increasing service efficiency	Other - generic	IT systems, eHealth and mHealth
Food and Drug Administration (FDA) surveys 1998	Policy planning	Remote patient monitoring systems	10	Supporting patients receiving care (designed to be used by individuals)	Other - generic	IT systems, eHealth and mHealth
Food and Drug Administration (FDA) surveys 1998	Policy planning	Glucose monitoring products	10	Diagnosis and monitoring of disease	IV - endocrine	Diagnostic tests and imaging
Food and Drug Administration (FDA) surveys 1998	Policy planning	Tissue engineered products	10	Treatment and management of symptoms and disease	Other - generic	Regenerative medicine
Food and Drug Administration (FDA) surveys 1998	Policy planning	Advanced prosthetic limbs	10	Rehabilitation	XIII - musculoskeletal	Devices and biomaterials
Food and Drug Administration (FDA) surveys 1998	Policy planning	Minimally invasive implants (e.g., percutaneous or natural orifice delivery)	10	Treatment and management of symptoms and disease	Other - generic	Devices and biomaterials
Food and Drug Administration (FDA) surveys 1998	Policy planning	Virtual reality systems for immersive training and other applications	10	Unknown	Other - generic	IT systems, eHealth and mHealth
Food and Drug Administration (FDA) surveys 1998	Policy planning	Optical therapeutic devices	10	Treatment and management of symptoms and disease	Other - generic	Devices and biomaterials
Food and Drug Administration (FDA) surveys 1998	Policy planning	Internet-based medical device systems	10	Supporting patients receiving care (designed to be used by	Other - generic	IT systems, eHealth and mHealth

For peer review only - http://bmjopen.bmj.com/site/about/guidelines.xhtml

Appendix1: List of identified healthcare technologies

				individuals)		
Food and Drug Administration (FDA) surveys 1998	Policy planning	New (non-eluting) drug delivery systems	10	Treatment and management of symptoms and disease	Other - generic	Drugs (not prophylactic vaccines)
Food and Drug Administration (FDA) surveys 1998	Policy planning	Robotic prosthetics	20	Rehabilitation	Other - generic	Devices and biomaterials
Food and Drug Administration (FDA) surveys 1998	Policy planning	New types of insulin pumps and delivery systems	20	Treatment and management of symptoms and disease	IV - endocrine	Devices and biomaterials
Food and Drug Administration (FDA) surveys 1998	Policy planning	New types of stents	20	Treatment and management of symptoms and disease	IX - circulation	Devices and biomaterials
Food and Drug Administration (FDA) surveys 1998	Policy planning	Joint replacements	20	Treatment and management of symptoms and disease	XIII - musculoskeletal	Devices and biomaterials
Food and Drug Administration (FDA) surveys 1998	Policy planning	Image-guided therapy systems	20	Treatment and management of symptoms and disease	Other - generic	Therapeutic procedures
Food and Drug Administration (FDA) surveys 1998	Policy planning	Advanced ultrasound imaging systems	20	Diagnosis and monitoring of disease	Other - generic	Diagnostic tests and imaging
Food and Drug Administration (FDA) surveys 1998	Policy planning	Advanced optical imaging systems	20	Diagnosis and monitoring of disease	Other - generic	Diagnostic tests and imaging
Food and Drug Administration (FDA) surveys 1998	Policy planning	Advanced MR imaging systems	20	Diagnosis and monitoring of disease	Other - generic	Diagnostic tests and imaging
Food and Drug Administration (FDA) surveys 1998	Policy planning	Minimally invasive radiotherapy systems	20	Treatment and management of symptoms and disease	II - cancers	Non-surgical therapy
Food and Drug Administration (FDA) surveys 1998	Policy planning	Genetic diagnostic products	20	Diagnosis and monitoring of disease	Other - generic	Diagnostic tests and imaging
Food and Drug Administration (FDA) surveys 1998	Policy planning	Remote patient monitoring systems	20	Supporting patients receiving care (designed to be used by	Other - generic	IT systems, eHealth and mHealth

For peer review only - http://bmjopen.bmj.com/site/about/guidelines.xhtml

Appendix1: List of identified healthcare technologies

				individuals)		
Institute of the Future, 2009	Research	Bio-marker based tools will screen for future disease risk and produce new diagnoses based on probabilities	11	Diagnosis and monitoring of disease	Other - generic	Diagnostic tests and imaging
Institute of the Future, 2009	Research	Visualizations improve diet & exercise	11	Health promotion and prevention of disease	Other - generic	Non-surgical therapy
Institute of the Future, 2009	Research	GPS-enabled asthma inhalers	11	Supporting the provision of care and/or increasing service efficiency	X - respiratory	IT systems, eHealth and mHealth
Institute of the Future, 2009	Research	Workplace clinics	11	Health promotion and prevention of disease	Other - generic	Organisational programmes
Institute of the Future, 2009	Research	Urban planning for walkability, pollution reduction, neighbourood food systems	11	Health promotion and prevention of disease	Other - generic	Population programmes
Institute of the Future, 2009	Research	Report cards include Body Mass Index (BMI) scores	11	Health promotion and prevention of disease	Other - generic	Population programmes
Institute of the Future, 2009	Research	iPhone-synched medical devices	11	Supporting patients receiving care (designed to be used by individuals)	Other - generic	IT systems, eHealth and mHealth
Institute of the Future, 2009	Research	Quantifying, visualizing, and mapping health implications of environments	11	Health promotion and prevention of disease	Other - generic	Population programmes
Institute of the Future, 2009	Research	Deep brain stimulation used for neurological, behavioral issues	11	Treatment and management of symptoms and disease	VI - nervous	Devices and biomaterials
Institute of the Future, 2009	Research	Home sensors for elder care	11	Supporting patients receiving care (designed to be used by individuals)	Other - generic	IT systems, eHealth and mHealth
Institute of the Future, 2009	Research	Individually targeted therapies	11	Treatment and management of symptoms and	Other - generic	Drugs (not prophylactic vaccines)

For peer review only - http://bmjopen.bmj.com/site/about/guidelines.xhtml

Appendix1: List of identified healthcare technologies

				disease		
Institute of the Future, 2009	Research	Sensors track drug efficacy	11	Supporting patients receiving care (designed to be used by individuals)	Other - generic	Devices and biomaterials
Institute of the Future, 2009	Research	Genotypic diagnostics and risk	11	Diagnosis and monitoring of disease	Other - generic	Diagnostic tests and imaging
Institute of the Future, 2009	Research	New technologies enable individuals to experience the future effects of present-day behavior choices	11	Treatment and management of symptoms and disease	Other - generic	Unknown
Institute of the Future, 2009	Research	Regenerative medicine (general)will replace, restore, maintain, or enhance tissue and organ functions	11	Treatment and management of symptoms and disease	Other - generic	Regenerative medicine
Institute of the Future, 2009	Research	New ways of reliably measuring subjective states such as happiness, anxiety, or pain, and finding the neural correlates of personality and behavioral patterns	11	Diagnosis and monitoring of disease	VI - nervous	Diagnostic tests and imaging
Institute of the Future, 2009	Research	prototype of a prosthetic arm that can be controlled naturally and provide sensory feedback.	11	Rehabilitation	XIII - musculoskeletal	Devices and biomaterials
Stevens et al 1997	Research	Magnetic resonance imaging	1	Diagnosis and monitoring of disease	Other - generic	Diagnostic tests and imaging
Stevens et al 1997	Research	Minimally invasive surgery	1	Treatment and management of symptoms and disease	Other - generic	Therapeutic procedures
Stevens et al 1997	Research	drugs for Therapeutic refractory	1	Treatment and management of symptoms and	V - mental health	Drugs (not prophylactic vaccines)

For peer review only - http://bmjopen.bmj.com/site/about/guidelines.xhtml

Appendix1: List of identified healthcare technologies

		schizphrenia		disease		
Stevens et al 1997	Research	Implantable vascular stents	1	Treatment and management of symptoms and disease	IX - circulation	Devices and biomaterials
Stevens et al 1997	Research	Peripheral blood stem cells transplantation	1	Treatment and management of symptoms and disease	Other - generic	Regenerative medicine
Stevens et al 1997	Research	Laser Therapeutic of benign prostatic hyperplasia	1	Treatment and management of symptoms and disease	XIV - genitourinary	Non-surgical therapy
Stevens et al 1997	Research	Gene therapy advances	4	Treatment and management of symptoms and disease	Other - generic	Regenerative medicine
Stevens et al 1997	Research	Polymerase chain reaction	4	Diagnosis and monitoring of disease	Other - generic	Diagnostic tests and imaging
Stevens et al 1997	Research	Telemedicine	4	Supporting the provision of care and/or increasing service efficiency	Other - generic	IT systems, eHealth and mHealth
Stevens et al 1997	Research	Interventional radiology	1	Treatment and management of symptoms and disease	Other - generic	Non-surgical therapy
Stevens et al 1997	Research	Angioplasty	1	Treatment and management of symptoms and disease	IX - circulation	Therapeutic procedures
Stevens et al 1997	Research	Interferon for chronic granulocytic leukemia	1	Treatment and management of symptoms and disease	II - cancers	Drugs (not prophylactic vaccines)
Stevens et al 1997	Research	Interferon for hepatitis C	1	Treatment and management of symptoms and disease	I - infections	Drugs (not prophylactic vaccines)
Stevens et al 1997	Research	Lasers for dermatology	1	Treatment and management of symptoms and disease	XII - skin	Devices and biomaterials
Stevens et al 1997	Research	Adjuvant chemotherapy in lung cancer	4	Treatment and management of symptoms and disease	II - cancers	Drugs (not prophylactic vaccines)

For peer review only - http://bmjopen.bmj.com/site/about/guidelines.xhtml

Appendix1: List of identified healthcare technologies

Stevens et al 1997	Research	Near patient testing	2	Diagnosis and monitoring of disease	Other - generic	Diagnostic tests and imaging
Stevens et al 1997	Research	Revision of joint replacement	1	Treatment and management of symptoms and disease	XIII - musculoskeletal	Therapeutic procedures
Stevens et al 1997	Research	Genetic screening	4	Diagnosis and monitoring of disease	Other - generic	Diagnostic tests and imaging
Stevens et al 1997	Research	Helicobactor pylori eradication	1	Treatment and management of symptoms and disease	I - infections	Drugs (not prophylactic vaccines)
Stevens et al 1997	Research	Positron emission tomography	4	Diagnosis and monitoring of disease	Other - generic	Diagnostic tests and imaging
Stevens et al 1997	Research	Phacoemulsification	1	Treatment and management of symptoms and disease	VII - eye	Therapeutic procedures
Stevens et al 1997	Research	Cochlear implants	1	Rehabilitation	VIII - ear	Devices and biomaterials
Stevens et al 1997	Research	Paclitaxels for ovarian & breast cancer	2	Treatment and management of symptoms and disease	II - cancers	Drugs (not prophylactic vaccines)
Stevens et al 1997	Research	Bone densitometry screening	1	Diagnosis and monitoring of disease	XIII - musculoskeletal	Diagnostic tests and imaging
Stevens et al 1997	Research	Anticoagulants for atrial fibrillation	1	Health promotion and prevention of disease	IX - circulation	Drugs (not prophylactic vaccines)
Stevens et al 1997	Research	Picture archiving and communication system	4	Supporting the provision of care and/or increasing service efficiency	Other - generic	IT systems, eHealth and mHealth
Stevens et al 1997	Research	rhDNase	2	Treatment and management of symptoms and disease	IV - endocrine	Drugs (not prophylactic vaccines)
Stevens et al 1997	Research	Biomaterials in bone and joint replacement	2	Treatment and management of symptoms and disease	XIII - musculoskeletal	Devices and biomaterials
Stevens et al 1997	Research	Increased use of adjuvant chemotherapy in colorectal cancer	2	Treatment and management of symptoms and disease	II - cancers	Drugs (not prophylactic vaccines)
Stevens et al 1997	Research	Increased use of high purity factor	2	Treatment and management of	III - blood	Drugs (not prophylactic

For peer review only

Appendix1: List of identified healthcare technologies

		VIII and recombinant factor VIII in hemophilia		symptoms and disease		vaccines)
Stevens et al 1997	Research	Mirena intrauterine contraceptive device	2	Health promotion and prevention of disease	XIV - genitourinary	Drugs (not prophylactic vaccines)
Stevens et al 1997	Research	Adjuvant chemotherapy in lung cancer	4	Treatment and management of symptoms and disease	II - cancers	Drugs (not prophylactic vaccines)
Stevens et al 1997	Research	drugs for Alzheimer's disease	6	Treatment and management of symptoms and disease	V - mental health	Drugs (not prophylactic vaccines)
Stevens et al 1997	Research	Xenotransplantation	6	Treatment and management of symptoms and disease	Other - generic	Regenerative medicine
Stevens et al 1997	Research	Screening for abdominal aortic aneurysm	6	Health promotion and prevention of disease	IX - circulation	Population programmes
Stevens et al 1997	Research	Remotely controlled robots (laboratory)	6	Supporting the provision of care and/or increasing service efficiency	Other - generic	Medical equipment
Stevens et al 1997	Research	Gene therapy for cystic fibrosis	6	Treatment and management of symptoms and disease	IV - endocrine	Regenerative medicine
Stevens et al 1997	Research	New anesthetic vapors	2	Treatment and management of symptoms and disease	Other - generic	Drugs (not prophylactic vaccines)
Stevens et al 1997	Research	Magnetic resonance angiography	4	Diagnosis and monitoring of disease	IX - circulation	Diagnostic tests and imaging
Stevens et al 1997	Research	Digital radiography	4	Supporting the provision of care and/or increasing service efficiency	Other - generic	IT systems, eHealth and mHealth
Stevens et al 1997	Research	Alendronate for osteoporosis	2	Treatment and management of symptoms and disease	XIII - musculoskeletal	Drugs (not prophylactic vaccines)
Stevens et al 1997	Research	Continuous positive airways pressure	1	Treatment and management of symptoms and disease	X - respiratory	Non-surgical therapy

For peer review only

Appendix1: List of identified healthcare technologies

Stevens et al 1997	Research	Expanding metal stents for esophageal cancer	1	Treatment and management of symptoms and disease	II - cancers	Devices and biomaterials
Stevens et al 1997	Research	Community placements for severe mental illness	1	Supporting patients receiving care (designed to be used by individuals)	V - mental health	Organisational programmes
Stevens et al 1997	Research	Fludarabine in lymphomas and chronic leukemias	2	Treatment and management of symptoms and disease	II - cancers	Drugs (not prophylactic vaccines)
Stevens et al 1997	Research	Combined therapy for HIV/AIDS	2	Treatment and management of symptoms and disease	I - infections	Drugs (not prophylactic vaccines)
Stevens et al 1997	Research	Epilepsy surgery	1	Treatment and management of symptoms and disease	VI - nervous	Therapeutic procedures
Stevens et al 1997	Research	Lipid-lowering drugs for elevated cholesterol	1	Health promotion and prevention of disease	IX - circulation	Drugs (not prophylactic vaccines)
Stevens et al 1997	Research	Repair of abdominal aortic aneurysm with transfemoral endovasculate (bifurcated) graft	4	Treatment and management of symptoms and disease	IX - circulation	Devices and biomaterials
Stevens et al 1997	Research	Intracytoplasmatic sperm injection	2	Treatment and management of symptoms and disease	XIV - genitourinary	Therapeutic procedures
Stevens et al 1997	Research	Computed tomography (CT) scan advances	2	Diagnosis and monitoring of disease	Other - generic	Diagnostic tests and imaging
Stevens et al 1997	Research	Voice activated dictation technology	1	Supporting the provision of care and/or increasing service efficiency	Other - generic	Organisational programmes
Stevens et al 1997	Research	Intra-arterial metallic stents	1	Treatment and management of symptoms and disease	IX - circulation	Devices and biomaterials
Tremblay and Yiu, 2006	Policy planning	Hand-held robotic and remote ultrasound	14	Diagnosis and monitoring of disease	Other - generic	Diagnostic tests and imaging
Tremblay and Yiu, 2006	Policy planning	MRI guided catheterization	14	Diagnosis and monitoring of disease	IX - circulation	Diagnostic tests and imaging

For peer review only - http://bmjopen.bmj.com/site/about/guidelines.xhtml

Appendix1: List of identified healthcare technologies

Tremblay and Yiu, 2006	Policy planning	Image-guided therapy and surgery and robotics	14	Treatment and management of symptoms and disease	Other - generic	Therapeutic procedures
Tremblay and Yiu, 2006	Policy planning	Computed Tomographic Colonography	14	Diagnosis and monitoring of disease	XI - GI tract, dental health	Diagnostic tests and imaging
Tremblay and Yiu, 2006	Policy planning	Digital Tomosynthesis	14	Diagnosis and monitoring of disease	Other - generic	Diagnostic tests and imaging
Tremblay and Yiu, 2006	Policy planning	Molecular-level imaging	14	Diagnosis and monitoring of disease	Other - generic	Diagnostic tests and imaging
Tremblay and Yiu, 2006	Policy planning	More economical scanners with improved image processing	14	Supporting the provision of care and/or increasing service efficiency	Other - generic	Medical equipment
Tremblay and Yiu, 2006	Policy planning	4D imaging	14	Diagnosis and monitoring of disease	Other - generic	Diagnostic tests and imaging
Tremblay and Yiu, 2006	Policy planning	Faster cycle time to capture an image and general reduction in scan times	14	Supporting the provision of care and/or increasing service efficiency	Other - generic	Medical equipment
Tremblay and Yiu, 2006	Policy planning	Hybrid PET/CT increasingly replacing invasive procedures	14	Diagnosis and monitoring of disease	Other - generic	Diagnostic tests and imaging
Tremblay and Yiu, 2006	Policy planning	Stereotactic radio-surgery for early detection of coronary heart diseases	14	Diagnosis and monitoring of disease	IX - circulation	Diagnostic tests and imaging
Tremblay and Yiu, 2006	Policy planning	Magnetic Resonance Guided Focused Ultrasound for Imaging of inflammation	14	Diagnosis and monitoring of disease	Other - generic	Diagnostic tests and imaging
Tremblay and Yiu, 2006	Policy planning	Wireless home-health monitoring	14	Supporting patients receiving care (designed to be used by individuals)	Other - generic	IT systems, eHealth and mHealth
Tremblay and Yiu, 2006	Policy planning	E-health diagnostic platform	14	Supporting the provision of care and/or increasing service efficiency	Other - generic	IT systems, eHealth and mHealth
Tremblay and Yiu, 2006	Policy planning	Hand-held gamma camera	14	Diagnosis and monitoring of	Other - generic	Diagnostic tests and imaging

For peer review only - http://bmjopen.bmj.com/site/about/guidelines.xhtml

Appendix1: List of identified healthcare technologies

				disease		
Tremblay and Yiu, 2006	Policy planning	"one-stop" diagnosis and Therapeutic	14	Supporting the provision of care and/or increasing service efficiency	Other - generic	Organisational programmes
Tremblay and Yiu, 2006	Policy planning	customised hybrid systems for specific clinical situations	14	Unknown	Other - generic	Organisational programmes
Tremblay and Yiu, 2006	Policy planning	Near Infra-red Spectroscopy	14	Diagnosis and monitoring of disease	Other - generic	Diagnostic tests and imaging
Tremblay and Yiu, 2006	Policy planning	Spatially-resolved Spectroscopy	14	Diagnosis and monitoring of disease	Other - generic	Diagnostic tests and imaging
Tremblay and Yiu, 2006	Policy planning	Optical Coherence Tomography	14	Diagnosis and monitoring of disease	Other - generic	Diagnostic tests and imaging
Tremblay and Yiu, 2006	Policy planning	Capsule Endoscopy	14	Diagnosis and monitoring of disease	Other - generic	Devices and biomaterials
Tremblay and Yiu, 2006	Policy planning	Tele-echography	14	Supporting the provision of care and/or increasing service efficiency	Other - generic	IT systems, eHealth and mHealth
Tremblay and Yiu, 2006	Policy planning	Tele-colposcopy	14	Supporting the provision of care and/or increasing service efficiency	Other - generic	IT systems, eHealth and mHealth
Tremblay and Yiu, 2006	Policy planning	Electronic Nose	14	Diagnosis and monitoring of disease	Other - generic	Diagnostic tests and imaging
Tremblay and Yiu, 2006	Policy planning	New contrast agents for Rubidium	14	Diagnosis and monitoring of disease	Other - generic	Diagnostic tests and imaging
Tremblay and Yiu, 2006	Policy planning	Hyperpolarised gases	14	Diagnosis and monitoring of disease	Other - generic	Diagnostic tests and imaging
Tremblay and Yiu, 2006	Policy planning	Terahertz waves	14	Diagnosis and monitoring of disease	Other - generic	Diagnostic tests and imaging
Tremblay and Yiu, 2006	Policy planning	Microwaves, light spectrum	14	Unknown	Other - generic	Devices and biomaterials
Tremblay and Yiu, 2006	Policy planning	Hyperpolarised imaging	14	Diagnosis and monitoring of disease	Other - generic	Diagnostic tests and imaging
Tremblay and Yiu, 2006	Policy planning	Specific advances in MRI, Ultrasound, Radiography…etc	14	Diagnosis and monitoring of disease	Other - generic	Diagnostic tests and imaging

For peer review only - http://bmjopen.bmj.com/site/about/guidelines.xhtml

Appendix1: List of identified healthcare technologies

Tremblay and Yiu, 2006	Policy planning	Reduction in use of invasive diagnostic procedures	14	Supporting the provision of care and/or increasing service efficiency	Other - generic	Organisational programmes
Tremblay and Yiu, 2006	Policy planning	Diagnosis of kidney disorders without renal biopsy	14	Diagnosis and monitoring of disease	XIV - genitourina ry	Unknown
Tremblay and Yiu, 2006	Policy planning	Ex vivo molecular-computer diagnosis	14	Diagnosis and monitoring of disease	Other - generic	IT systems, eHealth and mHealth
Tremblay and Yiu, 2006	Policy planning	Smart-drug delivery systems	14	Treatment and management of symptoms and disease	Other - generic	Devices and biomaterials
Tremblay and Yiu, 2006	Policy planning	Polymer-encapsulated, bioconjugated quantum dots	9	Diagnosis and monitoring of disease	Other - generic	Diagnostic tests and imaging
Tremblay and Yiu, 2006	Policy planning	Patient accessed universal health records	4	Supporting the provision of care and/or increasing service efficiency	Other - generic	Organisational programmes
Tremblay and Yiu, 2006	Policy planning	In silico-in vivo integration (lab on-a-chip)	14	Diagnosis and monitoring of disease	Other - generic	Diagnostic tests and imaging
Tremblay and Yiu, 2006	Policy planning	Wide deployment of non-invasive technologies and biosensors	9	Supporting the provision of care and/or increasing service efficiency	Other - generic	Devices and biomaterials
Tremblay and Yiu, 2006	Policy planning	Direct consumer access to diagnostic tests	6	Supporting patients receiving care (designed to be used by individuals)	Other - generic	Organisational programmes
Tremblay and Yiu, 2006	Policy planning	Molecular imaging with single molecular pecision	9	Diagnosis and monitoring of disease	Other - generic	Diagnostic tests and imaging
Tremblay and Yiu, 2006	Policy planning	diagnostic testing throughout the care pathway	7	Supporting the provision of care and/or increasing service efficiency	Other - generic	Diagnostic tests and imaging
Tremblay and Yiu, 2006	Policy planning	Neuro-imaging at cellular level of brain processes (thoughts)	14	Diagnosis and monitoring of disease	VI - nervous	Diagnostic tests and imaging
Tremblay and Yiu, 2006	Policy planning	Molecular-computers used to design smart therapies	14	Other	Other - generic	Drugs (not prophylactic vaccines)

For peer review only - http://bmjopen.bmj.com/site/about/guidelines.xhtml

Appendix1: List of identified healthcare technologies

Tremblay and Yiu, 2006	Policy planning	Remote internet diagnosis based on data received from patient's home	9	Supporting patients receiving care (designed to be used by individuals)	Other - generic	IT systems, eHealth and mHealth
Tremblay and Yiu, 2006	Policy planning	Molecular diagnosis	14	Diagnosis and monitoring of disease	Other - generic	Diagnostic tests and imaging
Tremblay and Yiu, 2006	Policy planning	diagnostic -guided therapy for individualised medicine	14	Treatment and management of symptoms and disease	Other - generic	Drugs (not prophylactic vaccines)
Tremblay and Yiu, 2006	Policy planning	Computer-assisted diagnosis with pattern recognition	14	Diagnosis and monitoring of disease	Other - generic	IT systems, eHealth and mHealth
Tremblay and Yiu, 2006	Policy planning	Self-propelled micro-machines for diagnosis and Therapeutic	9	Unknown	Other - generic	Devices and biomaterials
Tremblay and Yiu, 2006	Policy planning	diagnostic bio-chip for cancer	9	Diagnosis and monitoring of disease	II - cancers	Diagnostic tests and imaging
Tremblay and Yiu, 2006	Policy planning	Investigation of thrombosis and vascular pathology using 3D tomography with remote controlled micro-instruments	14	Diagnosis and monitoring of disease	IX - circulation	Devices and biomaterials
Tremblay and Yiu, 2006	Policy planning	Personal care nurse-robots	12	Supporting patients receiving care (designed to be used by individuals)	Other - generic	Medical equipment
Tremblay and Yiu, 2006	Policy planning	Functional combination of targeting and imaging	14	Diagnosis and monitoring of disease	Other - generic	Diagnostic tests and imaging
Tremblay and Yiu, 2006	Policy planning	Intelligent and context sensitive clinical systems	14	Supporting the provision of care and/or increasing service efficiency	Other - generic	Organisational programmes
Tremblay and Yiu, 2006	Policy planning	Personal access to genomic information	14	Supporting patients receiving care (designed to be used by individuals)	Other - generic	Unknown
Tremblay and Yiu, 2006	Policy planning	Development of effective gene	9	Treatment and management of	Other - generic	Regenerative medicine

For peer review only - http://bmjopen.bmj.com/site/about/guidelines.xhtml

Appendix1: List of identified healthcare technologies

		therapies		symptoms and disease		
Science & Technology Foresight survey Japan 1997	Policy planning	Widespread use of scientific guidelines for adult-disease-preventing lifestyles (nutrition, rest and exercise)	9	Health promotion and prevention of disease	Other - generic	Organisational programmes
Science & Technology Foresight survey Japan 1997	Policy planning	Practical use of chemotherapy that brings complete remission to digestive organ cancer with low drug-responsiveness	18	Treatment and management of symptoms and disease	II - cancers	Drugs (not prophylactic vaccines)
Science & Technology Foresight survey Japan 1997	Policy planning	Widespread use of techniques to overcome the drug resistance of malignant tumours	16	Treatment and management of symptoms and disease	II - cancers	Drugs (not prophylactic vaccines)
Science & Technology Foresight survey Japan 1997	Policy planning	Widespread use of drugs that cure viral liver disease	13	Treatment and management of symptoms and disease	XI - GI tract, dental health	Drugs (not prophylactic vaccines)
Science & Technology Foresight survey Japan 1997	Policy planning	Widespread use of biological and immunological therapy effective for cancer	14	Treatment and management of symptoms and disease	II - cancers	Drugs (not prophylactic vaccines)
Science & Technology Foresight survey Japan 1997	Policy planning	Development of effective methods of preventing Alzheimer's disease	16	Health promotion and prevention of disease	V - mental health	Unknown
Science & Technology Foresight survey Japan 1997	Policy planning	Possible to cure allergic diseases	21	Treatment and management of symptoms and disease	III - blood	Unknown
Science & Technology Foresight survey Japan 1997	Policy planning	Development of an HIV vaccine	10	Health promotion and prevention of disease	I - infections	Prophylactic vaccines
Science & Technology Foresight survey Japan 1997	Policy planning	Widespread use of gene therapy against malignant tumors	17	Treatment and management of symptoms and disease	II - cancers	Regenerative medicine

For peer review only - http://bmjopen.bmj.com/site/about/guidelines.xhtml

Appendix1: List of identified healthcare technologies

Science & Technology Foresight survey Japan 1997	Policy planning	Widespread use of prevention methods for the contraction of diabetic complications	11	Health promotion and prevention of disease	IV - endocrine	Unknown
Science & Technology Foresight survey Japan 1997	Policy planning	Practical use of anti-AIDS therapy	12	Treatment and management of symptoms and disease	I - infections	Drugs (not prophylactic vaccines)
Science & Technology Foresight survey Japan 1997	Policy planning	Widespread use of an early cancer diagnosis technique based on biochemical examination	10	Diagnosis and monitoring of disease	II - cancers	Diagnostic tests and imaging
Science & Technology Foresight survey Japan 1997	Policy planning	Widespread use of a technique to eliminate viruses from blood	12	Treatment and management of symptoms and disease	I - infections	Therapeutic procedures
Science & Technology Foresight survey Japan 1997	Policy planning	Practical use of prevention methods for stress-induced mental disorders	14	Health promotion and prevention of disease	V - mental health	Unknown
Science & Technology Foresight survey Japan 1997	Policy planning	Practical use of a method to quantitatively assess the level of aging (biological age) in relation to chronological age.	9	Diagnosis and monitoring of disease	Other - generic	Unknown
Science & Technology Foresight survey Japan 1997	Policy planning	Practical use of a preventive measure against diabetic complications.	14	Health promotion and prevention of disease	IV - endocrine	Unknown
Science & Technology Foresight survey Japan 1997	Policy planning	Widespread use of presbyopia prevention methods	29	Health promotion and prevention of disease	VII - eye	Unknown
Science & Technology Foresight survey Japan 1997	Policy planning	Widespread use of a laser-applied tooth decay treatment method	14	Treatment and management of symptoms and disease	XI - GI tract, dental health	Devices and biomaterials
Science & Technology Foresight survey Japan 1997	Policy planning	Widespread method of prevention against a premature delivery through	8	Health promotion and prevention of disease	XV - pregnancy	Organisational programmes

For peer review only

Appendix1: List of identified healthcare technologies

		the progression of management system of fetal information.				
Science & Technology Foresight survey Japan 1997	Policy planning	Practical use of the prevention against congenital anomaly originating in the embryonic or fetal period.	19	Health promotion and prevention of disease	XV - pregnancy	Unknown
Science & Technology Foresight survey Japan 1997	Policy planning	Widespread use of preventive measure for cerebral palsy	19	Health promotion and prevention of disease	VI - nervous	Unknown
Science & Technology Foresight survey Japan 1997	Policy planning	Widespread use of a cancer spread diagnosis technique based on 3-D imaging.	7	Diagnosis and monitoring of disease	II - cancers	Diagnostic tests and imaging
Science & Technology Foresight survey Japan 1997	Policy planning	Widespread use of a cancer risk assessment technique based on genetic analysis.	11	Diagnosis and monitoring of disease	II - cancers	Unknown
Science & Technology Foresight survey Japan 1997	Policy planning	Widespread use of against of cancer cells drug sensitivity measurement method.	9	Unknown	II - cancers	Unknown
Science & Technology Foresight survey Japan 1997	Policy planning	Widespread use of automatic testing equipment for cancer cytodiagnosis.	9	Diagnosis and monitoring of disease	II - cancers	Diagnostic tests and imaging
Science & Technology Foresight survey Japan 1997	Policy planning	Practical use of classification and stage determination of schizophrenia based on diagnostic imaging.	24	Diagnosis and monitoring of disease	V - mental health	Diagnostic tests and imaging
Science & Technology Foresight survey Japan 1997	Policy planning	Practical use of diagnosing methods for determining the level and spread of arteriosclerosis	9	Diagnosis and monitoring of disease	IX - circulation	Diagnostic tests and imaging

For peer review only - http://bmjopen.bmj.com/site/about/guidelines.xhtml

Appendix1: List of identified healthcare technologies

		focused by a non-invasive process				
Science & Technology Foresight survey Japan 1997	Policy planning	Establishment of a correct diagnostic measure for kidney diseases without renal biopsy.	19	Diagnosis and monitoring of disease	XIV - genitourinary	Diagnostic tests and imaging
Science & Technology Foresight survey Japan 1997	Policy planning	Development of a capsule-type total gastro-intestinal tract monitoring system.	6	Diagnosis and monitoring of disease	XI - GI tract, dental health	Devices and biomaterials
Science & Technology Foresight survey Japan 1997	Policy planning	Practical use of specialist-level medical diagnosis assistance systems.	9	Supporting the provision of care and/or increasing service efficiency	Other - generic	IT systems, eHealth and mHealth
Science & Technology Foresight survey Japan 1997	Policy planning	Development of non-invasive cell-level diagnostic imaging.	8	Diagnosis and monitoring of disease	Other - generic	Diagnostic tests and imaging
Science & Technology Foresight survey Japan 1997	Policy planning	Practical use of a non-invasive technique to obtain blood biochemical examination data using body surface sensors.	9	Diagnosis and monitoring of disease	III - blood	Devices and biomaterials
Science & Technology Foresight survey Japan 1997	Policy planning	Practical use of a rapid diagnostic method for rejection in organ and tissue transplantation.	6	Diagnosis and monitoring of disease	III - blood	Unknown
Science & Technology Foresight survey Japan 1997	Policy planning	Widespread use of malaria vaccines.	10	Health promotion and prevention of disease	I - infections	Prophylactic vaccines
Science & Technology Foresight survey Japan 1997	Policy planning	Development of an effective insulin that can be administered orally.	16	Treatment and management of symptoms and disease	IV - endocrine	Drugs (not prophylactic vaccines)
Science & Technology Foresight survey Japan 1997	Policy planning	Practical use of gene therapy for diabetes.	16	Treatment and management of symptoms and disease	IV - endocrine	Regenerative medicine

For peer review only - http://bmjopen.bmj.com/site/about/guidelines.xhtml

Appendix1: List of identified healthcare technologies

Science & Technology Foresight survey Japan 1997	Policy planning	Widespread use of gene therapy for familial hypercholesterolemia.	14	Treatment and management of symptoms and disease	IX - circulation	Regenerative medicine
Science & Technology Foresight survey Japan 1997	Policy planning	Development of radiosensitizers effective in cancer treatment.	8	Treatment and management of symptoms and disease	II - cancers	Therapeutic procedures
Science & Technology Foresight survey Japan 1997	Policy planning	Widespread use of heavy particle against cancer	14	Treatment and management of symptoms and disease	II - cancers	Non-surgical therapy
Science & Technology Foresight survey Japan 1997	Policy planning	Development of a technique to cause drug delivery to targeted sites in the brain.	19	Treatment and management of symptoms and disease	Other - generic	Drugs (not prophylactic vaccines)
Science & Technology Foresight survey Japan 1997	Policy planning	Practical use of a safe pain control method without side effects	12	Treatment and management of symptoms and disease	Other - generic	Unknown
Science & Technology Foresight survey Japan 1997	Policy planning	Widespread use of gene therapy for muscular dystrophy.	24	Treatment and management of symptoms and disease	VI - nervous	Regenerative medicine
Science & Technology Foresight survey Japan 1997	Policy planning	Development of effective treatment for amyotrophic lateral sclerosis.	31	Treatment and management of symptoms and disease	VI - nervous	Unknown
Science & Technology Foresight survey Japan 1997	Policy planning	Practical use of artificial muscles for limbs.	21	Rehabilitation	XXI - screening, rehab	Devices and biomaterials
Science & Technology Foresight survey Japan 1997	Policy planning	Practical use of a technique to induce the differentiation of fibroblasts into cardiac muscle and its application to the treatment of myocardial infarction.	20	Treatment and management of symptoms and disease	IX - circulation	Regenerative medicine
Science & Technology Foresight survey Japan 1997	Policy planning	Development of hybrid-type artificial endocrine organs in which living	11	Treatment and management of symptoms and disease	IV - endocrine	Regenerative medicine

For peer review only

Appendix1: List of identified healthcare technologies

		cells and artificial objects coexist.				
Science & Technology Foresight survey Japan 1997	Policy planning	Widespread use of functional blood purifiers that selectively remove specified components from blood	19	Treatment and management of symptoms and disease	III - blood	Devices and biomaterials
Science & Technology Foresight survey Japan 1997	Policy planning	Widespread use of artificial hemoglobin as a red blood cell substitute.	15	Treatment and management of symptoms and disease	III - blood	Regenerative medicine
Science & Technology Foresight survey Japan 1997	Policy planning	Development of fully implanted artificial heart	14	Treatment and management of symptoms and disease	IX - circulation	Devices and biomaterials
Science & Technology Foresight survey Japan 1997	Policy planning	Practical use of fully implanted artificial lungs.	29	Treatment and management of symptoms and disease	X - respiratory	Devices and biomaterials
Science & Technology Foresight survey Japan 1997	Policy planning	Practical use of fully implanted artificial kidneys	26	Treatment and management of symptoms and disease	XIV - genitourina ry	Devices and biomaterials
Science & Technology Foresight survey Japan 1997	Policy planning	Practical use of fully implanted artificial pancreas	12	Treatment and management of symptoms and disease	XI - GI tract, dental health	Devices and biomaterials
Science & Technology Foresight survey Japan 1997	Policy planning	Widespread use of prevention and treatment methods for tooth decay and periodontitis to ensure the retention of 20 or more teeth at 80 year of age	14	Health promotion and prevention of disease	XI - GI tract, dental health	Population programmes
Science & Technology Foresight survey Japan 1997	Policy planning	Widespread use of a technique to store, cultivate and transplant one's own tissues.	15	Treatment and management of symptoms and disease	Other - generic	Regenerative medicine
Science & Technology Foresight survey Japan 1997	Policy planning	Practical use of implanted bladder control devices	12	Treatment and management of symptoms and disease	XIV - genitourina ry	Devices and biomaterials

For peer review only - http://bmjopen.bmj.com/site/about/guidelines.xhtml

Appendix1: List of identified healthcare technologies

Science & Technology Foresight survey Japan 1997	Policy planning	Practical use of gene therapy for genetic disorders	19	Treatment and management of symptoms and disease	Other - generic	Regenerative medicine
Science & Technology Foresight survey Japan 1997	Policy planning	Availability of genetherapy based on oral delivery	24	Treatment and management of symptoms and disease	Other - generic	Regenerative medicine
Science & Technology Foresight survey Japan 1997	Policy planning	Practical use of heterogeneous organ transplantation as means of treatment.	21	Treatment and management of symptoms and disease	Other - generic	Therapeutic procedures
Science & Technology Foresight survey Japan 1997	Policy planning	Widespread use of a worldwide organ supply system	12	Supporting the provision of care and/or increasing service efficiency	Other - generic	Organisational programmes
Science & Technology Foresight survey Japan 1997	Policy planning	Development of a partial tissue destruction method non-invasive to the living body.	6	Treatment and management of symptoms and disease	Other - generic	Non-surgical therapy
Science & Technology Foresight survey Japan 1997	Policy planning	Practical use of implanted artificial cochleas effective for both conductive hearing loss and perceptive hearing loss.	14	Rehabilitation	VIII - ear	Devices and biomaterials
Science & Technology Foresight survey Japan 1997	Policy planning	Development of a device to sense the needs of demented aged people.	21	Supporting patients receiving care (designed to be used by individuals)	V - mental health	Devices and biomaterials
Science & Technology Foresight survey Japan 1997	Policy planning	Development of controlling devices which help the cooperative muscular actions enabling object-oriented movement.	14	Rehabilitation	XXI - screening, rehab	Devices and biomaterials
Science & Technology Foresight survey Japan 1997	Policy planning	Widespread use of artificial legs featuring a small power source and computer control.	11	Rehabilitation	XXI - screening, rehab	Devices and biomaterials

For peer review only - http://bmjopen.bmj.com/site/about/guidelines.xhtml

Appendix1: List of identified healthcare technologies

Science & Technology Foresight survey Japan 1997	Policy planning	Practical use of a blood cell production and supply technique based on industrial-scale cultivation.	15	Treatment and management of symptoms and disease	III - blood	Regenerative medicine
Science & Technology Foresight survey Japan 1997	Policy planning	Practical use of systems for monitoring health condition and providing information for an appropriate diagnosis at home in event of accident or diseases.	11	Supporting patients receiving care (designed to be used by individuals)	XIX - injury	IT systems, eHealth and mHealth
Science & Technology Foresight survey Japan 1997	Policy planning	Widespread use of portable conversational speech interpretation systems.	24	Unknown	Other - generic	IT systems, eHealth and mHealth
Science & Technology Foresight survey Japan 1997	Policy planning	Widespread use of robots that care for people with severe physical and mental disabilities.	12	Supporting patients receiving care (designed to be used by individuals)	Other - generic	Medical equipment
Science & Technology Foresight survey Japan 1997	Policy planning	Advancements in patient and hospital information management, leading to a reduction in waiting time at Japanese hospitals to 15 minutes or less.	7	Supporting the provision of care and/or increasing service efficiency	Other - generic	Organisational programmes
Science & Technology Foresight survey Japan 1997	Policy planning	Practical use in Japan of an ID card system that thoroughly covers an individual's health conditions and medical data.	9	Supporting the provision of care and/or increasing service efficiency	Other - generic	Organisational programmes
Science & Technology Foresight survey	Policy planning	Elucidation of the biological clock, leading to drug	9	Supporting patients receiving care (designed to	Other - generic	Unknown

For peer review only

Appendix1: List of identified healthcare technologies

Japan 1997		administration that takes it into consideration		be used by individuals)		
Science & Technology Foresight survey Japan 1997	Policy planning	Practical application of the production of artificial cells with organ characteristics.	29	Treatment and management of symptoms and disease	Other - generic	Regenerative medicine
Science & Technology Foresight survey Japan 1997	Policy planning	Practical use of anti-AIDS therapy	12	Treatment and management of symptoms and disease	I - infections	Drugs (not prophylactic vaccines)
Science & Technology Foresight survey Japan 1997	Policy planning	Practical use of a rapid diagnostic method for rejection in organ and tissue transplantation	10	Diagnosis and monitoring of disease	Other - generic	Diagnostic tests and imaging
Science & Technology Foresight survey Japan 1997	Policy planning	Development of anti-cancer agents which target the manifestation functions of cancer genes	10	Treatment and management of symptoms and disease	II - cancers	Drugs (not prophylactic vaccines)
Science & Technology Foresight survey Japan 1997	Policy planning	Utilization of information about the gene structure of each individual patient in diagnosis and treatment	16	Diagnosis and monitoring of disease	Other - generic	Organisational programmes
Science & Technology Foresight survey Japan 1997	Policy planning	Development of technology to regenerate organs or individuals from separated animal cells	20	Treatment and management of symptoms and disease	Other - generic	Regenerative medicine
Science & Technology Foresight survey Japan 1997	Policy planning	Practical use of methods for directly determining DNA sequences by physical means such as X-rays.	14	Diagnosis and monitoring of disease	Other - generic	Diagnostic tests and imaging
Science & Technology Foresight survey Japan 1997	Policy planning	Control of signal transduction in the carcinogenesis of cells, and widespread use of treatment	20	Treatment and management of symptoms and disease	II - cancers	Drugs (not prophylactic vaccines)

For peer review only - http://bmjopen.bmj.com/site/about/guidelines.xhtml

Appendix1: List of identified healthcare technologies

		methods for dysdifferentiating carcinogenic cells.				
Science & Technology Foresight survey Japan 1997	Policy planning	Complete understanding of the factors contributing to stem cell multiplication, and widespread use of the practice of multiplying stem cells, as necessary, in test tubes and using them for treatment purpose	15	Treatment and management of symptoms and disease	Other - generic	Regenerative medicine
Science & Technology Foresight survey Japan 1997	Policy planning	Development of technology capable of synthesizing living cells by using only artificially produced chemical compounds.	31	Treatment and management of symptoms and disease	Other - generic	Regenerative medicine
Science & Technology Foresight survey Japan 1997	Policy planning	Practical use of elementary particle beam technology in genetic engineering.	19	Treatment and management of symptoms and disease	Other - generic	Regenerative medicine
Science & Technology Foresight survey Japan 1997	Policy planning	Development of drugs capable of preventing the occurrence of certain types of cancer.	11	Health promotion and prevention of disease	II - cancers	Drugs (not prophylactic vaccines)
Science & Technology Foresight survey Japan 1997	Policy planning	Practical use of effective means to prevent metastasis of cancer.	14	Treatment and management of symptoms and disease	II - cancers	Unknown
Science & Technology Foresight survey Japan 1997	Policy planning	Widespread use of technologies for long-term (semi-permanent) culturing and preservation of	19	Treatment and management of symptoms and disease	Other - generic	Regenerative medicine

For peer review only - http://bmjopen.bmj.com/site/about/guidelines.xhtml

Appendix1: List of identified healthcare technologies

		organs.				
Science & Technology Foresight survey Japan 1997	Policy planning	Practical use of artificial organs (pancreases, kidneys, livers, etc.) incorporating human cells and tissues	14	Treatment and management of symptoms and disease	Other - generic	Regenerative medicine
Science & Technology Foresight survey Japan 1997	Policy planning	Development of an entirely implantable artificial kidney.	18	Treatment and management of symptoms and disease	XIV - genitourinary	Regenerative medicine
Science & Technology Foresight survey Japan 1997	Policy planning	Development of an artificial cornea, allowing the visually impaired to regain their sight.	25	Treatment and management of symptoms and disease	VII - eye	Devices and biomaterials
Science & Technology Foresight survey Japan 1997	Policy planning	Development of artificial muscle elements.	21	Treatment and management of symptoms and disease	Other - generic	Regenerative medicine
Science & Technology Foresight survey Japan 1997	Policy planning	Establishment of interface technology between neural information and artificial organism structures.	25	Treatment and management of symptoms and disease	VI - nervous	Medical equipment
Science & Technology Foresight survey Japan 1997	Policy planning	Development of neural-computers with new logic structures modeled on brain functions.	24	Unknown	VI - nervous	IT systems, eHealth and mHealth
Science & Technology Foresight survey Japan 1997	Policy planning	Development of interfaces enabling direct linkage between the computer and the brain.	21	Treatment and management of symptoms and disease	XXI - screening, rehab	Devices and biomaterials
Science & Technology Foresight survey Japan 1997	Policy planning	Development of electric circuits with similar self-organization and self-restoration functions as neural networks.	14	Treatment and management of symptoms and disease	VI - nervous	Devices and biomaterials

For peer review only - http://bmjopen.bmj.com/site/about/guidelines.xhtml

Appendix1: List of identified healthcare technologies

Science & Technology Foresight survey Japan 1997	Policy planning	Development of diagnostic and medical treatment micromachines capable of traveling on their own inside organisms (body cavity organs).	24	Diagnosis and monitoring of disease	Other - generic	Devices and biomaterials
Science & Technology Foresight survey Japan 1997	Policy planning	Widespread use of remote surgery systems utilizing virtual reality technology.	14	Supporting the provision of care and/or increasing service efficiency	Other - generic	IT systems, eHealth and mHealth
Science & Technology Foresight survey Japan 1997	Policy planning	Widespread use of vicarious experience devices (using virtual reality technology) which allow bed-ridden patients to take vacations, etc.	9	Rehabilitation	XXI - screening, rehab	IT systems, eHealth and mHealth
Science & Technology Foresight survey Japan 1997	Policy planning	Widespread use of methods for controlling the immune system locally	14	Treatment and management of symptoms and disease	III - blood	Unknown
Science & Technology Foresight survey Japan 1997	Policy planning	Development of effective Therapeutic for amyotrophic lateral sclerosis	21	Treatment and management of symptoms and disease	VI - nervous	Drugs (not prophylactic vaccines)
Science & Technology Foresight survey Japan 1997	Policy planning	Practical use of batteries of artificial organs implanted in the living body	17	Treatment and management of symptoms and disease	Other - generic	Devices and biomaterials
Science & Technology Foresight survey Japan 1997	Policy planning	Development of artificial liver (external devices supporting liver functions) usable on a long-term, continuous basis	19	Treatment and management of symptoms and disease	XI - GI tract, dental health	Devices and biomaterials
Science & Technology Foresight survey Japan 1997	Policy planning	Practical use of heterogeneous organ transplantation as means of Therapeutic	19	Treatment and management of symptoms and disease	Other - generic	Therapeutic procedures

For peer review only - http://bmjopen.bmj.com/site/about/guidelines.xhtml

Appendix1: List of identified healthcare technologies

Science & Technology Foresight survey Japan 1997	Policy planning	Development of artificial eyes featuring electronic circuitry that can be connected to nerve and brain cells	24	Rehabilitation	VII - eye	Devices and biomaterials
Science & Technology Foresight survey Japan 1997	Policy planning	Development of methods for recombining disconnected central nerves	29	Rehabilitation	VI - nervous	Therapeutic procedures
Loveridge et al 1998	Policy planning	Practical use of directly visualising molecular structure at an atomic level (e.g. ultramicroscopy)	21	Diagnosis and monitoring of disease	Other - generic	Diagnostic tests and imaging
Loveridge et al 1998	Policy planning	At least 3 common diseases will be treated by portable or implanted systems that combine individually tailored drug delivery with automatic feedback control from a devices continuously monitoring the condition (e.g. diabetes control)	21	Treatment and management of symptoms and disease	Other - generic	Devices and biomaterials
Loveridge et al 1998	Policy planning	Over-the-counter nucleicacid-based tests suitable for self-administration become available for the first time	21	Supporting patients receiving care (designed to be used by individuals)	Other - generic	IT systems, eHealth and mHealth
Loveridge et al 1998	Policy planning	Results of more than 50% of all types of diagnostic tests are available at the point of need within one hour	21	Diagnosis and monitoring of disease	Other - generic	Diagnostic tests and imaging

For peer review only - http://bmjopen.bmj.com/site/about/guidelines.xhtml

Appendix1: List of identified healthcare technologies

Loveridge et al 1998	Policy planning	Widespread use of real time 3-D high resolutiom imaging of physiological and metabolic functions	21	Diagnosis and monitoring of disease	Other - generic	Diagnostic tests and imaging
Loveridge et al 1998	Policy planning	First practical use of non-invasive spectroscopy for in situ chemical pathology	21	Diagnosis and monitoring of disease	Other - generic	Diagnostic tests and imaging
Loveridge et al 1998	Policy planning	Practical use of biosensors in a wide range of medical manufacturing and environmental applications	21	Unknown	Other - generic	Devices and biomaterials
Loveridge et al 1998	Policy planning	All common single genes diseases can be treated successfully by gene theraby	21	Treatment and management of symptoms and disease	Other - generic	Drugs (not prophylactic vaccines)
Loveridge et al 1998	Policy planning	Practical use of gene therapy is extended to the treatment of 30% of major life threatening diseases	21	Treatment and management of symptoms and disease	Other - generic	Drugs (not prophylactic vaccines)
Loveridge et al 1998	Policy planning	Nucleic acid based diagnostic techniques account for 10% of all diagnostic e work load	21	Diagnosis and monitoring of disease	Other - generic	Organisational programmes
Loveridge et al 1998	Policy planning	First practical use of therapies based on purpose·designed non-peptide molecules which mimic the activity of peptides.	21	Treatment and management of symptoms and disease	Other - generic	Drugs (not prophylactic vaccines)
Loveridge et al 1998	Policy planning	Widespread use of polypeptide drugs which are delivered orally and targeted to	21	Treatment and management of symptoms and disease	Other - generic	Drugs (not prophylactic vaccines)

For peer review only - http://bmjopen.bmj.com/site/about/guidelines.xhtml

Appendix1: List of identified healthcare technologies

		specific tissues				
Loveridge et al 1998	Policy planning	Widespread use of cosmaceuticals with scientifically proven effectiveness to treat the sypmtoms of skin ageing (e.g. wrinkles)	21	Health promotion and prevention of disease	XII - skin	Drugs (not prophylactic vaccines)
Loveridge et al 1998	Policy planning	Widespread use of improved technologies for early diagnosis increases the 5-year survival rate of cancer patients by 50%	21	Diagnosis and monitoring of disease	II - cancers	Diagnostic tests and imaging
Loveridge et al 1998	Policy planning	Practical use of drugs modulating cellular systems to treat cancer	21	Treatment and management of symptoms and disease	II - cancers	Drugs (not prophylactic vaccines)
Loveridge et al 1998	Policy planning	Widespread use of nutritional supplements or special diets based on an understading of the role of nutrients such as anti-oxidants in cancer prevention	21	Health promotion and prevention of disease	II - cancers	Population programmes
Loveridge et al 1998	Policy planning	Practical use of substances that contain rather eleminate cancer (e.g. use agents that control metastasis)	21	Treatment and management of symptoms and disease	II - cancers	Drugs (not prophylactic vaccines)
Loveridge et al 1998	Policy planning	New methods of treating arterial occlusion and myocardial infarction dramatically reduce morbidity from cardiovascular diseases in 40-50 age group	21	Treatment and management of symptoms and disease	IX - circulation	Unknown

For peer review only - http://bmjopen.bmj.com/site/about/guidelines.xhtml

Appendix1: List of identified healthcare technologies

Loveridge et al 1998	Policy planning	50% of the UK population receive individual risk assessment for cardiovascular disease based on genetic screening and lifestyle measures	21	Health promotion and prevention of disease	IX - circulation	Population programmes
Loveridge et al 1998	Policy planning	Understanding the immune system is sufficietly developed to allow routine transplantation without the use of immuno-suupression	21	Treatment and management of symptoms and disease	III - blood	Therapeutic procedures
Loveridge et al 1998	Policy planning	Practical use of xenotransplantation of organs from animals to human	21	Treatment and management of symptoms and disease	Other - generic	Regenerative medicine
Loveridge et al 1998	Policy planning	Incidence of each of the 3 major viral diseases is reduced by 90% by new vaccines or drug development	21	Health promotion and prevention of disease	I - infections	Prophylactic vaccines
Loveridge et al 1998	Policy planning	Practical use of technologies for oral administration of multi-valent vaccines for the most important childhood illnesses	21	Health promotion and prevention of disease	I - infections	Prophylactic vaccines
Loveridge et al 1998	Policy planning	Widespread introduction of malaria vaccine reduces the worldwide incidence of the diseases by 50%	21	Health promotion and prevention of disease	I - infections	Prophylactic vaccines
Loveridge et al 1998	Policy planning	First effective vaccine of HIV becomes available	21	Health promotion and prevention of disease	I - infections	Prophylactic vaccines
Loveridge et al 1998	Policy planning	Development of drugs with proven	21	Treatment and management of	V - mental health	Drugs (not prophylactic

For peer review only

For peer review only - http://bmjopen.bmj.com/site/about/guidelines.xhtml

Appendix1: List of identified healthcare technologies

		efficacy for enhancing cognitive performance		symptoms and disease		vaccines)
Loveridge et al 1998	Policy planning	50% of patients presenting with mental illness such as schizphrenia and depression are diagnosed using objective genetic, biochemical or biophysical tests	21	Diagnosis and monitoring of disease	V - mental health	Diagnostic tests and imaging
Loveridge et al 1998	Policy planning	50% of surgical interventions carried out by minimum-access technique	21	Treatment and management of symptoms and disease	Other - generic	Therapeutic procedures
Loveridge et al 1998	Policy planning	10% of surgical interventions are carried out by robotic techniques	21	Treatment and management of symptoms and disease	Other - generic	Therapeutic procedures
Loveridge et al 1998	Policy planning	Development of biocompatible materials for implants which elicit the required host response (e.g. non-thrombogenic or stimulation of host repair mechanisms).	21	Treatment and management of symptoms and disease	Other - generic	Devices and biomaterials
Loveridge et al 1998	Policy planning	Widespread use for skin replacemt and/or repair of reliable products based on tissue engineering	21	Treatment and management of symptoms and disease	XII - skin	Regenerative medicine
Loveridge et al 1998	Policy planning	Practical availability for regular clinical use of functional human organ tissue for kidney, liver or pancreas	21	Treatment and management of symptoms and disease	Other - generic	Regenerative medicine
Loveridge et al 1998	Policy planning	Widespread use of non-surgical (eg. by	21	Health promotion and prevention of disease	XXI - screening, rehab	Drugs (not prophylactic vaccines)

For peer review only - http://bmjopen.bmj.com/site/about/guidelines.xhtml

Appendix1: List of identified healthcare technologies

		vaccination) single application contraception offering lifetime control				
Loveridge et al 1998	Policy planning	50% of contraception involving pharmacological or vaccine methods resides with men	21	Health promotion and prevention of disease	XXI - screening, rehab	Drugs (not prophylactic vaccines)
Loveridge et al 1998	Policy planning	50% of diagnostic and treatment decisions involve use of expert systems	21	Supporting patients receiving care (designed to be used by individuals)	Other - generic	IT systems, eHealth and mHealth
Loveridge et al 1998	Policy planning	20% of time spent by doctors in primary care screeing is replaced by patient interaction with experts systems	21	Supporting patients receiving care (designed to be used by individuals)	Other - generic	IT systems, eHealth and mHealth
Loveridge et al 1998	Policy planning	Practical use of remote electronic systems which substitute human labour in montoring the physical parameter of elderly and people with disability in the community	21	Supporting patients receiving care (designed to be used by individuals)	Other - generic	IT systems, eHealth and mHealth
Loveridge et al 1998	Policy planning	Widespread use of unified and standardised electronic data collection systems and data informats for medical records	21	Supporting the provision of care and/or increasing service efficiency	Other - generic	IT systems, eHealth and mHealth
Loveridge et al 1998	Policy planning	At least 50% of health care centres (hospitals, clinic,etc) are connected to national information	21	Supporting the provision of care and/or increasing service efficiency	Other - generic	IT systems, eHealth and mHealth

For peer review only - http://bmjopen.bmj.com/site/about/guidelines.xhtml

Appendix1: List of identified healthcare technologies

		electronic networks for patient records and information on diagnosis, treatment and outcome				
Loveridge et al 1998	Policy planning	Widespread use in health care of electronic information systems which span existing professional and organisational boundries (health, social service, housing etc)	21	Supporting the provision of care and/or increasing service efficiency	Other - generic	IT systems, eHealth and mHealth
Loveridge et al 1998	Policy planning	Improved understanding of the role of gene-environment interactions in causing common structural birth birth defects (e.g. cleft palate) permits the implementation of primary preventable programmes	21	Health promotion and prevention of disease	XXI - screening, rehab	Population programmes
Loveridge et al 1998	Policy planning	Understanding how environmental factors encountered in early life cause adult disease leads to national standards for diet and lifestyle in pre-natal and early life	21	Health promotion and prevention of disease	XXI - screening, rehab	Population programmes
Loveridge et al 1998	Policy planning	Widespread use of financial inducements by national and private health insurers or similar bodies to adopt	21	Health promotion and prevention of disease	Other - generic	Organisational programmes

For peer review only - http://bmjopen.bmj.com/site/about/guidelines.xhtml

Appendix1: List of identified healthcare technologies

		healthier lifestyle or diets				
Loveridge et al 1998	Policy planning	The causes of the rising prevalence of asthma are elucidated and action based on this knowledge leads to a 50% reduction in the incidence of severe disease	21	Health promotion and prevention of disease	X - respiratory	Population programmes
Operating theatre of the year 2010; Department of Trade and Industry	Policy planning	X-ray replaced by Magnetic Resonance Imaging (MRI)	14	Diagnosis and monitoring of disease	Other - generic	Diagnostic tests and imaging
Operating theatre of the year 2010; Department of Trade and Industry	Policy planning	operating theatre of 2010 will be built with an ultra-open MRI system with parallel ultrasound facilities and complete optical capability	14	Supporting the provision of care and/or increasing service efficiency	Other - generic	Medical equipment
Operating theatre of the year 2010; Department of Trade and Industry	Policy planning	Endoscopes will reduce in size as fibres, lenses and electronic sensors get smaller,	14	Supporting patients receiving care (designed to be used by individuals)	Other - generic	Devices and biomaterials
Operating theatre of the year 2010; Department of Trade and Industry	Policy planning	CCD image sensors have the ability to see in both ultra-violet and near infra-red.	14	Diagnosis and monitoring of disease	Other - generic	Diagnostic tests and imaging
Operating theatre of the year 2010; Department of Trade and Industry	Policy planning	New magnetic sensing technologies will detect the configuration of the endoscope and accessory devices inside the patient.	14	Supporting patients receiving care (designed to be used by individuals)	Other - generic	Devices and biomaterials

For peer review only

Appendix1: List of identified healthcare technologies

Operating theatre of the year 2010; Department of Trade and Industry	Policy planning	Auto focus and zoom optical systems will be interfaced with 30 to take the endoscope imaging capability beyond what is currently possible with human vision.	14	Diagnosis and monitoring of disease	Other - generic	Diagnostic tests and imaging
Operating theatre of the year 2010; Department of Trade and Industry	Policy planning	High resolution ultrasound in closer proximity to the tissue and structures to be studied	14	Diagnosis and monitoring of disease	Other - generic	Diagnostic tests and imaging
Operating theatre of the year 2010; Department of Trade and Industry	Policy planning	Laser induced fluorescence imaging systems for diagnosis of early malignant changes, monitoring and therapy will become available.	14	Diagnosis and monitoring of disease	II - cancers	Diagnostic tests and imaging
Operating theatre of the year 2010; Department of Trade and Industry	Policy planning	Near infra-red spectroscopy, for looking at tissue oxygenation state	14	Diagnosis and monitoring of disease	Other - generic	Diagnostic tests and imaging
Operating theatre of the year 2010; Department of Trade and Industry	Policy planning	Confocal, or other optical, ultrasonic or magnetic resonance microscopy, for on-line histology of bulk samples	14	Diagnosis and monitoring of disease	Other - generic	Diagnostic tests and imaging
Operating theatre of the year 2010; Department of Trade and Industry	Policy planning	Minimal Access Surgery is likely to account for as much as 75% of surgical procedures	14	Treatment and management of symptoms and disease	Other - generic	Therapeutic procedures
Operating theatre of the year 2010; Department of Trade and Industry	Policy planning	Robotic camera holders are likely to become routine in laparoscopic procedures.	14	Treatment and management of symptoms and disease	Other - generic	Medical equipment

For peer review only - http://bmjopen.bmj.com/site/about/guidelines.xhtml

Appendix1: List of identified healthcare technologies

Operating theatre of the year 2010; Department of Trade and Industry	Policy planning	The operating table's control system and the robotics system could become integrated in the future operating table.	14	Supporting the provision of care and/or increasing service efficiency	Other - generic	Medical equipment
Operating theatre of the year 2010; Department of Trade and Industry	Policy planning	Ability to ventilate all patients from neonates to adults	14	Treatment and management of symptoms and disease	X - respiratory	Non-surgical therapy
Operating theatre of the year 2010; Department of Trade and Industry	Policy planning	Control handsets will incorporate dot matrix displays which will give the operator information about the condition of the table, position of the top and warnings about possible hazards	14	Supporting the provision of care and/or increasing service efficiency	Other - generic	Medical equipment
Operating theatre of the year 2010; Department of Trade and Industry	Policy planning	The operating table may have a built-in patient temperature regulation system.	14	Supporting the provision of care and/or increasing service efficiency	Other - generic	Medical equipment
Operating theatre of the year 2010; Department of Trade and Industry	Policy planning	Connection between the Operating Room and the Anaesthetic machine in the Control Room will be via the Anaesthetist in the Operating Room.	14	Supporting the provision of care and/or increasing service efficiency	Other - generic	Medical equipment
Operating theatre of the year 2010; Department of Trade and Industry	Policy planning	The Anaesthetic machine will incorporate both inhalation agent vaporisation and multi-drug infusion systems These devices will be electronically integrated into the monitoring	14	Supporting the provision of care and/or increasing service efficiency	Other - generic	Medical equipment

For peer review only

Appendix1: List of identified healthcare technologies

		and information management and anaesthetic record keeping system so that vaporiser concentration settings and infusion rates will be continuously recorded				
Operating theatre of the year 2010; Department of Trade and Industry	Policy planning	Mechanical flow-meters will be replaced by electronic gas mixing systems	14	Supporting the provision of care and/or increasing service efficiency	Other - generic	Medical equipment
Operating theatre of the year 2010; Department of Trade and Industry	Policy planning	Patient monitoring will be very simple and intuitive probably touch screen and quite possibly voice activation.	14	Supporting patients receiving care (designed to be used by individuals)	Other - generic	IT systems, eHealth and mHealth
Operating theatre of the year 2010; Department of Trade and Industry	Policy planning	Monitors will also network into central areas outside the operating theatre so that, for example, anaesthetics being delivered by less experienced staff can be monitored remotely.	14	Supporting patients receiving care (designed to be used by individuals)	Other - generic	IT systems, eHealth and mHealth
Operating theatre of the year 2010; Department of Trade and Industry	Policy planning	The operation room will capture the data from one or perhaps two small 'wireless' sensors placed on the body that will provide pulse oxyimetry C02, ECG and other key parameter data and then transmit them to the monitor via radio or IR technology.	14	Supporting patients receiving care (designed to be used by individuals)	Other - generic	IT systems, eHealth and mHealth

For peer review only

Appendix1: List of identified healthcare technologies

Operating theatre of the year 2010; Department of Trade and Industry	Policy planning	Patient will be equipped with a belt (or other suitable garment) into which all monitoring and feed lines will be terminated, and which will include significant active capability. Drip and other lines will also be terminated in the belt	14	Supporting patients receiving care (designed to be used by individuals)	Other - generic	Devices and biomaterials
Operating theatre of the year 2010; Department of Trade and Industry	Policy planning	Patient can be "plugged" into electrical support, monitoring and stimulation as well as fluid and chemical agents using a single connector to facilitate transfer of patient from one ward to another or from the operation theatre	14	Supporting patients receiving care (designed to be used by individuals)	Other - generic	IT systems, eHealth and mHealth
Operating theatre of the year 2010; Department of Trade and Industry	Policy planning	Operation lamp will be replaced by a better light source	14	Supporting the provision of care and/or increasing service efficiency	Other - generic	Medical equipment
Operating theatre of the year 2010; Department of Trade and Industry	Policy planning	Automatic control of light pattern in the operation theatre	14	Supporting the provision of care and/or increasing service efficiency	Other - generic	Medical equipment
Operating theatre of the year 2010; Department of Trade and Industry	Policy planning	The operating lamp of the future is likely to use much more electronics to accurately control the voltage, dimming, focus, tracking and to limit maximum light intensity to	14	Supporting the provision of care and/or increasing service efficiency	Other - generic	Medical equipment

For peer review only - http://bmjopen.bmj.com/site/about/guidelines.xhtml

Appendix1: List of identified healthcare technologies

		safe levels.				
Operating theatre of the year 2010; Department of Trade and Industry	Policy planning	The operating lamps of the future will be sealed for easy disinfection	14	Supporting the provision of care and/or increasing service efficiency	Other - generic	Medical equipment
Operating theatre of the year 2010; Department of Trade and Industry	Policy planning	Telemedicine	14	Unknown	Other - generic	IT systems, eHealth and mHealth
Operating theatre of the year 2010; Department of Trade and Industry	Policy planning	Telemonitoring	14	Supporting the provision of care and/or increasing service efficiency	Other - generic	IT systems, eHealth and mHealth
Operating theatre of the year 2010; Department of Trade and Industry	Policy planning	Teleconsultation	14	Supporting the provision of care and/or increasing service efficiency	Other - generic	IT systems, eHealth and mHealth
Operating theatre of the year 2010; Department of Trade and Industry	Policy planning	Telesurgery for demonstartion or teaching	14	Supporting the provision of care and/or increasing service efficiency	Other - generic	IT systems, eHealth and mHealth
Operating theatre of the year 2010; Department of Trade and Industry	Policy planning	Minimal access surgery (MAS)	14	Treatment and management of symptoms and disease	Other - generic	Therapeutic procedures
Operating theatre of the year 2010; Department of Trade and Industry	Policy planning	Systems in which as much of the patient's own blood is recovered and recycled as possible.	14	Treatment and management of symptoms and disease	III - blood	Non-surgical therapy
Operating theatre of the year 2010; Department of Trade and Industry	Policy planning	Use of flexible endoscope technology to allow surgeons to see around and behind structures, giving the surgeon greater access and the patient a greater degree of safety.	14	Treatment and management of symptoms and disease	XI - GI tract, dental health	Devices and biomaterials

For peer review only - http://bmjopen.bmj.com/site/about/guidelines.xhtml

Appendix1: List of identified healthcare technologies

Operating theatre of the year 2010; Department of Trade and Industry	Policy planning	A move away from the use of X-rays and image intensification towards more mobile methods of MRI scanning.	14	Diagnosis and monitoring of disease	Other - generic	Diagnostic tests and imaging
Operating theatre of the year 2010; Department of Trade and Industry	Policy planning	Developments in the use of laser and particularly electrosurgery	14	Treatment and management of symptoms and disease	Other - generic	Therapeutic procedures
Operating theatre of the year 2010; Department of Trade and Industry	Policy planning	Use of robotics and head up display allowing surgeon sitting in a control room rather than being in the theatre with the patient	14	Supporting the provision of care and/or increasing service efficiency	Other - generic	Medical equipment
Operating theatre of the year 2010; Department of Trade and Industry	Policy planning	Coding will be used to associate patient, operating procedure (and so appropriate tools and drugs), support facilities and to generate automatic records of usage.	14	Supporting the provision of care and/or increasing service efficiency	Other - generic	IT systems, eHealth and mHealth
Operating theatre of the year 2010; Department of Trade and Industry	Policy planning	Tools and devices for a procedure will be located in a carousel analogous to that for a computer numerically controlled machine tool. This will allow the surgeon to present his tool holder to it, have the old tool extracted and a new one inserted.	14	Supporting the provision of care and/or increasing service efficiency	Other - generic	Medical equipment
Operating theatre of the year 2010; Department of Trade and Industry	Policy planning	Robots will be used as transport vehicles both within the operating room	14	Supporting the provision of care and/or increasing service efficiency	Other - generic	Medical equipment

For peer review only - http://bmjopen.bmj.com/site/about/guidelines.xhtml

Appendix1: List of identified healthcare technologies

		and for the surrounding support areas				
Operating theatre of the year 2010; Department of Trade and Industry	Policy planning	Robots will carry out very precise procedures controlled by the surgeon from a distance,	14	Supporting patients receiving care (designed to be used by individuals)	Other - generic	Medical equipment
Operating theatre of the year 2010; Department of Trade and Industry	Policy planning	Joint replacement be performed robotically	14	Treatment and management of symptoms and disease	XIII - musculoske letal	Therapeutic procedures
Operating theatre of the year 2010; Department of Trade and Industry	Policy planning	The operation theatre may be controlled by a robot "software" box th at will respond to voice recognition of the surgeon and change factors such as the heating of the theatre, the lighting and position of the table	14	Supporting the provision of care and/or increasing service efficiency	Other - generic	Medical equipment
Operating theatre of the year 2010; Department of Trade and Industry	Policy planning	A robot would be able to transfer sterile instrument trays from a central sterile store to the surgeon at the operating table.	14	Supporting the provision of care and/or increasing service efficiency	Other - generic	Medical equipment
Operating theatre of the year 2010; Department of Trade and Industry	Policy planning	Transport Robots could be used in the supply and disposal functions outside the operating room within the operating suite	14	Supporting the provision of care and/or increasing service efficiency	Other - generic	Medical equipment
Operating theatre of the year 2010; Department of Trade and Industry	Policy planning	Robots will be used in moving patients from ward to theatre areas (to replace the use of porters)	14	Supporting the provision of care and/or increasing service efficiency	Other - generic	Medical equipment

For peer review only

Appendix1: List of identified healthcare technologies

Operating theatre of the year 2010; Department of Trade and Industry	Policy planning	Using dedicated robot trucks similar to the supply trucks, waste could be collected on a regular basis from each theatre and taken to an automatic local waste reduction/ disposal facility adjacent to the theatre suite.	14	Supporting the provision of care and/or increasing service efficiency	Other - generic	Medical equipment
Operating theatre of the year 2010; Department of Trade and Industry	Policy planning	Disposal of hospital material could be performed on site with the use of machines such as "needle eaters"	14	Supporting the provision of care and/or increasing service efficiency	Other - generic	Medical equipment
Operating theatre of the year 2010; Department of Trade and Industry	Policy planning	Bulky X-Ray machines will probably be a thing of the past and replaced by computerised radiology	14	Diagnosis and monitoring of disease	Other - generic	Medical equipment
Operating theatre of the year 2010; Department of Trade and Industry	Policy planning	A central control room, with visual links to each theatre served, could house all of the equipment.	14	Supporting the provision of care and/or increasing service efficiency	Other - generic	Medical equipment
Operating theatre of the year 2010; Department of Trade and Industry	Policy planning	All theatres could be designed with ultra-clean ventilation systems or an adapted containment system to reduce the risk of the spread of infection	14	Health promotion and prevention of disease	I - infections	Medical equipment
Operating theatre of the year 2010; Department of Trade and Industry	Policy planning	Patients identification bracelets could be coded and bar checked as they moved through	14	Supporting patients receiving care (designed to be used by individuals)	Other - generic	Medical equipment

For peer review only - http://bmjopen.bmj.com/site/about/guidelines.xhtml

Appendix1: List of identified healthcare technologies

		the system so that their progress could be monitored at all times				
Operating theatre of the year 2010; Department of Trade and Industry	Policy planning	Automated trolleys and doors that can be electronically controlled by robots	14	Supporting the provision of care and/or increasing service efficiency	Other - generic	Medical equipment
UK technology and innovation futures for the 2020s, 2010	Policy planning	Further development of 3D imaging techniques.	10	Diagnosis and monitoring of disease	Other - generic	Diagnostic tests and imaging
UK technology and innovation futures for the 2020s, 2010	Policy planning	Fluorescenceassisted Resection and Exploration (FLARE) technologies, which combine both visible light imaging with near-infra red imaging of fluorescent biomarkers, can detect particular pathologies associated with tumours without the need for biopsy	10	Diagnosis and monitoring of disease	Other - generic	Diagnostic tests and imaging
UK technology and innovation futures for the 2020s, 2010	Policy planning	Colour X-ray or TEDDI imaging can be used to biopsy tissues by utilising scattered X-rays to build a spatially-resolved picture that can distinguish between different materials.	10	Diagnosis and monitoring of disease	Other - generic	Diagnostic tests and imaging
UK technology and innovation futures for the 2020s, 2010	Policy planning	In vivo neuroimaging, both at the tissue and cellular level	10	Diagnosis and monitoring of disease	Other - generic	Diagnostic tests and imaging
UK technology and innovation futures for the	Policy planning	Brain imaging techniques such as functional	10	Diagnosis and monitoring of disease	Other - generic	Diagnostic tests and imaging

For peer review only - http://bmjopen.bmj.com/site/about/guidelines.xhtml

Appendix1: List of identified healthcare technologies

2020s, 2010		magnetic resonance imaging (fMRI)				
UK technology and innovation futures for the 2020s, 2010	Policy planning	Lab-on-a-Chip (LOC) devices integrate and scale-down biomedical and other analytical laboratory functions and processes to a miniaturised chip format.	10	Diagnosis and monitoring of disease	Other - generic	Diagnostic tests and imaging
UK technology and innovation futures for the 2020s, 2010	Policy planning	Nucleic acid probes and diagnostic microarray systems can detect the nucleic acid sequence or sequences that identify a specific infectious disease agent, human genetic disease or individual (DNA profiling).	10	Diagnosis and monitoring of disease	Other - generic	Diagnostic tests and imaging
UK technology and innovation futures for the 2020s, 2010	Policy planning	A new focus on single-stranded DNA, micro RNA (miRNA) and double-stranded RNA (dsRNA) technologies as therapeutics may lead to clinically-approved interventions for gene therapy and treatment of medical disease.	10	Treatment and management of symptoms and disease	Other - generic	Regenerative medicine
UK technology and innovation futures for the 2020s, 2010	Policy planning	RNA interference (RNAi) may lead to new treatments.	10	Treatment and management of symptoms and disease	Other - generic	Regenerative medicine
UK technology and innovation futures for the 2020s, 2010	Policy planning	Viruses, bacteria or yeasts may be able to act as carriers for new sequences of DNA	10	Treatment and management of symptoms and disease	Other - generic	Regenerative medicine

For peer review only - http://bmjopen.bmj.com/site/about/guidelines.xhtml

Appendix1: List of identified healthcare technologies

UK technology and innovation futures for the 2020s, 2010	Policy planning	Increased understanding of individual sensitivities to drugs	10	Diagnosis and monitoring of disease	III - blood	Drugs (not prophylactic vaccines)
UK technology and innovation futures for the 2020s, 2010	Policy planning	DNA vaccines for AIDS, malaria, hepatitis B and certain cancers may be available within 10 years	10	Health promotion and prevention of disease	Other - generic	Prophylactic vaccines
UK technology and innovation futures for the 2020s, 2010	Policy planning	New treatments for for neurodegenerative conditions such as dementia.	10	Treatment and management of symptoms and disease	VI - nervous	Unknown
UK technology and innovation futures for the 2020s, 2010	Policy planning	‘Cognition enhancers’; substances that enhance the performance of our brain in specific ways, such as improving short-term memory and increasing our speed of thought enhancement through lifestyle drugs may will become the norm.	10	Treatment and management of symptoms and disease	VI - nervous	Drugs (not prophylactic vaccines)
UK technology and innovation futures for the 2020s, 2010	Policy planning	‘Emoticeuticals’, drugs that act by changing hormone levels in the body, may mediate our responses in our private lives and work.	10	Treatment and management of symptoms and disease	V - mental health	Drugs (not prophylactic vaccines)
UK technology and innovation futures for the 2020s, 2010	Policy planning	Sensoceuticals’ might enhance pleasure by restoring or accentuating the senses.	10	Treatment and management of symptoms and disease	Other - generic	Drugs (not prophylactic vaccines)
UK technology and innovation futures for the 2020s, 2010	Policy planning	The advent of a drug that mimics the positive effects of alcohol	10	Health promotion and prevention of disease	V - mental health	Drugs (not prophylactic vaccines)

For peer review only - http://bmjopen.bmj.com/site/about/guidelines.xhtml

Appendix1: List of identified healthcare technologies

		with none of its drawbacks.				
UK technology and innovation futures for the 2020s, 2010	Policy planning	Stem cells replacing malfunctioning liver cells or even growing entire livers to replace diseased ones	10	Treatment and management of symptoms and disease	XI - GI tract, dental health	Regenerative medicine
UK technology and innovation futures for the 2020s, 2010	Policy planning	Stem cells giving new neurons to those with brain damage	10	Treatment and management of symptoms and disease	VI - nervous	Regenerative medicine
UK technology and innovation futures for the 2020s, 2010	Policy planning	Stem cells giving new heart cells to those with heart disease	10	Treatment and management of symptoms and disease	IX - circulation	Regenerative medicine
UK technology and innovation futures for the 2020s, 2010	Policy planning	Stem cells giving therapies for Parkinson's and Alzheimer's diseases	10	Treatment and management of symptoms and disease	VI - nervous	Regenerative medicine
UK technology and innovation futures for the 2020s, 2010	Policy planning	Stem cells giving therapies for spinal cord injury, stroke, cancer, burns, cardiovascular disease, diabetes, osteoarthritis and rheumatoid arthritis; as well as furnishing an endless supply of red blood cells for transfusions.	10	Treatment and management of symptoms and disease	Other - generic	Regenerative medicine
UK technology and innovation futures for the 2020s, 2010	Policy planning	Development of a cheap anti-malarial drug.	10	Treatment and management of symptoms and disease	I - infections	Drugs (not prophylactic vaccines)
UK technology and innovation futures for the 2020s, 2010	Policy planning	Programming a bacterium or virus that can identify malignant cells and can deliver a therapeutic agent could have major benefits for treating cancer and similar illnesses	10	Treatment and management of symptoms and disease	II - cancers	Regenerative medicine

For peer review only - http://bmjopen.bmj.com/site/about/guidelines.xhtml

Appendix1: List of identified healthcare technologies

UK technology and innovation futures for the 2020s, 2010	Policy planning	Stratified medicine: it will become increasingly possible to analyse an individual for certain biomarkers to determine the best course of treatment	10	Diagnosis and monitoring of disease	Other - generic	Diagnostic tests and imaging
UK technology and innovation futures for the 2020s, 2010	Policy planning	Personalised medicine	10	Treatment and management of symptoms and disease	Other - generic	Drugs (not prophylactic vaccines)
UK technology and innovation futures for the 2020s, 2010	Policy planning	In-situ tissue regeneration: Material is implanted directly in the body and regenerates the tissue over time as the material degrades.	10	Treatment and management of symptoms and disease	Other - generic	Regenerative medicine
UK technology and innovation futures for the 2020s, 2010	Policy planning	Tissue engineering: Material can act as temporary scaffold for cell culture and tissue growth in vitro	10	Treatment and management of symptoms and disease	Other - generic	Regenerative medicine
UK technology and innovation futures for the 2020s, 2010	Policy planning	Micro-masonry and 3D organ printing techniques	10	Treatment and management of symptoms and disease	Other - generic	Regenerative medicine
UK technology and innovation futures for the 2020s, 2010	Policy planning	Brain Computer Interface	10	Rehabilitation	Other - generic	Devices and biomaterials
UK technology and innovation futures for the 2020s, 2010	Policy planning	Neuroprosthetics: the connection can occur between any part of the nervous system (such as peripheral nerves) and a device.	10	Rehabilitation	VI - nervous	Devices and biomaterials

For peer review only

Appendix1: List of identified healthcare technologies

UK technology and innovation futures for the 2020s, 2010	Policy planning	Magnetoencephalography (MEG): maps brain activity by recording the magnetic fields produced by electrical currents occurring in the brain	10	Diagnosis and monitoring of disease	VI - nervous	Diagnostic tests and imaging
UK technology and innovation futures for the 2020s, 2010	Policy planning	functional magnetic imaging (fMRI) – involves measuring changes in blood flow related to neural activity in the brain.	10	Diagnosis and monitoring of disease	VI - nervous	Diagnostic tests and imaging
UK technology and innovation futures for the 2020s, 2010	Policy planning	functional Near-Infrared Spectroscopy (fNIRS) for optical topography	10	Diagnosis and monitoring of disease	Other - generic	Diagnostic tests and imaging
UK technology and innovation futures for the 2020s, 2010	Policy planning	Electrocorticography (EoCG), measure the electrical activity of the brain using electrodes which are implanted inside the skull, but outside the brain tissue.	10	Diagnosis and monitoring of disease	VI - nervous	Diagnostic tests and imaging
UK technology and innovation futures for the 2020s, 2010	Policy planning	Implanted brain chips for controlling computers	10	Rehabilitation	XXI - screening, rehab	Devices and biomaterials
UK technology and innovation futures for the 2020s, 2010	Policy planning	Neural prosthetics replace lost limbs	5	Rehabilitation	XXI - screening, rehab	Devices and biomaterials
UK technology and innovation futures for the 2020s, 2010	Policy planning	Thought-controlled robots for personal healthcare	10	Supporting patients receiving care (designed to be used by individuals)	Other - generic	Medical equipment
UK technology and innovation futures for the 2020s, 2010	Policy planning	Thought-controlled wheelchairs	10	Rehabilitation	XXI - screening, rehab	Medical equipment

For peer review only - http://bmjopen.bmj.com/site/about/guidelines.xhtml

Appendix1: List of identified healthcare technologies

UK technology and innovation futures for the 2020s, 2010	Policy planning	ehealth: Examples include health information networks, electronic health records, tele-medicine services, personal wearable and portable mobile devices, health portals and other tools that assist in health monitoring, diagnosis and treatment.	10	Supporting the provision of care and/or increasing service efficiency	Other - generic	IT systems, eHealth and mHealth
UK technology and innovation futures for the 2020s, 2010	Policy planning	Lab-on-a-chip technologies could also bring diagnostics down to a personal scale, where patients can test themselves and transmit data electronically for analysis and treatment.	10	Supporting patients receiving care (designed to be used by individuals)	Other - generic	IT systems, eHealth and mHealth
UK technology and innovation futures for the 2020s, 2010	Policy planning	Tailored Medicine	10	Treatment and management of symptoms and disease	Other - generic	Drugs (not prophylactic vaccines)
Technology foresight towards 2020_China	Policy planning	The anti-viral medicine with highly efficiency applies to clinical medicine widely	17	Treatment and management of symptoms and disease	I - infections	Drugs (not prophylactic vaccines)
Technology foresight towards 2020_China	Policy planning	standardized safe evaluation system which include the technology for monitoring and examination of trans-gene biology will be established;	17	Supporting the provision of care and/or increasing service efficiency	Other - generic	Organisational programmes

For peer review only - http://bmjopen.bmj.com/site/about/guidelines.xhtml

BMJ Open

Past speculations of future health technologies: a description of technologies predicted in 15 forecasting studies 1986-2010

Journal:	*BMJ Open*
Manuscript ID	bmjopen-2017-016206.R2
Article Type:	Research
Date Submitted by the Author:	24-May-2017
Complete List of Authors:	Doos, Lucy; University of Birmingham, Institute of Applied Health Research, NIHR Horizon Scanning Research and Intelligence Centre<br>Packer, Claire; NIHR Horizon Scanning Research and Intelligence Centre, Insitute of Applied Health Research, University of Birmingham<br>Ward, Derek; University of Birmingham, NIHR Horizon Scanning Research and Intelligence Centre, Institute of Applied Health Research<br>Simpson, Sue; University of Birmingham, NIHR Horizon Scanning Research and Intelligence Centre, Institute of Applied Health Research<br>Stevens, Andrew; University of Birmingham, Institute of Applied Health Research
**Primary Subject Heading**:	Public health
Secondary Subject Heading:	Health policy
Keywords:	Forecasting, prediction, health technology, innovation, healthcare

SCHOLARONE™
Manuscripts

Past speculations of future health technologies: a description of technologies predicted in 15 forecasting studies 1986-2010

Lucy Doos[1], Claire Packer[1], Derek Ward[1], Sue Simpson[1], Andrew Stevens[2]

[1]NIHR Horizon Scanning Research and Intelligence Centre, Institute of Applied Health Research, University of Birmingham

[2] Institute of Applied Health Research, University of Birmingham

Corresponding author:

Dr Lucy Doos

Institute of Applied Health Research,

Public Health Building,

University of Birmingham, Edgbaston,

Birmingham, B15 2TT, UK.

Email: l.doos@bham.ac.uk

Phone: (+44)121 414 8594

Keywords: Forecasting, prediction, health technology, innovation, healthcare

Word count: 3,934

For peer review only

ABSTRACT

Objective: To describe and classify health technologies predicted in forecasting studies.

Design and methods: Description of health technologies predicted in 15 forecasting studies published between 1986 and 2010 that were identified in a previous systematic review. Health technologies are classified according to their type, purpose and clinical use; relating these to the original purpose and timing of the forecasting studies.

Data sources: All health-related technologies predicted in 15 forecasting studies identified in a previously published systematic review.

Main outcome measure: Outcomes related to i) each forecasting study including country, year, intention and forecasting methods used and ii) the predicted technologies including technology type, purpose, targeted clinical area and forecast timeframe.

Results: Of the 896 identified health-related technologies, 685 (76.5%) were health technologies with an explicit or implied health application and included in our study. Of these, 19.1% were diagnostic or imaging tests, 14.3% devices or biomaterials, 12.6% information technology systems, eHealth or mHealth, and 12% drugs. The majority of the technologies were intended to treat or manage disease (38.1%) or diagnose or monitor disease (26.1%). The most frequent targeted clinical areas were infectious diseases followed by cancer, circulatory and nervous system disorders. The most frequent technology types were for: infectious diseases - prophylactic vaccines (45.8%); cancer - drugs (40%); circulatory disease - devices and biomaterials (26.3%), and diseases of the nervous system - equally devices and biomaterials (25%) and regenerative medicine (25%). The mean timeframe for forecasting was 11.6 years (range 0-33 years, median=10, SD= 6.6). The forecasting timeframe significantly differed by technology type (p=0.002), the intent of the forecasting group (p<0.001), and the methods used (p<001).

For peer review only - http://bmjopen.bmj.com/site/about/guidelines.xhtml

Conclusion: Description and classification of predicted health-related technologies is crucial in preparing healthcare systems for adopting new innovations. Further work is needed to test the accuracy of predictions made.

Strengths and limitations of the study

- The study is a comprehensive description and classification of health-related technologies predicted in 15 forecasting studies identified in a previously published systematic review.
- The study summarises the forecast technologies by type e.g. medical devices, diagnostic tests or drugs; by clinical purpose e.g. prevention, diagnosis or treatment and by targeted clinical area by ICD10 chapter; and relates this to the forecasting purpose and timeframe.
- The analysis and the health-related technologies identified are constrained by the methods used in the forecasting studies for example by the areas of interest of the experts involved.
- Although the study highlights the identified technologies, the retrospective accuracy of the studies remains to be researched.

For peer review only

For peer review only - http://bmjopen.bmj.com/site/about/guidelines.xhtml

INTRODUCTION

People are in general living longer and surviving previously life threatening illnesses. A proportion of this effect is attributed to the use of novel health interventions and technologies, such as new vaccines to prevent disease; new diagnostic tests to diagnose diseases earlier and with improved accuracy; and new drugs, devices and surgical procedures to treat diseases more effectively. In addition, new health technologies may also be used to enhance function and improve quality of life, such as prostheses, sensory aids and cosmetic enhancements. Technological advances and innovation are leading to new health interventions becoming available to healthcare markets at an increasing speed; these often cost more than current alternatives and significantly affect the cost of healthcare services and delivery.[1,2] To ensure that patients can access the most effective and cost effective interventions, and because many health systems work within constrained budgets, healthcare systems need to be prepared to respond to new developments. A crucial step in such preparation is the accurate identification and forecasting of likely significant technological healthcare developments.[3,4]

Forecasting is widely used for planning and strategic decision making around industrial and economic development at organisational, regional and national levels.[5] Within healthcare, forecasting can be used to build strategies for supporting innovators and companies to develop new interventions; to plan future research programmes and delivery of healthcare; and to prepare health services and personnel to respond to technological developments of benefit to patients and service provision. Health technology forecasting uses various methods such as Delphi surveys and reviews of the literature[6,7,8] to identify emerging health technologies that are intended to address important unmet healthcare needs or that need additional evaluation, and to analyse their potential impact on patients and health systems.[4]

To get the maximum benefit from forecasting, it is important that the methods used to identify emerging interventions and technologies are appropriate for the aims and timeframe of the forecasting exercise and also as accurate as possible.

For peer review only - http://bmjopen.bmj.com/site/about/guidelines.xhtml

In a recent systematic review[9] we found 15 forecasting studies with 3 to 20 year timeframes that identified emerging health-related topics and technologies predicted between 1986 and 2010 from 12 high income countries, including six from the United Kingdom (UK). Identifying and classifying the predicted new technologies into meaningful groupings is important for healthcare system preparedness, allowing planned assessment and adoption as appropriate. Here we describe and classify the health technologies predicted in the forecasting studies according to their type, purpose and clinical use. We relate these to the forecast time period and develop an overview of the technological and clinical frontiers of innovation in health and healthcare provision.

METHODS

See table 1 for a full list of the 15 studies identified in our prior systematic review. We extracted all forecast topics from the 15 forecasting studies using a standardised set of data for each of these predicted topics and on the source study. Data related to the forecast topics included the name of the topic as written in the forecasting reports and papers, and the predicted forecast timeframe. Data related to the study included the country or region of the forecast, year the study was undertaken, its intention and remit, the study forecasting timeframe, and the number and type of forecasting methods used.

Identification and inclusion of health technologies

For final inclusion and analysis, topics had to be health technologies as defined by the National Institute of Health Research (NIHR) Health Technology Assessment (HTA) programme[10]: "*any method used to promote health; prevent and treat disease; and improve rehabilitation or long-term care. They are not confined to new drugs and include any intervention used in the treatment, prevention or diagnosis of disease*". We also included topics that related to the recognition of, or change in, health states or emotions, or health-related behaviours. All topics needed to have an explicit or implied health-related application or identified patient group for final inclusion. Topics were excluded if they related exclusively to foods, plants, animals, insects, new sources of fuel or energy, environmental contamination, legislation or health insurance.

For peer review only - http://bmjopen.bmj.com/site/about/guidelines.xhtml

For peer review only

LD read all the included studies and manually extracted all technologies mentioned in the forecast from the published text and related tables. Two authors (CP and LD) independently scrutinised each identified topic and applied the inclusion and exclusion criteria. Where there was insufficient information on a topic to make the inclusion/exclusion decision, we undertook brief and very limited searches on the Internet to find additional information.

All topics classed as a health technology with an explicit or implied health-related application, were subsequently coded based on their technology type, intended purpose, and targeted clinical use:

Technology type: we used broad categories: assistive devices and rehabilitation aids; devices and biomaterials; diagnostic tests and imaging; drugs; information technology (IT) systems, electronic health systems (eHealth) and mobile health (mHealth); medical equipment; non-surgical therapy; organisational programmes; prophylactic vaccines; population programmes; regenerative medicine approaches; and therapeutic procedures.

Technology purpose: we used categories that relate to the intended point on the patient pathway: health promotion and the prevention of disease; diagnosis and monitoring of disease; treatment and management of symptoms and disease; and rehabilitation. We supplemented these with two groupings that categorised technologies that support the provision of care i) supporting patients receiving care and designed to be used by individuals (patients, caregivers or healthcare professionals) and ii) supporting the provision of care and/or increasing service efficiency.

Targeted clinical area (specialty): we used the international statistical classification of diseases and related health problems 10th revision (ICD10)[11] chapter headings relating to diseases and other morbid conditions (chapters I-XVII); symptoms, signs and abnormal findings (chapter XVIII); injuries, poisoning and other consequences of external causes (chapter XIX); external causes of diseases and morbid conditions chapter XX); and factors influencing health status and contact with health services for people not currently sick (chapter XXI). Chapter XXI includes contraception,

For peer review only - http://bmjopen.bmj.com/site/about/guidelines.xhtml

technologies used in population screening, health promotion and disease prevention, and general rehabilitation.

All topics with disputed inclusion, exclusion or categorisation were initially discussed between two authors (LD and CP) and disagreements resolved where possible. Where disagreements remained, topics were discussed with the other authors as a group and the final categorisation agreed through consensus.

Technology forecast timeframe: for each health-related technology forecast we calculated the difference in years between each technology's predicted year of impact as identified in the original study, and the year when the prediction was made, i.e. the year of the original study. Technology forecasts were classified as short term if their forecasting timeframe was less than 3 years, short to medium term if it was 3-10 years, medium to long term if it was 11-20 years and long term for those with timeframes of more than 20 years. Although the original systematic review excluded forecasting studies which exclusively adopted a short time frame of less than 3 years, some studies with longer time frames also provided predictions with shorter time frames, and these were included in the analysis.

Data analysis

We analysed the data using IBM SPSS statistics (version 22) for Windows. We present descriptive analyses as means and standard deviations (SD) for normally distributed continuous variables. Statistically significant differences were determined using ANOVA for continuous normally distributed data and X^2 for dichotomous variables.

RESULTS

We identified 896 predicted topics from the 15 forecasting studies. Of these, we judged that 685 (76.5%) were health-related technologies with an explicit or implied health-related application. The most frequently excluded topics were those that described advances in the underpinning scientific knowledge (8.7%), such as determination of whole human DNA base sequence and identifying genetic links to

For peer review only - http://bmjopen.bmj.com/site/about/guidelines.xhtml

diseases. A full list of the included health-related technologies is attached as an online appendix.

Of the 685 health-related technologies, 52.3% were forecast from 6 studies by governmental organisations (such as the UK Department of Trade and Industry[12]), 21.9% by commercial/consultancy organisations (such as British Telecommunications plc[13]), 14.5% by policy planning groups (such as Loveridge et al 1995[6]), 8.9% by research groups (such as those by Stevens et al 1997[7]), and 2.5% by non-profit organisations (such as Institute of the Future[14]). Looking at the purpose of forecasting, our data showed that two thirds (68%) of the health technologies were forecast for policy planning purposes, 18.1% for research purposes and 13.9% for commercial purposes.

Nearly half (48.9%) of the identified technologies were from 6 UK-based studies and reports, 15.0% were from Japan, 11.7% from the Netherlands, 11.4% from the United States of America (USA) and 8.0% from Canada. The remainder were from a homogenous group of developing countries and South Africa (3.8%), other European countries (0.9%) and China (0.3%).

Technology type

Of the 685 health technologies forecast, 19.1% were diagnostic tests and imaging technologies, such as advanced ultrasound imaging systems and molecular diagnosis; 14.3% were devices and biomaterials, such as tissue engineered devices and drug impregnated devices; 12.6% were IT systems, eHealth and mHealth, such as electronic prescriptions and telemedicine; 12.0% were drugs, such as new anaesthetic vapours and electronically activated drugs; and 11.7% were regenerative medicine approaches, such as gene therapy for diabetes and widespread use of gene therapy for familial hypercholesterolemia (Table 2 and online appendix).Drug technologies were more commonly forecast in studies carried out by researchers (36.1%) than other groups, while technologies for diagnostic tests and imaging were more commonly forecast in studies by governmental agencies than other groups (Table 3). Regenerative medicine approaches were the most commonly forecast technologies (23.2%) by studies with a commercial intent while diagnostic tests and imaging were the most commonly forecast technologies by

For peer review only - http://bmjopen.bmj.com/site/about/guidelines.xhtml

studies with policy planning and research intentions (20.6% and 23.4% respectively) ($p<0.001$).

Technology purpose

Of the 685 health technologies included in the forecasting reports, 38.1% were for the treatment and management of symptoms and disease, such as development of effective treatment for amyotrophic lateral sclerosis and the practical use of gene therapy for genetic disorders; 21.6% for the diagnosis and monitoring of disease, for example a diagnostic bio-chip for cancer and the further development of 3D imaging techniques; 13% for health promotion and the prevention of disease, such as the first effective vaccine of HIV and DNA vaccines for AIDS, malaria, hepatitis B and certain cancers; 10.2% for supporting the provision of care, for example tele-monitoring and teleconsultation; 8.0% for supporting patients receiving care, such as smart pill bottles to remotely monitor medication use and thought-controlled robots for personal healthcare; and 4.7% for rehabilitation, for example artificial legs and robotic prosthetics.

Technologies for treatment and disease management were the most common regardless of the intention of the study: 35.8% among studies with a commercial intent, 35.8% for policy planning and 48.4% for research. Technologies for treatment and disease management were also the most common technologies forecast by all the various groups (60.7% among researchers, 39.9% among governmental organisations and 35.8% among commercial organisations).

Targeted clinical area

Using the ICD10 codes to define the targeted clinical area, just over half of the health technologies (50.4%) were for diseases and conditions that were not specified or that could not be coded or that crossed multiple specialities. Of the 340 health technologies that could be coded to a specific ICD10 chapter, the top five targeted clinical areas were: 1) infectious and parasitic diseases ICD-I (14.1%), such as development of vaccines for AIDS and vaccines for malaria, 2) neoplasms ICD-II (11.8%), such as expanding metal stents for oesophageal cancer and diagnostic bio-chip for cancer, 3) diseases of the circulatory system ICD-IX (11.2%), such as artificial muscles in replace of heart transplants and implantable vascular stents, 4)

For peer review only - http://bmjopen.bmj.com/site/about/guidelines.xhtml

diseases of the nervous system ICD-VI (10.6%), such as artificial brain cells and artificial peripheral nerves and 5) factors influencing health status and contact with health services ICD-XXI (10.3%), such as a personal wearable health monitor and extension of average lifespan to over 100.

Congenital malformations, deformations and chromosomal abnormalities ICD-XVII and symptoms, signs and abnormal clinical and laboratory findings, not elsewhere classified ICD- XVIII had the fewest forecast technologies (0.3% each).

Table 4 shows the technology types and purposes for the forecast technologies within the five most frequent targeted clinical areas. The most frequent technology type forecast for infectious and parasitic diseases were prophylactic vaccines (45.8%); for cancer were drugs (40%); for diseases of the circulatory system were devices and biomaterials (26.3%); for diseases of the nervous system were devices and biomaterials (25%) and regenerative medicine (25%); and for factors influencing health status and contact with health services including screening and rehabilitation, devices and biomaterials (31.4%). The most frequent technology purpose was health promotion and disease prevention for both infectious and parasitic diseases (ICD-I) (60.4%) and factors influencing health status and contact with health services including screening and rehabilitation (ICD-XXI) (51.4%). Technologies for treatment and disease management were most common for cancer (55.0%), diseases of the circulatory system (65.8%) and nervous system disorders (61.1%).

Technology forecast timeframe

Data was available to calculate the prediction timeframe for 607 of the identified health technologies. The mean timeframe for forecasting predictions was 11.6 years (range 0-33 years, median=10, SD=6.6). There was a significant difference in the mean prediction timeframe by technology type (p=0.002), with non-surgical therapies having the shortest mean forecast timeframe (9.07 years) and population health programmes having the longest mean forecast (14.53 years) (Table 5). There were no statistically significant differences in the mean duration of the technology prediction timeframe by the technology purpose and we were not able to detect any particular trend in the technology types or purposes of forecast over time.

For peer review only - http://bmjopen.bmj.com/site/about/guidelines.xhtml

There was a statistically significant difference in the forecast timeframe by the type of group undertaking the forecasting ($p<0.001$): 58% of the long-term forecasts (>20 years) were made by policy planning groups and 51% of short-term forecasts (<3 years) were by research groups.

Technologies forecast by studies with research intentions had the shortest mean forecast timeframe (4.49 years) while those for policy making had the longest timeframe (14.16 years) ($p<0.001$). Similarly, technologies forecast by researchers had significantly shorter mean timeframes (3.56 years) compared to those made by governmental agencies which had the longest mean forecast timeframes (13.42 years) ($P<0.001$).

There was a statistically significant difference in the mean forecast timeframe when considering the forecasting method ($p<0.001$) (Figure 1). Technologies forecast using methods that included creativity-based methods which require brainstorming, such as scenario building, had a significantly longer forecasting timeframe (13.5 years) compared to those did not include creativity-based methods such as literature reviews (9.7 years) ($p<0.001$). On the other hand technologies forecast using methods that included evidence-based methods such as literature reviews and evidence from health technology assessment (HTA) organisations, had significantly shorter forecasting timeframes (8.2 years) than those that did not include evidence-based methods (10.8 years) ($p<0.001$).

DISCUSSION

Being aware of forthcoming developments in healthcare and preparing for the future is desirable for health policy makers, whether developing healthcare policy, directing future research or commissioning health services. The use that health policy makers make of forecasting depends on having systems in place that receive and act on the information at an appropriate time. There are few examples of such systems in the published literature; [15] however the relationship between the UK's National Institute for Health and Care Excellence (NICE) and National Institute for Health Research (NIHR) Horizon Scanning Research and Intelligence Centre does provide a model of how shorter-term early awareness intelligence can shape prioritisation decisions.[16]

For peer review only - http://bmjopen.bmj.com/site/about/guidelines.xhtml

We found that more than two thirds of the identified technologies were forecast for policy planning purposes and over half were forecast by governmental organisations, and that the majority of long-term forecasts were made by policy planning groups as opposed to research groups. This finding supports the premise that the majority of forecasting is carried out for service preparedness and long-term planning, and strategic decision making.[5]

Our study identified and classified a number of important health-related technologies, which health services may have already had to manage or will have to do so in the future. Our finding that nearly 60% of the technologies were either for the treatment and management of symptoms and disease or for the diagnosis and monitoring of disease can perhaps be explained by the methods used for forecasting. Many of the studies involved soliciting the opinion of experts and, although it is not possible to be certain about the areas of interest of those involved, their expertise may have made them more likely to focus on diagnosis and treatment than, for example, organisational programmes or rehabilitation. Regenerative medicine approaches were the most commonly forecast technologies by studies with a commercial intent. In contrast, drugs and related technologies (but not prophylactic vaccines) were the most common forecasts made by researchers.

The four clinical areas with the greatest number of forecast health technologies: infectious diseases, cancer, and circulatory and nervous system diseases and disorders, include many of the major causes of death and disability in countries today. Again we are not able to determine if this merely reflects the interests of the groups carrying out the forecasting and their areas of expertise or whether it truly represents the technology innovation frontier. It is understandable that the forecasts for infectious disease included many prophylactic vaccines as these have proven to be a cost effective preventive strategy for their prevention.[17]This was also supported by our finding that forecasts of vaccine related technologies were only made by governmental agencies and policy research groups. In cancer, forecasting included both drugs and diagnostic tests and imaging, perhaps reflecting increasing targeting in novel cancer therapies.[18,19] Several forecasts of regenerative medicine approaches for nervous system diseases and disorders is also interesting, with many of these diseases not currently curable or effectively treated.[20,21]

For peer review only - http://bmjopen.bmj.com/site/about/guidelines.xhtml

Our finding that forecasts which included creativity-based methods such as scenario building and analysis had, on the whole, longer forecast timeframes than studies that included evidence-based methods may have a rationale. Evidence-based methods require there to be research evidence on technologies which will therefore probably be nearer to any predicted impact. In contrast, creativity-based methods require experts to think more widely about what the future could be, and are not therefore constrained by what is actually being currently researched and published. This may also explain our finding that just over half of the short-term forecasts were made by research groups who may be more likely to use published evidence to inform their deliberations.

We believe that this is the first study to comprehensively identify and summarise health-related technologies predicted in past forecasting projects and to categorise and describe them. Although as many predicted topics as possible were included, we did exclude a substantial number. This was often because we could not envisage a future health application or because there was insufficient information to make a judgement (and many topics had very sparse titles or descriptive information). By excluding topics in this way, we may have excluded some that would have had an explicit health application within a reasonable timeframe. In addition, it is likely that we were unable to identify a number of relevant forecasts made by commercial organisations, which will have been conducted and disseminated in confidence.

Our description and classification of predicted health-related technologies from prior forecasting studies provides an overview of the technological and clinical frontiers of innovation in health and health care provision. To complete our evaluation of the accuracy of previous forecasting predictions and the forecasting methods used, the accuracy of the predictions made should also be assessed. This may be difficult across all technology types and clinical areas, but possible by limiting the evaluation to specific clinical areas and/or technology types or to those topics with more obvious health applications and timeframes that have already occurred.

CONTRIBUTERS

All authors have directly participated in the planning and execution of the study. LD extracted the study data. LD and CP independently reviewed the technologies to be

For peer review only

included. LD and CP drafted the paper and DW, SS and AS critically revised the manuscript. All authors agreed the final version of the paper. All authors had full access to all of the data.

DISCLAIMER

This report presents independent research funded by the National Institute for Health Research (NIHR). The views expressed in this publication are those of the authors and not necessarily those of the National Health Service, the NIHR or the Department of Health.

COMPETING INTERESTS

The authors report that they do not have any conflicts of interest.

FUNDING

At the time of the study LD, CP, DW and SS were funded by the United Kingdom's National Institute for Health Research (NIHR), and AS was partly funded by the UK NIHR.

DATA SHARING

A full list of health technologies included in the study is available in a supplemental document submitted with this article. More details on data are available from the corresponding author at l.doos@bham.ac.uk.

REFERENCES

1. Herndon JH, Wang RH, Bozic KH. Healthcare technology and technology assessment. *Eur Spine J* 2007;16:1293–1302.
2. Beller GA. New technology and the cost of health care. *J Nucl Cardiol* 2005;12:515-7.
3. Simpson S, Hyde C, Cook A, et al. Assessing the accuracy of forecasting: applying standard diagnostic assessment tools to a health technology early warning system. *Int J Technol Assess Health Care* 2004;20(3):381-4.

For peer review only - http://bmjopen.bmj.com/site/about/guidelines.xhtml

4. Lerner JC, Robertson DC, Goldstein SM. Case studies on forecasting for innovative technologies: Frequent revisions improve accuracy. *Health Aff* 2015; 34(2):311-318.
5. Pankratova N, Bubnov RV. Solving strategic problems of the future on the basis of foresight methodology. *EPMA Journal* 2014;5(Suppl 1) A65-66.
6. Loveridge D, Georghiou L, Nedeva M. *United Kingdom Technology Foresight Programme: Delphi Survey*. A report by the Office of Science and Technology. Manchester, UK: Policy Research in Engineering, Science and Technology (PREST), 1995.
7. Stevens A, Robert N, Gabbay J. Identifying new health care technologies in the United Kingdom. *Int J Technol Assess Health Care* 1997;13:59–67.
8. Tremblay M, Yiu SH. Horizon Scan of Diagnostic Technologies-Technology push or treatment pull? Report published by Rainmaker Publications 2006. http://www.foresightfordevelopment.org/sobipro/55/365-horizon-scan-of-diagnostic-technologies-technology-push-or-treatment-pull (Accessed 2June 2014).
9. Doos L, Packer C, Ward D, Simpson S, Stevens A. Past speculations of the future: A review of the methods used for forecasting emerging health technologies. BMJ Open 2016 6:e010479 doi:10.1136/bmjopen-2015-010479.
10. The National Institute for Health Research Health Technology Assessment (NIHR HTA) programme. http://www.nets.nihr.ac.uk/programmes/hta/remit (Accessed 4 July 2016).
11. World Health Organisation (WHO). International statistical classification of diseases and related health problems. - 10th revision (ICD-10), Fifth edition, 2016. http://apps.who.int/classifications/icd10/browse/2016/en (Accessed 5 July 2016).
12. Rosin RD, Kemp CR. Operating theatre of the year 2010. Report by Department of Trade and Industry. DTI, 1999.
13. BT calendar 2005. Personal communication with the editor Ian Pearson.
14. Institute of the Future_HC2020 _The future of science, technology & wellbeing. http://www.iftf.org/our-work/health-self/health-horizons/hh2010-the-future-of-science-tech-and-well-being/ (Accessed 21 March 2015).
15. 15. Sun F and Schoelles K. A systematic review of methods for health care technology horizon scanning. Agency for Healthcare Research and Quality

For peer review only

For peer review only - http://bmjopen.bmj.com/site/about/guidelines.xhtml

(AHRQ). U.S. Department of Health & Human Services. Research Report – Publication No. 13-EHC104-EF August 2013. Available at https://effectivehealthcare.ahrq.gov/search-for-guides-reviews-and-reports/?pageaction=displayproduct&productID=1679 (Accessed 19 May 2017).

16. 16. Packer C, Fung M, Stevens A. Analyzing 10 Years of Early Awareness and Alert Activity in the United Kingdom. Int J Technol Assess Health Care 2012;28(3):308–314.

17. Berzofsky JA, Ahlers JD, Janik J, et al. Progress on new vaccine strategies against chronic viral infections. *J Clin Invest* Aug 2004;114:4: 450:462.

18. Cahill E, Scapolo F. The Future Project Technology Map. A report by European Commission Directorate General, December 1999.

19. Begley CG, Ellis LM. Raise standards for preclinical cancer research. *Nature* 29 March 2012;483:531-533.

20. Lindvall O, Kokaia Z, Martinez-Serrano A. Stem cell therapy for neurodegenerative disorders-How to make it work. *Nat Med* July 2004 (10); S42-S50.

21. Aboody K, Capela A, Niazi N, et al. Translating stem cell studies to the clinic for CNS repair: Current state of the art and the need for a rosetta stone. *Neuron* May 26, 2011;70:597-613.

For peer review only

Table 1: The 15 forecasting studies identified in the prior systematic review

Study name or first author	Year of forecast	Country
Dutch Steering Committee on Future Health Scenarios (STG) 1988	1986	Netherlands
Spiby, 1988	1988	UK
Loveridge et al 1995	1994	UK
Stevens et al, 1997	1995	UK
Karim, National Research and Technology Foresight Project, 1999	1996	South Africa
Operating theatre of the year 2010; Department of Trade and Industry report 1999	1996	UK
Cahill and Scapolo, 1999	1998	Europe
Daar et al, 2002	2002	Developing countries
Technology foresight towards 2020_China	2003	China
British Telecommunications (BT) calendar	1997 & 2005	United Kingdom (UK)
Tremblay and Yiu, 2006	2006	Canada
Food and Drug Administration (FDA) surveys	1998 & 2008	United States of America (USA)
Institute of the Future, 2009	2009	USA
Science & Technology Foresight Survey, 2010	Every 5 years from 1971, latest survey 2009-10	Japan
UK technology and innovation futures for the 2020s, 2010	2010	UK

Source: Doos et al 2016[9]

For peer review only

Table 2: Health technologies by technology type

**Technology type**	**Number**	**%**
Diagnostic tests and imaging	131	19.1
Devices and biomaterials	98	14.3
IT systems, eHealth and mHealth	86	12.6
Drugs (not prophylactic vaccines)	82	12.0
Regenerative medicine	80	11.7
Medical equipment	37	5.4
Therapeutic procedures	36	5.3
Unknown	33	4.8
Organisational programmes	26	3.8
Prophylactic vaccines	24	3.5
Population programmes	24	3.5
Non-surgical therapy	15	2.2
Other	9	1.3
Assistive devices	4	0.6
**Total**	**685**	**100.0**

For peer review only

For peer review only - http://bmjopen.bmj.com/site/about/guidelines.xhtml

Table 3: Technology type by forecasting group

Technology type	Forecasting group						Total
	Commercial organisation	Consultancy agency	Governmental agency	Independent non-profit organisation	Researchers	Policy research group	
Assistive devices	3 (3.2%)	0 (0.0%)	1 (0.3%)	0 (0.0%)	0 (0.0%)	0 (0.0%)	4 (0.6%)
Devices & biomaterials	16 (16.8%)	6 (10.9%)	56 (15.6%)	3 (17.6%)	7 (11.5%)	10 (10.1)	98 (14.3%)
Diagnostic tests & imaging	6 (6.3%)	26 (47.3%)	63 (17.6%)	3 (17.6%)	9 (14.8%)	24 (24.2%)	131 (19.1)
Drugs (non-prophylactic vaccine)	7 (7.4%)	2 (3.6%)	31 (8.7%)	1 (5.9%)	22 (36.1%)	19 (19.2%)	82 (12.0%)
IT systems, eHealth, mHealth	18 (18.9%)	7 (12.7%)	43 (12.0%)	3 (17.6%)	3 (4.9%)	12 (12.1%)	86 (12.6%)
Medical equipment	2 (2.1%)	5 (5.5%)	31 (8.7%)	0 (0.0%)	1 (1.6%)	0 (0.0%)	37 (5.4%)
Non-surgical therapy	1 (1.1%)	0 (0.0%)	9 (2.5%)	1 (5.9%)	3 (4.9%)	1 (1.0%)	15 (2.2%)
Population programme	5 (5.3%)	0 (0.0%)	9 (2.5%)	3 (17.6%)	1 (1.6%)	6 (6.1%)	24 (3.5%)

For peer review only - http://bmjopen.bmj.com/site/about/guidelines.xhtml

Prophylactic vaccine	0 (0.0%)	0 (0.0%)	18 (5.0%)	0 (0.0%)	0 (0.0%)	6 (6.1%)	24 (3.5%)
Regenerative Medicine	22 (23.2%)	1 (1.8%)	48 (13.4%)	1 (5.9%)	4 (6.6%)	4 (4.0%)	80 (11.7%)
Therapeutic procedure	0 (0.0%)	1 (1.8%)	20 (5.6%)	0 (0.0%)	6 (9.8%)	9 (9.1%)	36 (5.3%)
Organisational programme	2 (2.1%)	10 (10.6%)	11 (3.1%)	1 (5.9%)	4 (6.6%)	2 (2.0%)	26 (3.8%)
Unknown	9 (9.5%)	2 (3.6%)	16 (4.5%)	1 (5.9%)	1 (1.6%)	4 (4.0%)	33 (4.8%)
Others	4 (4.2%)	1 (1.8%)	2 (0.6%)	0 (0.0%)	0 (0.0%)	2 (2.0%)	9 (1.3%)
**Total**	95 (100%)	55 (100%)	358 (100%)	17 (100%)	61 (100%)	99 (100%)	685 (100%)

For peer review only

For peer review only - http://bmjopen.bmj.com/site/about/guidelines.xhtml

Table 4: Technology type and purpose for the five most common ICD_10 chapter headings

**Technology type**	Infectious disease ICD_I	Cancer ICD_II	Circulatory disorders ICD_IX	Nervous system ICD_VI	Health status, screening, rehab ICD_XXI	**Total**
Assistive devices	-	-	-	-	3 (8.6)	**3 (1.5)**
Diagnostic tests and imaging	5 (10.4)	12 (30.0)	7 (18.4)	6 (16.7)	2 (5.7)	**32 (16.2)**
Devices and biomaterials	3 (6.3)	1 (2.5)	10 (26.3)	9 (25.0)	11 (31.4)	**34 (17.3)**
IT systems, eHealth and mHealth	-	-	-	1 (2.8)	3 (8.6)	**4 (2.0)**
Drugs (not prophylactic vaccines)	10 (20.8)	16 (40.0)	7 (18.4)	2 (5.6)	5 (14.3)	**40 (20.3)**
Regenerative medicine	-	2 (5.0)	7 (18.4)	9 (25.0)	-	**18 (9.1)**
Medical equipment	2 (4.2)	-	-	1 (2.8)	1 (2.9)	**4 (2.0)**
Therapeutic procedures	1 (2.1)	1 (2.5)	3 (7.9)	4 (11.1)	-	**9 (4.6)**
Organisational programmes	3 (6.3)	-	-	-	1 (2.9)	**4 (2.0)**
Prophylactic vaccines	22 (45.8)	-	-	-	1 (2.9)	**23 (11.7)**
Population programmes	1 (2.1)	2 (5.0)	2 (5.3)	1 (2.8)	3 (8.6)	**9 (4.6)**
Non-surgical therapy	-	2 (5.0)		1 (2.8)	-	**3 (1.5)**
Other/ unknown	1 (2.1)	4 (10.0)	2 (5.3)	2 (5.6)	5 (14.3)	**14 (7.1)**
**Total**	**48 (100%)**	**40 (100%)**	**38 (100%)**	**36 (100%)**	**35 (100%)**	**197 (100%)**
**Technology purpose**						
Diagnosis & monitoring of disease	7 (14.6)	12 (30.0)	8 (21.1)	7 (19.4)	1 (2.9)	**35 (17.8)**
Health promotion & disease prevention	29 (60.4)	5 (12.5)	5 (13.2)	1 (2.8)	18 (51.4)	**58 (29.4)**

For peer review only - http://bmjopen.bmj.com/site/about/guidelines.xhtml

Treatment & disease management	11 (22.9)	22 (55.0)	25 (65.8)	22 (61.1)	1 (2.9)	**81 (41.1)**
Rehabilitation	0 (0.0)	0 (0.0)	0 (0.0)	4 (11.1)	15 (42.9)	**19 (9.6)**
Other and unknown	1 (2.1)	1 (2.5)	0 (0.0)	2 (5.6)	0 (0.0)	**4 (2.0)**
**Total**	**48 (100%)**	**40 (100%)**	**38 (100%)**	**36 (100%)**	**35 (100%)**	**197 (100%)**

For peer review only

Table5: Mean technology prediction timeframe (years) by technology type*

Technology type	Number	Mean timeframe Years (SD)
Non-surgical therapy	14	9.07 (5.7)
Diagnostic tests and imaging	113	10.11 (6.0)
Drugs (not prophylactic vaccines)	75	10.31 (6.3)
Therapeutic procedures	31	10.45 (8.2)
Organisational programmes	25	10.48 (5.3)
IT systems, eHealth and mHealth	77	11.08 (5.5)
Devices and biomaterials	88	11.93 (6.8)
Assistive devices	3	12.33 (17.9)
Other/ unknown	40	13.35 (7.2)
Medical equipment	36	13.53 (2.7)
Regenerative medicine	73	13.58 (7.4)
Prophylactic vaccines	13	14.00 (6.3)
Population programmes	19	14.53 (5.9)
**Total**	**607**	**11.59 (6.6)**

*p<0.002

Figure 1: Mean forecast timeframe by methods of forecasting*

For peer review only - http://bmjopen.bmj.com/site/about/guidelines.xhtml

Figure 1: Mean forecast timeframe by methods of forecasting*

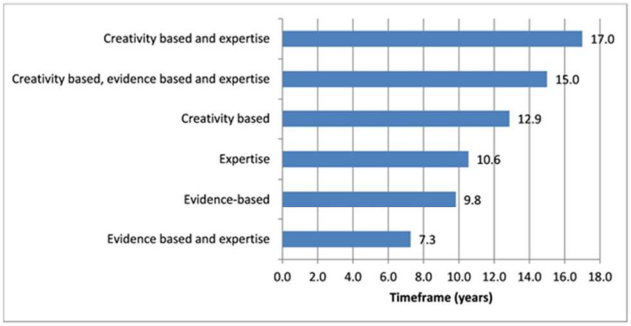

*p<0.001

81x60mm (300 x 300 DPI)

ew only

Appendix1: List of identified healthcare technologies

Report name (year)	Intention of the study	Technology Name	Tech. time frame	Purpose of the technology	Specialty	Technology type
BT Calendar 1997	Commercial	Artificial muscle use in place of heart transplants	1	Treatment and management of symptoms and disease	IX - circulation	Regenerative medicine
BT Calendar 1997	Commercial	Telemed link to home with home blood test kit	1	Diagnosis and monitoring of disease	Other - generic	IT systems, eHealth and mHealth
BT Calendar 1997	Commercial	Artificial intelligence based elderly and handicapped support devices	1	Rehabilitation	XXI - screening, rehab	Assistive devices
BT Calendar 1997	Commercial	Artificial pancreas	1	Treatment and management of symptoms and disease	XI - GI tract, dental health	Regenerative medicine
BT Calendar 1997	Commercial	Artificial blood	3	Treatment and management of symptoms and disease	III - blood	Regenerative medicine
BT Calendar 1997	Commercial	Disability assistance devices using thought recognition	3	Rehabilitation	XXI - screening, rehab	Assistive devices
BT Calendar 1997	Commercial	Artificial ears	3	Rehabilitation	VIII - ear	Regenerative medicine
BT Calendar 1997	Commercial	Electronic implants used to stimulate muscles in disabled people	5	Rehabilitation	XIII - musculoskeletal	Devices and biomaterials
BT Calendar 1997	Commercial	Home (health) diagnostic systems, daily/real time check-up	1	Diagnosis and monitoring of disease	Other - generic	IT systems, eHealth and mHealth
BT Calendar 1997	Commercial	Virtual Reality based exercisers in homes	1	Health promotion and prevention of disease	XXI - screening, rehab	IT systems, eHealth and mHealth
BT Calendar 1997	Commercial	Synthetic retinal implants for simple vision	8	Rehabilitation	VII - eye	Regenerative medicine
BT Calendar 1997	Commercial	Personal wearable health monitor	6	Health promotion and prevention of disease	XXI - screening, rehab	IT systems, eHealth and mHealth
BT Calendar 1997	Commercial	Artificial heart	13	Treatment and management of symptoms and disease	IX - circulation	Regenerative medicine

For peer review only - http://bmjopen.bmj.com/site/about/guidelines.xhtml

Appendix1: List of identified healthcare technologies

BT Calendar 1997	Commercial	Genetic screening widely used	10	Health promotion and prevention of disease	XXI - screening, rehab	Population programmes
BT Calendar 1997	Commercial	Fine particle beam gene engineering	12	Treatment and management of symptoms and disease	Other - generic	Regenerative medicine
BT Calendar 1997	Commercial	Artificial senses, sensors directly stimulating nerves	15	Rehabilitation	XXI - screening, rehab	Devices and biomaterials
BT Calendar 1997	Commercial	Prevention of cancer	16	Health promotion and prevention of disease	II - cancers	Population programmes
BT Calendar 1997	Commercial	Custom foods for particular medical conditions will exist.	13	Treatment and management of symptoms and disease	Other - generic	Other
BT Calendar 1997	Commercial	Genetic programs to enhance human well-being	23	Health promotion and prevention of disease	V - mental health	Population programmes
BT Calendar 1997	Commercial	Artificial lungs	18	Treatment and management of symptoms and disease	X - respiratory	Regenerative medicine
BT Calendar 1997	Commercial	Artificial kidneys	18	Treatment and management of symptoms and disease	XIV - genitourinary	Regenerative medicine
BT Calendar 1997	Commercial	Artificial brain cells	20	Treatment and management of symptoms and disease	VI - nervous	Regenerative medicine
BT Calendar 1997	Commercial	Artificial liver	23	Treatment and management of symptoms and disease	XI - GI tract, dental health	Regenerative medicine
BT Calendar 1997	Commercial	Extension of average lifespan to over 100	23	Health promotion and prevention of disease	XXI - screening, rehab	Unknown
BT Calendar 1997	Commercial	Artificial brain implants	28	Treatment and management of symptoms and disease	VI - nervous	Regenerative medicine
BT Calendar 1997	Commercial	Artificial peripheral nerves	28	Treatment and management of symptoms and disease	VI - nervous	Regenerative medicine
BT Calendar 1997	Commercial	Artificial legs	33	Rehabilitation	XXI - screening, rehab	Unknown

For peer review only - http://bmjopen.bmj.com/site/about/guidelines.xhtml

Appendix1: List of identified healthcare technologies

BT Calendar 1997	Commercial	Fully functional artificial eyes	33	Treatment and management of symptoms and disease	VII - eye	Regenerative medicine
BT Calendar 2005	Commercial	laser activated drug capsules	3	Treatment and management of symptoms and disease	Other - generic	drugs (not prophylactic vaccines)
BT Calendar 2005	Commercial	ultrasound or radio activated capsules	3	Treatment and management of symptoms and disease	Other - generic	drugs (not prophylactic vaccines)
BT Calendar 2005	Commercial	blood analysis chips	3	Diagnosis and monitoring of disease	Other - generic	Diagnostic tests and imaging
BT Calendar 2005	Commercial	e-Baybies electronic conception	5	Health promotion and prevention of disease	XXI - screening, rehab	Unknown
BT Calendar 2005	Commercial	Instant electronic identification of pathogens	5	Diagnosis and monitoring of disease	I - infections	Diagnostic tests and imaging
BT Calendar 2005	Commercial	Online surgeries dominate first line medical care	5	Supporting the provision of care and/or increasing service efficiency	Other - generic	IT systems, eHealth and mHealth
BT Calendar 2005	Commercial	active skin	5	Unknown	XII - skin	Unknown
BT Calendar 2005	Commercial	Cyber-drugs (electronically activated drugs)	5	Treatment and management of symptoms and disease	Other - generic	Drugs (not prophylactic vaccines)
BT Calendar 2005	Commercial	automated pain relief for soldiers	5	Treatment and management of symptoms and disease	XIX - injury	Devices and biomaterials
BT Calendar 2005	Commercial	bacteria in toothpaste to attack plaque	5	Health promotion and prevention of disease	XI - GI tract, dental health	Non-surgical therapy
BT Calendar 2005	Commercial	e-baybies, binary conception	5	Health promotion and prevention of disease	XXI - screening, rehab	Unknown
BT Calendar 2005	Commercial	hand-held scanner to detect tumours using tissue resonance interferometer	5	Diagnosis and monitoring of disease	II - cancers	Diagnostic tests and imaging
BT Calendar 2005	Commercial	smart pill bottles remotely monitor medication taking and use alarms	5	Supporting patients receiving care (designed to be used by individuals)	Other - generic	IT systems, eHealth and mHealth

For peer review only - http://bmjopen.bmj.com/site/about/guidelines.xhtml

Appendix1: List of identified healthcare technologies

BT Calendar 2005	Commercial	diabetes cure via stem cell research	5	Treatment and management of symptoms and disease	IV - endocrine	Regenerative medicine
BT Calendar 2005	Commercial	Use of human's own tissues to grow replacement organs	8	Treatment and management of symptoms and disease	Other - generic	Regenerative medicine
BT Calendar 2005	Commercial	Artificial senses	8	Rehabilitation	XXI - screening, rehab	Devices and biomaterials
BT Calendar 2005	Commercial	context sensitive cyber drugs	8	Treatment and management of symptoms and disease	Other - generic	Unknown
BT Calendar 2005	Commercial	electronic stimulation of brain sensations as recreational substitute for drugs	8	Other	VI - nervous	Devices and biomaterials
BT Calendar 2005	Commercial	Custom (GM) foods for particular medical conditions will exist.	8	Treatment and management of symptoms and disease	Other - generic	Other
BT Calendar 2005	Commercial	Gene-gel stimulation of re-growth of natural teeth on demand	8	Treatment and management of symptoms and disease	XI - GI tract, dental health	drugs (not prophylactic vaccines)
BT Calendar 2005	Commercial	retina regeneration using foetal retinal cell injection	8	Treatment and management of symptoms and disease	VII - eye	Regenerative medicine
BT Calendar 2005	Commercial	tooth regeneration	8	Treatment and management of symptoms and disease	XI - GI tract, dental health	Regenerative medicine
BT Calendar 2005	Commercial	plastic bones	8	Treatment and management of symptoms and disease	XIII - musculoskeletal	Devices and biomaterials
BT Calendar 2005	Commercial	emotionally specific drugs	8	Unknown	V - mental health	drugs (not prophylactic vaccines)
BT Calendar 2005	Commercial	synthetic organs by printing layers of cells	10	Treatment and management of symptoms and disease	Other - generic	Regenerative medicine
BT Calendar 2005	Commercial	synthetic viruses	10	Unknown	I -	Other

For peer review only - http://bmjopen.bmj.com/site/about/guidelines.xhtml

Appendix1: List of identified healthcare technologies

					infections	
BT Calendar 2005	Commercial	sensory augmentation using sensory implants, nanoparticles etc	10	Rehabilitation	VI - nervous	Devices and biomaterials
BT Calendar 2005	Commercial	use of stem cells in brain after strokes or accidents	10	Treatment and management of symptoms and disease	VI - nervous	Regenerative medicine
BT Calendar 2005	Commercial	gene therapy generates new hair cells in humans	10	Treatment and management of symptoms and disease	XII - skin	drugs (not prophylactic vaccines)
BT Calendar 2005	Commercial	artificial sensors used in cosmetic upgrade surgery	13	Treatment and management of symptoms and disease	Other - generic	Devices and biomaterials
BT Calendar 2005	Commercial	Electronic memory enhancement	20	Unknown	Other - generic	Unknown
BT Calendar 2005	Commercial	Nanobots in toothpaste attack plaque	20	Health promotion and prevention of disease	XI - GI tract, dental health	drugs (not prophylactic vaccines)
BT Calendar 2005	Commercial	Fully functioning artificial eyes	20	Treatment and management of symptoms and disease	VII - eye	Regenerative medicine
BT Calendar 2005	Commercial	electronic brain implants	20	Treatment and management of symptoms and disease	VI - nervous	Devices and biomaterials
BT Calendar 2005	Commercial	Intelligence or memory enhancement by external means	20	Unknown	Other - generic	Unknown
BT Calendar 2005	Commercial	e-baybies, genetic conception	20	Health promotion and prevention of disease	XXI - screening, rehab	Unknown
BT Calendar 2005	Commercial	synthetic immune system	20	Treatment and management of symptoms and disease	III - blood	Regenerative medicine
BT Calendar 2005	Commercial	Artificial peripheral nerves	30	Treatment and management of symptoms and disease	VI - nervous	Regenerative medicine
BT Calendar 2005	Commercial	e-baybies, digital emulation conception	30	Health promotion and prevention of disease	XXI - screening, rehab	Unknown

For peer review only - http://bmjopen.bmj.com/site/about/guidelines.xhtml

Appendix1: List of identified healthcare technologies

BT Calendar 2005	Commercial	Electronic prescriptions reduce fraud and improve speed	3	Supporting the provision of care and/or increasing service efficiency	Other - generic	IT systems, eHealth and mHealth
BT Calendar 2005	Commercial	24/7 blood chemistry monitoring	3	Diagnosis and monitoring of disease	Other - generic	Diagnostic tests and imaging
BT Calendar 2005	Commercial	supermarkets used as major source of medical alerts	3	Health promotion and prevention of disease	Other - generic	Other
BT Calendar 2005	Commercial	Expert systems used extensively in GP surgeries	5	Supporting patients receiving care (designed to be used by individuals)	Other - generic	IT systems, eHealth and mHealth
BT Calendar 2005	Commercial	Hospitals use virtual queuing systems	5	Supporting the provision of care and/or increasing service efficiency	Other - generic	IT systems, eHealth and mHealth
BT Calendar 2005	Commercial	Neural networks used for patient appointment management	5	Supporting the provision of care and/or increasing service efficiency	Other - generic	IT systems, eHealth and mHealth
BT Calendar 2005	Commercial	Multimedia patient records	5	Supporting patients receiving care (designed to be used by individuals)	Other - generic	IT systems, eHealth and mHealth
BT Calendar 2005	Commercial	Lifestyle monitoring and insurance linked to medical records	5	Health promotion and prevention of disease	Other - generic	Population programmes
BT Calendar 2005	Commercial	antibacterial coatings on domestic appliances, phones etc, especially in hospitals	5	Health promotion and prevention of disease	I - infections	Devices and biomaterials
BT Calendar 2005	Commercial	flexible displays used for body monitoring and alerts	5	Diagnosis and monitoring of disease	Other - generic	IT systems, eHealth and mHealth
BT Calendar 2005	Commercial	emotion alerts	5	Health promotion and prevention of disease	Other - generic	Devices and biomaterials
BT Calendar 2005	Commercial	(drug shortages caused by) internet diagnosis	5	Diagnosis and monitoring of disease	Other - generic	IT systems, eHealth and mHealth

For peer review only - http://bmjopen.bmj.com/site/about/guidelines.xhtml

Appendix1: List of identified healthcare technologies

BT Calendar 2005	Commercial	MRSA detection chips on sale in high street	5	Diagnosis and monitoring of disease	I - infections	Population programmes
BT Calendar 2005	Commercial	Devices roaming within blood vessels under own power	8	Unknown	Other - generic	Devices and biomaterials
BT Calendar 2005	Commercial	Operations videoed and stored as part of medical record	8	Supporting patients receiving care (designed to be used by individuals)	Other - generic	IT systems, eHealth and mHealth
BT Calendar 2005	Commercial	Direct electronic pleasure production	8	Other	V - mental health	IT systems, eHealth and mHealth
BT Calendar 2005	Commercial	Orgasm by email	8	Supporting patients receiving care (designed to be used by individuals)	XIV - genitourinary	IT systems, eHealth and mHealth
BT Calendar 2005	Commercial	Artificial senses, sensors directly stimulating nerves	8	Rehabilitation	XXI - screening, rehab	Devices and biomaterials
BT Calendar 2005	Commercial	Smart membranes for remote control of drug injection	8	Treatment and management of symptoms and disease	Other - generic	Devices and biomaterials
BT Calendar 2005	Commercial	Domestic bacterial detection devices	8	Health promotion and prevention of disease	I - infections	Diagnostic tests and imaging
BT Calendar 2005	Commercial	Micro-fluidic chips used for gene sequencing in every GP surgery	8	Diagnosis and monitoring of disease	Other - generic	Diagnostic tests and imaging
BT Calendar 2005	Commercial	Self certification for prescriptions using electronic diagnostics	8	Supporting patients receiving care (designed to be used by individuals)	Other - generic	IT systems, eHealth and mHealth
BT Calendar 2005	Commercial	Visitor sanitising in hospitals	8	Health promotion and prevention of disease	I - infections	Organisational programmes
BT Calendar 2005	Commercial	Outpatients at home - remote tele-medical consultations	8	Supporting patients receiving care (designed to be used by individuals)	Other - generic	IT systems, eHealth and mHealth
BT Calendar 2005	Commercial	Individual's genome part of their medical record	8	Supporting patients receiving care (designed to be used by	Other - generic	Organisational programmes

For peer review only - http://bmjopen.bmj.com/site/about/guidelines.xhtml

Appendix1: List of identified healthcare technologies

				individuals)		
BT Calendar 2005	Commercial	Portable bactericidal devices	10	Health promotion and prevention of disease	I - infections	Medical equipment
BT Calendar 2005	Commercial	Bactericidal saws incorporated onto materials in hospitals	10	Health promotion and prevention of disease	I - infections	Devices and biomaterials
BT Calendar 2005	Commercial	Robotic cleaners in hospitals	10	Supporting the provision of care and/or increasing service efficiency	Other - generic	Medical equipment
Daar et al 2002	Research	Modified molecular technologies for affordable, simple diagnosis of infectious diseases	8	Diagnosis and monitoring of disease	I - infections	Diagnostic tests and imaging
Daar et al 2002	Research	Recombinant technologies to develop vaccines against infectious diseases	8	Health promotion and prevention of disease	I - infections	Prophylactic vaccines
Daar et al 2002	Research	Technologies for more efficient drug and vaccine delivery systems	8	Treatment and management of symptoms and disease	Other - generic	Unknown
Daar et al 2002	Research	Technologies for environmental improvement (sanitation, clean water, bioremediation)	8	Health promotion and prevention of disease	Other - generic	Unknown
Daar et al 2002	Research	Female-controlled protection against sexually transmitted diseases, both with and without contraceptive effect	8	Health promotion and prevention of disease	XIV - genitourinary	Devices and biomaterials
Daar et al 2002	Research	Genetically modified crops with increased nutrients to counter specific deficiencies	8	Health promotion and prevention of disease	IV - endocrine	Unknown

For peer review only - http://bmjopen.bmj.com/site/about/guidelines.xhtml

Appendix1: List of identified healthcare technologies

Daar et al 2002	Research	Recombinant technology to make therapeutic products (for example, insulin, interferons) more affordable	8	Treatment and management of symptoms and disease	Other - generic	drugs (not prophylactic vaccines)
Karim2000	Policy planning	Telemedicine	15	Supporting patients receiving care (designed to be used by individuals)	Other - generic	IT systems, eHealth and mHealth
Karim2000	Policy planning	Medical databases	15	Supporting the provision of care and/or increasing service efficiency	Other - generic	IT systems, eHealth and mHealth
Karim2000	Policy planning	Expert System in managed care and preventive medicine	15	Supporting patients receiving care (designed to be used by individuals)	Other - generic	IT systems, eHealth and mHealth
Karim2000	Policy planning	diagnostic procedures (e.g. recombinant DNA techniques for parental screening)	15	Diagnosis and monitoring of disease	Other - generic	Diagnostic tests and imaging
Karim2000	Policy planning	Therapeutic procedures (e.g. microsurgery and implanted muscle stimulators).	15	Treatment and management of symptoms and disease	Other - generic	Therapeutic procedures
Karim2000	Policy planning	Nanotechnology (e.g. devices which interact with tissues within the body to boost the immune system).	15	Treatment and management of symptoms and disease	III - blood	Devices and biomaterials
Karim2000	Policy planning	Vaccines against infectious diseases	15	Health promotion and prevention of disease	I - infections	Prophylactic vaccines
Karim2000	Policy planning	Development of an AIDS vaccine	15	Health promotion and prevention of disease	I - infections	Prophylactic vaccines
Karim2000	Policy planning	Development of a TB vaccine	15	Health promotion and prevention of disease	I - infections	Prophylactic vaccines
Karim2000	Policy planning	Health promotion targeted at the youth	15	Health promotion and prevention of disease	Other - generic	Population programmes

For peer review only - http://bmjopen.bmj.com/site/about/guidelines.xhtml

Appendix1: List of identified healthcare technologies

Karim2000	Policy planning	Development of enriched and fortified staple foods	15	Health promotion and prevention of disease	Other - generic	Population programmes
Karim2000	Policy planning	Development of a malaria vaccine	15	Health promotion and prevention of disease	I - infections	Prophylactic vaccines
Karim2000	Policy planning	Development of on-site diagnostic tools	15	Diagnosis and monitoring of disease	Other - generic	Diagnostic tests and imaging
Karim2000	Policy planning	Development of female contraceptives	15	Health promotion and prevention of disease	XXI - screening, rehab	drugs (not prophylactic vaccines)
Karim2000	Policy planning	Development of barrier methods and microbicides for sexually transmitted diseases and HIV	15	Health promotion and prevention of disease	I - infections	Devices and biomaterials
Karim2000	Policy planning	Development of malaria drugs	15	Treatment and management of symptoms and disease	I - infections	drugs (not prophylactic vaccines)
Karim2000	Policy planning	Development of new TB drugs	15	Treatment and management of symptoms and disease	I - infections	drugs (not prophylactic vaccines)
Karim2000	Policy planning	Self management tools for chronic diseases (e.g. hypertension, diabetes, diarrhoea, psychiatric conditions)	15	Supporting patients receiving care (designed to be used by individuals)	Other - generic	Organisational programmes
Karim2000	Policy planning	Tissue regeneration and gene therapy	15	Treatment and management of symptoms and disease	Other - generic	Regenerative medicine
Sbipy 1988	Research	Monoclonal antibodies will be used in: Histopathological techniques	0	Diagnosis and monitoring of disease	Other - generic	Diagnostic tests and imaging
Sbipy 1988	Research	Monoclonal antibodies will be used in: Biochemical techniques	0	Diagnosis and monitoring of disease	Other - generic	Diagnostic tests and imaging
Sbipy 1988	Research	Monoclonal antibodies will be	0	Diagnosis and monitoring of	Other - generic	Diagnostic tests and imaging

For peer review only

Appendix1: List of identified healthcare technologies

		used in: in-vivo diagnostic techniques		disease		
Sbipy 1988	Research	Monoclonal antibodies will be used in: Identification and localization of tumours	0	Diagnosis and monitoring of disease	II - cancers	Diagnostic tests and imaging
Sbipy 1988	Research	Gene probes will be used in: diagnostic microbiology	0	Diagnosis and monitoring of disease	I - infections	Diagnostic tests and imaging
Sbipy 1988	Research	Gene probes will be used in: diagnostic virology	0	Diagnosis and monitoring of disease	I - infections	Diagnostic tests and imaging
Sbipy 1988	Research	Gene probes will be used in: To screen for potentially deleterious genes*	0	Diagnosis and monitoring of disease	Other - generic	Diagnostic tests and imaging
Sbipy 1988	Research	Gene probes will be used in: Diagnosing most genetic disorders	10	Diagnosis and monitoring of disease	XVII - congenital	Diagnostic tests and imaging
Sbipy 1988	Research	Genetic engineering will enable the isolation of high risk groups for common diseases	10	Health promotion and prevention of disease	Other - generic	Population programmes
Sbipy 1988	Research	Imaging techniques will be in widespread and less hazardous use including: Ultrasound	0	Diagnosis and monitoring of disease	Other - generic	Diagnostic tests and imaging
Sbipy 1988	Research	Imaging techniques will be in widespread and less hazardous use including: Doppler measurement	0	Diagnosis and monitoring of disease	Other - generic	Diagnostic tests and imaging
Sbipy 1988	Research	Imaging techniques will be in widespread and less hazardous use including: CT scanning	0	Diagnosis and monitoring of disease	Other - generic	Diagnostic tests and imaging

For peer review only - http://bmjopen.bmj.com/site/about/guidelines.xhtml

Appendix1: List of identified healthcare technologies

Sbipy 1988	Research	Imaging techniques will be in widespread and less hazardous use including: NMR scanning	0	Diagnosis and monitoring of disease	Other - generic	Diagnostic tests and imaging
Sbipy 1988	Research	Imaging techniques will be in widespread and less hazardous use including: Nuclear medicine	0	Diagnosis and monitoring of disease	Other - generic	Diagnostic tests and imaging
Sbipy 1988	Research	Imaging techniques will be in widespread and less hazardous use including: Positron detection*	0	Diagnosis and monitoring of disease	Other - generic	Diagnostic tests and imaging
Sbipy 1988	Research	Imaging techniques will be in widespread and less hazardous use including: Three-dimensional dynamic imaging	10	Diagnosis and monitoring of disease	Other - generic	Diagnostic tests and imaging
Sbipy 1988	Research	Drug therapy will be enhanced by: Genetic engineering	10	Treatment and management of symptoms and disease	Other - generic	drugs (not prophylactic vaccines)
Sbipy 1988	Research	Drug therapy will be enhanced by: Monoclonal antibodies used for drug targeting	10	Treatment and management of symptoms and disease	Other - generic	drugs (not prophylactic vaccines)
Sbipy 1988	Research	Drug delivery devices with or without in-vivo sensors	10	Treatment and management of symptoms and disease	Other - generic	Devices and biomaterials
Sbipy 1988	Research	More effective Therapeutics will be available for: Viral infections	0	Treatment and management of symptoms and disease	I - infections	drugs (not prophylactic vaccines)
Sbipy 1988	Research	More effective Therapeutics will be available for: Autoimmune diseases (monoclonal antibodies)	10	Treatment and management of symptoms and disease	III - blood	drugs (not prophylactic vaccines)
Sbipy 1988	Research	More effective Therapeutics will	0	Treatment and management of	IX - circulation	drugs (not prophylactic

For peer review only - http://bmjopen.bmj.com/site/about/guidelines.xhtml

Appendix1: List of identified healthcare technologies

		be available for: Heart failure (better drugs)		symptoms and disease		vaccines)
Sbipy 1988	Research	More effective Therapeutics will be available for: Obstructive arterial lesions (by removal and control of platelets and synthetic vascular prostheses)	10	Treatment and management of symptoms and disease	IX - circulation	drugs (not prophylactic vaccines)
Sbipy 1988	Research	Cancer (hyperthermia, growth regulation factors and monoclonal antibodies)	10	Other	II - cancers	Other
Sbipy 1988	Research	Cardiac arrhythmias (implantable mechanisms)	10	Treatment and management of symptoms and disease	IX - circulation	Devices and biomaterials
Sbipy 1988	Research	More effective Therapeutics will be available for: Arthritic joints (more biocompatible prosthetic materials and a wider range of joint replacements)	0	Treatment and management of symptoms and disease	XIII - musculoskeletal	Devices and biomaterials
Sbipy 1988	Research	More effective Therapeutics will be available for: Incontinence (stimulation via implantable electrodes)	0	treatment and management of symptoms and disease	XIV - genitourinary	Devices and biomaterials
Sbipy 1988	Research	More effective Therapeutics will be available for: Disability (wider range of flow technology aids and biological servomechanisms linked to computers and	0	Rehabilitation	Other - generic	Other

For peer review only - http://bmjopen.bmj.com/site/about/guidelines.xhtml

Appendix1: List of identified healthcare technologies

		robotics)				
Sbipy 1988	Research	More effective Therapeutics will be available for: Limb damage (permanent internal bone repair)	0	Treatment and management of symptoms and disease	XIII - musculoskeletal	Devices and biomaterials
Sbipy 1988	Research	More effective Therapeutics will be available for: Tropical parasitic diseases (vaccines)	0	Health promotion and prevention of disease	I - infections	Prophylactic vaccines
Sbipy 1988	Research	More effective Therapeutics will be available for: Mental disorders (neurotransmitters)	10	Treatment and management of symptoms and disease	V - mental health	drugs (not prophylactic vaccines)
Sbipy 1988	Research	Laser microsurgery	0	Treatment and management of symptoms and disease	Other - generic	Therapeutic procedures
Sbipy 1988	Research	Laser endoscopic surgery	0	Treatment and management of symptoms and disease	Other - generic	Therapeutic procedures
Sbipy 1988	Research	Laser angioplasty and angiography	0	Treatment and management of symptoms and disease	IX - circulation	Therapeutic procedures
Sbipy 1988	Research	Other laser and endoscopic surgical techniques	10	Treatment and management of symptoms and disease	Other - generic	Therapeutic procedures
Sbipy 1988	Research	Cryosurgery	10	Treatment and management of symptoms and disease	Other - generic	Therapeutic procedures
Sbipy 1988	Research	Lithotripsy	10	Treatment and management of symptoms and disease	Other - generic	Therapeutic procedures
Sbipy 1988	Research	Transplantation will be enhanced by better techniques	0	Treatment and management of symptoms and disease	Other - generic	Devices and biomaterials

For peer review only - http://bmjopen.bmj.com/site/about/guidelines.xhtml

Appendix1: List of identified healthcare technologies

		enabling long term in-vitro organ preservation and the prevention of rejection				
Sbipy 1988	Research	Bone marrow transplantation with purified stem cells and autologous material will be available	0	Treatment and management of symptoms and disease	III - blood	Regenerative medicine
Sbipy 1988	Research	A safe male pill for long-term use will be available	10	Health promotion and prevention of disease	XXI - screening, rehab	drugs (not prophylactic vaccines)
Sbipy 1988	Research	Improved techniques for detecting ovulation will enhance natural family planning*	0	Diagnosis and monitoring of disease	XIV - genitourinary	Diagnostic tests and imaging
Sbipy 1988	Research	Optical disc storage and communication will be used with X-rays and other diagnostic images	0	Supporting the provision of care and/or increasing service efficiency	Other - generic	Diagnostic tests and imaging
Sbipy 1988	Research	Computers will be used by clinical departments for data collection	10	Supporting the provision of care and/or increasing service efficiency	Other - generic	IT systems, eHealth and mHealth
Sbipy 1988	Research	Expert interrogation will be possible due to data centralization	0	Supporting the provision of care and/or increasing service efficiency	Other - generic	IT systems, eHealth and mHealth
Sbipy 1988	Research	Patient interviewing systems will be available in the surgery and in patients' homes	10	Supporting patients receiving care (designed to be used by individuals)	Other - generic	IT systems, eHealth and mHealth
Sbipy 1988	Research	Expert systems will enable diagnosis and define the most applicable treatment	10	Supporting patients receiving care (designed to be used by individuals)	Other - generic	IT systems, eHealth and mHealth

For peer review only - http://bmjopen.bmj.com/site/about/guidelines.xhtml

Appendix1: List of identified healthcare technologies

Sbipy 1988	Research	Linkage of computerized patient data will be available between all hospital, community and primary care services and settings	10	Supporting the provision of care and/or increasing service efficiency	Other - generic	IT systems, eHealth and mHealth
STG 1988	Policy planning	Life style changing behaviours	N/A	Health promotion and prevention of disease	Other - generic	Population programmes
STG 1988	Policy planning	Screening for phenylketonuria	N/A	Health promotion and prevention of disease	IV - endocrine	Population programmes
STG 1988	Policy planning	Screening for congenital hypothyroidism	N/A	Health promotion and prevention of disease	IV - endocrine	Population programmes
STG 1988	Policy planning	Blood pressure can be checked by self-service meters	N/A	Diagnosis and monitoring of disease	IX - circulation	Diagnostic tests and imaging
STG 1988	Policy planning	High serum cholesterol can be checked by fingerprick blood test	N/A	Diagnosis and monitoring of disease	IX - circulation	Diagnostic tests and imaging
STG 1988	Policy planning	Certain cancer tests can be self-administered	N/A	Diagnosis and monitoring of disease	II - cancers	Diagnostic tests and imaging
STG 1988	Policy planning	Improved diagnostic tests may be available for widespread use for screening at the community level	N/A	Health promotion and prevention of disease	Other - generic	Population programmes
STG 1988	Policy planning	Improved vaccine against cholera in the tropical developing countries	N/A	Health promotion and prevention of disease	I - infections	Prophylactic vaccines
STG 1988	Policy planning	New malaria vaccine in the tropical developing countries	N/A	Health promotion and prevention of disease	I - infections	Prophylactic vaccines
STG 1988	Policy planning	New vaccine against hepatitus B	N/A	Health promotion and prevention of disease	I - infections	Prophylactic vaccines

For peer review only - http://bmjopen.bmj.com/site/about/guidelines.xhtml

Appendix1: List of identified healthcare technologies

STG 1988	Policy planning	New vaccine against respiratory syncytial virus	N/A	Health promotion and prevention of disease	I - infections	Prophylactic vaccines
STG 1988	Policy planning	New vaccine against influenza	N/A	Health promotion and prevention of disease	I - infections	Prophylactic vaccines
STG 1988	Policy planning	New vaccine against AIDS	N/A	Health promotion and prevention of disease	I - infections	Prophylactic vaccines
STG 1988	Policy planning	New vaccine against cytomegalovirus	N/A	Health promotion and prevention of disease	I - infections	Prophylactic vaccines
STG 1988	Policy planning	Better vaccine against Perussis	N/A	Health promotion and prevention of disease	I - infections	Prophylactic vaccines
STG 1988	Policy planning	Oral administration or administartion by nasal spray will facilitate mass immunization	N/A	Health promotion and prevention of disease	I - infections	Prophylactic vaccines
STG 1988	Policy planning	Creating vaccines containing antigens for several organisms given together (e.g. DPTPM)	N/A	Health promotion and prevention of disease	I - infections	Prophylactic vaccines
STG 1988	Policy planning	Shifts in diet toward lower intake of fat especially saturated fat	N/A	Health promotion and prevention of disease	Other - generic	Population programmes
STG 1988	Policy planning	Reliable and simple test for cholesterol	N/A	Diagnosis and monitoring of disease	IX - circulation	Diagnostic tests and imaging
STG 1988	Policy planning	More effective and safer drugs for treatment of elevated cholesterol	N/A	Health promotion and prevention of disease	IX - circulation	drugs (not prophylactic vaccines)
STG 1988	Policy planning	Technologies for home use without professional supervision (e.g. home diagnostic kits)	N/A	Supporting patients receiving care (designed to be used by individuals)	Other - generic	Diagnostic tests and imaging
STG 1988	Policy planning	Disease conditions will be monitored from distant professional sites	N/A	Supporting patients receiving care (designed to be used by	Other - generic	IT systems, eHealth and mHealth

For peer review only

For peer review only - http://bmjopen.bmj.com/site/about/guidelines.xhtml

Appendix1: List of identified healthcare technologies

				individuals)		
STG 1988	Policy planning	Robotics will be used for mobility and assistance at home such as nutrition and cleaning	N/A	Rehabilitation	Other - generic	Assistive devices
STG 1988	Policy planning	Regeneration of central nervous system through the use of nerve growth factors or transplant neurotransmitter-producing cells into the brain	N/A	Treatment and management of symptoms and disease	VI - nervous	Regenerative medicine
STG 1988	Policy planning	Grafting of the spinal cord or the peripheral nervous system	N/A	Treatment and management of symptoms and disease	VI - nervous	Therapeutic procedures
STG 1988	Policy planning	More safer & effective contraception	N/A	Health promotion and prevention of disease	XXI - screening, rehab	Drugs (not prophylactic vaccines)
STG 1988	Policy planning	Contraceptive vaccine	N/A	Health promotion and prevention of disease	XXI - screening, rehab	Prophylactic vaccines
STG 1988	Policy planning	Alternatives to surgical abortion such as combination of antiprogestins and prostaglandins	N/A	Treatment and management of symptoms and disease	XV - pregnancy	drugs (not prophylactic vaccines)
STG 1988	Policy planning	Treatment of infertility using new technologies such as in vitro fertilization, surrogate motherhood, freezing of germ cells and embro with embryo transfer	N/A	Treatment and management of symptoms and disease	XIV - genitourinary	Other
STG 1988	Policy planning	Fetal cells can be isolated from the mother's bloodstream as a method for prenatal screening	N/A	Health promotion and prevention of disease	XXI - screening, rehab	Diagnostic tests and imaging

For peer review only - http://bmjopen.bmj.com/site/about/guidelines.xhtml

Appendix1: List of identified healthcare technologies

STG 1988	Policy planning	Metabolic, physiologic information can be obtained by spectroscopy	N/A	Diagnosis and monitoring of disease	Other - generic	Diagnostic tests and imaging
STG 1988	Policy planning	Data from CT scanner will be stored on a disk, transmit by a phone line or othe communication	N/A	Supporting patients receiving care (designed to be used by individuals)	Other - generic	Diagnostic tests and imaging
STG 1988	Policy planning	Film may disappear and computer will directly interpret the diagnosis	N/A	Supporting patients receiving care (designed to be used by individuals)	Other - generic	Diagnostic tests and imaging
STG 1988	Policy planning	Biosensors to monitor disease condition or results of therapy (e.g. diabetes)	N/A	Diagnosis and monitoring of disease	Other - generic	Diagnostic tests and imaging
STG 1988	Policy planning	Tumor markers that can be measured in blood	N/A	Diagnosis and monitoring of disease	II - cancers	Diagnostic tests and imaging
STG 1988	Policy planning	Flow cytometer will be used in transplantation or monitoring of cancer therapy	N/A	Diagnosis and monitoring of disease	II - cancers	Diagnostic tests and imaging
STG 1988	Policy planning	Monoclonal antibodies for diagnosis such as colon cancer and area of cardiac infarctions	N/A	Diagnosis and monitoring of disease	Other - generic	Diagnostic tests and imaging
STG 1988	Policy planning	Human gene therapy for a wide variety of diseases including cancer, diabetes, cardiovascular and affective disorders such as depression	N/A	Treatment and management of symptoms and disease	Other - generic	Regenerative medicine
STG 1988	Policy planning	DNA probe for intra-uterine diagnosis of genetic diseases	N/A	Diagnosis and monitoring of disease	XVII - congenital	Diagnostic tests and imaging
STG 1988	Policy planning	Home diagnostic kits for sexually	7	Diagnosis and monitoring of	I - infections	Organisational programmes

For peer review only - http://bmjopen.bmj.com/site/about/guidelines.xhtml

Appendix1: List of identified healthcare technologies

		transmitted diseases and hepatitits		disease		
STG 1988	Policy planning	Home diagnostic kits for screening for cancer	7	Diagnosis and monitoring of disease	I - infections	Organisational programmes
STG 1988	Policy planning	Home diagnostic kits to test for common diseases with genetic basis such as diabetes	N/A	Diagnosis and monitoring of disease	Other - generic	Organisational programmes
STG 1988	Policy planning	Genetic insertion therapy for treatment of certain blood disorders such as thalassaemia and sickle cell anaemia	N/A	Treatment and management of symptoms and disease	III - blood	Regenerative medicine
STG 1988	Policy planning	Oral treatment forms for hormones, enzymes and other peptides may be developed	N/A	Treatment and management of symptoms and disease	Other - generic	Drugs (not prophylactic vaccines)
STG 1988	Policy planning	New drugs for asthma	N/A	Treatment and management of symptoms and disease	X - respiratory	Drugs (not prophylactic vaccines)
STG 1988	Policy planning	New drugs for coronary artery diseases	N/A	Treatment and management of symptoms and disease	IX - circulation	Drugs (not prophylactic vaccines)
STG 1988	Policy planning	Monoclonal antibodies to replace immune globulins	N/A	Treatment and management of symptoms and disease	III - blood	Drugs (not prophylactic vaccines)
STG 1988	Policy planning	Cloning of skin	N/A	Treatment and management of symptoms and disease	XII - skin	Regenerative medicine
STG 1988	Policy planning	Growth of retinal tissue and corneal endothelium	N/A	Treatment and management of symptoms and disease	VII - eye	Regenerative medicine
STG 1988	Policy planning	Total body irradiation for transplant (to prevent rejection)	N/A	Treatment and management of symptoms and disease	III - blood	Non-surgical therapy
STG 1988	Policy planning	Artificial heart	N/A	Treatment and management of symptoms and	IX - circulation	Regenerative medicine

For peer review only

Appendix1: List of identified healthcare technologies

				disease		
STG 1988	Policy planning	Artificial pancreas	N/A	Treatment and management of symptoms and disease	IV - endocrine	Regenerative medicine
STG 1988	Policy planning	Shoulder joint replacement	N/A	Treatment and management of symptoms and disease	XIII - musculoskeletal	Devices and biomaterials
STG 1988	Policy planning	Cochlear implants	N/A	Treatment and management of symptoms and disease	VIII - ear	Devices and biomaterials
STG 1988	Policy planning	Coating of implant materials to improve acceptability	N/A	Other	Other - generic	Devices and biomaterials
STG 1988	Policy planning	Functional electrical stimulation in prosthetics allowing brain to control the action	N/A	Rehabilitation	XXI - screening, rehab	Devices and biomaterials
STG 1988	Policy planning	Prostheic fingers	N/A	Rehabilitation	XIII - musculoskeletal	Devices and biomaterials
STG 1988	Policy planning	Organ transplant and improvement of functional activities of organs (pancreas, bone, small bowel, endocine glands..)	N/A	Treatment and management of symptoms and disease	Other - generic	Other
STG 1988	Policy planning	Microprocessor to restore damaged areas of the brain	N/A	Rehabilitation	VI - nervous	Devices and biomaterials
STG 1988	Policy planning	Laser cytofluorometry will be used to identify RNA and DNA in the plasma	N/A	Diagnosis and monitoring of disease	Other - generic	Diagnostic tests and imaging
STG 1988	Policy planning	Laser doppler to measure blood flow in the retina	N/A	Diagnosis and monitoring of disease	Other - generic	Diagnostic tests and imaging
STG 1988	Policy planning	Laser to stop gastrointestinal bleeding	N/A	Treatment and management of symptoms and disease	XI - GI tract, dental health	Therapeutic procedures

For peer review only

Appendix1: List of identified healthcare technologies

STG 1988	Policy planning	Laser to break urinary stones	N/A	Treatment and management of symptoms and disease	XIV - genitourinary	Therapeutic procedures
STG 1988	Policy planning	Laser surgery through endoscope	N/A	Treatment and management of symptoms and disease	Other - generic	Therapeutic procedures
STG 1988	Policy planning	Widespread use of microsurgery	N/A	Treatment and management of symptoms and disease	Other - generic	Therapeutic procedures
STG 1988	Policy planning	Electronic gas valve regulator for safety in anaesthaesia	N/A	Supporting the provision of care and/or increasing service efficiency	XIX - injury	Medical equipment
STG 1988	Policy planning	Instantaneous measurement of blood gases and drug levels in the body during anasthaesia	N/A	Supporting patients receiving care (designed to be used by individuals)	XIX - injury	Diagnostic tests and imaging
STG 1988	Policy planning	Computerised ECG operating room	N/A	Supporting the provision of care and/or increasing service efficiency	Other - generic	IT systems, eHealth and mHealth
STG 1988	Policy planning	Data on each patient available at any moment during the operation	N/A	Supporting patients receiving care (designed to be used by individuals)	Other - generic	IT systems, eHealth and mHealth
STG 1988	Policy planning	Computerised electroanaesthesia	N/A	Supporting patients receiving care (designed to be used by individuals)	Other - generic	IT systems, eHealth and mHealth
STG 1988	Policy planning	New methods to close wounds e.g. tissue glue with ultrasound	N/A	Treatment and management of symptoms and disease	Other - generic	Devices and biomaterials
STG 1988	Policy planning	Spectroscopy using optical methods to diagnose caries	N/A	Diagnosis and monitoring of disease	XI - GI tract, dental health	Diagnostic tests and imaging
STG 1988	Policy planning	Bone like implants for teeth and bones	N/A	Treatment and management of symptoms and disease	XI - GI tract, dental health	Devices and biomaterials
STG 1988	Policy planning	Home monitoring especially of the elderly	N/A	Supporting patients receiving care (designed to	Other - generic	IT systems, eHealth and mHealth

For peer review only - http://bmjopen.bmj.com/site/about/guidelines.xhtml

Appendix1: List of identified healthcare technologies

				be used by individuals)		
STG 1988	Policy planning	Networks for communications between and within hospitals	N/A	Supporting the provision of care and/or increasing service efficiency	Other - generic	IT systems, eHealth and mHealth
STG 1988	Policy planning	Huge mass memories (e.g. on laser disc) for the storage of medical records	N/A	Supporting the provision of care and/or increasing service efficiency	Other - generic	IT systems, eHealth and mHealth
STG 1988	Policy planning	Video disk oriented data base for medical records	N/A	Supporting the provision of care and/or increasing service efficiency	Other - generic	IT systems, eHealth and mHealth
STG 1988	Policy planning	Computer aided examination and testing	N/A	Supporting patients receiving care (designed to be used by individuals)	Other - generic	IT systems, eHealth and mHealth
STG 1988	Policy planning	Improved biomaterials for joint implants and plastic surgery	N/A	Treatment and management of symptoms and disease	XIII - musculoskeletal	Devices and biomaterials
STG 1988	Policy planning	Improved materials for artificial blood vessels	N/A	Treatment and management of symptoms and disease	IX - circulation	Devices and biomaterials
Cahill et al Technology Map 1999	policy planning	Therapeutics for arterial occlusion and myocardial infarction	4	Treatment and management of symptoms and disease	IX - circulation	Drugs (not prophylactic vaccines)
Cahill et al Technology Map 1999	Policy planning	Guidelines for adult disease preventing life style	8	Supporting the provision of care and/or increasing service efficiency	XXI - screening, rehab	Organisational programmes
Cahill et al Technology Map 1999	policy planning	Development of anti-cancer agents (gene level)	11	Treatment and management of symptoms and disease	II - cancers	Drugs (not prophylactic vaccines)
Cahill et al Technology Map 1999	policy planning	drugs preventing the occurrence of cancer	12	Health promotion and prevention of disease	II - cancers	Drugs (not prophylactic vaccines)
Cahill et al Technology Map 1999	policy planning	Effective means to prevent metastasis	15	Health promotion and prevention of disease	II - cancers	Unknown
Cahill et al Technology Map 1999	policy planning	Genetic/biochemical tests for schizophrenia and depression	9	Diagnosis and monitoring of disease	VI - nervous	Diagnostic tests and imaging

For peer review only - http://bmjopen.bmj.com/site/about/guidelines.xhtml

Appendix1: List of identified healthcare technologies

Food and Drug Administration (FDA) surveys 1998	Policy planning	Biosensors	10	Unknown	Other - generic	Diagnostic tests and imaging
Food and Drug Administration (FDA) surveys 1998	Policy planning	Laser diagnosis	10	Diagnosis and monitoring of disease	Other - generic	Diagnostic tests and imaging
Food and Drug Administration (FDA) surveys 1998	Policy planning	Tissue engineered devices	10	Treatment and management of symptoms and disease	Other - generic	Devices and biomaterials
Food and Drug Administration (FDA) surveys 1998	Policy planning	Blood vessel prosthetics	10	Treatment and management of symptoms and disease	IX - circulation	Regenerative medicine
Food and Drug Administration (FDA) surveys 1998	Policy planning	Bone prosthetics/ growth	10	Treatment and management of symptoms and disease	XIII - musculoskeletal	Regenerative medicine
Food and Drug Administration (FDA) surveys 1998	Policy planning	Cardiac stimulation	10	Treatment and management of symptoms and disease	IX - circulation	Unknown
Food and Drug Administration (FDA) surveys 1998	Policy planning	cartilage prosthetics	10	Treatment and management of symptoms and disease	XIII - musculoskeletal	Devices and biomaterials
Food and Drug Administration (FDA) surveys 1998	Policy planning	Computer aided clinical labs	10	Diagnosis and monitoring of disease	Other - generic	IT systems, eHealth and mHealth
Food and Drug Administration (FDA) surveys 1998	Policy planning	Computer aided diagnosis	10	Supporting the provision of care and/or increasing service efficiency	Other - generic	IT systems, eHealth and mHealth
Food and Drug Administration (FDA) surveys 1998	Policy planning	Telemedicine	10	Supporting patients receiving care (designed to be used by individuals)	Other - generic	IT systems, eHealth and mHealth
Food and Drug Administration (FDA) surveys 1998	Policy planning	Drug impregnated devices	10	Treatment and management of symptoms and disease	Other - generic	Devices and biomaterials
Food and Drug Administration (FDA) surveys 1998	Policy planning	Endoscopy	10	Diagnosis and monitoring of disease	Other - generic	Diagnostic tests and imaging
Food and Drug Administration	Policy planning	Hearing aids/Nonimplante	10	Rehabilitation	VIII - ear	Devices and biomaterials

For peer review only - http://bmjopen.bmj.com/site/about/guidelines.xhtml

Appendix1: List of identified healthcare technologies

(FDA) surveys 1998		d sensory aids				
Food and Drug Administration (FDA) surveys 1998	Policy planning	Heart valves	10	Treatment and management of symptoms and disease	IX - circulation	Devices and biomaterials
Food and Drug Administration (FDA) surveys 1998	Policy planning	Heart pumps	10	Treatment and management of symptoms and disease	IX - circulation	Devices and biomaterials
Food and Drug Administration (FDA) surveys 1998	Policy planning	Home diagnostics /Home/self monitoring & diagnosis	10	Supporting patients receiving care (designed to be used by individuals)	Other - generic	Organisational programmes
Food and Drug Administration (FDA) surveys 1998	Policy planning	Image contrast agents	10	Diagnosis and monitoring of disease	Other - generic	Diagnostic tests and imaging
Food and Drug Administration (FDA) surveys 1998	Policy planning	Integrated patient medical info systems	10	Supporting the provision of care and/or increasing service efficiency	Other - generic	IT systems, eHealth and mHealth
Food and Drug Administration (FDA) surveys 1998	Policy planning	Laser treatment	10	Treatment and management of symptoms and disease	Other - generic	Non-surgical therapy
Food and Drug Administration (FDA) surveys 1998	Policy planning	kidney prosthetics	10	Treatment and management of symptoms and disease	XIV - genitourina ry	Regenerative medicine
Food and Drug Administration (FDA) surveys 1998	Policy planning	Laser surgery	10	Treatment and management of symptoms and disease	Other - generic	Non-surgical therapy
Food and Drug Administration (FDA) surveys 1998	Policy planning	Liver prosthetics	10	Treatment and management of symptoms and disease	XI - GI tract, dental health	Regenerative medicine
Food and Drug Administration (FDA) surveys 1998	Policy planning	Mini invasive neurosurgery	10	Treatment and management of symptoms and disease	VI - nervous	Therapeutic procedures
Food and Drug Administration (FDA) surveys 1998	Policy planning	Mini invasive cardiac surgery	10	Treatment and management of symptoms and disease	IX - circulation	Therapeutic procedures
Food and Drug Administration (FDA) surveys 1998	Policy planning	MRI	10	Diagnosis and monitoring of disease	Other - generic	Diagnostic tests and imaging

For peer review only - http://bmjopen.bmj.com/site/about/guidelines.xhtml

Appendix1: List of identified healthcare technologies

Food and Drug Administration (FDA) surveys 1998	Policy planning	Neuromuscular stimulation	10	Treatment and management of symptoms and disease	Other - generic	Non-surgical therapy
Food and Drug Administration (FDA) surveys 1998	Policy planning	Nerve regeneration	10	Treatment and management of symptoms and disease	VI - nervous	Regenerative medicine
Food and Drug Administration (FDA) surveys 1998	Policy planning	Neural stimulation	10	Treatment and management of symptoms and disease	VI - nervous	Non-surgical therapy
Food and Drug Administration (FDA) surveys 1998	Policy planning	Ocular prosthetics	10	Treatment and management of symptoms and disease	VII - eye	Devices and biomaterials
Food and Drug Administration (FDA) surveys 1998	Policy planning	Pancreas prosthetics	10	Treatment and management of symptoms and disease	IV - endocrine	Regenerative medicine
Food and Drug Administration (FDA) surveys 1998	Policy planning	Patient smart cards	10	Supporting patients receiving care (designed to be used by individuals)	Other - generic	IT systems, eHealth and mHealth
Food and Drug Administration (FDA) surveys 1998	Policy planning	Robotic surgery	10	Treatment and management of symptoms and disease	Other - generic	Therapeutic procedures
Food and Drug Administration (FDA) surveys 1998	Policy planning	Skin prosthetics	10	Treatment and management of symptoms and disease	XII - skin	Regenerative medicine
Food and Drug Administration (FDA) surveys 1998	Policy planning	Telemedicine-home use	10	Supporting patients receiving care (designed to be used by individuals)	Other - generic	IT systems, eHealth and mHealth
Food and Drug Administration (FDA) surveys 1998	Policy planning	Telemedicine-radiology	10	Supporting patients receiving care (designed to be used by individuals)	Other - generic	IT systems, eHealth and mHealth
Food and Drug Administration (FDA) surveys 2008	Policy planning	Robotic surgical systems	0	Treatment and management of symptoms and disease	Other - generic	Therapeutic procedures
Food and Drug Administration (FDA) surveys 1998	Policy planning	Point-of-care products	10	Diagnosis and monitoring of disease	Other - generic	Diagnostic tests and imaging

For peer review only - http://bmjopen.bmj.com/site/about/guidelines.xhtml

Appendix1: List of identified healthcare technologies

Food and Drug Administration (FDA) surveys 1998	Policy planning	Computer-assisted diagnostic systems	10	Diagnosis and monitoring of disease	Other - generic	IT systems, eHealth and mHealth
Food and Drug Administration (FDA) surveys 1998	Policy planning	Neuro-sensory devices	10	Diagnosis and monitoring of disease	VI - nervous	Devices and biomaterials
Food and Drug Administration (FDA) surveys 1998	Policy planning	Optical diagnostic products	10	Diagnosis and monitoring of disease	Other - generic	Diagnostic tests and imaging
Food and Drug Administration (FDA) surveys 1998	Policy planning	Integrated electronic patient medical records	10	Supporting the provision of care and/or increasing service efficiency	Other - generic	IT systems, eHealth and mHealth
Food and Drug Administration (FDA) surveys 1998	Policy planning	Remote patient monitoring systems	10	Supporting patients receiving care (designed to be used by individuals)	Other - generic	IT systems, eHealth and mHealth
Food and Drug Administration (FDA) surveys 1998	Policy planning	Glucose monitoring products	10	Diagnosis and monitoring of disease	IV - endocrine	Diagnostic tests and imaging
Food and Drug Administration (FDA) surveys 1998	Policy planning	Tissue engineered products	10	Treatment and management of symptoms and disease	Other - generic	Regenerative medicine
Food and Drug Administration (FDA) surveys 1998	Policy planning	Advanced prosthetic limbs	10	Rehabilitation	XIII - musculoskeletal	Devices and biomaterials
Food and Drug Administration (FDA) surveys 1998	Policy planning	Minimally invasive implants (e.g., percutaneous or natural orifice delivery)	10	Treatment and management of symptoms and disease	Other - generic	Devices and biomaterials
Food and Drug Administration (FDA) surveys 1998	Policy planning	Virtual reality systems for immersive training and other applications	10	Unknown	Other - generic	IT systems, eHealth and mHealth
Food and Drug Administration (FDA) surveys 1998	Policy planning	Optical therapeutic devices	10	Treatment and management of symptoms and disease	Other - generic	Devices and biomaterials
Food and Drug Administration (FDA) surveys 1998	Policy planning	Internet-based medical device systems	10	Supporting patients receiving care (designed to be used by	Other - generic	IT systems, eHealth and mHealth

For peer review only

Appendix1: List of identified healthcare technologies

				individuals)		
Food and Drug Administration (FDA) surveys 1998	Policy planning	New (non-eluting) drug delivery systems	10	Treatment and management of symptoms and disease	Other - generic	Drugs (not prophylactic vaccines)
Food and Drug Administration (FDA) surveys 1998	Policy planning	Robotic prosthetics	20	Rehabilitation	Other - generic	Devices and biomaterials
Food and Drug Administration (FDA) surveys 1998	Policy planning	New types of insulin pumps and delivery systems	20	Treatment and management of symptoms and disease	IV - endocrine	Devices and biomaterials
Food and Drug Administration (FDA) surveys 1998	Policy planning	New types of stents	20	Treatment and management of symptoms and disease	IX - circulation	Devices and biomaterials
Food and Drug Administration (FDA) surveys 1998	Policy planning	Joint replacements	20	Treatment and management of symptoms and disease	XIII - musculoske letal	Devices and biomaterials
Food and Drug Administration (FDA) surveys 1998	Policy planning	Image-guided therapy systems	20	Treatment and management of symptoms and disease	Other - generic	Therapeutic procedures
Food and Drug Administration (FDA) surveys 1998	Policy planning	Advanced ultrasound imaging systems	20	Diagnosis and monitoring of disease	Other - generic	Diagnostic tests and imaging
Food and Drug Administration (FDA) surveys 1998	Policy planning	Advanced optical imaging systems	20	Diagnosis and monitoring of disease	Other - generic	Diagnostic tests and imaging
Food and Drug Administration (FDA) surveys 1998	Policy planning	Advanced MR imaging systems	20	Diagnosis and monitoring of disease	Other - generic	Diagnostic tests and imaging
Food and Drug Administration (FDA) surveys 1998	Policy planning	Minimally invasive radiotherapy systems	20	Treatment and management of symptoms and disease	II - cancers	Non-surgical therapy
Food and Drug Administration (FDA) surveys 1998	Policy planning	Genetic diagnostic products	20	Diagnosis and monitoring of disease	Other - generic	Diagnostic tests and imaging
Food and Drug Administration (FDA) surveys 1998	Policy planning	Remote patient monitoring systems	20	Supporting patients receiving care (designed to be used by	Other - generic	IT systems, eHealth and mHealth

For peer review only - http://bmjopen.bmj.com/site/about/guidelines.xhtml

Appendix1: List of identified healthcare technologies

				individuals)		
Institute of the Future, 2009	Research	Bio-marker based tools will screen for future disease risk and produce new diagnoses based on probabilities	11	Diagnosis and monitoring of disease	Other - generic	Diagnostic tests and imaging
Institute of the Future, 2009	Research	Visualizations improve diet & exercise	11	Health promotion and prevention of disease	Other - generic	Non-surgical therapy
Institute of the Future, 2009	Research	GPS-enabled asthma inhalers	11	Supporting the provision of care and/or increasing service efficiency	X - respiratory	IT systems, eHealth and mHealth
Institute of the Future, 2009	Research	Workplace clinics	11	Health promotion and prevention of disease	Other - generic	Organisational programmes
Institute of the Future, 2009	Research	Urban planning for walkability, pollution reduction, neighbourood food systems	11	Health promotion and prevention of disease	Other - generic	Population programmes
Institute of the Future, 2009	Research	Report cards include Body Mass Index (BMI) scores	11	Health promotion and prevention of disease	Other - generic	Population programmes
Institute of the Future, 2009	Research	iPhone-synched medical devices	11	Supporting patients receiving care (designed to be used by individuals)	Other - generic	IT systems, eHealth and mHealth
Institute of the Future, 2009	Research	Quantifying, visualizing, and mapping health implications of environments	11	Health promotion and prevention of disease	Other - generic	Population programmes
Institute of the Future, 2009	Research	Deep brain stimulation used for neurological, behavioral issues	11	Treatment and management of symptoms and disease	VI - nervous	Devices and biomaterials
Institute of the Future, 2009	Research	Home sensors for elder care	11	Supporting patients receiving care (designed to be used by individuals)	Other - generic	IT systems, eHealth and mHealth
Institute of the Future, 2009	Research	Individually targeted therapies	11	Treatment and management of symptoms and	Other - generic	Drugs (not prophylactic vaccines)

For peer review only - http://bmjopen.bmj.com/site/about/guidelines.xhtml

Appendix1: List of identified healthcare technologies

				disease		
Institute of the Future, 2009	Research	Sensors track drug efficacy	11	Supporting patients receiving care (designed to be used by individuals)	Other - generic	Devices and biomaterials
Institute of the Future, 2009	Research	Genotypic diagnostics and risk	11	Diagnosis and monitoring of disease	Other - generic	Diagnostic tests and imaging
Institute of the Future, 2009	Research	New technologies enable individuals to experience the future effects of present-day behavior choices	11	Treatment and management of symptoms and disease	Other - generic	Unknown
Institute of the Future, 2009	Research	Regenerative medicine (general)will replace, restore, maintain, or enhance tissue and organ functions	11	Treatment and management of symptoms and disease	Other - generic	Regenerative medicine
Institute of the Future, 2009	Research	New ways of reliably measuring subjective states such as happiness, anxiety, or pain, and finding the neural correlates of personality and behavioral patterns	11	Diagnosis and monitoring of disease	VI - nervous	Diagnostic tests and imaging
Institute of the Future, 2009	Research	prototype of a prosthetic arm that can be controlled naturally and provide sensory feedback.	11	Rehabilitation	XIII - musculoskeletal	Devices and biomaterials
Stevens et al 1997	Research	Magnetic resonance imaging	1	Diagnosis and monitoring of disease	Other - generic	Diagnostic tests and imaging
Stevens et al 1997	Research	Minimally invasive surgery	1	Treatment and management of symptoms and disease	Other - generic	Therapeutic procedures
Stevens et al 1997	Research	drugs for Therapeutic refractory	1	Treatment and management of symptoms and	V - mental health	Drugs (not prophylactic vaccines)

For peer review only - http://bmjopen.bmj.com/site/about/guidelines.xhtml

Appendix1: List of identified healthcare technologies

		schizphrenia		disease		
Stevens et al 1997	Research	Implantable vascular stents	1	Treatment and management of symptoms and disease	IX - circulation	Devices and biomaterials
Stevens et al 1997	Research	Peripheral blood stem cells transplantation	1	Treatment and management of symptoms and disease	Other - generic	Regenerative medicine
Stevens et al 1997	Research	Laser Therapeutic of benign prostatic hyperplasia	1	Treatment and management of symptoms and disease	XIV - genitourina ry	Non-surgical therapy
Stevens et al 1997	Research	Gene therapy advances	4	Treatment and management of symptoms and disease	Other - generic	Regenerative medicine
Stevens et al 1997	Research	Polymerase chain reaction	4	Diagnosis and monitoring of disease	Other - generic	Diagnostic tests and imaging
Stevens et al 1997	Research	Telemedicine	4	Supporting the provision of care and/or increasing service efficiency	Other - generic	IT systems, eHealth and mHealth
Stevens et al 1997	Research	Interventional radiology	1	Treatment and management of symptoms and disease	Other - generic	Non-surgical therapy
Stevens et al 1997	Research	Angioplasty	1	Treatment and management of symptoms and disease	IX - circulation	Therapeutic procedures
Stevens et al 1997	Research	Interferon for chronic granulocytic leukemia	1	Treatment and management of symptoms and disease	II - cancers	Drugs (not prophylactic vaccines)
Stevens et al 1997	Research	Interferon for hepatitis C	1	Treatment and management of symptoms and disease	I - infections	Drugs (not prophylactic vaccines)
Stevens et al 1997	Research	Lasers for dermatology	1	Treatment and management of symptoms and disease	XII - skin	Devices and biomaterials
Stevens et al 1997	Research	Adjuvant chemotherapy in lung cancer	4	Treatment and management of symptoms and disease	II - cancers	Drugs (not prophylactic vaccines)

For peer review only - http://bmjopen.bmj.com/site/about/guidelines.xhtml

Appendix1: List of identified healthcare technologies

Stevens et al 1997	Research	Near patient testing	2	Diagnosis and monitoring of disease	Other - generic	Diagnostic tests and imaging
Stevens et al 1997	Research	Revision of joint replacement	1	Treatment and management of symptoms and disease	XIII - musculoskeletal	Therapeutic procedures
Stevens et al 1997	Research	Genetic screening	4	Diagnosis and monitoring of disease	Other - generic	Diagnostic tests and imaging
Stevens et al 1997	Research	Helicobactor pylori eradication	1	Treatment and management of symptoms and disease	I - infections	Drugs (not prophylactic vaccines)
Stevens et al 1997	Research	Positron emission tomography	4	Diagnosis and monitoring of disease	Other - generic	Diagnostic tests and imaging
Stevens et al 1997	Research	Phacoemulsification	1	Treatment and management of symptoms and disease	VII - eye	Therapeutic procedures
Stevens et al 1997	Research	Cochlear implants	1	Rehabilitation	VIII - ear	Devices and biomaterials
Stevens et al 1997	Research	Paclitaxels for ovarian & breast cancer	2	Treatment and management of symptoms and disease	II - cancers	Drugs (not prophylactic vaccines)
Stevens et al 1997	Research	Bone densitometry screening	1	Diagnosis and monitoring of disease	XIII - musculoskeletal	Diagnostic tests and imaging
Stevens et al 1997	Research	Anticoagulants for atrial fibrillation	1	Health promotion and prevention of disease	IX - circulation	Drugs (not prophylactic vaccines)
Stevens et al 1997	Research	Picture archiving and communication system	4	Supporting the provision of care and/or increasing service efficiency	Other - generic	IT systems, eHealth and mHealth
Stevens et al 1997	Research	rhDNase	2	Treatment and management of symptoms and disease	IV - endocrine	Drugs (not prophylactic vaccines)
Stevens et al 1997	Research	Biomaterials in bone and joint replacement	2	Treatment and management of symptoms and disease	XIII - musculoskeletal	Devices and biomaterials
Stevens et al 1997	Research	Increased use of adjuvant chemotherapy in colorectal cancer	2	Treatment and management of symptoms and disease	II - cancers	Drugs (not prophylactic vaccines)
Stevens et al 1997	Research	Increased use of high purity factor	2	Treatment and management of	III - blood	Drugs (not prophylactic

For peer review only - http://bmjopen.bmj.com/site/about/guidelines.xhtml

Appendix1: List of identified healthcare technologies

		VIII and recombinant factor VIII in hemophilia		symptoms and disease		vaccines)
Stevens et al 1997	Research	Mirena intrauterine contraceptive device	2	Health promotion and prevention of disease	XIV - genitourinary	Drugs (not prophylactic vaccines)
Stevens et al 1997	Research	Adjuvant chemotherapy in lung cancer	4	Treatment and management of symptoms and disease	II - cancers	Drugs (not prophylactic vaccines)
Stevens et al 1997	Research	drugs for Alzheimer's disease	6	Treatment and management of symptoms and disease	V - mental health	Drugs (not prophylactic vaccines)
Stevens et al 1997	Research	Xenotransplantation	6	Treatment and management of symptoms and disease	Other - generic	Regenerative medicine
Stevens et al 1997	Research	Screening for abdominal aortic aneurysm	6	Health promotion and prevention of disease	IX - circulation	Population programmes
Stevens et al 1997	Research	Remotely controlled robots (laboratory)	6	Supporting the provision of care and/or increasing service efficiency	Other - generic	Medical equipment
Stevens et al 1997	Research	Gene therapy for cystic fibrosis	6	Treatment and management of symptoms and disease	IV - endocrine	Regenerative medicine
Stevens et al 1997	Research	New anesthetic vapors	2	Treatment and management of symptoms and disease	Other - generic	Drugs (not prophylactic vaccines)
Stevens et al 1997	Research	Magnetic resonance angiography	4	Diagnosis and monitoring of disease	IX - circulation	Diagnostic tests and imaging
Stevens et al 1997	Research	Digital radiography	4	Supporting the provision of care and/or increasing service efficiency	Other - generic	IT systems, eHealth and mHealth
Stevens et al 1997	Research	Alendronate for osteoporosis	2	Treatment and management of symptoms and disease	XIII - musculoskeletal	Drugs (not prophylactic vaccines)
Stevens et al 1997	Research	Continuous positive airways pressure	1	Treatment and management of symptoms and disease	X - respiratory	Non-surgical therapy

For peer review only - http://bmjopen.bmj.com/site/about/guidelines.xhtml

Appendix1: List of identified healthcare technologies

Stevens et al 1997	Research	Expanding metal stents for esophageal cancer	1	Treatment and management of symptoms and disease	II - cancers	Devices and biomaterials
Stevens et al 1997	Research	Community placements for severe mental illness	1	Supporting patients receiving care (designed to be used by individuals)	V - mental health	Organisational programmes
Stevens et al 1997	Research	Fludarabine in lymphomas and chronic leukemias	2	Treatment and management of symptoms and disease	II - cancers	Drugs (not prophylactic vaccines)
Stevens et al 1997	Research	Combined therapy for HIV/AIDS	2	Treatment and management of symptoms and disease	I - infections	Drugs (not prophylactic vaccines)
Stevens et al 1997	Research	Epilepsy surgery	1	Treatment and management of symptoms and disease	VI - nervous	Therapeutic procedures
Stevens et al 1997	Research	Lipid-lowering drugs for elevated cholesterol	1	Health promotion and prevention of disease	IX - circulation	Drugs (not prophylactic vaccines)
Stevens et al 1997	Research	Repair of abdominal aortic aneurysm with transfemoral endovasculate (bifurcated) graft	4	Treatment and management of symptoms and disease	IX - circulation	Devices and biomaterials
Stevens et al 1997	Research	Intracytoplasmatic sperm injection	2	Treatment and management of symptoms and disease	XIV - genitourinary	Therapeutic procedures
Stevens et al 1997	Research	Computed tomography (CT) scan advances	2	Diagnosis and monitoring of disease	Other - generic	Diagnostic tests and imaging
Stevens et al 1997	Research	Voice activated dictation technology	1	Supporting the provision of care and/or increasing service efficiency	Other - generic	Organisational programmes
Stevens et al 1997	Research	Intra-arterial metallic stents	1	Treatment and management of symptoms and disease	IX - circulation	Devices and biomaterials
Tremblay and Yiu, 2006	Policy planning	Hand-held robotic and remote ultrasound	14	Diagnosis and monitoring of disease	Other - generic	Diagnostic tests and imaging
Tremblay and Yiu, 2006	Policy planning	MRI guided catheterization	14	Diagnosis and monitoring of disease	IX - circulation	Diagnostic tests and imaging

For peer review only - http://bmjopen.bmj.com/site/about/guidelines.xhtml

Appendix1: List of identified healthcare technologies

Tremblay and Yiu, 2006	Policy planning	Image-guided therapy and surgery and robotics	14	Treatment and management of symptoms and disease	Other - generic	Therapeutic procedures
Tremblay and Yiu, 2006	Policy planning	Computed Tomographic Colonography	14	Diagnosis and monitoring of disease	XI - GI tract, dental health	Diagnostic tests and imaging
Tremblay and Yiu, 2006	Policy planning	Digital Tomosynthesis	14	Diagnosis and monitoring of disease	Other - generic	Diagnostic tests and imaging
Tremblay and Yiu, 2006	Policy planning	Molecular-level imaging	14	Diagnosis and monitoring of disease	Other - generic	Diagnostic tests and imaging
Tremblay and Yiu, 2006	Policy planning	More economical scanners with improved image processing	14	Supporting the provision of care and/or increasing service efficiency	Other - generic	Medical equipment
Tremblay and Yiu, 2006	Policy planning	4D imaging	14	Diagnosis and monitoring of disease	Other - generic	Diagnostic tests and imaging
Tremblay and Yiu, 2006	Policy planning	Faster cycle time to capture an image and general reduction in scan times	14	Supporting the provision of care and/or increasing service efficiency	Other - generic	Medical equipment
Tremblay and Yiu, 2006	Policy planning	Hybrid PET/CT increasingly replacing invasive procedures	14	Diagnosis and monitoring of disease	Other - generic	Diagnostic tests and imaging
Tremblay and Yiu, 2006	Policy planning	Stereotactic radio-surgery for early detection of coronary heart diseases	14	Diagnosis and monitoring of disease	IX - circulation	Diagnostic tests and imaging
Tremblay and Yiu, 2006	Policy planning	Magnetic Resonance Guided Focused Ultrasound for Imaging of inflammation	14	Diagnosis and monitoring of disease	Other - generic	Diagnostic tests and imaging
Tremblay and Yiu, 2006	Policy planning	Wireless home-health monitoring	14	Supporting patients receiving care (designed to be used by individuals)	Other - generic	IT systems, eHealth and mHealth
Tremblay and Yiu, 2006	Policy planning	E-health diagnostic platform	14	Supporting the provision of care and/or increasing service efficiency	Other - generic	IT systems, eHealth and mHealth
Tremblay and Yiu, 2006	Policy planning	Hand-held gamma camera	14	Diagnosis and monitoring of	Other - generic	Diagnostic tests and imaging

For peer review only - http://bmjopen.bmj.com/site/about/guidelines.xhtml

Appendix1: List of identified healthcare technologies

				disease		
Tremblay and Yiu, 2006	Policy planning	"one-stop" diagnosis and Therapeutic	14	Supporting the provision of care and/or increasing service efficiency	Other - generic	Organisational programmes
Tremblay and Yiu, 2006	Policy planning	customised hybrid systems for specific clinical situations	14	Unknown	Other - generic	Organisational programmes
Tremblay and Yiu, 2006	Policy planning	Near Infra-red Spectroscopy	14	Diagnosis and monitoring of disease	Other - generic	Diagnostic tests and imaging
Tremblay and Yiu, 2006	Policy planning	Spatially-resolved Spectroscopy	14	Diagnosis and monitoring of disease	Other - generic	Diagnostic tests and imaging
Tremblay and Yiu, 2006	Policy planning	Optical Coherence Tomography	14	Diagnosis and monitoring of disease	Other - generic	Diagnostic tests and imaging
Tremblay and Yiu, 2006	Policy planning	Capsule Endoscopy	14	Diagnosis and monitoring of disease	Other - generic	Devices and biomaterials
Tremblay and Yiu, 2006	Policy planning	Tele-echography	14	Supporting the provision of care and/or increasing service efficiency	Other - generic	IT systems, eHealth and mHealth
Tremblay and Yiu, 2006	Policy planning	Tele-colposcopy	14	Supporting the provision of care and/or increasing service efficiency	Other - generic	IT systems, eHealth and mHealth
Tremblay and Yiu, 2006	Policy planning	Electronic Nose	14	Diagnosis and monitoring of disease	Other - generic	Diagnostic tests and imaging
Tremblay and Yiu, 2006	Policy planning	New contrast agents for Rubidium	14	Diagnosis and monitoring of disease	Other - generic	Diagnostic tests and imaging
Tremblay and Yiu, 2006	Policy planning	Hyperpolarised gases	14	Diagnosis and monitoring of disease	Other - generic	Diagnostic tests and imaging
Tremblay and Yiu, 2006	Policy planning	Terahertz waves	14	Diagnosis and monitoring of disease	Other - generic	Diagnostic tests and imaging
Tremblay and Yiu, 2006	Policy planning	Microwaves, light spectrum	14	Unknown	Other - generic	Devices and biomaterials
Tremblay and Yiu, 2006	Policy planning	Hyperpolarised imaging	14	Diagnosis and monitoring of disease	Other - generic	Diagnostic tests and imaging
Tremblay and Yiu, 2006	Policy planning	Specific advances in MRI, Ultrasound, Radiography…etc	14	Diagnosis and monitoring of disease	Other - generic	Diagnostic tests and imaging

For peer review only - http://bmjopen.bmj.com/site/about/guidelines.xhtml

Appendix1: List of identified healthcare technologies

Tremblay and Yiu, 2006	Policy planning	Reduction in use of invasive diagnostic procedures	14	Supporting the provision of care and/or increasing service efficiency	Other - generic	Organisational programmes
Tremblay and Yiu, 2006	Policy planning	Diagnosis of kidney disorders without renal biopsy	14	Diagnosis and monitoring of disease	XIV - genitourinary	Unknown
Tremblay and Yiu, 2006	Policy planning	Ex vivo molecular-computer diagnosis	14	Diagnosis and monitoring of disease	Other - generic	IT systems, eHealth and mHealth
Tremblay and Yiu, 2006	Policy planning	Smart-drug delivery systems	14	Treatment and management of symptoms and disease	Other - generic	Devices and biomaterials
Tremblay and Yiu, 2006	Policy planning	Polymer-encapsulated, bioconjugated quantum dots	9	Diagnosis and monitoring of disease	Other - generic	Diagnostic tests and imaging
Tremblay and Yiu, 2006	Policy planning	Patient accessed universal health records	4	Supporting the provision of care and/or increasing service efficiency	Other - generic	Organisational programmes
Tremblay and Yiu, 2006	Policy planning	In silico-in vivo integration (lab on-a-chip)	14	Diagnosis and monitoring of disease	Other - generic	Diagnostic tests and imaging
Tremblay and Yiu, 2006	Policy planning	Wide deployment of non-invasive technologies and biosensors	9	Supporting the provision of care and/or increasing service efficiency	Other - generic	Devices and biomaterials
Tremblay and Yiu, 2006	Policy planning	Direct consumer access to diagnostic tests	6	Supporting patients receiving care (designed to be used by individuals)	Other - generic	Organisational programmes
Tremblay and Yiu, 2006	Policy planning	Molecular imaging with single molecular pecision	9	Diagnosis and monitoring of disease	Other - generic	Diagnostic tests and imaging
Tremblay and Yiu, 2006	Policy planning	diagnostic testing throughout the care pathway	7	Supporting the provision of care and/or increasing service efficiency	Other - generic	Diagnostic tests and imaging
Tremblay and Yiu, 2006	Policy planning	Neuro-imaging at cellular level of brain processes (thoughts)	14	Diagnosis and monitoring of disease	VI - nervous	Diagnostic tests and imaging
Tremblay and Yiu, 2006	Policy planning	Molecular-computers used to design smart therapies	14	Other	Other - generic	Drugs (not prophylactic vaccines)

For peer review only - http://bmjopen.bmj.com/site/about/guidelines.xhtml

Appendix1: List of identified healthcare technologies

Tremblay and Yiu, 2006	Policy planning	Remote internet diagnosis based on data received from patient's home	9	Supporting patients receiving care (designed to be used by individuals)	Other - generic	IT systems, eHealth and mHealth
Tremblay and Yiu, 2006	Policy planning	Molecular diagnosis	14	Diagnosis and monitoring of disease	Other - generic	Diagnostic tests and imaging
Tremblay and Yiu, 2006	Policy planning	diagnostic -guided therapy for individualised medicine	14	Treatment and management of symptoms and disease	Other - generic	Drugs (not prophylactic vaccines)
Tremblay and Yiu, 2006	Policy planning	Computer-assisted diagnosis with pattern recognition	14	Diagnosis and monitoring of disease	Other - generic	IT systems, eHealth and mHealth
Tremblay and Yiu, 2006	Policy planning	Self-propelled micro-machines for diagnosis and Therapeutic	9	Unknown	Other - generic	Devices and biomaterials
Tremblay and Yiu, 2006	Policy planning	diagnostic bio-chip for cancer	9	Diagnosis and monitoring of disease	II - cancers	Diagnostic tests and imaging
Tremblay and Yiu, 2006	Policy planning	Investigation of thrombosis and vascular pathology using 3D tomography with remote controlled micro-instruments	14	Diagnosis and monitoring of disease	IX - circulation	Devices and biomaterials
Tremblay and Yiu, 2006	Policy planning	Personal care nurse-robots	12	Supporting patients receiving care (designed to be used by individuals)	Other - generic	Medical equipment
Tremblay and Yiu, 2006	Policy planning	Functional combination of targeting and imaging	14	Diagnosis and monitoring of disease	Other - generic	Diagnostic tests and imaging
Tremblay and Yiu, 2006	Policy planning	Intelligent and context sensitive clinical systems	14	Supporting the provision of care and/or increasing service efficiency	Other - generic	Organisational programmes
Tremblay and Yiu, 2006	Policy planning	Personal access to genomic information	14	Supporting patients receiving care (designed to be used by individuals)	Other - generic	Unknown
Tremblay and Yiu, 2006	Policy planning	Development of effective gene	9	Treatment and management of	Other - generic	Regenerative medicine

For peer review only - http://bmjopen.bmj.com/site/about/guidelines.xhtml

Appendix1: List of identified healthcare technologies

		therapies		symptoms and disease		
Science & Technology Foresight survey Japan 1997	Policy planning	Widespread use of scientific guidelines for adult-disease-preventing lifestyles (nutrition, rest and exercise)	9	Health promotion and prevention of disease	Other - generic	Organisational programmes
Science & Technology Foresight survey Japan 1997	Policy planning	Practical use of chemotherapy that brings complete remission to digestive organ cancer with low drug-responsiveness	18	Treatment and management of symptoms and disease	II - cancers	Drugs (not prophylactic vaccines)
Science & Technology Foresight survey Japan 1997	Policy planning	Widespread use of techniques to overcome the drug resistance of malignant tumours	16	Treatment and management of symptoms and disease	II - cancers	Drugs (not prophylactic vaccines)
Science & Technology Foresight survey Japan 1997	Policy planning	Widespread use of drugs that cure viral liver disease	13	Treatment and management of symptoms and disease	XI - GI tract, dental health	Drugs (not prophylactic vaccines)
Science & Technology Foresight survey Japan 1997	Policy planning	Widespread use of biological and immunological therapy effective for cancer	14	Treatment and management of symptoms and disease	II - cancers	Drugs (not prophylactic vaccines)
Science & Technology Foresight survey Japan 1997	Policy planning	Development of effective methods of preventing Alzheimer's disease	16	Health promotion and prevention of disease	V - mental health	Unknown
Science & Technology Foresight survey Japan 1997	Policy planning	Possible to cure allergic diseases	21	Treatment and management of symptoms and disease	III - blood	Unknown
Science & Technology Foresight survey Japan 1997	Policy planning	Development of an HIV vaccine	10	Health promotion and prevention of disease	I - infections	Prophylactic vaccines
Science & Technology Foresight survey Japan 1997	Policy planning	Widespread use of gene therapy against malignant tumors	17	Treatment and management of symptoms and disease	II - cancers	Regenerative medicine

For peer review only

Appendix1: List of identified healthcare technologies

Science & Technology Foresight survey Japan 1997	Policy planning	Widespread use of prevention methods for the contraction of diabetic complications	11	Health promotion and prevention of disease	IV - endocrine	Unknown
Science & Technology Foresight survey Japan 1997	Policy planning	Practical use of anti-AIDS therapy	12	Treatment and management of symptoms and disease	I - infections	Drugs (not prophylactic vaccines)
Science & Technology Foresight survey Japan 1997	Policy planning	Widespread use of an early cancer diagnosis technique based on biochemical examination	10	Diagnosis and monitoring of disease	II - cancers	Diagnostic tests and imaging
Science & Technology Foresight survey Japan 1997	Policy planning	Widespread use of a technique to eliminate viruses from blood	12	Treatment and management of symptoms and disease	I - infections	Therapeutic procedures
Science & Technology Foresight survey Japan 1997	Policy planning	Practical use of prevention methods for stress-induced mental disorders	14	Health promotion and prevention of disease	V - mental health	Unknown
Science & Technology Foresight survey Japan 1997	Policy planning	Practical use of a method to quantitatively assess the level of aging (biological age) in relation to chronological age.	9	Diagnosis and monitoring of disease	Other - generic	Unknown
Science & Technology Foresight survey Japan 1997	Policy planning	Practical use of a preventive measure against diabetic complications.	14	Health promotion and prevention of disease	IV - endocrine	Unknown
Science & Technology Foresight survey Japan 1997	Policy planning	Widespread use of presbyopia prevention methods	29	Health promotion and prevention of disease	VII - eye	Unknown
Science & Technology Foresight survey Japan 1997	Policy planning	Widespread use of a laser-applied tooth decay treatment method	14	Treatment and management of symptoms and disease	XI - GI tract, dental health	Devices and biomaterials
Science & Technology Foresight survey Japan 1997	Policy planning	Widespread method of prevention against a premature delivery through	8	Health promotion and prevention of disease	XV - pregnancy	Organisational programmes

For peer review only - http://bmjopen.bmj.com/site/about/guidelines.xhtml

Appendix1: List of identified healthcare technologies

		the progression of management system of fetal information.				
Science & Technology Foresight survey Japan 1997	Policy planning	Practical use of the prevention against congenital anomaly originating in the embryonic or fetal period.	19	Health promotion and prevention of disease	XV - pregnancy	Unknown
Science & Technology Foresight survey Japan 1997	Policy planning	Widespread use of preventive measure for cerebral palsy	19	Health promotion and prevention of disease	VI - nervous	Unknown
Science & Technology Foresight survey Japan 1997	Policy planning	Widespread use of a cancer spread diagnosis technique based on 3-D imaging.	7	Diagnosis and monitoring of disease	II - cancers	Diagnostic tests and imaging
Science & Technology Foresight survey Japan 1997	Policy planning	Widespread use of a cancer risk assessment technique based on genetic analysis.	11	Diagnosis and monitoring of disease	II - cancers	Unknown
Science & Technology Foresight survey Japan 1997	Policy planning	Widespread use of against of cancer cells drug sensitivity measurement method.	9	Unknown	II - cancers	Unknown
Science & Technology Foresight survey Japan 1997	Policy planning	Widespread use of automatic testing equipment for cancer cytodiagnosis.	9	Diagnosis and monitoring of disease	II - cancers	Diagnostic tests and imaging
Science & Technology Foresight survey Japan 1997	Policy planning	Practical use of classification and stage determination of schizophrenia based on diagnostic imaging.	24	Diagnosis and monitoring of disease	V - mental health	Diagnostic tests and imaging
Science & Technology Foresight survey Japan 1997	Policy planning	Practical use of diagnosing methods for determining the level and spread of arteriosclerosis	9	Diagnosis and monitoring of disease	IX - circulation	Diagnostic tests and imaging

For peer review only - http://bmjopen.bmj.com/site/about/guidelines.xhtml

Appendix1: List of identified healthcare technologies

		focused by a non-invasive process				
Science & Technology Foresight survey Japan 1997	Policy planning	Establishment of a correct diagnostic measure for kidney diseases without renal biopsy.	19	Diagnosis and monitoring of disease	XIV - genitourinary	Diagnostic tests and imaging
Science & Technology Foresight survey Japan 1997	Policy planning	Development of a capsule-type total gastro-intestinal tract monitoring system.	6	Diagnosis and monitoring of disease	XI - GI tract, dental health	Devices and biomaterials
Science & Technology Foresight survey Japan 1997	Policy planning	Practical use of specialist-level medical diagnosis assistance systems.	9	Supporting the provision of care and/or increasing service efficiency	Other - generic	IT systems, eHealth and mHealth
Science & Technology Foresight survey Japan 1997	Policy planning	Development of non-invasive cell-level diagnostic imaging.	8	Diagnosis and monitoring of disease	Other - generic	Diagnostic tests and imaging
Science & Technology Foresight survey Japan 1997	Policy planning	Practical use of a non-invasive technique to obtain blood biochemical examination data using body surface sensors.	9	Diagnosis and monitoring of disease	III - blood	Devices and biomaterials
Science & Technology Foresight survey Japan 1997	Policy planning	Practical use of a rapid diagnostic method for rejection in organ and tissue transplantation.	6	Diagnosis and monitoring of disease	III - blood	Unknown
Science & Technology Foresight survey Japan 1997	Policy planning	Widespread use of malaria vaccines.	10	Health promotion and prevention of disease	I - infections	Prophylactic vaccines
Science & Technology Foresight survey Japan 1997	Policy planning	Development of an effective insulin that can be administered orally.	16	Treatment and management of symptoms and disease	IV - endocrine	Drugs (not prophylactic vaccines)
Science & Technology Foresight survey Japan 1997	Policy planning	Practical use of gene therapy for diabetes.	16	Treatment and management of symptoms and disease	IV - endocrine	Regenerative medicine

For peer review only - http://bmjopen.bmj.com/site/about/guidelines.xhtml

For peer review only

Appendix1: List of identified healthcare technologies

Science & Technology Foresight survey Japan 1997	Policy planning	Widespread use of gene therapy for familial hypercholesterolemia.	14	Treatment and management of symptoms and disease	IX - circulation	Regenerative medicine
Science & Technology Foresight survey Japan 1997	Policy planning	Development of radiosensitizers effective in cancer treatment.	8	Treatment and management of symptoms and disease	II - cancers	Therapeutic procedures
Science & Technology Foresight survey Japan 1997	Policy planning	Widespread use of heavy particle against cancer	14	Treatment and management of symptoms and disease	II - cancers	Non-surgical therapy
Science & Technology Foresight survey Japan 1997	Policy planning	Development of a technique to cause drug delivery to targeted sites in the brain.	19	Treatment and management of symptoms and disease	Other - generic	Drugs (not prophylactic vaccines)
Science & Technology Foresight survey Japan 1997	Policy planning	Practical use of a safe pain control method without side effects	12	Treatment and management of symptoms and disease	Other - generic	Unknown
Science & Technology Foresight survey Japan 1997	Policy planning	Widespread use of gene therapy for muscular dystrophy.	24	Treatment and management of symptoms and disease	VI - nervous	Regenerative medicine
Science & Technology Foresight survey Japan 1997	Policy planning	Development of effective treatment for amyotrophic lateral sclerosis.	31	Treatment and management of symptoms and disease	VI - nervous	Unknown
Science & Technology Foresight survey Japan 1997	Policy planning	Practical use of artificial muscles for limbs.	21	Rehabilitation	XXI - screening, rehab	Devices and biomaterials
Science & Technology Foresight survey Japan 1997	Policy planning	Practical use of a technique to induce the differentiation of fibroblasts into cardiac muscle and its application to the treatment of myocardial infarction.	20	Treatment and management of symptoms and disease	IX - circulation	Regenerative medicine
Science & Technology Foresight survey Japan 1997	Policy planning	Development of hybrid-type artificial endocrine organs in which living	11	Treatment and management of symptoms and disease	IV - endocrine	Regenerative medicine

For peer review only - http://bmjopen.bmj.com/site/about/guidelines.xhtml

Appendix1: List of identified healthcare technologies

		cells and artificial objects coexist.				
Science & Technology Foresight survey Japan 1997	Policy planning	Widespread use of functional blood purifiers that selectively remove specified components from blood	19	Treatment and management of symptoms and disease	III - blood	Devices and biomaterials
Science & Technology Foresight survey Japan 1997	Policy planning	Widespread use of artificial hemoglobin as a red blood cell substitute.	15	Treatment and management of symptoms and disease	III - blood	Regenerative medicine
Science & Technology Foresight survey Japan 1997	Policy planning	Development of fully implanted artificial heart	14	Treatment and management of symptoms and disease	IX - circulation	Devices and biomaterials
Science & Technology Foresight survey Japan 1997	Policy planning	Practical use of fully implanted artificial lungs.	29	Treatment and management of symptoms and disease	X - respiratory	Devices and biomaterials
Science & Technology Foresight survey Japan 1997	Policy planning	Practical use of fully implanted artificial kidneys	26	Treatment and management of symptoms and disease	XIV - genitourina ry	Devices and biomaterials
Science & Technology Foresight survey Japan 1997	Policy planning	Practical use of fully implanted artificial pancreas	12	Treatment and management of symptoms and disease	XI - GI tract, dental health	Devices and biomaterials
Science & Technology Foresight survey Japan 1997	Policy planning	Widespread use of prevention and treatment methods for tooth decay and periodontitis to ensure the retention of 20 or more teeth at 80 year of age	14	Health promotion and prevention of disease	XI - GI tract, dental health	Population programmes
Science & Technology Foresight survey Japan 1997	Policy planning	Widespread use of a technique to store, cultivate and transplant one's own tissues.	15	Treatment and management of symptoms and disease	Other - generic	Regenerative medicine
Science & Technology Foresight survey Japan 1997	Policy planning	Practical use of implanted bladder control devices	12	Treatment and management of symptoms and disease	XIV - genitourina ry	Devices and biomaterials

For peer review only - http://bmjopen.bmj.com/site/about/guidelines.xhtml

Appendix1: List of identified healthcare technologies

Science & Technology Foresight survey Japan 1997	Policy planning	Practical use of gene therapy for genetic disorders	19	Treatment and management of symptoms and disease	Other - generic	Regenerative medicine
Science & Technology Foresight survey Japan 1997	Policy planning	Availability of genetherapy based on oral delivery	24	Treatment and management of symptoms and disease	Other - generic	Regenerative medicine
Science & Technology Foresight survey Japan 1997	Policy planning	Practical use of heterogeneous organ transplantation as means of treatment.	21	Treatment and management of symptoms and disease	Other - generic	Therapeutic procedures
Science & Technology Foresight survey Japan 1997	Policy planning	Widespread use of a worldwide organ supply system	12	Supporting the provision of care and/or increasing service efficiency	Other - generic	Organisational programmes
Science & Technology Foresight survey Japan 1997	Policy planning	Development of a partial tissue destruction method non-invasive to the living body.	6	Treatment and management of symptoms and disease	Other - generic	Non-surgical therapy
Science & Technology Foresight survey Japan 1997	Policy planning	Practical use of implanted artificial cochleas effective for both conductive hearing loss and perceptive hearing loss.	14	Rehabilitation	VIII - ear	Devices and biomaterials
Science & Technology Foresight survey Japan 1997	Policy planning	Development of a device to sense the needs of demented aged people.	21	Supporting patients receiving care (designed to be used by individuals)	V - mental health	Devices and biomaterials
Science & Technology Foresight survey Japan 1997	Policy planning	Development of controlling devices which help the cooperative muscular actions enabling object-oriented movement.	14	Rehabilitation	XXI - screening, rehab	Devices and biomaterials
Science & Technology Foresight survey Japan 1997	Policy planning	Widespread use of artificial legs featuring a small power source and computer control.	11	Rehabilitation	XXI - screening, rehab	Devices and biomaterials

For peer review only - http://bmjopen.bmj.com/site/about/guidelines.xhtml

Appendix1: List of identified healthcare technologies

Science & Technology Foresight survey Japan 1997	Policy planning	Practical use of a blood cell production and supply technique based on industrial-scale cultivation.	15	Treatment and management of symptoms and disease	III - blood	Regenerative medicine
Science & Technology Foresight survey Japan 1997	Policy planning	Practical use of systems for monitoring health condition and providing information for an appropriate diagnosis at home in event of accident or diseases.	11	Supporting patients receiving care (designed to be used by individuals)	XIX - injury	IT systems, eHealth and mHealth
Science & Technology Foresight survey Japan 1997	Policy planning	Widespread use of portable conversational speech interpretation systems.	24	Unknown	Other - generic	IT systems, eHealth and mHealth
Science & Technology Foresight survey Japan 1997	Policy planning	Widespread use of robots that care for people with severe physical and mental disabilities.	12	Supporting patients receiving care (designed to be used by individuals)	Other - generic	Medical equipment
Science & Technology Foresight survey Japan 1997	Policy planning	Advancements in patient and hospital information management, leading to a reduction in waiting time at Japanese hospitals to 15 minutes or less.	7	Supporting the provision of care and/or increasing service efficiency	Other - generic	Organisational programmes
Science & Technology Foresight survey Japan 1997	Policy planning	Practical use in Japan of an ID card system that thoroughly covers an individual's health conditions and medical data.	9	Supporting the provision of care and/or increasing service efficiency	Other - generic	Organisational programmes
Science & Technology Foresight survey	Policy planning	Elucidation of the biological clock, leading to drug	9	Supporting patients receiving care (designed to	Other - generic	Unknown

For peer review only - http://bmjopen.bmj.com/site/about/guidelines.xhtml

Appendix1: List of identified healthcare technologies

Japan 1997		administration that takes it into consideration		be used by individuals)		
Science & Technology Foresight survey Japan 1997	Policy planning	Practical application of the production of artificial cells with organ characteristics.	29	Treatment and management of symptoms and disease	Other - generic	Regenerative medicine
Science & Technology Foresight survey Japan 1997	Policy planning	Practical use of anti-AIDS therapy	12	Treatment and management of symptoms and disease	I - infections	Drugs (not prophylactic vaccines)
Science & Technology Foresight survey Japan 1997	Policy planning	Practical use of a rapid diagnostic method for rejection in organ and tissue transplantation	10	Diagnosis and monitoring of disease	Other - generic	Diagnostic tests and imaging
Science & Technology Foresight survey Japan 1997	Policy planning	Development of anti-cancer agents which target the manifestation functions of cancer genes	10	Treatment and management of symptoms and disease	II - cancers	Drugs (not prophylactic vaccines)
Science & Technology Foresight survey Japan 1997	Policy planning	Utilization of information about the gene structure of each individual patient in diagnosis and treatment	16	Diagnosis and monitoring of disease	Other - generic	Organisational programmes
Science & Technology Foresight survey Japan 1997	Policy planning	Development of technology to regenerate organs or individuals from separated animal cells	20	Treatment and management of symptoms and disease	Other - generic	Regenerative medicine
Science & Technology Foresight survey Japan 1997	Policy planning	Practical use of methods for directly determining DNA sequences by physical means such as X-rays.	14	Diagnosis and monitoring of disease	Other - generic	Diagnostic tests and imaging
Science & Technology Foresight survey Japan 1997	Policy planning	Control of signal transduction in the carcinogenesis of cells, and widespread use of treatment	20	Treatment and management of symptoms and disease	II - cancers	Drugs (not prophylactic vaccines)

For peer review only - http://bmjopen.bmj.com/site/about/guidelines.xhtml

Appendix1: List of identified healthcare technologies

		methods for dysdifferentiating carcinogenic cells.				
Science & Technology Foresight survey Japan 1997	Policy planning	Complete understanding of the factors contributing to stem cell multiplication, and widespread use of the practice of multiplying stem cells, as necessary, in test tubes and using them for treatment purpose	15	Treatment and management of symptoms and disease	Other - generic	Regenerative medicine
Science & Technology Foresight survey Japan 1997	Policy planning	Development of technology capable of synthesizing living cells by using only artificially produced chemical compounds.	31	Treatment and management of symptoms and disease	Other - generic	Regenerative medicine
Science & Technology Foresight survey Japan 1997	Policy planning	Practical use of elementary particle beam technology in genetic engineering.	19	Treatment and management of symptoms and disease	Other - generic	Regenerative medicine
Science & Technology Foresight survey Japan 1997	Policy planning	Development of drugs capable of preventing the occurrence of certain types of cancer.	11	Health promotion and prevention of disease	II - cancers	Drugs (not prophylactic vaccines)
Science & Technology Foresight survey Japan 1997	Policy planning	Practical use of effective means to prevent metastasis of cancer.	14	Treatment and management of symptoms and disease	II - cancers	Unknown
Science & Technology Foresight survey Japan 1997	Policy planning	Widespread use of technologies for long-term (semi-permanent) culturing and preservation of	19	Treatment and management of symptoms and disease	Other - generic	Regenerative medicine

For peer review only - http://bmjopen.bmj.com/site/about/guidelines.xhtml

Appendix1: List of identified healthcare technologies

		organs.				
Science & Technology Foresight survey Japan 1997	Policy planning	Practical use of artificial organs (pancreases, kidneys, livers, etc.) incorporating human cells and tissues	14	Treatment and management of symptoms and disease	Other - generic	Regenerative medicine
Science & Technology Foresight survey Japan 1997	Policy planning	Development of an entirely implantable artificial kidney.	18	Treatment and management of symptoms and disease	XIV - genitourinary	Regenerative medicine
Science & Technology Foresight survey Japan 1997	Policy planning	Development of an artificial cornea, allowing the visually impaired to regain their sight.	25	Treatment and management of symptoms and disease	VII - eye	Devices and biomaterials
Science & Technology Foresight survey Japan 1997	Policy planning	Development of artificial muscle elements.	21	Treatment and management of symptoms and disease	Other - generic	Regenerative medicine
Science & Technology Foresight survey Japan 1997	Policy planning	Establishment of interface technology between neural information and artificial organism structures.	25	Treatment and management of symptoms and disease	VI - nervous	Medical equipment
Science & Technology Foresight survey Japan 1997	Policy planning	Development of neural-computers with new logic structures modeled on brain functions.	24	Unknown	VI - nervous	IT systems, eHealth and mHealth
Science & Technology Foresight survey Japan 1997	Policy planning	Development of interfaces enabling direct linkage between the computer and the brain.	21	Treatment and management of symptoms and disease	XXI - screening, rehab	Devices and biomaterials
Science & Technology Foresight survey Japan 1997	Policy planning	Development of electric circuits with similar self-organization and self-restoration functions as neural networks.	14	Treatment and management of symptoms and disease	VI - nervous	Devices and biomaterials

For peer review only - http://bmjopen.bmj.com/site/about/guidelines.xhtml

Appendix1: List of identified healthcare technologies

Science & Technology Foresight survey Japan 1997	Policy planning	Development of diagnostic and medical treatment micromachines capable of traveling on their own inside organisms (body cavity organs).	24	Diagnosis and monitoring of disease	Other - generic	Devices and biomaterials
Science & Technology Foresight survey Japan 1997	Policy planning	Widespread use of remote surgery systems utilizing virtual reality technology.	14	Supporting the provision of care and/or increasing service efficiency	Other - generic	IT systems, eHealth and mHealth
Science & Technology Foresight survey Japan 1997	Policy planning	Widespread use of vicarious experience devices (using virtual reality technology) which allow bed-ridden patients to take vacations, etc.	9	Rehabilitation	XXI - screening, rehab	IT systems, eHealth and mHealth
Science & Technology Foresight survey Japan 1997	Policy planning	Widespread use of methods for controlling the immune system locally	14	Treatment and management of symptoms and disease	III - blood	Unknown
Science & Technology Foresight survey Japan 1997	Policy planning	Development of effective Therapeutic for amyotrophic lateral sclerosis	21	Treatment and management of symptoms and disease	VI - nervous	Drugs (not prophylactic vaccines)
Science & Technology Foresight survey Japan 1997	Policy planning	Practical use of batteries of artificial organs implanted in the living body	17	Treatment and management of symptoms and disease	Other - generic	Devices and biomaterials
Science & Technology Foresight survey Japan 1997	Policy planning	Development of artificial liver (external devices supporting liver functions) usable on a long-term, continuous basis	19	Treatment and management of symptoms and disease	XI - GI tract, dental health	Devices and biomaterials
Science & Technology Foresight survey Japan 1997	Policy planning	Practical use of heterogeneous organ transplantation as means of Therapeutic	19	Treatment and management of symptoms and disease	Other - generic	Therapeutic procedures

For peer review only - http://bmjopen.bmj.com/site/about/guidelines.xhtml

For peer review only

Appendix1: List of identified healthcare technologies

Science & Technology Foresight survey Japan 1997	Policy planning	Development of artificial eyes featuring electronic circuitry that can be connected to nerve and brain cells	24	Rehabilitation	VII - eye	Devices and biomaterials
Science & Technology Foresight survey Japan 1997	Policy planning	Development of methods for recombining disconnected central nerves	29	Rehabilitation	VI - nervous	Therapeutic procedures
Loveridge et al 1998	Policy planning	Practical use of directly visualising molecular structure at an atomic level (e.g. ultramicroscopy)	21	Diagnosis and monitoring of disease	Other - generic	Diagnostic tests and imaging
Loveridge et al 1998	Policy planning	At least 3 common diseases will be treated by portable or implanted systems that combine individually tailored drug delivery with automatic feedback control from a devices continuously monitoring the condition (e.g. diabetes control)	21	Treatment and management of symptoms and disease	Other - generic	Devices and biomaterials
Loveridge et al 1998	Policy planning	Over-the-counter nucleicacid-based tests suitable for self-administration become available for the first time	21	Supporting patients receiving care (designed to be used by individuals)	Other - generic	IT systems, eHealth and mHealth
Loveridge et al 1998	Policy planning	Results of more than 50% of all types of diagnostic tests are available at the point of need within one hour	21	Diagnosis and monitoring of disease	Other - generic	Diagnostic tests and imaging

For peer review only - http://bmjopen.bmj.com/site/about/guidelines.xhtml

Appendix1: List of identified healthcare technologies

Loveridge et al 1998	Policy planning	Widespread use of real time 3-D high resolutiom imaging of physiological and metabolic functions	21	Diagnosis and monitoring of disease	Other - generic	Diagnostic tests and imaging
Loveridge et al 1998	Policy planning	First practical use of non-invasive spectroscopy for in situ chemical pathology	21	Diagnosis and monitoring of disease	Other - generic	Diagnostic tests and imaging
Loveridge et al 1998	Policy planning	Practical use of biosensors in a wide range of medical manufacturing and environmental applications	21	Unknown	Other - generic	Devices and biomaterials
Loveridge et al 1998	Policy planning	All common single genes diseases can be treated successfully by gene theraby	21	Treatment and management of symptoms and disease	Other - generic	Drugs (not prophylactic vaccines)
Loveridge et al 1998	Policy planning	Practical use of gene therapy is extended to the treatment of 30% of major life threatening diseases	21	Treatment and management of symptoms and disease	Other - generic	Drugs (not prophylactic vaccines)
Loveridge et al 1998	Policy planning	Nucleic acid based diagnostic techniques account for 10% of all diagnostic e work load	21	Diagnosis and monitoring of disease	Other - generic	Organisational programmes
Loveridge et al 1998	Policy planning	First practical use of therapies based on purpose·designed non-peptide molecules which mimic the activity of peptides.	21	Treatment and management of symptoms and disease	Other - generic	Drugs (not prophylactic vaccines)
Loveridge et al 1998	Policy planning	Widespread use of polypeptide drugs which are delivered orally and targeted to	21	Treatment and management of symptoms and disease	Other - generic	Drugs (not prophylactic vaccines)

For peer review only - http://bmjopen.bmj.com/site/about/guidelines.xhtml

Appendix1: List of identified healthcare technologies

		specific tissues				
Loveridge et al 1998	Policy planning	Widespread use of cosmaceuticals with scientifically proven effectiveness to treat the sypmtoms of skin ageing (e.g. wrinkles)	21	Health promotion and prevention of disease	XII - skin	Drugs (not prophylactic vaccines)
Loveridge et al 1998	Policy planning	Widespread use of improved technologies for early diagnosis increases the 5-year survival rate of cancer patients by 50%	21	Diagnosis and monitoring of disease	II - cancers	Diagnostic tests and imaging
Loveridge et al 1998	Policy planning	Practical use of drugs modulating cellular systems to treat cancer	21	Treatment and management of symptoms and disease	II - cancers	Drugs (not prophylactic vaccines)
Loveridge et al 1998	Policy planning	Widespread use of nutritional supplements or special diets based on an understading of the role of nutrients such as anti-oxidants in cancer prevention	21	Health promotion and prevention of disease	II - cancers	Population programmes
Loveridge et al 1998	Policy planning	Practical use of substances that contain rather eleminate cancer (e.g. use agents that control metastasis)	21	Treatment and management of symptoms and disease	II - cancers	Drugs (not prophylactic vaccines)
Loveridge et al 1998	Policy planning	New methods of treating arterial occlusion and myocardial infarction dramatically reduce morbidity from cardiovascular diseases in 40-50 age group	21	Treatment and management of symptoms and disease	IX - circulation	Unknown

For peer review only - http://bmjopen.bmj.com/site/about/guidelines.xhtml

Appendix1: List of identified healthcare technologies

Loveridge et al 1998	Policy planning	50% of the UK population receive individual risk assessment for cardiovascular disease based on genetic screening and lifestyle measures	21	Health promotion and prevention of disease	IX - circulation	Population programmes
Loveridge et al 1998	Policy planning	Understanding the immune system is sufficietly developed to allow routine transplantation without the use of immuno-suupression	21	Treatment and management of symptoms and disease	III - blood	Therapeutic procedures
Loveridge et al 1998	Policy planning	Practical use of xenotransplantation of organs from animals to human	21	Treatment and management of symptoms and disease	Other - generic	Regenerative medicine
Loveridge et al 1998	Policy planning	Incidence of each of the 3 major viral diseases is reduced by 90% by new vaccines or drug development	21	Health promotion and prevention of disease	I - infections	Prophylactic vaccines
Loveridge et al 1998	Policy planning	Practical use of technologies for oral administration of multi-valent vaccines for the most important childhood illnesses	21	Health promotion and prevention of disease	I - infections	Prophylactic vaccines
Loveridge et al 1998	Policy planning	Widespread introduction of malaria vaccine reduces the worldwide incidence of the diseases by 50%	21	Health promotion and prevention of disease	I - infections	Prophylactic vaccines
Loveridge et al 1998	Policy planning	First effective vaccine of HIV becomes available	21	Health promotion and prevention of disease	I - infections	Prophylactic vaccines
Loveridge et al 1998	Policy planning	Development of drugs with proven	21	Treatment and management of	V - mental health	Drugs (not prophylactic

For peer review only - http://bmjopen.bmj.com/site/about/guidelines.xhtml

Appendix1: List of identified healthcare technologies

		efficacy for enhancing cognitive performance		symptoms and disease		vaccines)
Loveridge et al 1998	Policy planning	50% of patients presenting with mental illness such as schizphrenia and depression are diagnosed using objective genetic, biochemical or biophysical tests	21	Diagnosis and monitoring of disease	V - mental health	Diagnostic tests and imaging
Loveridge et al 1998	Policy planning	50% of surgical interventions carried out by minimum-access technique	21	Treatment and management of symptoms and disease	Other - generic	Therapeutic procedures
Loveridge et al 1998	Policy planning	10% of surgical interventions are carried out by robotic techniques	21	Treatment and management of symptoms and disease	Other - generic	Therapeutic procedures
Loveridge et al 1998	Policy planning	Development of biocompatible materials for implants which elicit the required host response (e.g. non-thrombogenic or stimulation of host repair mechanisms).	21	Treatment and management of symptoms and disease	Other - generic	Devices and biomaterials
Loveridge et al 1998	Policy planning	Widespread use for skin replacemt and/or repair of reliable products based on tissue engineering	21	Treatment and management of symptoms and disease	XII - skin	Regenerative medicine
Loveridge et al 1998	Policy planning	Practical availability for regular clinical use of functional human organ tissue for kidney, liver or pancreas	21	Treatment and management of symptoms and disease	Other - generic	Regenerative medicine
Loveridge et al 1998	Policy planning	Widespread use of non-surgical (eg. by	21	Health promotion and prevention of disease	XXI - screening, rehab	Drugs (not prophylactic vaccines)

For peer review only - http://bmjopen.bmj.com/site/about/guidelines.xhtml

Appendix1: List of identified healthcare technologies

		vaccination) single application contraception offering lifetime control				
Loveridge et al 1998	Policy planning	50% of contraception involving pharmacological or vaccine methods resides with men	21	Health promotion and prevention of disease	XXI - screening, rehab	Drugs (not prophylactic vaccines)
Loveridge et al 1998	Policy planning	50% of diagnostic and treatment decisions involve use of expert systems	21	Supporting patients receiving care (designed to be used by individuals)	Other - generic	IT systems, eHealth and mHealth
Loveridge et al 1998	Policy planning	20% of time spent by doctors in primary care screeing is replaced by patient interaction with experts systems	21	Supporting patients receiving care (designed to be used by individuals)	Other - generic	IT systems, eHealth and mHealth
Loveridge et al 1998	Policy planning	Practical use of remote electronic systems which substitute human labour in montoring the physical parameter of elderly and people with disability in the community	21	Supporting patients receiving care (designed to be used by individuals)	Other - generic	IT systems, eHealth and mHealth
Loveridge et al 1998	Policy planning	Widespread use of unified and standardised electronic data collection systems and data informats for medical records	21	Supporting the provision of care and/or increasing service efficiency	Other - generic	IT systems, eHealth and mHealth
Loveridge et al 1998	Policy planning	At least 50% of health care centres (hospitals, clinic,etc) are connected to national information	21	Supporting the provision of care and/or increasing service efficiency	Other - generic	IT systems, eHealth and mHealth

For peer review only - http://bmjopen.bmj.com/site/about/guidelines.xhtml

Appendix1: List of identified healthcare technologies

		electronic networks for patient records and information on diagnosis, treatment and outcome				
Loveridge et al 1998	Policy planning	Widespread use in health care of electronic information systems which span existing professional and organisational boundries (health, social service, housing etc)	21	Supporting the provision of care and/or increasing service efficiency	Other - generic	IT systems, eHealth and mHealth
Loveridge et al 1998	Policy planning	Improved understanding of the role of gene-environment interactions in causing common structural birth birth defects (e.g. cleft palate) permits the implementation of primary preventable programmes	21	Health promotion and prevention of disease	XXI - screening, rehab	Population programmes
Loveridge et al 1998	Policy planning	Understanding how environmental factors encountered in early life cause adult disease leads to national standards for diet and lifestyle in pre-natal and early life	21	Health promotion and prevention of disease	XXI - screening, rehab	Population programmes
Loveridge et al 1998	Policy planning	Widespread use of financial inducements by national and private health insurers or similar bodies to adopt	21	Health promotion and prevention of disease	Other - generic	Organisational programmes

For peer review only - http://bmjopen.bmj.com/site/about/guidelines.xhtml

Appendix1: List of identified healthcare technologies

		healthier lifestyle or diets				
Loveridge et al 1998	Policy planning	The causes of the rising prevalence of asthma are elucidated and action based on this knowledge leads to a 50% reduction in the incidence of severe disease	21	Health promotion and prevention of disease	X - respiratory	Population programmes
Operating theatre of the year 2010; Department of Trade and Industry	Policy planning	X-ray replaced by Magnetic Resonance Imaging (MRI)	14	Diagnosis and monitoring of disease	Other - generic	Diagnostic tests and imaging
Operating theatre of the year 2010; Department of Trade and Industry	Policy planning	operating theatre of 2010 will be built with an ultra-open MRI system with parallel ultrasound facilities and complete optical capability	14	Supporting the provision of care and/or increasing service efficiency	Other - generic	Medical equipment
Operating theatre of the year 2010; Department of Trade and Industry	Policy planning	Endoscopes will reduce in size as fibres, lenses and electronic sensors get smaller,	14	Supporting patients receiving care (designed to be used by individuals)	Other - generic	Devices and biomaterials
Operating theatre of the year 2010; Department of Trade and Industry	Policy planning	CCD image sensors have the ability to see in both ultra-violet and near infra-red.	14	Diagnosis and monitoring of disease	Other - generic	Diagnostic tests and imaging
Operating theatre of the year 2010; Department of Trade and Industry	Policy planning	New magnetic sensing technologies will detect the configuration of the endoscope and accessory devices inside the patient.	14	Supporting patients receiving care (designed to be used by individuals)	Other - generic	Devices and biomaterials

For peer review only - http://bmjopen.bmj.com/site/about/guidelines.xhtml

Appendix1: List of identified healthcare technologies

Operating theatre of the year 2010; Department of Trade and Industry	Policy planning	Auto focus and zoom optical systems will be interfaced with 30 to take the endoscope imaging capability beyond what is currently possible with human vision.	14	Diagnosis and monitoring of disease	Other - generic	Diagnostic tests and imaging
Operating theatre of the year 2010; Department of Trade and Industry	Policy planning	High resolution ultrasound in closer proximity to the tissue and structures to be studied	14	Diagnosis and monitoring of disease	Other - generic	Diagnostic tests and imaging
Operating theatre of the year 2010; Department of Trade and Industry	Policy planning	Laser induced fluorescence imaging systems for diagnosis of early malignant changes, monitoring and therapy will become available.	14	Diagnosis and monitoring of disease	II - cancers	Diagnostic tests and imaging
Operating theatre of the year 2010; Department of Trade and Industry	Policy planning	Near infra-red spectroscopy, for looking at tissue oxygenation state	14	Diagnosis and monitoring of disease	Other - generic	Diagnostic tests and imaging
Operating theatre of the year 2010; Department of Trade and Industry	Policy planning	Confocal, or other optical, ultrasonic or magnetic resonance microscopy, for on-line histology of bulk samples	14	Diagnosis and monitoring of disease	Other - generic	Diagnostic tests and imaging
Operating theatre of the year 2010; Department of Trade and Industry	Policy planning	Minimal Access Surgery is likely to account for as much as 75% of surgical procedures	14	Treatment and management of symptoms and disease	Other - generic	Therapeutic procedures
Operating theatre of the year 2010; Department of Trade and Industry	Policy planning	Robotic camera holders are likely to become routine in laparoscopic procedures.	14	Treatment and management of symptoms and disease	Other - generic	Medical equipment

For peer review only - http://bmjopen.bmj.com/site/about/guidelines.xhtml

Appendix1: List of identified healthcare technologies

Operating theatre of the year 2010; Department of Trade and Industry	Policy planning	The operating table's control system and the robotics system could become integrated in the future operating table.	14	Supporting the provision of care and/or increasing service efficiency	Other - generic	Medical equipment
Operating theatre of the year 2010; Department of Trade and Industry	Policy planning	Ability to ventilate all patients from neonates to adults	14	Treatment and management of symptoms and disease	X - respiratory	Non-surgical therapy
Operating theatre of the year 2010; Department of Trade and Industry	Policy planning	Control handsets will incorporate dot matrix displays which will give the operator information about the condition of the table, position of the top and warnings about possible hazards	14	Supporting the provision of care and/or increasing service efficiency	Other - generic	Medical equipment
Operating theatre of the year 2010; Department of Trade and Industry	Policy planning	The operating table may have a built-in patient temperature regulation system.	14	Supporting the provision of care and/or increasing service efficiency	Other - generic	Medical equipment
Operating theatre of the year 2010; Department of Trade and Industry	Policy planning	Connection between the Operating Room and the Anaesthetic machine in the Control Room will be via the Anaesthetist in the Operating Room.	14	Supporting the provision of care and/or increasing service efficiency	Other - generic	Medical equipment
Operating theatre of the year 2010; Department of Trade and Industry	Policy planning	The Anaesthetic machine will incorporate both inhalation agent vaporisation and multi-drug infusion systems These devices will be electronically integrated into the monitoring	14	Supporting the provision of care and/or increasing service efficiency	Other - generic	Medical equipment

For peer review only

Appendix1: List of identified healthcare technologies

		and information management and anaesthetic record keeping system so that vaporiser concentration settings and infusion rates will be continuously recorded				
Operating theatre of the year 2010; Department of Trade and Industry	Policy planning	Mechanical flow-meters will be replaced by electronic gas mixing systems	14	Supporting the provision of care and/or increasing service efficiency	Other - generic	Medical equipment
Operating theatre of the year 2010; Department of Trade and Industry	Policy planning	Patient monitoring will be very simple and intuitive probably touch screen and quite possibly voice activation.	14	Supporting patients receiving care (designed to be used by individuals)	Other - generic	IT systems, eHealth and mHealth
Operating theatre of the year 2010; Department of Trade and Industry	Policy planning	Monitors will also network into central areas outside the operating theatre so that, for example, anaesthetics being delivered by less experienced staff can be monitored remotely.	14	Supporting patients receiving care (designed to be used by individuals)	Other - generic	IT systems, eHealth and mHealth
Operating theatre of the year 2010; Department of Trade and Industry	Policy planning	The operation room will capture the data from one or perhaps two small 'wireless' sensors placed on the body that will provide pulse oxyimetry C02, ECG and other key parameter data and then transmit them to the monitor via radio or IR technology.	14	Supporting patients receiving care (designed to be used by individuals)	Other - generic	IT systems, eHealth and mHealth

For peer review only

For peer review only - http://bmjopen.bmj.com/site/about/guidelines.xhtml

Appendix1: List of identified healthcare technologies

Operating theatre of the year 2010; Department of Trade and Industry	Policy planning	Patient will be equipped with a belt (or other suitable garment) into which all monitoring and feed lines will be terminated, and which will include significant active capability. Drip and other lines will also be terminated in the belt	14	Supporting patients receiving care (designed to be used by individuals)	Other - generic	Devices and biomaterials
Operating theatre of the year 2010; Department of Trade and Industry	Policy planning	Patient can be "plugged" into electrical support, monitoring and stimulation as well as fluid and chemical agents using a single connector to facilitate transfer of patient from one ward to another or from the operation theatre	14	Supporting patients receiving care (designed to be used by individuals)	Other - generic	IT systems, eHealth and mHealth
Operating theatre of the year 2010; Department of Trade and Industry	Policy planning	Operation lamp will be replaced by a better light source	14	Supporting the provision of care and/or increasing service efficiency	Other - generic	Medical equipment
Operating theatre of the year 2010; Department of Trade and Industry	Policy planning	Automatic control of light pattern in the operation theatre	14	Supporting the provision of care and/or increasing service efficiency	Other - generic	Medical equipment
Operating theatre of the year 2010; Department of Trade and Industry	Policy planning	The operating lamp of the future is likely to use much more electronics to accurately control the voltage, dimming, focus, tracking and to limit maximum light intensity to	14	Supporting the provision of care and/or increasing service efficiency	Other - generic	Medical equipment

For peer review only - http://bmjopen.bmj.com/site/about/guidelines.xhtml

Appendix1: List of identified healthcare technologies

		safe levels.				
Operating theatre of the year 2010; Department of Trade and Industry	Policy planning	The operating lamps of the future will be sealed for easy disinfection	14	Supporting the provision of care and/or increasing service efficiency	Other - generic	Medical equipment
Operating theatre of the year 2010; Department of Trade and Industry	Policy planning	Telemedicine	14	Unknown	Other - generic	IT systems, eHealth and mHealth
Operating theatre of the year 2010; Department of Trade and Industry	Policy planning	Telemonitoring	14	Supporting the provision of care and/or increasing service efficiency	Other - generic	IT systems, eHealth and mHealth
Operating theatre of the year 2010; Department of Trade and Industry	Policy planning	Teleconsultation	14	Supporting the provision of care and/or increasing service efficiency	Other - generic	IT systems, eHealth and mHealth
Operating theatre of the year 2010; Department of Trade and Industry	Policy planning	Telesurgery for demonstartion or teaching	14	Supporting the provision of care and/or increasing service efficiency	Other - generic	IT systems, eHealth and mHealth
Operating theatre of the year 2010; Department of Trade and Industry	Policy planning	Minimal access surgery (MAS)	14	Treatment and management of symptoms and disease	Other - generic	Therapeutic procedures
Operating theatre of the year 2010; Department of Trade and Industry	Policy planning	Systems in which as much of the patient's own blood is recovered and recycled as possible.	14	Treatment and management of symptoms and disease	III - blood	Non-surgical therapy
Operating theatre of the year 2010; Department of Trade and Industry	Policy planning	Use of flexible endoscope technology to allow surgeons to see around and behind structures, giving the surgeon greater access and the patient a greater degree of safety.	14	Treatment and management of symptoms and disease	XI - GI tract, dental health	Devices and biomaterials

For peer review only - http://bmjopen.bmj.com/site/about/guidelines.xhtml

Appendix1: List of identified healthcare technologies

Operating theatre of the year 2010; Department of Trade and Industry	Policy planning	A move away from the use of X-rays and image intensification towards more mobile methods of MRI scanning.	14	Diagnosis and monitoring of disease	Other - generic	Diagnostic tests and imaging
Operating theatre of the year 2010; Department of Trade and Industry	Policy planning	Developments in the use of laser and particularly electrosurgery	14	Treatment and management of symptoms and disease	Other - generic	Therapeutic procedures
Operating theatre of the year 2010; Department of Trade and Industry	Policy planning	Use of robotics and head up display allowing surgeon sitting in a control room rather than being in the theatre with the patient	14	Supporting the provision of care and/or increasing service efficiency	Other - generic	Medical equipment
Operating theatre of the year 2010; Department of Trade and Industry	Policy planning	Coding will be used to associate patient, operating procedure (and so appropriate tools and drugs), support facilities and to generate automatic records of usage.	14	Supporting the provision of care and/or increasing service efficiency	Other - generic	IT systems, eHealth and mHealth
Operating theatre of the year 2010; Department of Trade and Industry	Policy planning	Tools and devices for a procedure will be located in a carousel analogous to that for a computer numerically controlled machine tool. This will allow the surgeon to present his tool holder to it, have the old tool extracted and a new one inserted.	14	Supporting the provision of care and/or increasing service efficiency	Other - generic	Medical equipment
Operating theatre of the year 2010; Department of Trade and Industry	Policy planning	Robots will be used as transport vehicles both within the operating room	14	Supporting the provision of care and/or increasing service efficiency	Other - generic	Medical equipment

For peer review only - http://bmjopen.bmj.com/site/about/guidelines.xhtml

Appendix1: List of identified healthcare technologies

		and for the surrounding support areas				
Operating theatre of the year 2010; Department of Trade and Industry	Policy planning	Robots will carry out very precise procedures controlled by the surgeon from a distance,	14	Supporting patients receiving care (designed to be used by individuals)	Other - generic	Medical equipment
Operating theatre of the year 2010; Department of Trade and Industry	Policy planning	Joint replacement be performed robotically	14	Treatment and management of symptoms and disease	XIII - musculoske letal	Therapeutic procedures
Operating theatre of the year 2010; Department of Trade and Industry	Policy planning	The operation theatre may be controlled by a robot "software" box th at will respond to voice recognition of the surgeon and change factors such as the heating of the theatre, the lighting and position of the table	14	Supporting the provision of care and/or increasing service efficiency	Other - generic	Medical equipment
Operating theatre of the year 2010; Department of Trade and Industry	Policy planning	A robot would be able to transfer sterile instrument trays from a central sterile store to the surgeon at the operating table.	14	Supporting the provision of care and/or increasing service efficiency	Other - generic	Medical equipment
Operating theatre of the year 2010; Department of Trade and Industry	Policy planning	Transport Robots could be used in the supply and disposal functions outside the operating room within the operating suite	14	Supporting the provision of care and/or increasing service efficiency	Other - generic	Medical equipment
Operating theatre of the year 2010; Department of Trade and Industry	Policy planning	Robots will be used in moving patients from ward to theatre areas (to replace the use of porters)	14	Supporting the provision of care and/or increasing service efficiency	Other - generic	Medical equipment

For peer review only - http://bmjopen.bmj.com/site/about/guidelines.xhtml

Appendix1: List of identified healthcare technologies

Operating theatre of the year 2010; Department of Trade and Industry	Policy planning	Using dedicated robot trucks similar to the supply trucks, waste could be collected on a regular basis from each theatre and taken to an automatic local waste reduction/ disposal facility adjacent to the theatre suite.	14	Supporting the provision of care and/or increasing service efficiency	Other - generic	Medical equipment
Operating theatre of the year 2010; Department of Trade and Industry	Policy planning	Disposal of hospital material could be performed on site with the use of machines such as "needle eaters"	14	Supporting the provision of care and/or increasing service efficiency	Other - generic	Medical equipment
Operating theatre of the year 2010; Department of Trade and Industry	Policy planning	Bulky X-Ray machines will probably be a thing of the past and replaced by computerised radiology	14	Diagnosis and monitoring of disease	Other - generic	Medical equipment
Operating theatre of the year 2010; Department of Trade and Industry	Policy planning	A central control room, with visual links to each theatre served, could house all of the equipment.	14	Supporting the provision of care and/or increasing service efficiency	Other - generic	Medical equipment
Operating theatre of the year 2010; Department of Trade and Industry	Policy planning	All theatres could be designed with ultra-clean ventilation systems or an adapted containment system to reduce the risk of the spread of infection	14	Health promotion and prevention of disease	I - infections	Medical equipment
Operating theatre of the year 2010; Department of Trade and Industry	Policy planning	Patients identification bracelets could be coded and bar checked as they moved through	14	Supporting patients receiving care (designed to be used by individuals)	Other - generic	Medical equipment

For peer review only - http://bmjopen.bmj.com/site/about/guidelines.xhtml

Appendix1: List of identified healthcare technologies

		the system so that their progress could be monitored at all times				
Operating theatre of the year 2010; Department of Trade and Industry	Policy planning	Automated trolleys and doors that can be electronically controlled by robots	14	Supporting the provision of care and/or increasing service efficiency	Other - generic	Medical equipment
UK technology and innovation futures for the 2020s, 2010	Policy planning	Further development of 3D imaging techniques.	10	Diagnosis and monitoring of disease	Other - generic	Diagnostic tests and imaging
UK technology and innovation futures for the 2020s, 2010	Policy planning	Fluorescenceassisted Resection and Exploration (FLARE) technologies, which combine both visible light imaging with near-infra red imaging of fluorescent biomarkers, can detect particular pathologies associated with tumours without the need for biopsy	10	Diagnosis and monitoring of disease	Other - generic	Diagnostic tests and imaging
UK technology and innovation futures for the 2020s, 2010	Policy planning	Colour X-ray or TEDDI imaging can be used to biopsy tissues by utilising scattered X-rays to build a spatially-resolved picture that can distinguish between different materials.	10	Diagnosis and monitoring of disease	Other - generic	Diagnostic tests and imaging
UK technology and innovation futures for the 2020s, 2010	Policy planning	In vivo neuroimaging, both at the tissue and cellular level	10	Diagnosis and monitoring of disease	Other - generic	Diagnostic tests and imaging
UK technology and innovation futures for the	Policy planning	Brain imaging techniques such as functional	10	Diagnosis and monitoring of disease	Other - generic	Diagnostic tests and imaging

For peer review only - http://bmjopen.bmj.com/site/about/guidelines.xhtml

Appendix1: List of identified healthcare technologies

2020s, 2010		magnetic resonance imaging (fMRI)				
UK technology and innovation futures for the 2020s, 2010	Policy planning	Lab-on-a-Chip (LOC) devices integrate and scale-down biomedical and other analytical laboratory functions and processes to a miniaturised chip format.	10	Diagnosis and monitoring of disease	Other - generic	Diagnostic tests and imaging
UK technology and innovation futures for the 2020s, 2010	Policy planning	Nucleic acid probes and diagnostic microarray systems can detect the nucleic acid sequence or sequences that identify a specific infectious disease agent, human genetic disease or individual (DNA profiling).	10	Diagnosis and monitoring of disease	Other - generic	Diagnostic tests and imaging
UK technology and innovation futures for the 2020s, 2010	Policy planning	A new focus on single-stranded DNA, micro RNA (miRNA) and double-stranded RNA (dsRNA) technologies as therapeutics may lead to clinically-approved interventions for gene therapy and treatment of medical disease.	10	Treatment and management of symptoms and disease	Other - generic	Regenerative medicine
UK technology and innovation futures for the 2020s, 2010	Policy planning	RNA interference (RNAi) may lead to new treatments.	10	Treatment and management of symptoms and disease	Other - generic	Regenerative medicine
UK technology and innovation futures for the 2020s, 2010	Policy planning	Viruses, bacteria or yeasts may be able to act as carriers for new sequences of DNA	10	Treatment and management of symptoms and disease	Other - generic	Regenerative medicine

For peer review only - http://bmjopen.bmj.com/site/about/guidelines.xhtml

Appendix1: List of identified healthcare technologies

UK technology and innovation futures for the 2020s, 2010	Policy planning	Increased understanding of individual sensitivities to drugs	10	Diagnosis and monitoring of disease	III - blood	Drugs (not prophylactic vaccines)
UK technology and innovation futures for the 2020s, 2010	Policy planning	DNA vaccines for AIDS, malaria, hepatitis B and certain cancers may be available within 10 years	10	Health promotion and prevention of disease	Other - generic	Prophylactic vaccines
UK technology and innovation futures for the 2020s, 2010	Policy planning	New treatments for for neurodegenerative conditions such as dementia.	10	Treatment and management of symptoms and disease	VI - nervous	Unknown
UK technology and innovation futures for the 2020s, 2010	Policy planning	'Cognition enhancers'; substances that enhance the performance of our brain in specific ways, such as improving short-term memory and increasing our speed of thought enhancement through lifestyle drugs may will become the norm.	10	Treatment and management of symptoms and disease	VI - nervous	Drugs (not prophylactic vaccines)
UK technology and innovation futures for the 2020s, 2010	Policy planning	'Emoticeuticals', drugs that act by changing hormone levels in the body, may mediate our responses in our private lives and work.	10	Treatment and management of symptoms and disease	V - mental health	Drugs (not prophylactic vaccines)
UK technology and innovation futures for the 2020s, 2010	Policy planning	Sensoceuticals' might enhance pleasure by restoring or accentuating the senses.	10	Treatment and management of symptoms and disease	Other - generic	Drugs (not prophylactic vaccines)
UK technology and innovation futures for the 2020s, 2010	Policy planning	The advent of a drug that mimics the positive effects of alcohol	10	Health promotion and prevention of disease	V - mental health	Drugs (not prophylactic vaccines)

For peer review only

Appendix1: List of identified healthcare technologies

		with none of its drawbacks.				
UK technology and innovation futures for the 2020s, 2010	Policy planning	Stem cells replacing malfunctioning liver cells or even growing entire livers to replace diseased ones	10	Treatment and management of symptoms and disease	XI - GI tract, dental health	Regenerative medicine
UK technology and innovation futures for the 2020s, 2010	Policy planning	Stem cells giving new neurons to those with brain damage	10	Treatment and management of symptoms and disease	VI - nervous	Regenerative medicine
UK technology and innovation futures for the 2020s, 2010	Policy planning	Stem cells giving new heart cells to those with heart disease	10	Treatment and management of symptoms and disease	IX - circulation	Regenerative medicine
UK technology and innovation futures for the 2020s, 2010	Policy planning	Stem cells giving therapies for Parkinson's and Alzheimer's diseases	10	Treatment and management of symptoms and disease	VI - nervous	Regenerative medicine
UK technology and innovation futures for the 2020s, 2010	Policy planning	Stem cells giving therapies for spinal cord injury, stroke, cancer, burns, cardiovascular disease, diabetes, osteoarthritis and rheumatoid arthritis; as well as furnishing an endless supply of red blood cells for transfusions.	10	Treatment and management of symptoms and disease	Other - generic	Regenerative medicine
UK technology and innovation futures for the 2020s, 2010	Policy planning	Development of a cheap anti-malarial drug.	10	Treatment and management of symptoms and disease	I - infections	Drugs (not prophylactic vaccines)
UK technology and innovation futures for the 2020s, 2010	Policy planning	Programming a bacterium or virus that can identify malignant cells and can deliver a therapeutic agent could have major benefits for treating cancer and similar illnesses	10	Treatment and management of symptoms and disease	II - cancers	Regenerative medicine

For peer review only - http://bmjopen.bmj.com/site/about/guidelines.xhtml

Appendix1: List of identified healthcare technologies

UK technology and innovation futures for the 2020s, 2010	Policy planning	Stratified medicine: it will become increasingly possible to analyse an individual for certain biomarkers to determine the best course of treatment	10	Diagnosis and monitoring of disease	Other - generic	Diagnostic tests and imaging
UK technology and innovation futures for the 2020s, 2010	Policy planning	Personalised medicine	10	Treatment and management of symptoms and disease	Other - generic	Drugs (not prophylactic vaccines)
UK technology and innovation futures for the 2020s, 2010	Policy planning	In-situ tissue regeneration: Material is implanted directly in the body and regenerates the tissue over time as the material degrades.	10	Treatment and management of symptoms and disease	Other - generic	Regenerative medicine
UK technology and innovation futures for the 2020s, 2010	Policy planning	Tissue engineering: Material can act as temporary scaffold for cell culture and tissue growth in vitro	10	Treatment and management of symptoms and disease	Other - generic	Regenerative medicine
UK technology and innovation futures for the 2020s, 2010	Policy planning	Micro-masonry and 3D organ printing techniques	10	Treatment and management of symptoms and disease	Other - generic	Regenerative medicine
UK technology and innovation futures for the 2020s, 2010	Policy planning	Brain Computer Interface	10	Rehabilitation	Other - generic	Devices and biomaterials
UK technology and innovation futures for the 2020s, 2010	Policy planning	Neuroprosthetics: the connection can occur between any part of the nervous system (such as peripheral nerves) and a device.	10	Rehabilitation	VI - nervous	Devices and biomaterials

For peer review only - http://bmjopen.bmj.com/site/about/guidelines.xhtml

Appendix1: List of identified healthcare technologies

UK technology and innovation futures for the 2020s, 2010	Policy planning	Magnetoencephalography (MEG): maps brain activity by recording the magnetic fields produced by electrical currents occurring in the brain	10	Diagnosis and monitoring of disease	VI - nervous	Diagnostic tests and imaging
UK technology and innovation futures for the 2020s, 2010	Policy planning	functional magnetic imaging (fMRI) – involves measuring changes in blood flow related to neural activity in the brain.	10	Diagnosis and monitoring of disease	VI - nervous	Diagnostic tests and imaging
UK technology and innovation futures for the 2020s, 2010	Policy planning	functional Near-Infrared Spectroscopy (fNIRS) for optical topography	10	Diagnosis and monitoring of disease	Other - generic	Diagnostic tests and imaging
UK technology and innovation futures for the 2020s, 2010	Policy planning	Electrocorticography (EoCG), measure the electrical activity of the brain using electrodes which are implanted inside the skull, but outside the brain tissue.	10	Diagnosis and monitoring of disease	VI - nervous	Diagnostic tests and imaging
UK technology and innovation futures for the 2020s, 2010	Policy planning	Implanted brain chips for controlling computers	10	Rehabilitation	XXI - screening, rehab	Devices and biomaterials
UK technology and innovation futures for the 2020s, 2010	Policy planning	Neural prosthetics replace lost limbs	5	Rehabilitation	XXI - screening, rehab	Devices and biomaterials
UK technology and innovation futures for the 2020s, 2010	Policy planning	Thought-controlled robots for personal healthcare	10	Supporting patients receiving care (designed to be used by individuals)	Other - generic	Medical equipment
UK technology and innovation futures for the 2020s, 2010	Policy planning	Thought-controlled wheelchairs	10	Rehabilitation	XXI - screening, rehab	Medical equipment

For peer review only

For peer review only - http://bmjopen.bmj.com/site/about/guidelines.xhtml

Appendix1: List of identified healthcare technologies

UK technology and innovation futures for the 2020s, 2010	Policy planning	ehealth: Examples include health information networks, electronic health records, tele-medicine services, personal wearable and portable mobile devices, health portals and other tools that assist in health monitoring, diagnosis and treatment.	10	Supporting the provision of care and/or increasing service efficiency	Other - generic	IT systems, eHealth and mHealth
UK technology and innovation futures for the 2020s, 2010	Policy planning	Lab-on-a-chip technologies could also bring diagnostics down to a personal scale, where patients can test themselves and transmit data electronically for analysis and treatment.	10	Supporting patients receiving care (designed to be used by individuals)	Other - generic	IT systems, eHealth and mHealth
UK technology and innovation futures for the 2020s, 2010	Policy planning	Tailored Medicine	10	Treatment and management of symptoms and disease	Other - generic	Drugs (not prophylactic vaccines)
Technology foresight towards 2020_China	Policy planning	The anti-viral medicine with highly efficiency applies to clinical medicine widely	17	Treatment and management of symptoms and disease	I - infections	Drugs (not prophylactic vaccines)
Technology foresight towards 2020_China	Policy planning	standardized safe evaluation system which include the technology for monitoring and examination of trans-gene biology will be established;	17	Supporting the provision of care and/or increasing service efficiency	Other - generic	Organisational programmes

For peer review only - http://bmjopen.bmj.com/site/about/guidelines.xhtml
